# Global distribution, pathogen spectrum, and invasion potential of *Hyalomma anatolicum* at the human-livestock interface: A systematic review, meta-analysis, and ecological niche modeling

**DOI:** 10.1016/j.onehlt.2026.101385

**Published:** 2026-03-13

**Authors:** Shuo Zhou, Qing Xu, Jing Liu, Jia-Yi Pan, Xiao-Yang Wang, Hui-Jun Yu, Bing-Bing Gu, Bao-Yu Wang, Guo-Yao Zu, Chen Shan, Wu-Chun Cao, Lin Zhao

**Affiliations:** a Institute of EcoHealth, School of Public Health, Cheeloo College of Medicine, Shandong University, Jinan, China; b State Key Laboratory of Pathogen and Biosecurity, Beijing Institute of Microbiology and Epidemiology, Beijing, China

**Keywords:** *Hyalomma anatolicum*, One health, Tick-borne diseases, Livestock, Ecological niche modeling, Global distribution, Pathogen spectrum

## Abstract

*Hyalomma anatolicum* is an efficient vector at the human-livestock interface, yet its global ecological profile remains insufficiently defined. This study provides a comprehensive analysis by integrating data from a systematic review, meta-analysis, and ecological niche modeling (ENM) to reveal its distribution, host associations, pathogen spectrum, and expansion potential. Following PRISMA 2020 guidelines, we systematically searched international literature databases and the Global Biodiversity Information Facility (GBIF) for records up to April 2024. We mapped the global distribution of *H. anatolicum* and associated pathogens, identified key environmental determinants using machine learning, and projected potential invasion risks. Our findings showed that the distribution of *H. anatolicum* was predominantly clustered within anthropogenic landscapes, specifically urban and agricultural zones, creating a direct interface for zoonotic transmission. We demonstrated a specialized association with domestic livestock, particularly cattle, which anchored the vector's dispersal to global husbandry and trade systems rather than wildlife cycles. *H. anatolicum* was identified as a pathogen-rich vector harboring at least 66 microbial species, including high-prevalence pathogens such as Crimean-Congo hemorrhagic fever virus (CCHFV) and *Theileria annulata*. The spatial overlap between pathogen hotspots in *H. anatolicum* and its hosts and national human case reports confirms its role as a key zoonotic vector. Furthermore, ENM projections identified extensive suitable habitats in non-endemic regions, including parts of Australia and the western and eastern United States, driven by land cover and temperature. Transboundary One Health surveillance and livestock-focused control strategies for *H. anatolicum* should be strengthened to mitigate its global expansion.

## Highlights

• *Hyalomma anatolicum* Concentrated in human-dominated landscapes (urban & agricultural).
• 66 species of microbes could be carried by *H. anatolicum*, posing a dual threat to public health and the livestock economy.
• Ecological niche models warn of high invasion risks in regions like Australia and the USA, a global biosecurity threat.

## Graphical abstract

**Figure:**
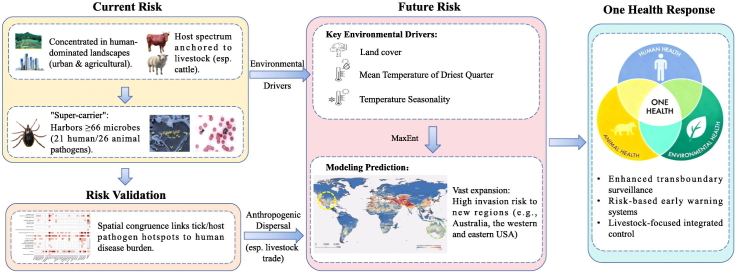

## 1 Introduction

Tick-borne diseases (TBDs) impose a multi-billion dollar burden on global public health and agriculture [1], [2]. *Hyalomma anatolicum* emerges as a high-impact vector bridging the livestock-human interface [3]. It serves as the primary vector for *Theileria annulata*—a parasite that impairs livestock productivity [4], [5], [6]—and a competent vector for Crimean-Congo hemorrhagic fever virus (CCHFV), a priority pathogen of the World Health Organization (WHO) with case fatality rate reaching up to 40% in humans [7], [8].

Despite its medical and veterinary significance, the global ecological profile of *H. anatolicum* remains poorly defined. Previous research has been largely confined to fragmented reports from specific countries or regions [9], [10], lacking the synthesis needed for a global threat assessment. Specifically, three core scientific questions persist: (1) the precise global distribution and its correlation with anthropogenic drivers like urbanization; (2) the full extent of its pathogen diversity and the reservoir hosts that maintain them; and (3) the potential for range expansion into non-endemic regions driven by climate change and land use change. These gaps impede robust global risk assessments and mitigation efforts.

To bridge these gaps, we synthesized global data through systematic review, meta-analysis, and ecological niche modeling (ENM). We defined distribution hotspots, quantified niche overlap with human-dominated environments, cataloged the pathogen spectrum, and projected invasion risks for new regions. These findings establish a rigorous baseline for transboundary surveillance and targeted control.

## 2 Methods

### 2.1 Data acquisition and systematic review

To construct a global-scale database for *H. anatolicum*, we conducted a systematic review integrating multi-source data. Two researchers independently searched PubMed, Web of Science, China National Knowledge Infrastructure (CNKI) and Wanfang Database for articles published from January 1, 1966, to April 1, 2024. The search term “*Hyalomma anatolicum*” (and its Chinese equivalent) was used. These results were supplemented with records from the specialized reference book, Fauna Sinica-Arachnida Ixodida, and the Global Biodiversity Information Facility (GBIF, https://www.gbif.org/) (accessed in April 2024).

A two-stage literature screening process was adopted, with inter-rater agreement quantified using Cohen's kappa coefficient (κ) [12]. Two investigators (SZ, JYP) independently screened titles, abstracts and full texts; discrepancies were resolved via discussion or consultation with a third investigator (LZ). Inclusion criteria were strictly defined as: (1) original research reporting the geographic distribution, animal hosts, or carried pathogens of *H. anatolicum*; and (2) articles published in English or Chinese. We excluded (3) duplicate publications, reviews, purely experimental studies, and irrelevant topics. All extracted data were cross-validated prior to entry into the standardized database. Details of the study's inclusion and exclusion procedures and information extraction are provided in Supplementary Data 1 (Fig. S1; table S1-S3). The study followed the Preferred Reporting Items of Systematic Reviews and Meta-Analyses (PRISMA 2020) guidelines [11]. The PRISMA checklist is provided in Supplementary Data 1 (table S5). All studies were imported into EndNote (version X9.3.3 (Bld 13,966)) and Microsoft Excel 2021 for automated and manual deduplication.

### 2.2 Environmental variables

To identify the ecological drivers of *H. anatolicum* distribution, 19 bioclimatic variables (BIO1-BIO19) and elevation data were obtained from the WorldClim database at a 10 km spatial resolution (https://www.worldclim.org). Topographic metrics, including slope and aspect, were derived from elevation data using ArcGIS 10.8. Global land cover data were integrated from the Resource and Environmental Science and Data Center (https://globalmaps.github.io/) (Supplementary Data 1 Table S6). All environmental layers were standardized to a uniform projection and resolution to ensure analytical consistency.

### 2.3 Geospatial mapping of known distribution

The geographic distribution of *H. anatolicum* and its associated pathogens was mapped using ArcGIS 10.8. For records lacking precise GPS coordinates, we used the centroid coordinates of the corresponding administrative level as a substitute.

### 2.4 Meta-analysis of pathogen prevalence

A meta-analysis was conducted to quantitatively analyze the pathogen infection rates associated with *H. anatolicum* and its hosts, respectively. Inclusion was restricted to original studies providing explicit denominators (total individuals tested). When only one study identified a specific pathogen species, the positive rate was calculated as the ratio of positive ticks to the total number of ticks tested, with no 95% confidence interval (CI) estimated. For pathogen species with two or more included studies, the pooled positive rate and corresponding 95% CI were computed using R software (version 4.4.1) with the “meta” package. Different pathogen-host associations reported within the same tick population were extracted as independent observational units, with their positivity rates calculated separately or used for subsequent meta-analysis. Interspecific heterogeneity was assessed via the *I*^*2*^ statistic. A random-effects model was employed when *I*^*2*^ > 50%, indicating significant heterogeneity; otherwise, a fixed-effects model was applied.

### 2.5 Geospatial congruence analysis of human infection threat

To evaluate the epidemiological alignment between environmental risk and reported human morbidity, we systematically retrieved published literature reports of human infection cases. These cases were (a) caused by the same key pathogens carried by *H. anatolicum* and its hosts (e.g., CCHFV, *Coxiella burnetii*) and (b) occurred within the known distribution countries of *H. anatolicum*. All extracted data were cross-validated prior to entry into the standardized database. Details of the literature search process and included studies are provided in Supplementary Data 1(Fig. S2; text S2; table S4). We emphasize that these cumulative figures serve as “evidence of threat” rather than absolute case counts; inherent reporting biases and heterogeneities in national surveillance sensitivity preclude formal estimates of population-level incidence or prevalence.

Our analytical objective was spatial congruence validation. Consequently, the extracted literature-reported case numbers were aggregated by country and categorized into different “threat evidence levels” (e.g., 0 cases, 1–100, 101–1000, 1001–10,000, >10,000). This stratification was visualized in Fig. 5C, aiming to intuitively reveal the degree of geographic overlap between the “tick-host-pathogen” circulation hotspots (Fig. 5A and B) and the “published human disease burden evidence” (Fig. 5C).

### 2.6 Modeling potential distribution

The global potential distribution of *H. anatolicum* was modeled using the Maximum Entropy (MaxEnt, v3.4.1) algorithm [13], [14]. The final model was constructed based on 268 high-precision occurrence records and a refined subset of environmental predictors, ensuring adherence to PRISMA 2020 guidelines [11]. Occurrence data and standardized environmental factor raster layers are provided in Supplementary Data 1 Table S7, Supplementary Data 2 and Supplementary Data 5, respectively. To mitigate spatial autocorrelation, occurrence data were processed in ENMTools (v1.4.4) through the elimination of duplicate records and spatial rarefaction.

To minimize overfitting due to multi-collinearity, we conducted a Pearson correlation analysis on the candidate variables; for any pair with |r| ≥ 0.8, the variable with the lower contribution rate was excluded. Model hyper-parameters, specifically regularization multipliers and feature classes, were optimized to achieve the best-fit configuration [15]. Model performance was evaluated using the mean Area Under the Receiver Operating Characteristic Curve (AUC) derived from 25 replicate runs. Final suitability projections were integrated into ArcGIS 10.8 for visualization and geographical analysis. Detailed modeling protocols and datasources are provided in Supplementary Data 1 (Text S3; table S7) and Supplementary Data 2.

## 3 Results

**Fig. 1:**
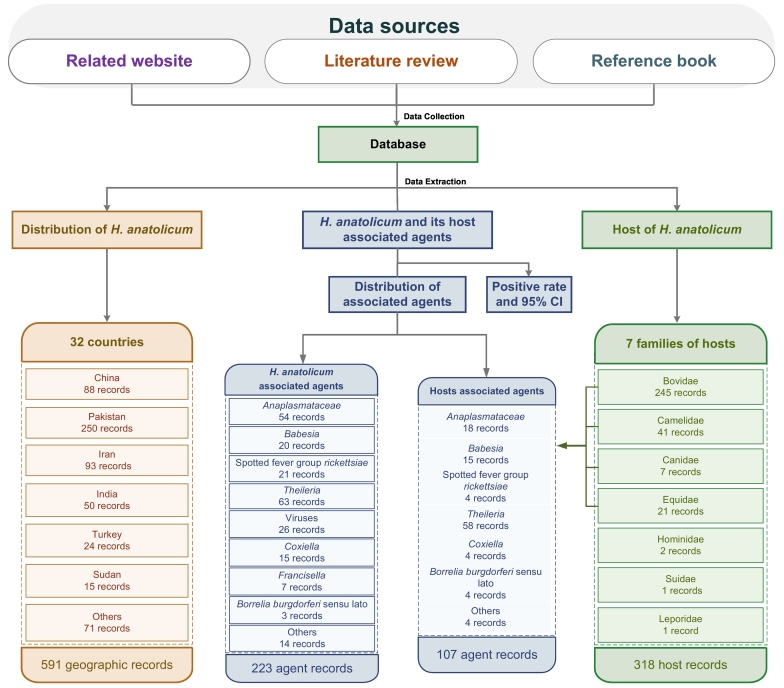
Research design and data compilation. Flowchart illustrating the systematic review process and the types and quantities of data collected on *H. anatolicum* distribution, hosts, and associated pathogens from reference book, published literature, and related website. Data were compiled from 32 endemic countries. *H. anatolicum* = *Hyalomma anatolicum* (see Supplementary Data 2–4).

A total of 361 original articles were included in this study (Supplementary Data 1 Text S1). There was substantial inter-researcher agreement in literature screening (Cohen's κ = 0.79, 95% CI: 0.76–0.82), indicating good reliability of the screening process. Our systematic integration of multi-source data yielded 591 geographic distribution records for *H. anatolicum* across 32 countries mainly in Asia, Africa, and Europe (Supplementary Fig. S3), identifying 7 host families and at least 66 associated microorganisms (Fig. 1; Supplementary Text S4; Supplementary Data 2–4).

### 3.1 Global geographical distribution of *H. anatolicum*

**Fig. 2:**
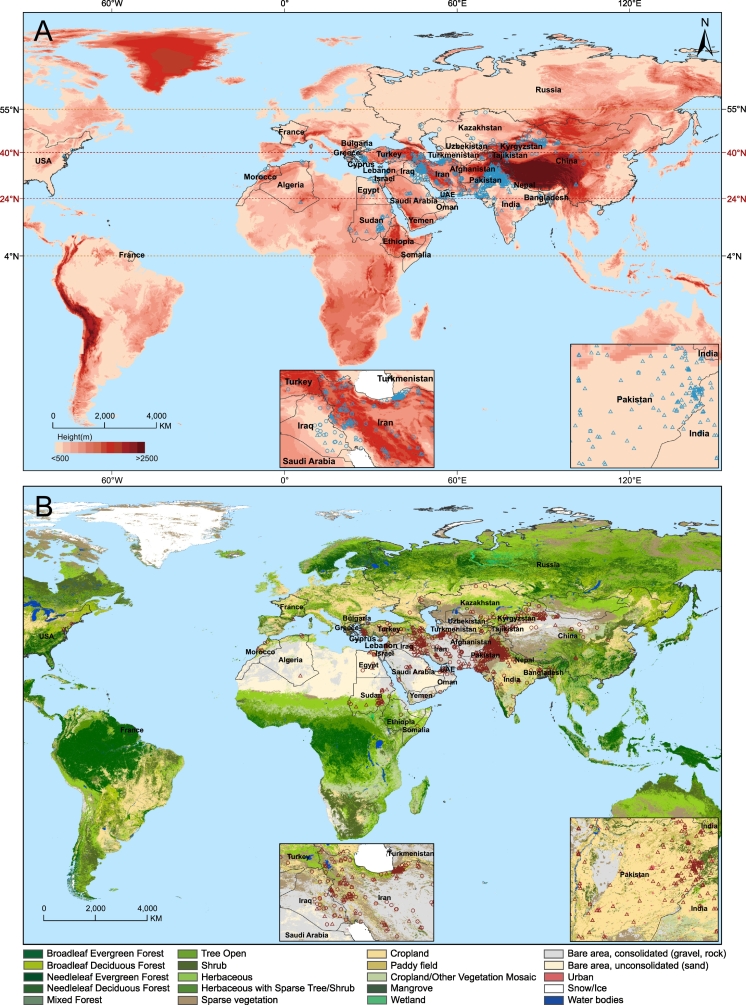
Global geographical distribution of *H. anatolicum*. (A) Distribution records overlaid on elevation. (B) Distribution records overlaid on land cover types. Unfilled triangles represent both records with precise coordinates and administrative centroids at the third-level administrative division. Unfilled circles represent administrative centroids at the second-level administrative division or higher. The analysis revealed a concentration between 24°N and 40°N, predominantly at low elevations and within urban and agricultural landscapes (see Supplementary Data 2).

Our geospatial analysis of the 591 records delineated the distribution of *H. anatolicum* across Asia, Africa, and Europe (Fig. 2; Supplementary Data 2). The species spans a wide latitudinal range from 4°N to 55°N but shows a pronounced concentration between 24°N and 40°N. Spatially, it occurrs in both coastal and inland regions, predominantly at low elevations (Fig. 2A). Notably, our analysis revealed a strong preference for human-modified environments: its distribution hotspots were significantly clustered in urban areas and agricultural landscapes (Fig. 2B). Detailed regional distributions, such as the hotspots in Pakistan and China, are provided in Supplementary Data 1 (Fig. S4). This finding reframed the species from a typical wildlife tick to a vector closely associated with human activity, forming the ecological basis of its public health threat.

### 3.2 Host spectrum of *H. anatolicum*

**Fig. 3:**
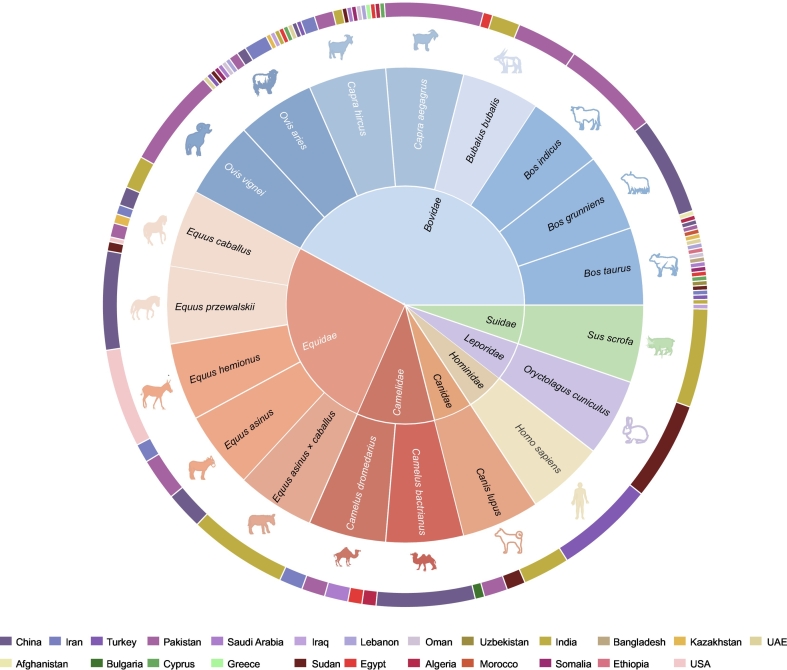
Host spectrum of *H. anatolicum* across different countries. Concentric circles display the host data: inner circle represents host families, middle circle represents host species (19 species from 7 families identified in total), and the outer circle indicates the 24 countries included in the host analysis where these species were reported (see Supplementary Data 4).

To investigate the ecological drivers behind the human-associated distribution, we next resolved its global host spectrum. Our analysis identified 19 distinct host species belonging to 7 families parasitized by *H. anatolicum* (Fig. 3; Supplementary Text S4; Supplementary Data 4). The analysis revealed a clear dependence on livestock. As visualized in Fig. 3, *Bos taurus* (domestic cattle) were the predominant host, recorded in 21 countries. Other major hosts, such as sheep, goats, and camels, are similarly core components of animal husbandry. In contrast, the tick was rarely documented on wildlife hosts. While national-level host diversity varied (highest in Pakistan), the overarching pattern of livestock dependence provides strong evidence that the global distribution of *H. anatolicum* is anchored in the global livestock production system, facilitating its colonizing and population persistence and dispersal.

### 3.3 Pathogens associated with *H. anatolicum* and its hosts

**Fig. 4:**
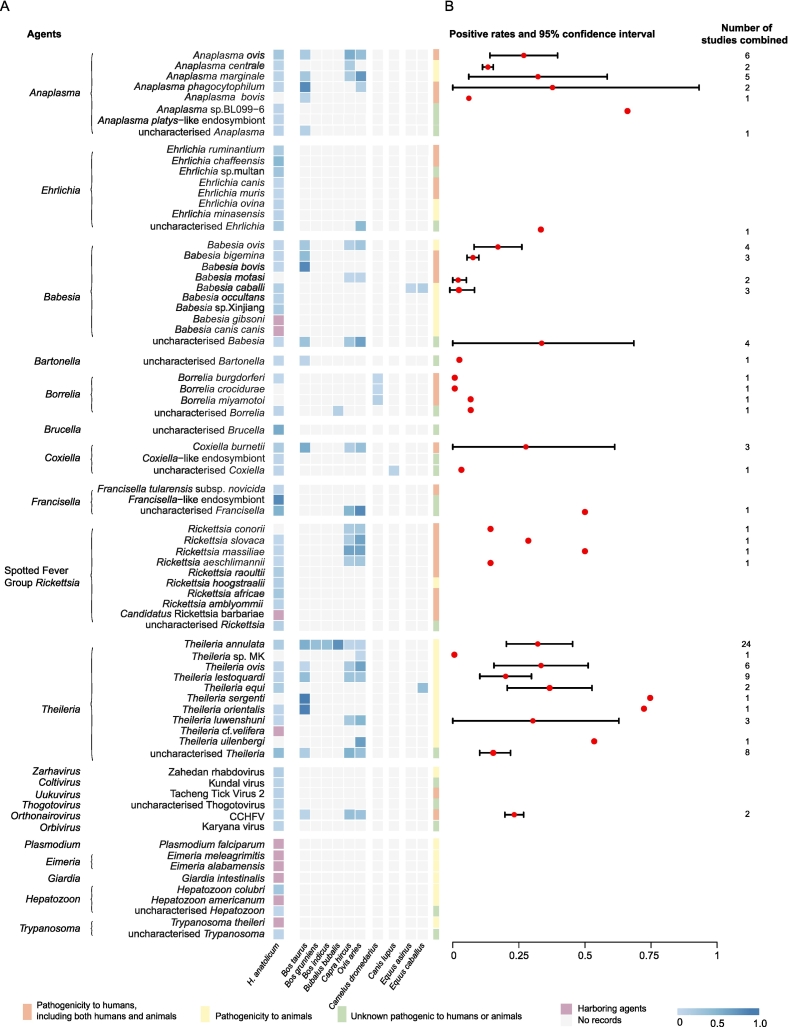
Meta-analysis of pathogen prevalence associated with *H. anatolicum* and its hosts. (A) Pooled prevalence of pathogens. For each pathogen listed (rows): the single leftmost grid column indicates detection in *H. anatolicum* ticks, while the 10 grid columns to the right indicate detection across its different host animal species (as labeled below). The color intensity within these grids reflects the pooled prevalence rate calculated via meta-analysis. (B) The plot on the far right displays the overall pooled prevalence estimate (dot) and 95% confidence interval (bar) for that pathogen aggregated across all host species combined. CCHFV=Crimean-Congo hemorrhagic fever virus (see Supplementary Data 3 and Data 4).

Our analysis identified at least 66 distinct microbial species in *H. anatolicum* samples, comprising 21 known human pathogens and 26 animal pathogens (Fig. 4A; Supplementary Text S4). The detailed aggregated dataset was provided in Supplementary Data 3.The meta-analysis quantified the prevalence of these agents. Data revealed the tick is a key vector of the economically significant *Theileria annulata*, with a high pooled prevalence of 11.83% (95% CI: 6.56–18.39). The tick also carries critical human pathogens, including CCHFV and *C. burnetii* (Fig. 4A; Supplementary Figs. S5 and S6).

Concurrently, the host animals (Fig. 4B) function as a major pathogen reservoir, carrying at least 36 distinct microbes, including 15 human pathogens and 13 animal pathogens. Pathogen prevalence in hosts was substantial (Supplementary Data 1 Fig. S7); for example, several *Theileria* species had positivity rates exceeding 20% (*T. sergenti* reached 74.67%). Notably, the pooled positivity rate for the key human pathogen CCHFV in its hosts was also high, at 24.39%. These data demonstrated that the tick and its livestock hosts form a persistent, high-risk reservoir posing a dual burden on public health and the livestock economy.

### 3.4 Geospatial analysis of pathogen distribution and human infection reports

**Fig. 5:**
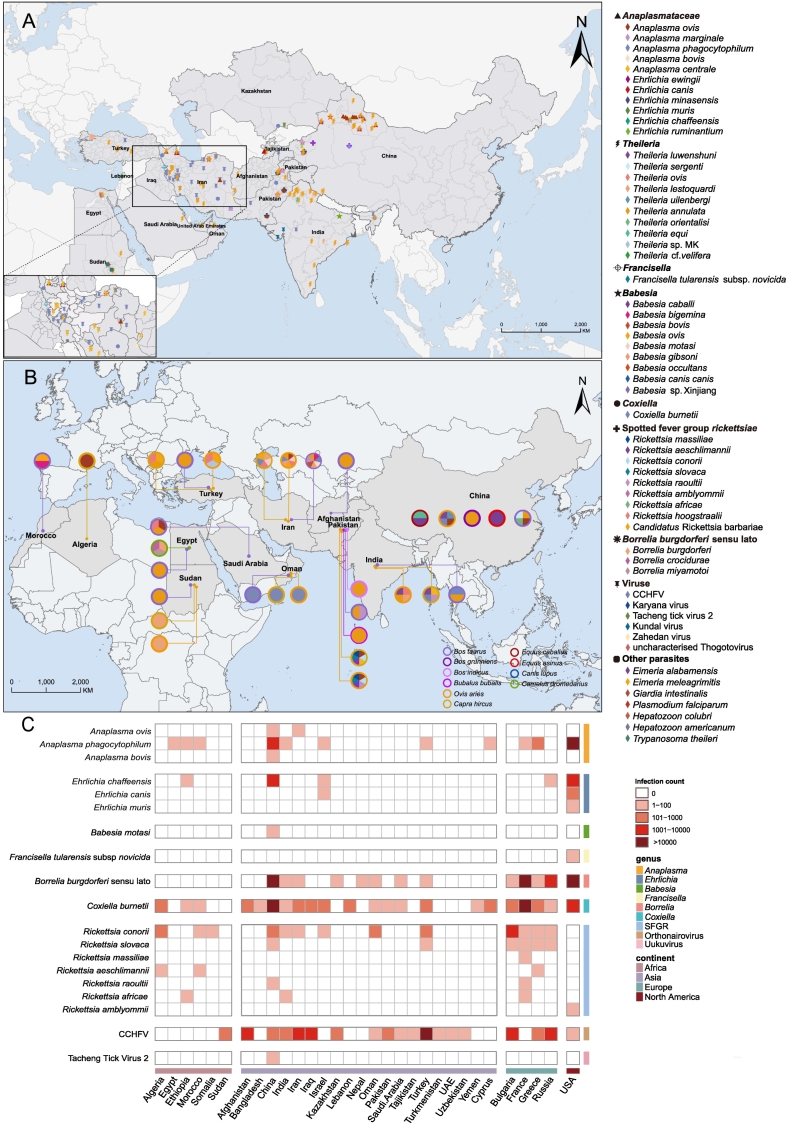
Spatial congruence analysis of pathogens in *H. anatolicum*, hosts, and associated human infection reports. (A) Geographical distribution of pathogens detected in *H. anatolicum* (see Supplementary Data 3). (B) Geographical distribution at the country level where pathogens were detected in host animals (see Supplementary Data 4). (C) Geographical distribution at the country level of cumulative reported human cases for key associated pathogens, categorized by count (0,1−100,101−1000,1001−10,000,〉10,000) based on literature review.

We next analyzed the geospatial distribution of these pathogens (Fig. 5A and B; Supplementary Data 3–4). In ticks, pathogen distribution was highly concentrated, with hotspots in Pakistan, Iran, and northwestern China. Pakistan exhibited the highest pathogen diversity (27 species), including CCHFV and *C. burnetii*, followed by northwestern China (16 species). *Theileria* was the most widespread pathogen group, reported in 10 countries, followed by viruses in 9 countries. In contrast, no pathogens were reported in *H. anatolicum* specimens from several European countries, including France, Greece, and Russia (Fig. 5A). In host animals, pathogens were also primarily detected in Asian countries. Hosts in Iran displayed the highest pathogen richness (14 species), including four *Anaplasmataceae*, four *Babesia*, three *Theileria* species, *C. burnetii* and CCHFV (Fig. 5B).

Finally, we assessed the spatial relationship at the country scale between this ecological risk and the reported human disease burden (Fig. 5C). In the tick's primary distribution range across Central Asia, West Asia, and North Africa, multiple associated pathogens have been detected in humans. Importantly, we found strong spatial congruence. In countries with high pathogen detection in ticks and hosts, such as China, Iran, and Pakistan, there was a clear geographic overlap with published reports of human infections. For example, in Pakistan, the co-occurrence of CCHFV and *C. burnetii* in local tick populations (Fig. 5A) and their corresponding human case reports (Fig. 5C) substantiates the functional “tick-host-human” transmission interface, identifying these regions as high-risk zones for zoonotic spillover.

### 3.5 Predictive modeling of potential suitable habitat for *H. anatolicum*

**Fig. 6:**
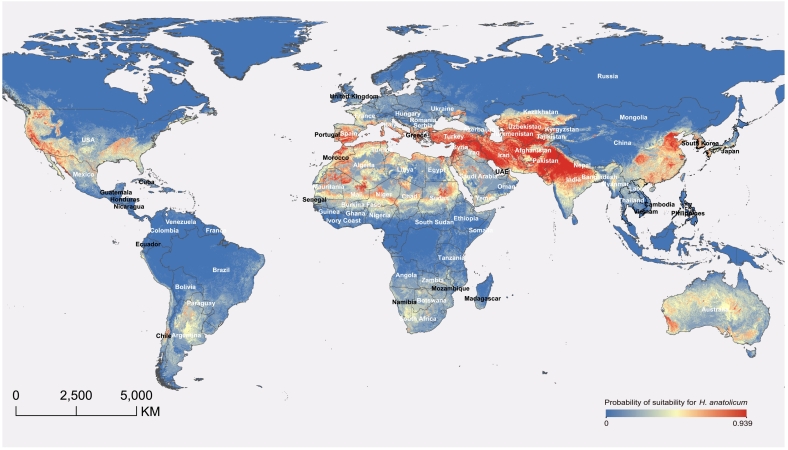
Predicted global potential distribution of *H. anatolicum*. Output map from the Maxent ecological niche model, predicting environmental suitability for *H. anatolicum*. The color gradient indicates the probability of suitability, ranging from low (blue) to high (red). (For interpretation of the references to color in this figure legend, the reader is referred to the web version of this article.)

The global potential distribution of *H. anatolicum* was projected using an ecological niche model (ENM) (Fig. 6). The parameters of the optimal model were: the regularization multiplier was 3.1 and the feature combination was LQP (linear, quadratic, product). The model demonstrated robust predictive performance, as evidenced by the high concordance between suitability projections and validated occurrence records and a mean test AUC of 0.966 ± 0.013 (Supplementary Data 1 Fig. S8). This high-resolution prediction was based on 268 validated precise distribution sites worldwide, with raster data for all environmental factors provided in Supplementary Data 5. Model analysis identified land cover, the mean temperature of the driest quarter (BIO9), and temperature seasonality (BIO4) as the primary environmental determinants shaping its potential distribution (Supplementary Data 1 Table S8). Compared with BIO9, land cover and BIO4, the contribution rates of the remaining bioclimatic variables, elevation, slope, and aspect were relatively lower (Supplementary Data 1 Fig. S9 and Table S8).

Model projections identified extensive suitable habitats across Asia and Africa, particularly encompassing Central, Western, and South Asia, as well as Mediterranean North Africa (Fig. 6). Significantly, the model revealed potential habitat suitability in several non-endemic regions, most notably in western and southern Australia, the western and eastern United States, and parts of Southern Europe (e.g., Spain). These findings indicate a substantial risk of geographic expansion into previously unrecognized high-risk zones, facilitated by favorable environmental conditions.

## 4 Discussion

This study integrates multi-source data with predictive modeling to define the global ecological niche and risk profile of *H. anatolicum*. By mapping its distribution, host associations, and pathogen diversity, we identified the drivers underlying its persistence and expansion potential. These findings provide an evidence-based foundation for vector management under the One Health framework.

Geospatial analysis indicates a distinct distribution pattern for *H. anatolicum*, with the species clustering in low-elevation anthropogenic landscapes—specifically urban and agricultural zones. Such a contrast with tick species that primarily inhabit natural environments [16], [17] reflects its close adaptation to human activity. This niche specialization positions the vector at the interface of human and livestock populations, potentially facilitating zoonotic spillover. Furthermore, land cover analysis (Supplementary Fig. S9 and Table S8) confirms that land use change expand suitable habitats for the tick. This relationship enables the vector to maintain transmission cycles in high-density anthropogenic settings, enhancing its public health significance.

Our analysis defines the ecological basis of *H. anatolicum* distribution as a strict dependence on domestic livestock. The host spectrum (Fig. 3) is centered on domestic ruminants—primarily cattle, sheep, goats, and camels—with minimal wildlife association [18], [19]. This specificity explains the vector's colonization of anthropogenic niches and identifies global livestock trade as its primary dispersal mechanism. While environmental factors limit habitat suitability, livestock transport drives long-distance movement across geographical barriers [20], [21], [22]. This interaction amplifies the risk of spatial expansion, posing a biosecurity threat at both regional and transcontinental levels.

Third, our analysis characterizes the extensive microbial diversity associated with *H. anatolicum*, identifying at least 66 distinct microbial species (Fig. 4A; Supplementary Data 1 Fig. S5). This microbial richness surpasses the documented diversity of other ecologically significant hard ticks, including *Haemaphysalis longicornis*, *Haemaphysalis concinna*, and *Ixodes persulcatus* [16], [17], [23]. These pathogens threaten both public and animal health. For instance, *H. anatolicum* serves as an important vector for CCHFV, which is associated with high human fatality rates. It also transmits *Theileria annulata*, the causative agent of tropical theileriosis, which imposes a substantial economic burden on the global livestock industry [24], [25], [26]. These findings identify the ‘tick-livestock’ interface as a persistent reservoir of diverse pathogens, necessitating integrated surveillance under the One Health framework.

Our spatial congruence analysis aligns the identified ecological risk hotspots with the documented human disease burden at the national level (Fig. 5). Pathogen distributions in tick populations and their hosts (Fig. 5A and B) show spatial overlap with published human infection reports (Fig. 5C), particularly in endemic regions including Pakistan and Iran. This consistency indicates that *H. anatolicum* is a competent vector for multiple major zoonoses, rather than a merely theoretical risk. While human case data derived from literature are subject to inherent reporting biases and likely underrepresent the disease burden, the observed spatial congruence analysis highlights the epidemiological significance of this vector in driving regional zoonotic transmission.

Finally, ENM projections reveal a potential for geographic range expansion of *H. anatolicum* into previously non-endemic regions. The model indicates (Fig. 6) that suitable habitats extend beyond the current known distribution, identifying a potential invasion risk for continents presently considered free of the vector, most notably western and southern Australia and the western and eastern United States. These findings suggest that the global habitat suitability for *H. anatolicum* may be broader than previously characterized. In our model, the most important variables affecting the distribution of *H. anatolicum* were mean temperature of the driest quarter (BIO9), land cover, and temperature seasonality (BIO4), which could explain over 80% of contributions to the model. The observed influence of temperature aligns with the known biological requirements of the species, specifically an optimal survival range of 15–30 °C [27], [28]. While topographic factors can influence microclimates and tick development [29], [30], [31], their relative contribution in our global-scale analysis was limited (Supplementary Data 1 Fig. S9; table S8). This suggests that the distribution of *H. anatolicum* is more constrained by anthropogenic landscape modifications and climatic conditions than by natural topographic variations. While the ENM characterizes environmental suitability, the vector's association with livestock suggests that climatic shifts and international trade may act as combined drivers facilitating long-distance dispersal [32], [33]. Consequently, transcontinental introduction of this vector could pose a substantial threat to global biosecurity, public health, and livestock economies.

This study has two main limitations. First, the primary reliance on literature published in English and Chinese may introduce linguistic and geographic bias, potentially underrepresenting localized findings from endemic regions such as Iran and Pakistan. Second, uneven surveillance across Central and Western Asia has created notable gaps in ecological and epidemiological data. Consequently, the reported absence of pathogens in certain regions may stem from inadequate surveillance or low diagnostic sensitivity—not true biological absence—and could thus lead to an underestimation of the actual public health risk. These limitations highlight the decentralized structure of the current global vector surveillance system. They necessitate coordinated cross-border surveillance and targeted capacity building in resource-limited regions to improve global risk assessments of *H. anatolicum* and its associated pathogens.

## 5 Conclusion

*H. anatolicum* serves as a vector for zoonotic and veterinary pathogens, affecting global public health and livestock productivity. This synthesis shows the species clusters in human-modified landscapes and depends on domestic livestock. It carries at least 66 microbial species. Ecological niche modeling reveals a risk of expansion into non-endemic regions, including Australia and the United States. These findings necessitate transboundary surveillance under the One Health framework. Early warning systems should combine climatic and trade data. Simultaneously, livestock-focused control measures should be implemented to reduce risks. Tightened monitoring is vital to prevent vector establishment in new areas.

## CRediT authorship contribution statement

**Shuo Zhou:** Writing – original draft, Visualization, Validation, Methodology, Investigation, Formal analysis, Data curation. **Qing Xu:** Formal analysis. **Jing Liu:** Formal analysis. **Jia-Yi Pan:** Investigation. **Xiao-Yang Wang:** Resources. **Hui-Jun Yu:** Resources. **Bing-Bing Gu:** Formal analysis. **Bao-Yu Wang:** Investigation. **Guo-Yao Zu:** Formal analysis. **Chen Shan:** Investigation. **Wu-Chun Cao:** Conceptualization. **Lin Zhao:** Writing – review & editing, Conceptualization.

## Funding

This study was supported by the 10.13039/501100001809Natural Science Foundation of China (82574162), the 10.13039/501100007129Natural Science Foundation of Shandong Province, China (ZR2024MH182), Young Talent of Lifting engineering for Science and Technology in Shandong, China (SDAST2024QTA079), the Youth Innovation Team of Shandong Provincial Colleges and Universities (2022KJ016), and 10.13039/100009108Cheeloo Young Scholar Program of Shandong University.

## Declaration of competing interest

We declare no competing interests.

## Data Availability

All data supporting the study's conclusions are available in the main text and supplementary materials. The global distribution dataset of *Hyalomma anatolicum* (including original coordinates, host and pathogen data, and environmental factor data) is in Supplementary Data 2–5. A complete list of original literature and datasets for meta-analysis and niche modeling is provided in Supplementary Data 1, with all data in a reusable format for future research and validation.

## Appendix A Supplementary data

Supplementary material 1: Supplementary Data 1: Methodological details and supplementary information. Image 1

Supplementary material 2: Supplementary Data 2: Global distribution of *Hyalomma anatolicum*. Image 2

Supplementary material 3: Supplementary Data 3: Agents of *Hyalomma anatolicum*. Image 3

Supplementary material 4: Supplementary Data 4: Hosts and Agents Image 4

Supplementary material 5: Supplementary Data 5: Bioclimatic variables (bio1-bio19). Note: Multimedia Components 5-28 are standardized variables used for Maxent modeling. Image 5

Supplementary material 6 Image 6

Supplementary material 7 Image 7

Supplementary material 8 Image 8

Supplementary material 9 Image 9

Supplementary material 10 Image 10

Supplementary material 11 Image 11

Supplementary material 12 Image 12

Supplementary material 13 Image 13

Supplementary material 14 Image 14

Supplementary material 15 Image 15

Supplementary material 16 Image 16

Supplementary material 17 Image 17

Supplementary material 18 Image 18

Supplementary material 19 Image 19

Supplementary material 20 Image 20

Supplementary material 21 Image 21

Supplementary material 22 Image 22

Supplementary material 23 Image 23

Supplementary material 24: Supplementary Data 5: Environmental variable: Aspect. Image 24

Supplementary material 25: Supplementary Data 5: Environmental variable: Elevation. Image 25

Supplementary material 26: Supplementary Data 5: Environmental variable: Land cover. Image 26

Supplementary material 27: Supplementary Data 5: Environmental variable: Slope. Image 27

Supplementary material 28: Supplementary Data 5: Environmental variable: Vegetation. Image 28
