# Supplementary material for: Global distribution, pathogen spectrum, and invasion potential of Hyalomma anatolicum at the human-livestock interface: A systematic review, meta-analysis, and ecological niche modeling
Source: One Health. 2026 Mar 13;22:101385. doi: 10.1016/j.onehlt.2026.101385 (PMC13050002; doi:10.1016/j.onehlt.2026.101385)
Supplement: Supplementary file 1 — Supplementary material 1: Supplementary Data 1: Methodological details and supplementary information. [file mmc1.pdf]

Supplementary data

Content

Figure S1: PRISMA flow diagram of study selection process 1

Table S1: The detailed search strategy for each database 2

Table S2: The inclusion and exclusion criteria of screening publications 2

Table S3: List of variables extracted from reviewed studies........ 3

Text S1: Literature and references on *Hyalomma anatolicum* 4

Figure S2: PRISMA flow diagram of study selection process 26

Table S4: Search terminology used for human infections........ 27

Text S2: References for Human Infection........ 28

Table S5: The PRISMA 2020 Checklist 114

Table S6: Environmental and meteorological variables downloaded for ecological modeling for *H. anatolicum* 118

Table S7: Known distribution locations of *H. anatolicum* for niche modeling........ 119

Text S3: Predictive modeling of the potential distribution of *H. anatolicum* 126

Text S4: Data Collection Characteristics 128

Figure S3: Geographic distribution of *H. anatolicum* in 32 countries 129

Figure S4: The distribution of *H. anatolicum* in Pakistan and China........ 130

Figure S5: Prevalence of *H. anatolicum*-associated microbes 131

Figure S6: Meta-analysis of the prevalence of each *H. anatolicum*-associated microbes..... 132

Figure S7: Meta-analysis of pathogens carried by hosts 166

Figure S8: The results of Maxent model for *H. anatolicum*........ 183

Figure S9: Jackknife plots of Maxent model for *H. anatolicum* 184

Table S8: Relative contributions of the environmental and meteorological variables to the Maxent model........ 185

Figure S10: Response curves of environmental variables to probability of *H. anatolicum* presence........ 186

Figure S1: PRISMA flow diagram of study selection process.

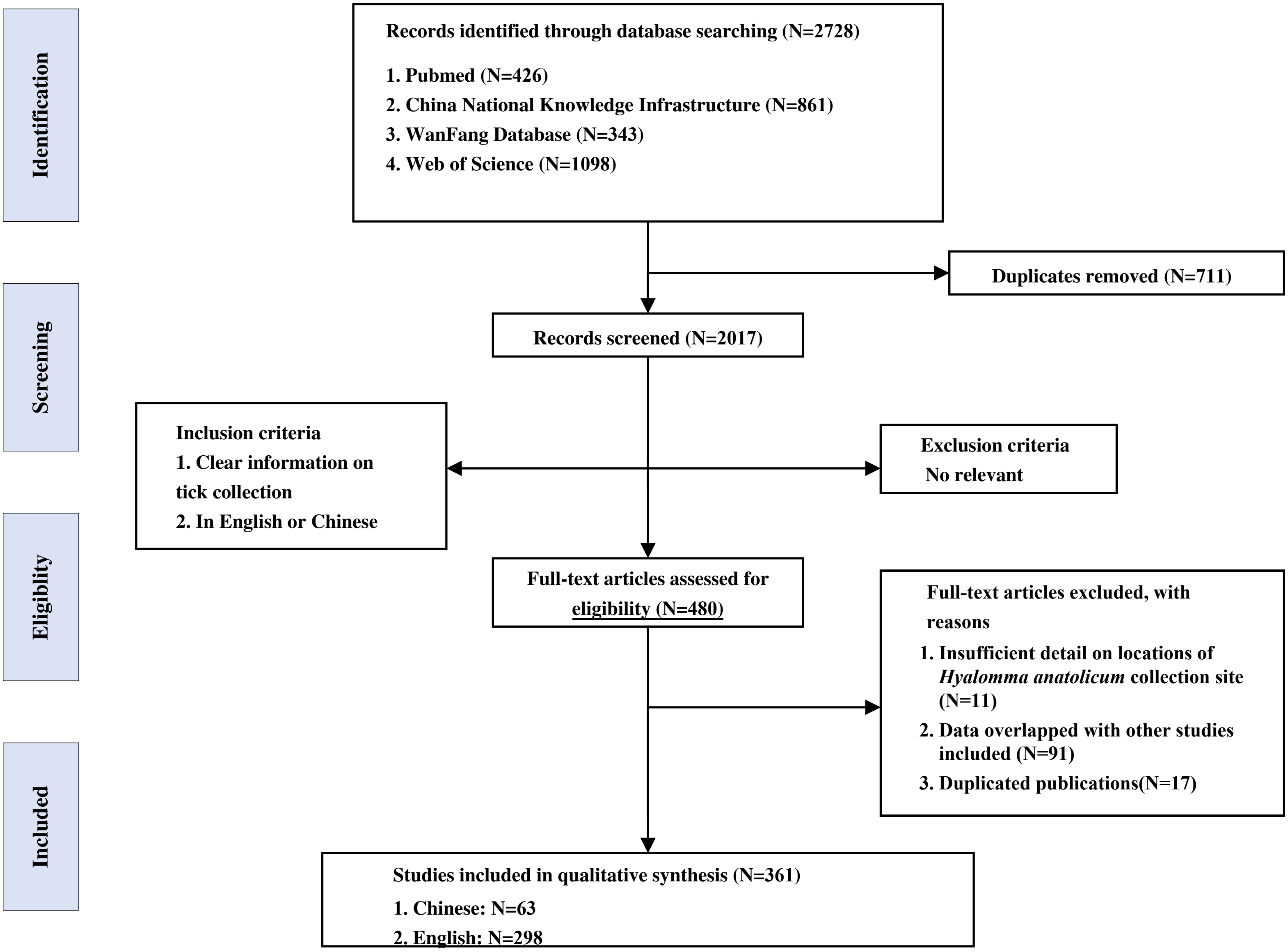

Table S1: The detailed search strategy for each database

The database searches were conducted up to April 1, 2024.

Database	Searches	Results
PubMed	"*Hyalomma anatolicum*"[All Fields] OR "*H. anatolicum*"[All Fields]	426
Web of Science	"*Hyalomma anatolicum*" OR "*H. anatolicum*"	1098
CNKI	检索范围：小亚璃眼蜱 (全文)	861
WanFang Database	检索范围：小亚璃眼蜱 (全文)	343

Table S2: The inclusion and exclusion criteria of screening publications

Criteria	Guidance	Outcome
**Title/Abstract screening**		
#1: Language restriction	Is the article written in Chinese or English?	If Yes, remain and evaluate #2.<br>If No, exclude.
#2: Target tick species	Does the Title/Abstract refer to *Hyalomma anatolicum* that were collected in the field?	If Yes, remain and evaluate #3.<br>If No, exclude.
#3: Core content	Does the Title/Abstract relate to *H. anatolicum*'s distribution, host animals, or carried pathogens?	If Yes, remain and evaluate #4.<br>If No, exclude.
#4: Primary data	Does the Title/Abstract indicate the article presents original data (not a review, commentary, or meta-analysis)?	If No, remain for full text review.<br>If Yes. exclude.
**Full text screening**		
#1: Full text	Does the full text of the article exist?	If Yes, remain and evaluate #2.<br>If No, exclude.
#2: Re-screening	Does the article meet all of the following:<br>1. Not clinical treatment research<br>2. Not only molecular mechanism/modeling research<br>3. Reports *H. anatolicum* in natural environments	If Yes, remain and evaluate #3.<br>If No, exclude.
#3: Species identification	Does the article report *H. anatolicum* identification with valid methods (morphological identification or molecular detection, e.g., 16S rRNA)?	If Yes, remain and evaluate #4.<br>If No, exclude.
#4: Geographical information	Does the article provide geographic location data (at least country/subnational level, or latitude/longitude coordinates)?	If Yes, remain for data extracting.<br>If No, exclude.
#5: Pathogen/host records	If the article involves pathogens/hosts:<br>Hosts and pathogens are identified to at least the genus level.	If Yes, remain for data extracting.<br>If No, exclude.

Table S3: List of variables extracted from reviewed studies

Variables	Explanation
Basic information of the study	
Reference ID	Unique identifier assigned to an article.
Article title	Article title that included in the review.
Authors	Authors of the included article.
Publication year	Publication year of the included article.
Study period	The start and end time for the period over which the sample collected.
Study site	Including the country, specific locality (province, city, or sampling point), and precise geographic coordinates (latitude and longitude).
Hyalomma anatolicum-associated information	
Tick life stage	Life stages of collected *Hyalomma anatolicum* (larva, nymph, adult male, adult female).
Tick sample size	Total number of *Hyalomma anatolicum* collected and examined in the study.
Habitat type	Describes the ecological environment of the sampling site, including both natural and human-modified habitats.
Host-associated information	
Host species	Taxonomic information of hosts at family, genus, and species levels.
Host sample size	Total number of hosts captured or examined for tick infestation.
Tick infestation information	Number of hosts found to be infested with Hyalomma anatolicum.
Host pathogen detection information	Number of hosts tested positive for tick-borne pathogens.
Detection method	The technology used to detect host-borne pathogens.
Pathogen-associated information	
Species of pathogen	Refers to the identified pathogen detected in *Hyalomma anatolicum*
Detection tick number	Total number of *Hyalomma anatolicum* subjected to pathogen detection.
Detection method	The technology used to detect tick-borne pathogens.
Number of pathogen - positive ticks	Number of Hyalomma anatolicum tested positive for the target pathogen.

Text S1: Literature and Reference on *Hyalomma anatolicum*

From the listed studies, we gather data on the primary author, the year of publication, the geographical sites of collection, the total count of *H. anatolicum* specimens examined, the quantity of samples that tested positive for particular pathogens, and the species of host animals.

Literature:

1. Kartashov MY, Kononova YV, Petrova ID, Tupota NL, Mikryukova TP, Ternovoi VA, et al. Detection of Ehrlichia spp. and Theileria spp. in *Hyalomma anatolicum* ticks collected in Tajikistan. Vavilovskii Zhurnal Genet Selektsii. 2020;24(1):55-9.
2. Ahmed BM, Hussein AME, Khider AOE. Some observations on ticks (Acari: Ixodidae) infesting sheep in River Nile Province of Northern Sudan. Onderstepoort Journal of Veterinary Research. 2005;72(3):239-43.
3. Kasi KK, von Arnim F, Schulz A, Rehman A, Chudhary A, Oneeb M, et al. Crimean-Congo haemorrhagic fever virus in ticks collected from livestock in Balochistan, Pakistan. Transbound Emerg Dis. 2020;67(4):1543-52.
4. Dhaka P, Malik SVS, Yadav JP, Ghosh S, Kumar M, Barbuddhe SB, et al. Molecular Investigation of the Status of Ticks on Infected Cattle for Coxiella burnetii in India. Acta Parasitol. 2020;65(3):779-82.
5. Abbasi F, Abbasi IHR, Nissa TF, Bhutto ZA, Arain MA, Soomro RN, et al. Epidemiological study of tick infestation in buffalo of various regions of district Khairpur, Pakistan. Vet World. 2017;10(6):688-94.
6. Singh NK, Jyoti, Vemu B, Nandi A, Singh H, Kumar R, et al. Laboratory assessment of acaricidal activity of Cymbopogon winterianus, Vitex negundo and Withania somnifera extracts against deltamethrin resistant *Hyalomma anatolicum*. Exp Appl Acarol. 2014;63(3):423-30.
7. Biglari P, Bakhshi H, Chinikar S, Belqeiszadeh H, Ghaffari M, Javaherizadeh S, et al. *Hyalomma anatolicum* as the Main Infesting Tick in an Important Livestock Rearing Region, Central Area of Iran. Iranian journal of public health. 2018;47(5):742.
8. Hosseini-Chegeni A, Hosseini R, Telmadarraiy Z, Abdigoudarzi M. The Iranian Hyalomma (Acari: Ixodidae) with molecular evidences to understand taxonomic status of species complexes. Persian Journal of Acarology. 2019;8(4):291-308.
9. Sharifinia N, Rafinejad J, Hanafi-Bojd AA, Chinikar S, Piazak N, Baniardalani M, et al. Hard ticks (Ixodidae) and Crimean-Congo hemorrhagic fever virus in south west of Iran. Acta medica Iranica. 2015;53(3):177-81.
10. Durrani AZ, Shakoori AR, Kamal N. Bionomics of Hyalomma ticks in three districts of Punjab, Pakistan. J Anim Plant Sci. 2008;18(1):17-23.
11. Guma E, Hussien M, Salih D, Salim B, Hassan S. Prevalence of ticks (Acari: Ixodidae) and Theileria annulata antibodies in White Nile State, Sudan. Journal of Advanced Veterinary and Animal Research. 2015;2(1).
12. Nangru A, Maharana BR, Vohra S, Kumar B, Ganguly A. Molecular Detection and Differentiation of Different Theileria Species in Naturally Infected Goats Using Nested PCR-RFLP: A First Report

from Northern India. Acta Parasitol. 2022;67(2):997-1006.
13. Petrova ID, Kononova YV, Chausov EV, Shestopalov AM, Tishkova FH. Genetic variants of the Crimean-Congo hemorrhagic fever virus circulating in endemic areas of Southern Tajikistan in 2009. Molecular Genetics Microbiology and Virology. 2013;28(3):119-26.
14. Qamar MF, Ayaz MM, Nazir MM. Isolation and identification of ectoparasites in single humped camels (Camelus dromedarius) of Cholistan area, Pakistan. Iraqi Journal of Veterinary Sciences. 2018;32(2):291-7.
15. Rahmani-Varmale M, Tavassoli M, Esmaeilnejad B. Molecular Detection and Differentiation of Theileria lestoquardi, T. ovis and T. annulata in Blood of Goats and Ticks in Kermanshah Province, Iran. Journal of arthropod-borne diseases. 2019;13(3):297.
16. Hamlili FZ, Laroche M, Diarra AZ, Lafri I, Gassen B, Boutefna B, et al. MALDI-TOF MS Identification of Dromedary Camel Ticks and Detection of Associated Microorganisms, Southern Algeria. Microorganisms. 2022;10(11).
17. Arjmand Yamchi J, Tavassoli M. Survey on infection rate, vectors and molecular identification of Theileria annulata in cattle from North West, Iran. J Parasit Dis. 2016;40(3):1071-6.
18. Jafarbekloo A, Ramzgouyan MR, Shirian S, Faghihi F, Bakhshi H, Naseri F, et al. Molecular Characterization and Phylogenetic Analysis of Anaplasma spp. and Ehrlichia spp. Isolated from Various Ticks in Southeastern and Northwestern Regions of Iran. Vector-Borne and Zoonotic Diseases. 2018;18(5):252-257.
19. Tila H, Khan M, Almutairi MM, Alouffi A, Ahmed H, Tanaka T, et al. First report on detection of Hepatozoon ayorgbor in Rhipicephalus haemaphysaloides and Hepatozoon colubri in Haemaphysalis sulcata and *Hyalomma anatolicum*: risks of spillover of Hepatozoon spp. from wildlife to domestic animals. Frontiers in Veterinary Science. 2023;10.
20. Sagar SV, Saini K, Kumar R, Shakya M, Saravanan BC, Ghosh S. Occurrence of unusually large *Hyalomma anatolicum* from Dewas district, Madhya Pradesh-case report. Journal of Veterinary Parasitology. 2018;32(2).
21. Hussain S, Saqib M, Ashfaq K, Sindhu ZUD. First Molecular Evidence of Coxiella burnetii in Ticks Collected from Dromedary Camels in Punjab, Pakistan. Pakistan Veterinary Journal. 2022;42(2):276-80.
22. Fard SR, Fathi S, Asl EN, Nazhad HA, Kazeroni SS. Hard ticks on one-humped camel (Camelus dromedarius) and their seasonal population dynamics in southeast, Iran. Trop Anim Health Prod. 2012;44(1):197-200.
23. Shehla S, Almutairi MM, Alouffi A, Tanaka T, Chang S-C, Chen C-C, et al. Molecular Survey of Rickettsia raoultii in Ticks Infesting Livestock from Pakistan with Notes on Pathogen Distribution in Palearctic and Oriental Regions. Veterinary Sciences. 2023;10(11).
24. Jadhao SG, Sanyal PK, Borkar SD, Chigure GM, Jadhav ND, Shirsikar PM, et al. Prevalence of ixodid ticks infesting in cattle of Chhattisgarh state, an east-central part of India. International Journal of Tropical Insect Science. 2020;40(4):951-4.
25. Zhao L, Lv J, Li F, Li K, He B, Zhang L, et al. Identification and Molecular Analysis of Ixodid Ticks (Acari: Ixodidae) Infesting Domestic Animals and Tick-Borne Pathogens at the Tarim Basin of Southern Xinjiang, China. Korean J Parasitol. 2020;58(1):37-46.
26. Prerna M, Singh NK, Jyoti, Singh H, Rath SS. Enzymatic detoxification mediated deltamethrin resistance in *Hyalomma anatolicum* (acari: ixodidae) populations of western Punjab. Exploratory

Animal & Medical Research. 2019;9(1).
27. Bursali A, Tekin S, Orhan M, Keskin A, Ozkan M. Ixodid ticks (Acari: Ixodidae) infesting humans in Tokat Province of Turkey: species diversity and seasonal activity. Journal of Vector Ecology. 2010;35(1):180-6.
28. Batool M, Nasir S, Rafique A, Yousaf I, Yousaf M. Prevalence of Tick Infestation in Farm Animals from Punjab, Pakistan. Pakistan Veterinary Journal. 2019;39(3):406-10.
29. Nabian S, Rahbari S. Occurrence of soft and hard ticks on ruminants in Zagros mountainous areas of Iran. Journal of Arthropod-Borne Diseases. 2008;2(1):16-20.
30. Tiwari A, Singh NK, Singh H, Jyoti, Bhat SA, Rath SS. Prevalence of Theileria annulata infection in *Hyalomma anatolicum anatolicum* collected from crossbred cattle of Ludhiana, Punjab. J Parasit Dis. 2015;39(1):57-61.
31. Nangru A, Maharana BR, Vohra S, Kumar B. Molecular identification of Theileria species in naturally infected sheep using nested PCR-RFLP. Parasitol Res. 2022;121(5):1487-97.
32. Lange Jv, Dessouky Age, Manor E, I.Merdan A, F.Azad A. Spotted fever rickettsiae in ticks from the northern Sinai Governate, Egypt. The American journal of tropical medicine and hygiene. 1992;46(5):546-51.
33. Samiurahman Amiri M, Yaghfoori S, Razmi G. Molecular Detection of Theileria annulata among Dairy Cattle and Vector Ticks in the Herat Area, Afghanistan. Arch Razi Inst. 2021;76(1):79-85.
34. Latha BR, Aiyasami SS, Pattabiraman G, Sivaraman T, Rajavelu G. Seasonal Activity of Ticks on Small Ruminants in TamilNadu State, India. Tropical Animal Health and Production. 2004;36:123-33.
35. Ashraf S, Parveen A, Muhammad Awais M, Gillani Q, Aktas M, Ozubek S, et al. A Report on Molecular Detection and Phylogenetic Evaluation of Anaplasma marginale in Ticks and Blood Samples Collected from Cattle in District Layyah in Punjab (Pakistan). Curr Microbiol. 2021;78(1):274-81.
36. Iqbal Z, Afshan K, Kayani AR, Ahmad H, Irfan M, Qayyum M. Epidemiology and Risk Mapping of hard ticks (Ixodidae) infecting Small Ruminants in Khyber Pakhtunkhwa Province, Pakistan. Journal of the Hellenic Veterinary Medical Society. 2023;74(1):5259-66.
37. Ni J, Lin H, Xu X, Ren Q, Aizezi M, Luo J, et al. Coxiella burnetii is widespread in ticks (Ixodidae) in the Xinjiang areas of China. BMC Vet Res. 2020;16(1):317.
38. Adegoke A, Kumar D, Bobo C, Rashid MI, Durrani AZ, Sajid MS, et al. Tick-Borne Pathogens Shape the Native Microbiome Within Tick Vectors. Microorganisms. 2020;8(9).
39. Yakhchali M, Bahramnejad K, Almasi O. Ticks (Acari: Ixodida: Ixodidae and Argasidae) abundance and associated risk factors for animals in the natural habitat of Sanandaj suburb, Iran. International Journal of Acarology. 2012;38(4):353-61.
40. Magzoub A, El Ghali A, Hussien MO, Juma Y, Mohammed SB. Prevalence of ticks (Acari: Ixodidae) and Theileria lestoquardi in sheep at El Huda and El Nuhud animals production research stations, Sudan. J Parasit Dis. 2021;45(1):146-52.
41. Ozubek S, Aktas M. Molecular and Parasitological Survey of Ovine Piroplasmosis, Including the First Report of Theileria annulata (Apicomplexa: Theileridae) in Sheep and Goats from Turkey. J Med Entomol. 2017;54(1):212-20.
42. Sajid MS, Iqbal Z, Khan MN, Muhammad G, Needham G, Khan MK. Prevalence, associated determinants, and in vivo chemotherapeutic control of hard ticks (Acari: Ixodidae) infesting domestic goats (Capra hircus) of lower Punjab, Pakistan. Parasitol Res. 2011;108(3):601-9.
43. Dehuri M, Panda M, Sahoo N, Mohanty B, Behera B. Nested PCR assay for detection of Theileria

annulata in *Hyalomma anatolicum* infesting cattle from coastal Odisha, India. Anim Biotechnol. 2022;33(6):1229-34.

44. Hosseini A, Dalimi A, Abdigoudarzi M. Morphometric Study on Male Specimens of Hyalomma anatolicum (Acari: Ixodidae) in West of Iran. Iranian Journal of Arthropod-Borne Diseases. 2011;5(2):23-31.

45. Razmi GR, Naghibi A, Aslani MR, Dastjerdi K, Hossieni H. An epidemiological study on Babesia infection in small ruminants in Mashhad suburb, Khorasan province, Iran. Small Ruminant Research. 2003;50(1-2):39-44.

46. Khan Z, Shehla S, Alouffi A, Kashif Obaid M, Zeb Khan A, Almutairi MM, et al. Molecular Survey and Genetic Characterization of Anaplasma marginale in Ticks Collected from Livestock Hosts in Pakistan. Animals (Basel). 2022;12(13).

47. Razmi G, Yaghfoori S. Molecular surveillance of Theileria ovis, Theileria lestoquardi and Theileria annulata infection in sheep and ixodid ticks in Iran. Onderstepoort J Vet Res. 2013;80(1):635.

48. Haghi FM, Razmi G, Fakhar M, Mohammadpoor RA. The hard ticks (Ixodidae) fauna of livestock in Sari suburb, Northern Iran. Comparative clinical pathology. 2013;22(1):5-8.

49. Haque M, Jyoti, Singh NK, Rath SS. Prevalence of Theileria annulata infection in *Hyalomma anatolicum anatolicum* in Punjab state, India. J Parasit Dis. 2010;34(1):48-51.

50. Paikade S, Chavan R. Studies on taxonomy of parasitic tick genus Hyalomma (Ixodida: Ixodidae) from Aurangabad district M.S. India. International Journal of Entomology Research. 2019;4(3):27-30.

51. Omer LT, Kadir MA, Seitzer U, Ahmed JS. A survey of ticks (Acari:Ixodidae) on cattle, sheep and goats in the Dohuk Governorate, Iraq. Parasitol Res. 2007;101 Suppl 2:S179-81.

52. Ghoneim NH, Abdel-Moein KA, Zaher HM, Abuowarda MM. Investigation of Ixodidae ticks infesting camels at slaughterhouse and its potential role in transmitting Coxiella burnetii in Egypt. Small Ruminant Research. 2020;191.

53. Adil MM, Parveen A, Asif M, Farooq M, Iqbal F. Prevalence and identification of tick species on large ruminants from district Rajanpur in Punjab Pakistan. International Journal of Acarology. 2021;47(7):633-7.

54. Khan V, Zala DB, Joshi KM. Occurrence of Hyalomma, (Acari: Ixodidae) Koch, 1844 on domestic animal in the Union Territory of Dadra & Nagar Haveli, Indian. J Parasit Dis. 2016;40(2):543-5.

55. Çapin GA, Emre Z, Canpolat S, Vatansever Y, Düzgün A. Detection of Coxiella burnetii from ticks by Polymerase Chain Reaction and Restriction Fragment Length Polymorphism. Ankara Üniversitesi Veteriner Fakültesi Dergisi. 2013;60(4):263-8.

56. Jamil M, Kashif M, Habibullah, Mubeen M, Jelani G, Ullah N, et al. Identification oftick species infesting livestock in Dera lsmail Khan Pakistan. Systematic and Applied Acarology. 2021;26(12):2247-52.

57. Sofizadeh A, Telmadarraiy Z, Rahnama A, Gorganli-Davaji A, Hosseini-Chegeni A. Hard Tick Species of Livestock and their Bioecology in Golestan Province, North of Iran. Journal of arthropod-borne diseases. 2014;8(1):108.

58. Moradi-asl E, Vatandoost H, Kumar S. Prevalence and Seasonal Activity of Ticks Infesting Livestock in North West Areas of Iran. Pakistan journal of medical & health sciences. 2020;14(1):455-8.

59. Gupta S, Gupta S, Kumar S. Emergence of fipronil resistance in cattle ticks Rhipicephalus

microplusand *Hyalomma anatolicum* collected from Haryana, India. International Journal of Tropical Insect Science. 2021;41(1):401-7.
60. Gaur RS, Sangwan AK, Sangwan N, Ghosh M, Kumar S. Comparative study of esterases in deltamethrin and diazinon resistant Rhipicephalus microplus and *Hyalomma anatolicum* ticks collected from the Trans-Gangetic plains of India. Exp Appl Acarol. 2017;73(1):115-27.
61. Shkap V, Pipano E, Rasulov I, Azimov D, Savitsky I, Fish L, et al. Proteolytic enzyme activity and attenuation of virulence in Theileria annulata schizont-infected cells. Veterinary Parasitology. 2003;115(3):247-255.
62. Singh NK, Rath SS. Epidemiology of ixodid ticks in cattle population of various agro-climatic zones of Punjab, India. Asian Pac J Trop Med. 2013;6(12):947-51.
63. Miranpuri G, Bindra O, Prasad V. Tick fauna of north-western India (Acarina : Metastigmata). International Journal of Acarology. 1975;1(1):31-54.
64. Sharma K, Sharma D, Moudgil AD, Kumar P, Thakur D. Spatial surveillance and risk factor assessment of ixodid ticks in cattle population of Himachal Shivalik hill zone of North-Western Himalayas. International Journal of Acarology. 2021;47(4):284-8.
65. Duzlu O, Yildirim A, Inci A, Gumussoy KS, Ciloglu A, Onder Z. Molecular Investigation of Francisella-Like Endosymbiont in Ticks and Francisella tularensis in Ixodid Ticks and Mosquitoes in Turkey. Vector Borne Zoonotic Dis. 2016;16(1):26-32.
66. Asmaa NM, ElBably MA, Shokier KA. Studies on prevalence, risk indicators and control options for tick infestation in ruminants. Beni-Suef University Journal of Basic and Applied Sciences. 2014;3(1):68-73.
67. Champour M, Chinikar S, Mohammadi G, Razmi G, Mostafavi E, Shah-Hosseini N, et al. Crimean-Congo Hemorrhagic Fever in the One-Humped Camel (Camelus dromedarius) in East and Northeast of Iran. Journal of Arthropod-Borne Diseases. 2016;10(2):168.
68. Dumanli N, Aktas M, Cetinkaya B, Cakmak A, Koroglu E, Saki CE, et al. Prevalence and distribution of tropical theileriosis in eastern Turkey. Vet Parasitol. 2005;127(1):9-15.
69. Singh NK, Rath SS. Epidemiology of ixodid ticks in buffaloes (Bubalus bubalis) of Punjab, India. Buffalo Bulletin. 2016;35(3):347-53.
70. Ramzan M, Naeem-Ullah U, Saba S, Iqbal N, Saeed S. Prevalence and identification of tick species (Ixodidae) on domestic animals in district Multan, Punjab Pakistan. International Journal of Acarology. 2020;46(2):83-7.
71. Snow KR, Arthur DR. Oviposition in *Hyalomma anatolicum anatolicum*(Koch, 1844)(Ixodoidea: Ixodidae). Parasitology. 1966;56(3):555-68.
72. Shehla S, Ullah F, Alouffi A, Almutairi MM, Khan Z, Tanaka T, et al. Association of SFG Rickettsia massiliae and Candidatus Rickettsia shennongii with Different Hard Ticks Infesting Livestock Hosts. Pathogens. 2023;12(9).
73. Razmi GR, Najarnejad v, Rashtibaf M. Determination the frequency of lxodid ticks on the sheepin Khorasan Razavi province, Iran. Archives of Razi Institute. 2011;66(2):129-32.
74. Rafiq N, Naseem M, Kakar A, Shirazi JH, Masood MI. A preliminary evaluation of tick cement-cone protein extract for a vaccine against Hyalomma infestation. Iranian Journal of Veterinary Research. 2022;23(3):255.
75. Sultan S, Zeb J, Ayaz S, Rehman SU, Khan S, Hussain M, et al. Epidemiologic profile of hard ticks and molecular characterization of Rhipicephalus microplus infesting cattle in central part of

Khyber Pakhtunkhwa, Pakistan. Parasitol Res. 2022;121(9):2481-93.
76. Sahibi H, Rhalem A, Berrag B, Goff WL. Bovine babesiosis. Seroprevalence and ticks associated with cattle from two different regions of Morocco. Ann N Y Acad Sci. 1998;849:213-8.
77. Moshaverinia A, Moghaddas E. Prevalence of tick infestation in dromedary camels (Camelus dromedarius) brought for slaughter in Mashhad abattoir, Iran. J Parasit Dis. 2015;39(3):452-5.
78. Davari B, Alam FN, Nasirian H, Nazari M, Abdigoudarzi M, Salehzadeh A. Seasonal distribution and faunistic of ticks in the Alashtar county (Lorestan Province), Iran. Pan Afr Med J. 2017;27:284.
79. Azizi S, Yakhchali M. Transitory lameness in sheep due to Hyalomma spp. infestation in Urmia, Iran. Small Ruminant Research. 2006;63(3):262-4.
80. Ali A, Obaid MK, Almutairi MM, Alouffi A, Numan M, Ullah S, et al. Molecular detection of Coxiella spp. in ticks (Ixodidae and Argasidae) infesting domestic and wild animals: with notes on the epidemiology of tick-borne Coxiella burnetii in Asia. Front Microbiol. 2023;14:1229950.
81. Deger MS, Bicek K, Oguz B. Infestation rate and distribution of hard ticks on cattle in the Eastern Anatolia Region of Turkey. Sci Parasitol. 2016;17(3-4):76-82.
82. Jafari A, Asadolahi S, Rasekh M, Saadati D, Faghihi F, Fazlalipour M, et al. Distribution and biodiversity components of hard ticks as potential vectors of Crimean-Congo haemorrhagic fever virus (CCHFV) in borderline of Iran-Afghanistan. International Journal of Acarology. 2021;47(6):510-9.
83. Kaur H, Chhillar S. Phylogenetic analysis of some hard ticks from India using mitochondrial 16s rDNA. Journal of Applied Biology & Biotechnology. 2016;4(3):24-32.
84. Sang C, Yang M, Xu B, Liu G, Yang Y, Kairullayev K, et al. Tick distribution and detection of Babesia and Theileria species in Eastern and Southern Kazakhstan. Ticks and Tick-Borne Diseases. 2021;12(6).
85. Sadeddine R, Diarra AZ, Laroche M, Mediannikov O, Righi S, Benakhla A, et al. Molecular identification of protozoal and bacterial organisms in domestic animals and their infesting ticks from north-eastern Algeria. Ticks Tick Borne Dis. 2020;11(2):101330.
86. Singh NK, Saini SPS, Singh H, Jyoti, Sharma SK, Rath SS. In vitro assessment of the acaricidal activity of Piper longum, Piper nigrum, and Zingiber officinale extracts against *Hyalomma anatolicum* ticks. Exp Appl Acarol. 2017;71(3):303-17.
87. Bochani N, Hashemi S. Investigation of ticks infecting sheep and seasonal changes of their population in Eyvan city. Ukrainian Journal of Ecology. 2018;8(1):432-6.
88. Vathsala M, Mohan P, Sacikumar, Ramessh S. Survey of tick species distribution in sheep and goats in Tamil Nadu, India. Small Ruminant Research. 2008;74(1-3):238-42.
89. Sumbria D, Singla LD, Sharma A, Bal MS. Detection of Theileria equi infection in ixodid ticks of equines using nested polymerase chain reaction from Punjab, India. The Indian Journal of Animal Sciences. 2018;88(10):1127-32.
90. Yadav PD, Whitmer SLM, Sarkale P, Fei Fan Ng T, Goldsmith CS, Nyayanit DA, et al. Characterization of Novel Reoviruses Wad Medani Virus (Orbivirus) and Kundal Virus (Coltivirus) Collected from *Hyalomma anatolicum* Ticks in India during Surveillance for Crimean Congo Hemorrhagic Fever. Journal of Virology. 2019;93(13).
91. Majid A, Almutairi MM, Alouffi A, Tanaka T, Yen T-Y, Tsai K-H, et al. First report of spotted fever group Rickettsia aeschlimannii in Hyalomma turanicum, Haemaphysalis bispinosa, and Haemaphysalis montgomeryi infesting domestic animals: updates on the epidemiology of tick-borne Rickettsia aeschlimannii. Frontiers in Microbiology. 2023;14.

92. Shuaib YA, Elhag AMW, Brima YA, Abdalla MA, Bakiet AO, Mohmed-Noor SE, et al. Ixodid tick species and two tick-borne pathogens in three areas in the Sudan. Parasitol Res. 2020;119(2):385-94.
93. Bekloo AJ, Bakhshi H, Soufizadeh A, Sedaghat MM, Bekloo RJ, Ramzgouyan MR, et al. Ticks circulate Anaplasma, Ehrlichia, Babesia and Theileria parasites in North of Iran. Vet Parasitol. 2017;248:21-4.
94. Noaman V. Identification of hard ticks collected from sheep naturally infected with Anaplasma ovis in Isfahan province, central Iran. Comparative clinical pathology. 2012;21(3):367-9.
95. Tavassoli M, Tabatabaei M, Mohammadi M, Esmaeilnejad B, Mohamadpour H. PCR-based Detection of Babesia spp. Infection in Collected Ticks from Cattle in West and North-West of Iran. Journal of arthropod-borne diseases. 2013;7(2):132–8.
96. Razmi GR, Eshrati H, Rashtibaf M. Prevalence of Theileria spp. infection in sheep in South Khorasan province, Iran. Vet Parasitol. 2006;140(3-4):239-43.
97. Ica A, Inci A, Vatansever Z, Karaer Z. Status of tick infestation of cattle in the Kayseri region of Turkey. Parasitol Res. 2007;101 Suppl 2:S167-9.
98. Salehi-Vaziri M, Vatandoost H, Sanei-Dehkordi A, Fazlalipour M, Pouriayevali MH, Jalali T, et al. Molecular assay on detection of Crimean Congo hemorrhagic fever (CCHF) virus in ixodid ticks collected from livestock in slaughterhouse from South of Iran. Journal of arthropod-borne diseases. 2020;14(3):286.
99. Alanazi AD, Alouffi AS, Alshahrani MY, Alyousif MS, Abdullah H, Allam AM, et al. A report on tick burden and molecular detection of tick-borne pathogens in cattle blood samples collected from four regions in Saudi Arabia. Ticks Tick Borne Dis. 2021;12(3):101652.
100. Vatandoost H, Asl EM, Telmadarreiy Z, Mohebali M, Asl HM, Abai MR, et al. Field efficacy of flumethrin pour-on against livestock ticks in Iran. International Journal of Acarology. 2012;38(6):457-64.
101. Sajid MS, Iqbal A, Rizwan HM, Kausar A, Tahir UB, Younus M, et al. Guardians of the Herd: Molecular Surveillance of Tick Vectors Uncovers Theileriosis Perils in Large Ruminants. Microorganisms. 2023;11(11).
102. Orkun O, Karaer Z, Cakmak A, Nalbantoglu S. Crimean-Congo hemorrhagic fever virus in ticks in Turkey: A broad range tick surveillance study. Infection Genetics and Evolution. 2017;52:59-66.
103. Derradj L, Kohil K. Incidence, species and attachment sites of Ixodidae ticks in cattle, sheep and goats in Algeria. AgroLife Scientific Journal. 2022;11(1).
104. Shemshad K, Rafinejad J, Kamali K, Piazak N, Sedaghat MM, Shemshad M, et al. Species diversity and geographic distribution of hard ticks (Acari: Ixodoidea: Ixodidae) infesting domestic ruminants, in Qazvin Province, Iran. Parasitol Res. 2012;110(1):373-80.
105. Dilcher M, Faye O, Faye O, Weber F, Koch A, Sadegh C, et al. Zahedan rhabdovirus, a novel virus detected in ticks from Iran. Virol J. 2015;12:183.
106. Hajipour N, Tavassoli M, Gorgani-Firouzjaee T, Naem S, Pourreza B, Bahramnejad K, et al. Hedgehogs (Erinaceus europaeus) as a Source of Ectoparasites in Urban-suburban Areas of Northwest of Iran. Journal of Arthropod-Borne Diseases. 2015;9(1):98.
107. Orkun Ö. Comprehensive screening of tick-borne microorganisms indicates that a great variety of pathogens are circulating between hard ticks (Ixodoidea: Ixodidae) and domestic ruminants in natural foci of Anatolia. Ticks Tick Borne Dis. 2022;13(6):102027.

108. Zeb J, Szekeres S, Takács N, Kontschán J, Shams S, Ayaz S, et al. Genetic diversity, piroplasms and trypanosomes in Rhipicephalus microplus and *Hyalomma anatolicum* collected from cattle in northern Pakistan. Exp Appl Acarol. 2019;79(2):233-43.
109. A IM, A R, T P. Antibodies against rickettsia in humans and potential vector ticks from Dhofar, Oman. Journal for scientific research Medical sciences. 2000;2(1):7.
110. Khan M, Almutairi MM, Alouffi A, Tanaka T, Chang S-C, Chen C-C, et al. Molecular evidence of Borrelia theileri and closely related Borrelia spp. in hard ticks infesting domestic animals. Frontiers in Veterinary Science. 2023;10.
111. Kandi S, Chennuru S, Chitichoti J, Metta M, Krovvidi S. Morphological and molecular characterization of ticks infesting cattle and buffaloes in different agro-climatic zones in Andhra Pradesh, India, and factors associated with high tick prevalence. International Journal of Acarology. 2022;48(3):192-200.
112. Ali S, Hasan M, Ahmad AS, Ashraf K, Khan JA, Rashid MI. Molecular prevalence of Anaplasma marginale in ruminants and Rhipicephalus ticks in northern Pakistan. Trop Biomed. 2023;40(1):7-13.
113. Muhammad K, Idrees A, Iqbal J, Arif U, Safdar Baloch M, Faisal Shahzad M, et al. Prevalence of cattle ticks in various agro-ecological zones of Khyber Pakhtunkhwa, and evaluation of botanical extracts against Hyalomma detritum. Journal of King Saud University - Science. 2023;35(6).
114. Hussain N, Shabbir RMK, Ahmed H, Afzal MS, Ullah S, Ali A, et al. Prevalence of different tick species on livestock and associated equines and canine from different agro-ecological zones of Pakistan. Front Vet Sci. 2022;9:1089999.
115. Al-Hosary A, Ahmed L, Ahmed J, Nijhof A, Clausen PH. Epidemiological study on tropical theileriosis (Theileria annulata infection) in the Egyptian Oases with special reference to the molecular characterization of Theileria spp. Ticks Tick Borne Dis. 2018;9(6):1489-93.
116. Yaser SA, Sadegh C, Zakkyeh T, Hassan V, Maryam M, Ali OM, et al. Crimean--Congo hemorrhagic fever: a molecular survey on hard ticks (Ixodidae) in Yazd province, Iran. Asian Pac J Trop Med. 2011;4(1):61-3.
117. Khan SS, Ahmed H, Afzal MS, Khan MR, Birtles RJ, Oliver JD. Epidemiology, Distribution and Identification of Ticks on Livestock in Pakistan. Int J Environ Res Public Health. 2022;19(5).
118. Sanghai AA, Drago A, Zala DB, Khan V, Das VK. Population ecology of *Hyalomma anatolicum anatolicum* (Acari: Ixodidae) in cattle of UT of Dadra & Nagar Haveli, India. Indian Vet J. 2018;95(3):21-3.
119. Gholmohammadi S, Malekifard F, Yakhchali M. Study on species distribution and seasonal dynamics of equine tick infestation in Ardabil province, northwest of Iran. Veterinary Parasitology: Regional Studies and Reports. 2024;48.
120. O G, M Y, S S. PCR-RELP for detecting of Theileria annulata infection in cattle and Hyalomma species in Kermanshah Province, Iran. Archives of Razi Institute. 2015;70(1):7-12.
121. Parveen A, Ashraf S, Aktas M, Ozubek S, Iqbal F. Molecular epidemiology of Theileria annulata infection of cattle in Layyah District, Pakistan. Exp Appl Acarol. 2021;83(3):461-73.
122. Loui Monfared A, Mahmoodi M, Fattahi R. Prevalence of ixodid ticks on cattle, sheep and goats in Ilam County, Ilam Province, Iran. J Parasit Dis. 2015;39(1):37-40.
123. Aneela A, Almutairi MM, Alouffi A, Ahmed H, Tanaka T, da Silva Vaz I, et al. Molecular Detection of Rickettsia hoogstraalii in *Hyalomma anatolicum* and Haemaphysalis sulcata: Updated Knowledge on the Epidemiology of Tick-Borne Rickettsia hoogstraalii. Veterinary Sciences.

2023;10(10).
124. Naseem A, Khan MA, Ali SZ. Prevalence and risk factors associated with tick infestation of buffaloes in the eastern part of Uttar Pradesh, India. International Journal of Acarology. 2020;46(5):344-50.
125. Hassan MA, Raoofi A, Hosseini A, Mehrara MR, Amininajafi F. Prevalence of ixodid ticks on cattle and sheep northeast of Iran. J Parasit Dis. 2016;40(3):772-3.
126. Tekin S, Bursali A, Mutluay N, Keskin A, Dundar E. Crimean-Congo hemorrhagic fever virus in various ixodid tick species from a highly endemic area. Vet Parasitol. 2012;186(3-4):546-52.
127. Shamsi L, Samaeinasab S, Haghighatkhah A. Identification of ectoparasites of camels in Sabzevar City, Iran. Ann Parasitol. 2020;66(4):555-9.
128. Choubdar N, Karimian F, Koosha M, Nejati J, Oshaghi MA. Hyalomma spp. ticks and associated Anaplasma spp. and Ehrlichia spp. on the Iran-Pakistan border. Parasit Vectors. 2021;14(1):469.
129. Soundararajan C, Nagarajan K, Muthukrishnan S, Arul Prakash M. Tick infestation on sheep, goat, horse and wild hare in Tamil Nadu. J Parasit Dis. 2018;42(1):127-9.
130. Choubdar N, Karimian F, Koosha M, Oshaghi MA. An integrated overview of the bacterial flora composition of *Hyalomma anatolicum*, the main vector of CCHF. PLoS Negl Trop Dis. 2021;15(6):e0009480.
131. Yadav PD, Cherian SS, Zawar D, Kokate P, Gunjikar R, Jadhav S, et al. Genetic characterization and molecular clock analyses of the Crimean-Congo hemorrhagic fever virus from human and ticks in India, 2010-2011. Infect Genet Evol. 2013;14:223-31.
132. Latif AA. Tick (Acari= Ixodoidea: Ixodidae) immatures feeding on cattle, camels and sheep in the Sudan, with notes on behaviour of *Hyalomma anatolicum anatolicum* larvae fed on different hosts. Insect Science and Its Application. 1985;6(1):59-61.
133. Soltan-Alinejad P, Ramezani Z, Edalat H, Telmadarraiy Z, Dabiri F, Vatandoost H, et al. Molecular characterization of Ribosomal DNA (ITS2) of hard ticks in Iran: understanding the conspecificity of Dermacentor marginatus and D. niveus. BMC Res Notes. 2020;13(1):478.
134. Sutton AJ, Karagenc T, Bakirci S, Sarali H, Pekel G, Medley GF. Modelling the transmission dynamics of Theileria annulata: model structure and validation for the Turkish context. Parasitology. 2012;139(4):441-53.
135. Li Y, Guan G, Liu A, Peng Y, Luo J, Yin H. Experimental transmission of Theileria ovis by *Hyalomma anatolicum anatolicum*. Parasitol Res. 2010;106(4):991-4.
136. Nasiri A, Telmadarraiy Z, Vatandoost H, Chinikar S, Moradi M, Oshaghi M, et al. Tick infestation rate of sheep and their distribution in Abdanan County, Ilam Province, Iran, 2007–2008. Iranian journal of arthropod-borne diseases. 2010;4(2).
137. Sultankulova KT, Shynybekova GO, Issabek AU, Mukhami NN, Melisbek AM, Chervyakova OV, et al. The Prevalence of Pathogens among Ticks Collected from Livestock in Kazakhstan. Pathogens. 2022;11(10).
138. Salih DA, Sharieff OE, Lazarus AG, Hassan SM, Hussein AME. Natural infection rates and transmission of Theileria annulata by *Hyalomma anatolicum anatolicum* ticks in the Sudan. Onderstepoort Journal of Veterinary Research. 2005;72(4):303-7.
139. Liu Y, Mi X, Wang B, Wu J, He W, Luo T, et al. A case of gynandromorphism in *Hyalomma anatolicum* (Ixodida: Ixodidae). Exp Appl Acarol. 2023;91(1):133-7.
140. Karim S, Budachetri K, Mukherjee N, Williams J, Kausar A, Hassan MJ, et al. A study of ticks

and tick-borne livestock pathogens in Pakistan. PLoS Negl Trop Dis. 2017;11(6):e0005681.
141. Velusamy R, Ponnudurai G, Alagesan A, Rani N, Kolte SW, Rubinibala B. Epidemiology and molecular characterization of Theileria annulata in ticks collected from cattle in the central part of Tamil Nadu, India. Parasitology Research. 2023;122(12):3077-86.
142. Köseoğlu AE, Can H, Güvendi M, Erkunt Alak S, Kandemir Ç, Taşkın T, et al. Molecular investigation of bacterial and protozoal pathogens in ticks collected from different hosts in Turkey. Parasit Vectors. 2021;14(1):270.
143. Nabian S, Rahbari S, Changizi A, Shayan P. The distribution of Hyalomma spp. ticks from domestic ruminants in Iran. Med Vet Entomol. 2009;23(3):281-3.
144. Loftis AD, Reeves WK, Szumlas DE, Abbassy MM, Helmy IM, Moriarity JR, et al. Rickettsial agents in Egyptian ticks collected from domestic animals. Exp Appl Acarol. 2006;40(1):67-81.
145. Habeeba S, Bensalah OK, Ibrahim A, Al Muhairi S, Al Hammadi Z, Commey A, et al. Acaricide resistance in ticks from livestock farms in Abu Dhabi, United Arab Emirates. Acarologia. 2024;64(1):138-45.
146. Haque M, Jyoti, Singh NK, Rath SS, Ghosh S. Epidemiology and seasonal dynamics of Ixodid ticks of dairy animals of Punjab state, India. Indian Journal of Animal Sciences. 2011;81(7):661.
147. Rafiq N, Kakar A, Ghani A, Iqbal A, Achakzai WM, Sadozai S, et al. Ixodid Ticks (Arachnida: Acari) Prevalence Associated with Risk Factors in the Bovine Host in District Quetta, Balochistan. Pakistan Journal of Zoology. 2017;49(6):2113-21.
148. Leriche PD, Altan Y, Campbell JB, Efstathi Gc. Ticks (Ixodoidea) of domestic animals in Cyprus. Bulletin of Entomological Research. 1974;64(1):53-63.
149. Zeb J, Shams S, Ayaz S, Din IU, Khan A, Adil N, et al. Epidemiology of ticks and molecular characterization ofRhipicephalus microplusin cattle population in North-Western Pakistan. International Journal of Acarology. 2020;46(5):335-43.
150. Fathi A, Nabavi R, Noaman V, Sarani A, Saadati D, Ben Said M, et al. Molecular identification, risk factor assessment, and phylogenetic analysis of tick-borne pathogens in symptomatic and asymptomatic cattle from South-Eastern Iran. Experimental and Applied Acarology. 2024;92(3):479-506.
151. El-Azazy OME, Scrimgeour EM. Crimean-Congo haemorrhagic fever virus infection in the Western Province of Saudi Arabia. Transactions of the Royal Society of Tropical Medicine and Hygiene. 1997;91(3):275-8.
152. Williams RJ, Al-Busaidy S, Mehta FR, Maupin GO, Wagoner KD, Al-Awaidy S, et al. Crimean-congo haemorrhagic fever: a seroepidemiological and tick survey in the Sultanate of Oman. Trop Med Int Health. 2000;5(2):99-106.
153. Dimanopoulou AP, Starras AG, Diakou A, Lefkaditis M, Giadinis ND. Prevalence of tick species in sheep and goat flocks in areas of southern Greece. Journal of the Hellenic Veterinary Medical Society. 2018;68(2).
154. Asadollahi Z, Jalali MHR, Alborzi A, Hamidinejat H. Detection of Theileria-like organisms in Hyalomma ticks (Acarina: Ixodidae) in Khuzestan, Iran. Scientia Parasitologica. 2018;19(1-2):34-9.
155. Inci A, Ica A, Yildirim, Vatansever Z, Çakmak A, Albasan H, et al. Epidemiology of Tropical Theileriosis in the Cappadocia Region. Turkish Journal of Veterinary & Animal Sciences. 2008;32(1):57-64.
156. Polat E, Altinkum SM, Bagdatli Y, Baykara O. The tick fauna in Istanbul, Turkey, from 2013 to

2017 and identification of their pathogens by multiplex PCR: an epidemiological study. Exp Appl Acarol. 2021;84(4):825-34.
157. Shemshad M, Shemshad K, Sedaghat MM, Shokri M, Barmaki A, Baniardalani M, et al. First survey of hard ticks (Acari: Ixodidae) on cattle, sheep and goats in Boeen Zahra and Takistan counties, Iran. Asian Pac J Trop Biomed. 2012;2(6):489-92.
158. Ghazanabad AE, Esfandiari N, Najafi M, Mehrabi S, Sarani S, Khademi P, et al. Molecular detection of Coxiella burnetii in tick and blood samples from small ruminants in northwest of Iran. Experimental and Applied Acarology. 2024;92(3):529-46.
159. Williams RE, Hoogstraal H, Casals J, Kaiser MN, Moussa MI. Isolation of Wanowrie, Thogoto, and Dhori Viruses from Hyalomma Ticks Infesting Camels in Egypt1. Journal of Medical Entomology. 1973;10(2):143-6.
160. Ghafar A, Cabezas-Cruz A, Galon C, Obregon D, Gasser RB, Moutailler S, et al. Bovine Ticks Harbour a Diverse Array of Microorganisms in Pakistan. Parasites & Vectors. 2020;13(1):1.
161. Sarani M, Telmadarraiy Z, Moghaddam AS, Azam K, Sedaghat MM. Distribution of ticks (Acari: Ixodidae) infesting domestic ruminants in mountainous areas of Golestan province, Iran. Asian Pac J Trop Biomed. 2014;4(Suppl 1):S246-51.
162. Mohammadian M, Chinikar S, Telmadarraiy Z, Vatandoost H, Oshaghi MA, Hanafi-Bojd AA, et al. Molecular assay on Crimean Congo hemorrhagic fever virus in ticks (Ixodidae) collected from Kermanshah Province, western Iran. Journal of Arthropod-Borne Diseases. 2016;10(3):381.
163. Sedaghat MM, Sarani M, Chinikar S, Telmadarraiy Z, Moghaddam AS, Azam K, et al. Vector prevalence and detection of Crimean-Congo haemorrhagic fever virus in Golestan Province, Iran. Journal of Vector Borne Diseases. 2017;54(4):353-7.
164. Salih DA, Hassan SM, Hussein AME, Jongejan F. Preliminary survey of ticks (Acari: Ixodidae) on cattle in northern Sudan. Onderstepoort Journal of Veterinary Research. 2004;71(4):319-26.
165. Sangwan AK, CHhhabra MB, Samantaray S. Theileria infectivity ofHyalomma ticks in Haryana, India. Tropical animal health and production. 1986;18:149-54.
166. Bakirci S, Sarali H, Aydin L, Eren H, Karagenc T. Distribution and seasonal activity of tick species on cattle in the West Aegean region of Turkey. Experimental and Applied Acarology. 2012;56(2):165-78.
167. Rajabi S, Esmaeilnejad B, Tavassoli M. A molecular study on Babesia spp. in cattle and ticks in West-Azerbaijan province, Iran. Veterinary Research Forum. 2017;8(4):299 - 306.
168. Demir S, Erkunt Alak S, Köseoğlu AE, Ün C, Nalçacı M, Can H. Molecular investigation of Rickettsia spp. and Francisella tularensis in ticks from three provinces of Turkey. Exp Appl Acarol. 2020;81(2):239-53.
169. Jyoti, Singh NK, Singh H, Rath SS. Modified larval packet test based detection of amitraz resistance in *Hyalomma anatolicum* Koch (Acari: Ixodidae) from Punjab districts of India. International Journal of Acarology. 2019;45(6-7):391-4.
170. Choubdar N, Oshaghi MA, Rafinejad J, Pourmand MR, Maleki-Ravasan N, Salehi-Vaziri M, et al. Effect of Meteorological Factors on Hyalomma Species Composition and Their Host Preference, Seasonal Prevalence and Infection Status to Crimean-Congo Haemorrhagic Fever in Iran. Journal of Arthropod-Borne Diseases. 2019;13(3):268.
171. Yin H, Luo J, Guan G, Lu B, Ma M, Zhang Q, et al. Experiments on transmission of an unidentified Theileria sp. to small ruminants with Haemaphysalis qinghaiensis and *Hyalomma*

anatolicum anatolicum. Veterinary Parasitology. 2002;108(1):21-30.
172. Shyma KP, Kumar S, Sharma AK, Ray DD, Ghosh S. Acaricide resistance status in Indian isolates of *Hyalomma anatolicum*. Exp Appl Acarol. 2012;58(4):471-81.
173. Razmi GR, Naghibi A, Aslani MR, Fathivand M, Dastjerdi K. An epidemiological study on ovine babesiosis in the Mashhad suburb area, province of Khorasan, Iran. Veterinary Parasitology. 2002;108(2):109-15.
174. Patra G, Ghosh S, Priyanka, Efimova MA, Sahara A, Al-Awsi GRL, et al. Molecular detection of Coxiella burnetii and Borrelia burgdorferi in ticks infesting goats in North-Eastern states of India. International Journal of Acarology. 2020;46(6):431-8.
175. Omer SA, Alsuwaid DF, Mohammed OB. Molecular characterization of ticks and tick-borne piroplasms from cattle and camel in Hofuf, eastern Saudi Arabia. Saudi J Biol Sci. 2021;28(3):2023-8.
176. Dehaghi MM, Fathi S, Asl EN, Nezhad HA. Prevalence of ixodid ticks on cattle and sheep southeast of Iran. Trop Anim Health Prod. 2011;43(2):459-61.
177. Champour M, Chinikar S, Mohammadi G, Razmi G, Shah-Hosseini N, Khakifirouz S, et al. Molecular epidemiology of Crimean-Congo hemorrhagic fever virus detected from ticks of one humped camels (Camelus dromedarius) population in northeastern Iran. J Parasit Dis. 2016;40(1):110-5.
178. Ahmed S, Numan M, Manzoor AW, Ali FA. Investigations into Ixodidae ticks in cattle in Lahore, Pakistan. Vet Ital. 2012;48(2):185-91.
179. Al-Khalifa MS, Al-Asgah NA, Diab FM. Ticks (Acari: Ixodidae) Infesting Common Domestic Animals in Al-Qasim Province, Saudi Arabia. Journal of Medical Entomology. 1984;21(1):114-5.
180. Ullah N, Jamil M, Ramzan M, Arshad A, ul Haq MZ. Identification and new records of tick species on livestock from district Dera Ismail Khan, Pakistan. Persian Journal of Acarology. 2022;11(1):159-62.
181. Ghafar A, Khan A, Cabezas-Cruz A, Gauci CG, Niaz S, Ayaz S, et al. An Assessment of the Molecular Diversity of Ticks and Tick-Borne Microorganisms of Small Ruminants in Pakistan. Microorganisms. 2020;8(9).
182. Ozdarendeli A, Aydin K, Tonbak S, Aktas M, Altay K, Koksal I, et al. Genetic analysis of the M RNA segment of Crimean-Congo hemorrhagic fever virus strains in Turkey. Arch Virol. 2008;153(1):37-44.
183. Rooman M, Assad Y, Tabassum S, Sultan S, Ayaz S, Khan MF, et al. A cross-sectional survey of hard ticks and molecular characterization of Rhipicephalus microplus parasitizing domestic animals of Khyber Pakhtunkhwa, Pakistan. PLoS One. 2021;16(8):e0255138.
184. Pegram RG. Ticks (Acarina, Ixodoidea) of the northern regions of the Somali Democratic Republic. Bulletin of Entomological Research. 1976;66(2):345-63.
185. Bhagwan J, Kumar A, Kumar R, Goyal L, Goel P, Kumar S. Molecular evidence of Theileria equi infection in *Hyalomma anatolicum* ticks infested on sero-positive Indian horses. Acta Parasitol. 2015;60(2):322-9.
186. Ali S, Ijaz M, Ghaffar A, Oneeb M, Masud A, Durrani AZ, et al. Species Distribution and Seasonal Dynamics of Equine Tick Infestation in Two Subtropical Climate Niches in Punjab, Pakistan. Pakistan Veterinary Journal. 2020;40(1):25-30.
187. Arzamani K, Saghafipour A, Hashemi SA, Vatandoost H, Alavinia M, Raeghi S, et al. Biodiversity Indices and Medically Importance of Ticks in North Khorasan Province, Northeast of Iran.

Journal of Arthropod-Borne Diseases. 2021;18(7).
188. Rehman A, Nijhof AM, Sauter-Louis C, Schauer B, Staubach C, Conraths FJ. Distribution of ticks infesting ruminants and risk factors associated with high tick prevalence in livestock farms in the semi-arid and arid agro-ecological zones of Pakistan. Parasit Vectors. 2017;10(1):190.
189. Nader J, Król N, Pfeffer M, Ohlendorf V, Marklewitz M, Drosten C, et al. The diversity of tick-borne bacteria and parasites in ticks collected from the Strandja Nature Park in south-eastern Bulgaria. Parasites & Vectors. 2018;11(1).
190. Mohammadi SM, Esmaeilnejad B, Jalilzadeh-Amin G. Molecular detection, infection rate and vectors of Theileria lestoquardi in goats from West Azerbaijan province, Iran. veterinary Research Forum. 2017;8(2):139.
191. Haneef M. Incidence of ectoparasite in chiltan wild goat (Artiodactyla: Caprinae) native of Hazarganji chiltan national park (HCNP), Balochistan, Pakistan. Pure and Applied Biology. 2018;7(4).
192. Yu P, Liu Z, Niu Q, Yang J, Abdallah MO, Chen Z, et al. Molecular evidence of tick-borne pathogens in *Hyalomma anatolicum* ticks infesting cattle in Xinjiang Uygur Autonomous Region, Northwestern China. Exp Appl Acarol. 2017;73(2):269-81.
193. Singh NK, Gelot IS, Jyoti, Bhat SA, Singh H, Singh V. Detection of acaricidal resistance in *Hyalomma anatolicum anatolicum* from Banaskantha district, Gujarat. J Parasit Dis. 2015;39(3):563-6.
194. Ramezani Z, Chavshin AR, Telmadarraiy Z, Edalat H, Dabiri F, Vatandoost H, et al. Ticks (Acari: Ixodidae) of livestock and their seasonal activities, northwest of Iran. Asian Pacific Journal of Tropical Disease. 2014;4:S754-S7.
195. Shanan SMH, Abbas SF, Mohammad MK. Ixodid Ticks Diversity and Seasonal Dynamic on Cattle in North, Middle and South of Iraq. Systematic and Applied Acarology. 2017;22(10):1651-8.
196. Aktas M, Altay K, Dumanli N. A molecular survey of bovine Theileria parasites among apparently healthy cattle and with a note on the distribution of ticks in eastern Turkey. Vet Parasitol. 2006;138(3-4):179-85.
197. Hamzah KJ, Hasso SA. Molecular prevalence of Anaplasma phagocytophilum in sheep from Iraq. Open Vet J. 2019;9(3):238-45.
198. Gupta S, Gupta S, Kumar S. Cypermethrin resistance in *Hyalomma anatolicum* and Rhipicephalus microplusticks of arid and semi-arid zone of Haryana, a northern state of India. International Journal of Tropical Insect Science. 2021;41(1):703-9.
199. Alam S, Khan M, Alouffi A, Almutairi MM, Ullah S, Numan M, et al. Spatio-Temporal Patterns of Ticks and Molecular Survey of Anaplasma marginale, with Notes on Their Phylogeny. Microorganisms. 2022;10(8).
200. Lak SS, Vatandoost H, Telmadarraiy Z, Mahdi RE, Kia E. Seasonal Activity of Ticks and their Importance in Tick-Borne Infectious Diseases in West Azerbaijan, Iran. Journal of Arthropod-Borne Diseases. 2008;2(2):28-34.
201. Taha KM, Salih DA, Ahmed BM, Enan KA, Ali AM, Elhussein AM. First confirmed report of outbreak of malignant ovine theileriosis among goats in Sudan. Parasitol Res. 2011;109(6):1525-7.
202. Mohammed MS, Hassan SM. Distribution and population dynamics of ticks (Acari: Ixodidae) infesting sheep in Sennar State, Sudan. Onderstepoort Journal of Veterinary Research. 2007;74(4):301-6.
203. Yasmeen N, Kashif M, Arif T, Khan A, Ali M, Khan MA, et al. Distribution, prevalence and diversity of ticks (Acari: Ixodidae) infesting one-humped camels from Southwest Punjab, Pakistan. Vet

Parasitol Reg Stud Reports. 2023;37:100825.
204. Robson J, Robb JM, Hawa NJ. Ticks (Ixodoidea) of domestic animals in Iraq: Part 5. Infestations in the liwas of diwaniya and nasiriya (spring), karbala (winter), and hilla (autumn and winter). Journal of Medical Entomology. 1969;6(2):120-4.
205. Razmi GR, Hosseini M, Aslani MR. Identification of tick vectors of ovine theileriosis in an endemic region of Iran. Vet Parasitol. 2003;116(1):1-6.
206. Ul-Hasan M, Abubakar M, Muhammad G, Khan MN, Hussain M. Prevalence of tick infestation (Rhipicephalus sanguineus and *Hyalomma anatolicum anatolicum*) in dogs in Punjab, Pakistan. Veterinaria italiana. 2012;48(1):95-8.
207. Kandi S, Chennuru S, Chitichoti J, Metta M, Krovvidi S. Morphological and molecular characterization of ticks infesting cattle and buffaloes in different agro-climatic zones in Andhra Pradesh, India, and factors associated with high tick prevalence. International Journal of Acarology. 2022;48(3):192-200.
208. Dabaja MF, Tempesta M, Bayan A, Vesco G, Vesco G, Greco G, et al. Diversity and distribution of ticks from domestic ruminants in Lebanon. Vet Ital. 2017;53(2):147-55.
209. Abadi YS, Telmadarraiy Z, Vatandoost H, Chinikar S, Oshaghi M, Moradi M, et al. Hard Ticks on Domestic Ruminants and their Seasonal Population Dynamics in Yazd Province, Iran. Iranian journal of arthropod-borne diseases. 2010;4(1):66.
210. Albayrak H, Ozan E, Kurt M. An antigenic investigation of Crimean-Congo hemorrhagic fever virus (CCHFV) in hard ticks from provinces in northern Turkey. Tropical Animal Health and Production. 2010;42(7):1323-5.
211. Li Y, Wen X, Li M, Moumouni PFA, Galon EM, Guo Q, et al. Molecular detection of tick-borne pathogens harbored by ticks collected from livestock in the Xinjiang Uygur Autonomous Region, China. Ticks Tick Borne Dis. 2020;11(5):101478.
212. Ghashghaei O, Nourollahi-Fard SR, Khalili M, Sharifi H. A survey of ixodid ticks feeding on cattle and molecular detection of Coxiella burnetii from ticks in Southeast Iran. Turkish Journal of Veterinary & Animal Sciences. 2017;41(1):46-50.
213. AL-Fatlawi MAA, Ali MJ, Albayati HH. Morphological and phylogenetic study of *Hyalomma anatolicum* in Al-Najaf, Iraq. Iraqi Journal of Veterinary Sciences. 2018;32(2):261-6.
214. Singh NK, Jyoti, Haque M, Singh H, Rath SS, Ghosh S. A comparative study on cypermethrin resistance in Rhipicephalus (Boophilus) microplus and *Hyalomma anatolicum* from Punjab (India). Ticks Tick Borne Dis. 2014;5(2):90-4.
215. Perveen N, Muzaffar SB, Al-Deeb MA. Prevalence, Distribution, and Molecular Record of Four Hard Ticks from Livestock in the United Arab Emirates. Insects. 2021;12(11).
216. Orkun O. Molecular investigation of the natural transovarial transmission of tick-borne pathogens in Turkey. Veterinary Parasitology. 2019;273:97-104.
217. Moudgil P, Grakh K, Kumar R, Sharma M, Gupta R, Jindal N. First Molecular Confirmed Outbreak of Malignant Ovine Theileriosis in Sheep from North India. Acta Parasitol. 2023;68(3):527-34.
218. Jia Y, Wang S, Yang M, Ulzhan N, Omarova K, Liu Z, et al. First Detection of Tacheng Tick Virus 2 in Hard Ticks from Southeastern Kazakhstan. Kafkas Universitesi Veteriner Fakultesi Dergisi. 2022;28(1):139-42.
219. Ali Z, Maqbool A, Muhammad K, Khan MS, Younis M. Prevalence of Theileria annulata infected

hard ticks of cattle and buffalo in Punjab, Pakistan. The Journal of Animal & Plant Sciences. 2013;23(1):Anim.

220. Khattak RH, Liu Z, Teng L, Ahmed S, Shah SSA, Abdel - hakeem SS. Investigation on Parasites and Some Causes of Mortality in Cap tive Punjab urial (Ovis vignei punjabiensis), Pakistan. Iranian Journal of Parasitology. 2021;16(1):64.

221. Lotfi D, Karima K. Identification and incidence of hard tick species during summer season 2019 in Jijel Province (northeastern Algeria). J Parasit Dis. 2021;45(1):211-7.

222. Ashour R, Hamza D, Kadry M, Sabry MA. The Surveillance of Borrelia Species in Camelus dromedarius and Associated Ticks: The First Detection of Borrelia miyamotoi in Egypt. Vet Sci. 2023;10(2).

223. Mossaad E, Gaithuma A, Mohamed YO, Suganuma K, Umemiya-Shirafuji R, Ohari Y, et al. Molecular Characterization of Ticks and Tick-Borne Pathogens in Cattle from Khartoum State and East Darfur State, Sudan. Pathogens. 2021;10(5).

224. Norouzi M, Dayer MS, Ghaffarifar F. Molecular detection and characterisation of Theileria in hard ticks of small ruminants in Zarrin Dasht County, Southern Iran. Vet Med Sci. 2023;9(1):372-9.

225. Rehman A, Conraths FJ, Sauter-Louis C, Krücken J, Nijhof AM. Epidemiology of tick-borne pathogens in the semi-arid and the arid agro-ecological zones of Punjab province, Pakistan. Transbound Emerg Dis. 2019;66(1):526-36.

226. Farhadpour F, Telmadarraiy Z, Chinikar S, Akbarzadeh K, Moemenbellah-Fard MD, Faghihi F, et al. Molecular Detection of Crimean-Congo Haemorrhagic Fever Virus in Ticks Collected from Infested Livestock Populations in a New Endemic Area, South of Iran. Tropical Medicine & International Health. 2016;21(3):340-347.

227. Sajid MS, Iqbal Z, Khan MN, Muhammad G, Khan MK. Prevalence and associated risk factors for bovine tick infestation in two districts of lower Punjab, Pakistan. Prev Vet Med. 2009;92(4):386-91.

228. Abdallah MO, Niu Q, Yang J, Hassan MA, Yu P, Guan G, et al. Identification of 12 Piroplasms Infecting Ten Tick Species in China Using Reverse Line Blot Hybridization. Journal of Parasitology. 2017;103(3):221-7.

229. Khan A, Nasreen N, Niaz S, Shah SSA, Mitchell RD, III, Ayaz S, et al. Tick burden and tick species prevalence in small ruminants of different agencies of the Federally Administered Tribal Areas (FATA), Pakistan. International Journal of Acarology. 2019;45(6-7):374-80.

230. Shyma KP, Gupta JP, Parsani HR, Ankuya KJ, Singh V. Ivermectin resistance in the multi-host tick *Hyalomma anatolicum* (Acari: Ixodidae) in India. Ticks Tick Borne Dis. 2021;12(6):101791.

231. Farahi A, Ebrahimzade E, Nabian S, Hanafi-Bojd AA, Akbarzadeh K, Bahonar A. Temporal and Spatial Distribution and Species Diversity of Hard Ticks (Acari: Ixodidae) in the Eastern Region of Caspian Sea. Acta Tropica. 2016;164:1-9.

232. Jamil M, Idrees A, Khan S, Alwaili MA, Al-qahtani WS, Qadir ZA, et al. Distribution and identification of tick species infesting donkeys, in district Dera Ismail Khan, Khyber Pakhtunkhwa, Pakistan. Systematic and Applied Acarology. 2022;27(8):1518-24.

233. Ouhelli H, Pandey VS. Prevalence of cattle ticks in Morocco. Trop Anim Hlth Prod. 1982;14:151-4.

234. Banafshi O, Hanafi-Bojd AA, Karimi M, Faghihi F, Beik-Mohammadi M, Gholami S, et al. Tick Ectoparasites of Animals in Borderline of Iran-Iraq and Their Role on Disease Transmission. Journal of Arthropod-Borne Diseases. 2018;12(3):252.

235. Hayati MA, Hassan SM, Ahmed SK, Salih DA. Prevalence of ticks (Acari: Ixodidae) and Theileria annulata infection of cattle in Gezira State, Sudan. Parasite Epidemiol Control. 2020;10:e00148.
236. Yakhchali M, Rostami A, Esmailzadeh M. Diversity and seasonal distribution of ixodid ticks in the natural habitat of domestic ruminants in north and south of Iran. Revue De Medecine Veterinaire. 2011;162(5):229-35.
237. Becklund WW. Ticks of veterinary significance found on imports in the United States. 1968;54(3):622-8.
238. Abaker IA, Salih DA, Haj LME, Ahmed RE, Osman MM, Ali AM. Prevalence of Theileria annulata in dairy cattle in Nyala, South Darfur State, Sudan. Vet World. 2017;10(12):1475-80.
239. Alshammari A, Atuahene SA, Sheng QX, Jamil M, Ullah S, Ullah S, et al. Spatio-temporal epidemiological survey reveals high infestation and extensive species diversity of hard ticks infesting camels from Pakistan. South African Journal of Animal Science. 2023;53(2):174-84.
240. Perveen N, Muzaffar SB, Al-Deeb MA. Four Tick-Borne Microorganisms and Their Prevalence in Hyalomma Ticks Collected from Livestock in United Arab Emirates. Pathogens. 2021;10(8).
241. Telmadarraiy Z, Kooshki H, Edalat H, Vatandoost H, Bakhshi H, Faghihi F, et al. Study on Hard and Soft Ticks of Domestic and Wild Animals in Western Iran. Journal of Arthropod-Borne Diseases. 2023.
242. Aktas M, Dumanli N, Angin M. Cattle infestation by Hyalomma ticks and prevalence of Theileria in Hyalomma species in the east of Turkey. Vet Parasitol. 2004;119(1):1-8.
243. Zheng Z, Zeng W, Wang S, Tan W, Lu X, Kairullayev K, et al. Application of DNA barcodes in the genetic diversity of hard ticks (Acari: Ixodidae) in Kazakhstan. Experimental and Applied Acarology. 2024;92(3):547-54.
244. Hasheminasab SS, Moradi P, Wright I. A four year epidemiological and chemotherapy survey of babesiosis and theileriosis, and tick vectors in sheep, cattle and goats in Dehgolan, Iran. Ann Parasitol. 2018;64(1):43–8.
245. Perveen N, Muzaffar SB, Vijayan R, Al-Deeb MA. Microbial composition in *Hyalomma anatolicum* collected from livestock in the United Arab Emirates using next-generation sequencing. Parasit Vectors. 2022;15(1):30.
246. Shahhosseini N, Jafarbekloo A, Telmadarraiy Z, Chinikar S, Haeri A, Nowotny N, et al. Co-circulation of Crimean-Congo Hemorrhagic Fever virus strains Asia 1 and 2 between the border of Iran and Pakistan. Heliyon. 2017;3(11):e00439.
247. Tavassoli M, Tabatabaei M, Nejad BE, Tabatabaei MH, Najafabadi A, Pourseyed SH. Detection of Theileria annulata by the PCR-RFLP in ticks (Acari, Ixodidae) collected from cattle in West and North-West Iran. Acta Parasitologica. 2011;56(1):8-13.
248. Kayedi MH, Chinikar S, Mostafavi E, Khakifirouz S, Jalali T, Hosseini-Chegeni A, et al. Crimean-Congo Hemorrhagic Fever Virus Clade IV (Asia 1) in Ticks of Western Iran. J Med Entomol. 2015;52(5):1144-9.
249. Latif AA. Resistance to natural tick infestations in different breeds of cattle in the Sudan. Insect Science and Its Application. 1984;5(2):95-7.
250. Shiri A, Kheirandish F, Sazmand A, Kayedi MH, Hosseini-Chegeni A. Molecular identification of hemoparasites in ixodid ticks in Iran. Veterinary Parasitology: Regional Studies and Reports. 2024;47.
251. Aktas M, Altay K, Dumanli N, Kalkan A. Molecular detection and identification of Ehrlichia and

Anaplasma species in ixodid ticks. Parasitol Res. 2009;104(5):1243-8.
252. Zeb I, Parizi LF, Israr M, da Silva Vaz I, Ali A. Cross-species immunoprotective antigens (subolesin, ferritin 2 and P0) provide protection against Rhipicephalus sanguineus sensu lato. Parasites & Vectors. 2024;17(1).
253. Mohammed RR, Enferadi A, Sidiq KR, Sarani S, Khademi P, Jaydari A, et al. Molecular Detection of Francisella tularensis Isolated from Ticks of Livestock in Kurdistan Region, Iraq. Vector Borne Zoonotic Dis. 2023.
254. Islam MK, Alim MA, Tsuji N, Mondal MM. An investigation into the distribution, host-preference and population density of ixodid ticks affecting domestic animals in Bangladesh. Trop Anim Health Prod. 2006;38(6):485-90.
255. Mustafa I, Shabbir RMK, Subhani M, Ahmad I, Raza A, Jamil S, et al. Seasonal Activity of Tick Infestation in Goats and Buffalo of Punjab Province (District Sargodha), Pakistan. Kafkas Universitesi Veteriner Fakultesi Dergisi. 2014;20(5):655-62.
256. Sultankulova KT, Shynybekova GO, Kozhabergenov NS, Mukhami NN, Chervyakova OV, Burashev YD, et al. The Prevalence and Genetic Variants of the CCHF Virus Circulating among Ticks in the Southern Regions of Kazakhstan. Pathogens. 2022;11(8).
257. Abdigoudarzi M. Detection of Naturally Infected Vector Ticks (Acari: Ixodidae) by Different Species of Babesia and Theileria Agents from Three Differ- ent Enzootic Parts of Iran. Journal Of Arthropod-borne Diseases. 2013;7(2):164-72.
258. Jafarbekloo A, Ramzgouyan MR, Shirian S, Tajedin L, Bakhshi H, Faghihi F, et al. Molecular Characterization and Phylogenetic Analysis of Theileria spp. and Babesia spp. Isolated from Various Ticks in Southeastern and Northwestern Regions of Iran. Vector Borne Zoonotic Dis. 2018;18(11):595-600.
259. Nourollahi Fard SR, Omid Ghashghaei O, Khalili M, Sharifi H. Tick Diversity and Detection of Coxiella burnetii in Ticks of Small Ruminants Using Nested Trans PCR in Southeast Iran. Tropical Biomedicine. 2016;33(3):506-511.
260. Razmi G, Pourhosseini M, Yaghfouri S, et al. Molecular detection of Theileria spp. and Babesia spp. in sheep and ixodid ticks from the northeast of Iran. The Journal of parasitology. 2013;99(1): 77-81.
261. Ghafar A, Gasser RB, Rashid I, Ghafoor A, Jabbar A. Exploring the prevalence and diversity of bovine ticks in five agro-ecological zones of Pakistan using phenetic and genetic tools. Ticks Tick Borne Dis. 2020;11(5):101472.
262. Iqbal Z, Kayani AR, Akhter A, Qayyum M. Prevalence and Distribution of Hard Ticks and Their Associated Risk Factors in Sheep and Goats from Four Agro-Climatic Zones of Khyber Pakhtunkhwa (KPK), Pakistan. Int J Environ Res Public Health. 2022;19(18):11759.
263. Tafesse B. Survey on the distribution of ticks of domestic animals in the eastern zone of Ethiopia. Trop Anim Health Prod. 1996;28(2):145-6.
264. Patra G , Polley S , Efimova MA , Sahara A, Debbarma A, et al. Prevalence and molecular detection of tick borne pathogens in goats and ticks from different parts of North Eastern regions of India. International Journal of Acarology. 2022(2):48.
265. Patra G, Ghosh S, Polley S, Priyanka, Borthakur SK, Choudhary OP, Arya RS. Molecular detection and genetic characterization of Coxiella-like endosymbionts in dogs and ticks infesting dogs in Northeast India. Exp Appl Acarol. 2022;86(4):549-566.
266. Patel G, Shanker D, Jaiswal AK, Sudan V, Verma SK. Prevalence and seasonal variation in ixodid

ticks on cattle of Mathura district, Uttar Pradesh. J Parasit Dis. 2013;37(2):173-6.
267. Gaur RS, Sangwan AK, Sangwan N, Kumar S. Acaricide resistance in Rhipicephalus (Boophilus) microplus and Hyalomma anatolicum collected from Haryana and Rajasthan states of India. Exp Appl Acarol. 2016;69(4):487-500.
268. Shekhar S. Epidemiological Study on Ticks infestations in Cattle of Jharkhand and Therapeutic Evaluation of Cypermethrin, Deltamethrin and Flumethrin. Journal of Animal Research. 2020, 10(6).
269. Robson J, Robb JM, Al-Wahayyib T. Ticks (Ixodoidea) of domestic animals in Iraq. 2. Summer infestations in the Liwas of Hilla, Karbala, Diwaniya and Nasiriya. J Med Entomol. 1968;5(1):27-31.
270. Dabaja MF, Greco G, Blanda V, Tempesta M, Bayan A, Torina A, Vesco G, D'Agostino R, Lelli R, Ezzedine M, Mortada H, Raoult D, Fournier PE, Mortada M. Multispacer sequence typing of Coxiella burnetii from milk and hard tick samples from ruminant farms in Lebanon. Vet Ital. 2020;56(4):289-296.
271. Fernández de Mera IG, Blanda V, Torina A, Dabaja MF, El Romeh A, Cabezas-Cruz A, et al. Identification and molecular characterization of spotted fever group rickettsiae in ticks collected from farm ruminants in Lebanon. Ticks Tick Borne Dis. 2018;9(1):104-108.
272. Trukhachev VI , Tokhov YM , Lutsuk SN , Dyachenko YV. Distribution and ecological characteristics of hyalomma ixodid ticks in the ecosystems of the stavropol region.Ug Rossii: Ekologia, 2016.
273. Al-Khalifa MS, Al-Asgah NA, Diab FM. Hyalomma (Hyalommina) arabica, the Arabian goat and sheep tick) distribution and abundance in Saudi Arabia. J Med Entomol. 1986;23(2):220-1.
274. Bughdadi FA. A study on fat body cells ultrastructure in female camel tick (Hyalomma anatolicum anatolicum). Journal of Entomology and Nematology. 2012;4(4).
275. lghali A, Hassan SM. Ticks (Acari: Ixodidae) infesting camels (Camelus dromedarius) in Northern Sudan. Onderstepoort J Vet Res. 2009;76(2):177-85.
276. Sayin F, Dinçer S, Karaer Z, Cakmak A, Inci A, Yukari BA, et al. Vatansever Z, Nalbantoglu S. Studies on the epidemiology of tropical theileriosis (Theileria annulata infection) in cattle in Central Anatolia, Turkey. Trop Anim Health Prod. 2003;35(6):521-39.
277. Luo J, Yin H, Guan G, Zhang Q, Lu W. Description of a new Babesia sp. infective for cattle in China. Parasitol Res. 2002;88(13 Suppl 1):S13-5.
278. Fard SN, Khalili M. PCR-Detection of Coxiella burnetii in Ticks Collected from Sheep and Goats in Southeast Iran. Iran J Arthropod Borne Dis. 2011;5(1):1-6.
279. Zarei F, Ganjali M, Nabavi R. Identification of Theileria Species in Sheep and Vector Ticks Using PCR Method in Zabol, Eastern Iran. J Arthropod Borne Dis. 2019;13(1):76-82.
280. Ganjali M, Dabirzadeh M, Sargolzaie M. Species Diversity and Distribution of Ticks (Acari: Ixodidae) in Zabol County, Eastern Iran. J Arthropod Borne Dis. 2014;8(2):219-23.
281. Mirzaei M, Khedri J. Ixodidae ticks in cattle and sheep in Sistan and Baluchestan Province (Iran). Vet Ital. 2014;50(1):65-8.
282. Tajedin L, Bakhshi H, Faghihi F, Telmadarraiy Z. High infection of Anaplasma and Ehrlichia spp. among tick species collected from different geographical locations of Iran. Asian Pacific Journal of Tropical Disease. 2016;6(10):787-792.
283. Faghihi F, Telmadarraiy Z, Chinikar S, Nowotny N, Shahhosseini N. Spatial and Phylodynamic Survey on Crimean-Congo Hemorrhagic Fever Virus Strains in Northeast of Iran. Jundishapur Journal of Microbiology. 2018;In Press(In Press).
284. Atefeh, Khazeni, Zakieh, Telmadarraiy, Mohammad, Ali, et al. Molecular detection of Ehrlichia

canis in ticks population collected on dogs in Meshkin-Shahr, Ardebil Province, Iran. Journal of Biomedical Science & Engineering. 2013.
285. Abdoli R, Bakhshi H, Kheirandish S, Faghihi F, Sedaghat MM. Circulation of Brucellaceae, Anaplasma and Ehrlichia spp. in borderline of Iran, Azerbaijan, and Armenia. Asian Pacific Journal of Tropical Medicine. 2021;14(5):223-230.
286. Spitalska E, Namavari MM, Hosseini MH, Shad-Del F. Molecular surveillance of tick-borne diseases in Iranian small ruminants. Small Ruminant Research. 2005;57(2-3):245-248.
287. Tahmasebi F, Ghiasi SM, Mostafavi E, Moradi M, Piazak N, Mozafari A, et al. Molecular epidemiology of Crimean- Congo hemorrhagic fever virus genome isolated from ticks of Hamadan province of Iran. J Vector Borne Dis. 2010;47(4):211-6.
288. Telmadarraiy Z, Moradi AR, Vatandoost H, Mostafavi E, Chinikar S. Crimean-Congo hemorrhagic fever: a seroepidemiological and Molecular survey in Bahar, Hamadan province of Iran. Asian Journal of Animal & Veterinary Advances. 2008;3(5):321-327.
289. Biglari P, Chinikar S, Belqeiszadeh H, Telmadarraiy Z, Mostafavi E, Ghaffari M, et al. Phylogeny of tick-derived Crimean-Congo hemorrhagic fever virus strains in Iran. Ticks Tick Borne Dis. 2016;7(6):1216-1221.
290. Ranjbar R, Anjomruz M, Enayati AA, Khoobdel M, Rafinejad A, Rafinejad J. Anaplasma Infection in Ticks in Southeastern Region of Iran. J Arthropod Borne Dis. 2020;14(2):126-133.
291. Khalili M, Rezaei M, Akhtardanesh B, Abiri Z, Shahheidaripour S. Detection of Coxiella burnetii (Gammaproteobacteria: Coxiellaceae) in ticks collected from infested dogs in Kerman, Southeast of Iran. Persian Journal of Acarology. 2018;7(1).
292. Saghafipour A, Mousazadeh-Mojarrad A, Arzamani N, Telmadarraiy Z, Rajabzadeh R, Arzamani K. Molecular and seroepidemiological survey on Crimean-Congo Hemorrhagic Fever Virus in Northeast of Iran. Med J Islam Repub Iran. 2019;33:41.
293. Abedi V, Razmi G, Seifi H, Naghibi A. Molecular and serological detection of Theileria equi and Babesia caballi infection in horses and ixodid ticks in Iran. Ticks Tick Borne Dis. 2014;5(3):239-44.
294. Fakoorziba MR, Golmohammadi P, Moradzadeh R, Moemenbellah-Fard MD, Azizi K, Davari B, et al. Reverse transcription PCR-based detection of Crimean-Congo hemorrhagic fever virus isolated from ticks of domestic ruminants in Kurdistan province of Iran. Vector Borne Zoonotic Dis. 2012;12(9):794-9.
295. Nasser H, Hashemi Saeed A, Mohammad A. Molecular detection of Theileria ovis and T. lestoquardi in vector ticks in Lorestan province, Iran. iran. 2014;:78-83.
296. Song RQ, Zhai XJ, Li CS, Ge T, Gan L, et al. Comparative analysis of biological characteristics of Hyalomma asiaticum and H. anatolicum Xinjiang isolates at different developmental stages. 2022;369-378.
297. Jamil M, Bhatti AH, Zia R, Shabana K, Kashif M, Ullah N, et al. Collection, prevalence and identifying hard tick species among small ruminants in Southern Khyber Pakhtunkhwa, Pakistan. Bioscience Research. 2022;19(2):893-898.
298. S.P. Morzaria, A.A. Latif, F. Jongejan, et al. Transmission of a Trypanosoma sp. to cattle by the tick Hyalomma anatolicum anatolicum. Veterinary Parasitology. 1986;19(1-2):13-21.
299. 阿力米江·沙克，巴音查汗．阿合奇县色乡牦牛环形泰勒虫病流行病学调查报告．当代畜牧. 2012(04):25-7.
300. 阿力木江·加帕尔．鄯善县绵羊染蜱情况调查及重组 Cathepsin L 免疫的抗蜱效果分析［硕

士]2022.
301. 仇晓飞, 林汉亮, 罗金, 马力克·艾则孜, 康逢义, 倪军, et al. 新疆边境地区蜱携带梨形虫种类的分子鉴定. 动物医学进展. 2022;43(01):31-7.
302. 邓岗领 蒋, 叶瑞玉,乔月金,于心. 新疆叶尔羌河流域蜱螨区系调查报告. 地方病通报. 1999(03):58-60.
303. 杜兰兰. 巴州牦牛三个试验点蜱传牛环形泰勒虫病的流行病学调查 [硕士]: 新疆农业大学; 2018.
304. 苟惠天, 薛慧文, 殷宏, 孙晓林, 罗建勋. 基于 ITS 和 COI基因对于我国璃眼蜱的分类研究. 中国兽医科学. 2016;46(05):563-7.
305. 关贵全 殷, 罗建勋,吕文顺,张其才,马米玲,袁改玲,鲁炳义,王远江,木合塔尔. 羊的大型巴贝虫未定种的形态学和致病性初步研究. 中国兽医科技. 2001(11):35-6.
306. 郭庆勇. 新疆牛环形泰勒虫病早期诊断方法及其媒介蜱生物学特性与防治研究 [博士]2017.
307. 海尼木古力·艾合买提, 谢小婉, 郭子涵, 杜兰兰, 李才善, 闻秀秀, et al. 托克逊县牛环形泰勒虫媒介蜱形态鉴定及病原检测. 黑龙江畜牧兽医. 2018(14):120-2+247.
308. 李凯瑞. 新疆部分地区璃眼蜱分类鉴定及携带部分病原检测 [硕士]2019.
309. 何晓东. 托克逊县部分地区牛环形泰勒虫病的流行病学调查 [硕士]2015.
310. 姜媛, 马钰, 党娜娜, 祖力牙, 刘一凡, 范士龙, et al. 伊吾县部分养殖场感染绵羊无浆体病的流行病学调查. 新疆畜牧业. 2020;35(02):27-31.
311. 姜在阶, 常崇艳, 白春玲, 陈晓端. 璃眼蜱幼虫的形态学研究. 北京师范大学学报(自然科学版). 1988(02):69-74.
312. 蒋玉曦. 巴州塔里木马鹿蜱种类鉴定及其携带部分病原检测 [硕士]2020.
313. 卡丽比努尔·尔肯. 南疆驴常见寄生虫鉴定及其流行病学调查 [硕士]2015.
314. 孔昭敏 陈, 金根源,史丕裕,周新荣,赖学琴. 新疆蜱媒自然疫源性疾病的调查研究. 地方病通报. 1987(03):1-4.
315. 黎唯, 孙毅, 张桂林, 许荣满. 中国璃眼蜱属(蜱螨亚纲:硬蜱科)研究——附新记录种盾陷璃眼蜱 Hyalomma excavatum Koch,1844 的描述. 寄生虫与医学昆虫学报. 2015;22(02):94-103.
316. 李冰, 阿布力克木, 雷刚, 阿不力米提, 张渝疆. 塔里木盆地蜱类群落结构的研究. 寄生虫与医学昆虫学报. 2006(03):150-7.
317. 李连峰. 荆门蜱病毒分子流行病学调查和经媒介蜱传播规律的研究 [硕士]2020.
318. 刘丹丹, 布威佐拉古丽·萨依木, 张伟, editors. 新疆喀什地区疏勒县牛环形泰勒虫病及媒介蜱的初步调查. 第十一届全国寄生虫学青年工作者学术研讨会; 2018; 中国云南昆明.
319. 刘继荣, 米来, 王平福, 张艳艳, 薄新文. 准噶尔盆地硬蜱区系考察与名录记述. 中国动物传染病学报. 2013;21(01):60-5.
320. 刘洋. 河南省发热伴血小板减少综合征流行区蜱分布及携带新布尼亚病毒状况 [硕士]2012.
321. 吕继洲, 吴绍强, 张永宁, 王振宝, 冯春燕, 王彩霞, et al. 小亚璃眼蜱、亚洲璃眼蜱和残缘璃眼蜱的分子生物学鉴定. 中国畜牧兽医. 2013;40(06):7-14.
322. 罗建勋, 关贵全, 马米玲, 张其才, 吕文顺. 小亚璃眼蜱和亚洲璃眼蜱对环形泰勒虫传播能力的研究. 中国兽医科技. 2003(02):22-3.
323. 马钰. 新疆圈养普氏野马体内外寄生虫感染情况调查及驱虫方案的制定 [硕士]2021.
324. 庞道毛 艾, 陈国仕. 新疆北部蜱类的区系及垂直分布. 四川动物. 1985(03):15-8.
325. 曲志强, 林汉亮, 许肖枫, 马力克·艾则孜, 罗毅, 马站, et al. 新疆部分地区蜱传斑点热立克次体的分子流行病学研究. 甘肃农业大学学报. 2019;54(05):10-6.
326. 热孜万, 阿布力克木, 李冰, 雷刚, 阿不力米提, 梁新海, et al. 塔里木盆地蜱类群落组成和

分布. 中国媒介生物学及控制杂志. 2006(05):390-4.
327. 沙它尔·卡哈尔. 吐鲁番市周边地区牛巴贝斯虫病及其媒介蜱感染情况的调查研究 [硕士]2014.
328. 史智勇 杨, 李强,赵红斌. 甘肃省媒介硬蜱的种类与地理分布. 中国兽医科技. 2004(08):48-9.
329. 宋瑞其. 基于 IFN-γ 分子佐剂的重组 Cathepsin L 免疫抗蜱效果分析研究 [博士]2021.
330. 宋瑞其, 翟雪洁, 李才善, 葛婷, 甘露, 张梦圆, et al. 亚洲璃眼蜱和小亚璃眼蜱新疆地理株各发育阶段生物学特性的比较分析. 中国寄生虫学与寄生虫病杂志. 2022;40(03):369-78.
331. 孙明. 蜱病原真菌的分离、鉴定及其生物学特性研究 [硕士]2011.
332. 孙明, 聂英, 张伟, 马超, 程生忠. 民勤县畜禽寄生虫的种类厘定. 畜牧兽医杂志. 2013;32(02):34-8.
333. 孙明, 王淑芳, 王多全, 杨永彪, 马超. 甘肃省武威市蜱及蜱媒病种类及分布的初步调查. 中国动物检疫. 2016;33(02):23-5.
334. 瓦热斯·吐尔松, 李永畅, 朱玉涛, 图尔荪·萨迪尔, 杨红霞, 巴音查汗. 新疆昌吉部分地区马梨形虫病检测初报. 中国动物传染病学报. 2016;24(01):75-9.
335. 王冰洁. 新疆璃眼蜱种属鉴定、进化分析及其携带泰勒虫的分子检测 [硕士]2016.
336. 王冰洁, 朱玉涛, 巴音查汗. 小亚璃眼蜱的综合鉴定. 中国畜牧兽医学会 2014 年学术年会; 2014; 中国广东广州.
337. 王冰洁, 朱玉涛, 吉尔格力, 伊春阳, 巴音查汗. 小亚璃眼蜱源性牛环形泰勒虫裂殖子表面抗原(Tams1)基因检测及同源性分析. 畜牧与兽医. 2015;47(12):9-11.
338. 王冰洁, 朱玉涛, 刘梦丽, 宋瑞其, 巴音查汗. 新疆小亚璃眼蜱的鉴定及其携带牛环形泰勒虫病原 DNA 检测. 中国兽医学报. 2015;35(12):1939-42+47.
339. 王振宝. 小亚璃眼蜱的生活史和抗菌多肽活性的研究及牛环形泰勒虫病二温式 PCR 检测方法的建立 [硕士]2009.
340. 闻秀秀. 新疆部分地区三种硬蜱携带病原、进化分析及绵羊无浆体病的分子诊断 [硕士]2019.
341. 闻秀秀, 谢小婉, 呼尔查, 朱玉涛, 瓦热斯·吐尔松, 巴音查汗. 实验条件下小亚璃眼蜱的生物学特性研究. 草食家畜. 2016(06):53-7.
342. 吴永红, 刘玲, 王真, 巴音查汗. 南疆部分散养户牛场梨形虫及其媒介蜱感染情况的调查. 新疆农业科学. 2011;48(10):1918-22.
343. 肖晨冬. 四川九龙县蜱的鉴定及蜱和牦牛中蜱媒病原的分子检测 [硕士]2022.
344. 谢小婉. 新疆部分地区优势种璃眼蜱生物学特性及携带牛环形泰勒虫的遗传进化分析 [硕士]2018.
345. 谢小婉, 朱马别克·胡斯曼, 加那尔·努和买提, 木哈里·吾汗, 王志斌. 小亚璃眼蜱形态学鉴定及其携带牛环形泰勒虫病原的检测. 新疆畜牧业. 2021;36(05):25-8.
346. 叶瑞玉, 于心, 陈饮如, 张自建, 曹汉礼, 热孜万, 陈伟. 新疆南部地区婢螨区系及医学意义. 地方病通报. 1995(04):30-4+19.
347. 叶晓磊, 谭伟龙, 石清明. 我国西部地区蜱类携带斑点热群立克次体现状. 寄生虫与医学昆虫学报. 2023;30(02):121-8.
348. 于昊江. 昌吉部分地区牛感染蜱传绵羊无浆体的情况调查 [硕士]2019.
349. 张峰. 小海子垦区牛梨形虫感染情况调查及媒介蜱种类鉴定 [硕士]: 石河子大学; 2021.
350. 张艳艳. 准噶尔盆地硬蜱分类研究 [硕士]: 石河子大学; 2013.
351. 张渝疆, 曹汉礼, 戴翔, 艾择孜, 蒋卫, 阿布力克木, et al. 塔里木盆地蜱类群落的分型和多样性分析. 中国寄生虫学与寄生虫病杂志. 2006;24(6):404-9.
352. 赵建新, 巴音查汗, 吴国梁, 祁巧芬. 吐鲁番地区牛焦虫病流行病学调查. 新疆畜牧业.

2012(12):42-4.
353. 罗金，谭阳春，仇晓飞，马雨楠，黄学智，禹晓宁, et al. 甘肃省庆阳地区泰勒虫病流行趋势及分子特征分析. 中国兽医科学. 2020;50(8):946-51.
354. 谢小婉，刘世芳，杜兰兰，闻秀秀，郭子涵，李才善, et al. 新疆优势种小亚璃眼蜱龄期超微结构的观察与鉴定. 中国预防兽医学报. 2018;40(4):357-60.
355. 吐热古丽·阿力木，温丽翠，诺明达来，特力克，刘凯强，金敏, et al. 新疆吐鲁番市艾丁湖镇春季牛体表蜱种调查. 中国动物检疫. 2022;39(11):17-21.
356. 李有全，彭欲率，刘志杰，关贵全，杨吉飞，陈泽, et al. 中国部分地区羊泰勒虫病的流行病学调查及分类鉴定. 中国农业科学,45(16),3422-3429.
357. 吴泽功，林汉亮，罗金，马力克·艾则孜，任巧云，罗毅, et al. 新疆北部地区蜱传嗜吞噬细胞无浆体病原学检测与分子遗传进化分析. 动物医学进展. 2021;42(02):19-25.
358. 刘凯强，俞进，段真真，金敏，李佳，巴音查汗·盖力克. 新疆阿克苏地区蜱和羊感染无浆体的分子流行病学调查. 畜牧与饲料科学. 2023;44(3):122-8.
359. 张艳艳，阿德力·克坦，达木，巴合提，王开胜，刘继荣. 塔城地区硬蜱种类调查与群落分析. 石河子大学学报(自然科学版). 2013;31(04):457-62.
360. 贵有军，史深，罗勇军，王希江，张晓兵，王诚, et al. 新疆蜱传疾病及蜱媒防制. 中国动物传染病学报. 2023;31(03):213-20.
361. 陈联宏，阿依肯，赵明. 昌吉州蜱类群落组成和分布. 中华卫生杀虫药械. 2017;23(04):376-7.

Reference book:

362.孙毅，许荣满. 中国动物志・蛛形纲・蜱螨亚纲・硬蜱目. 北京：科学出版社.

Figure S2: PRISMA flow diagram of study selection process.

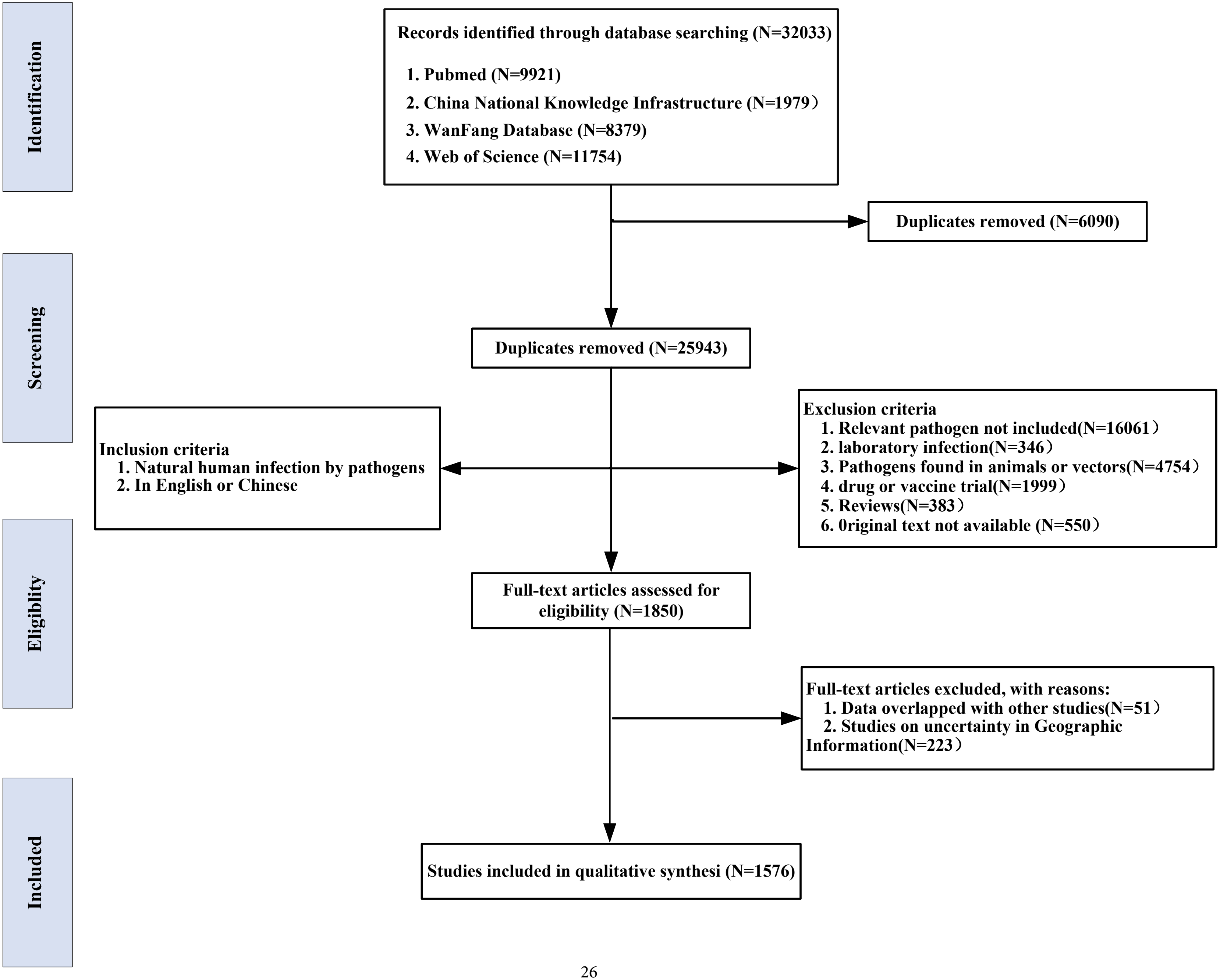

Table S4: Search terminology used for human infections.

Search	English (Pubmed, Web of Science) [Title/Abstract]	Chinese (CHKI and WanFang) [Title/Abstract]
#1 (pathogens and Diseases)	Anaplasma ovis OR Anaplasma phagocytophilum OR Anaplasma bovis OR Ehrlichia ruminantium OR Ehrlichia chaffeensis OR Ehrlichia canis OR Ehrlichia muris OR Babesia bigemina OR Babesia bovis OR Babesia motasi OR Borrelia burgdorferi OR Lyme disease OR Borrelia crocidurae OR Borrelia miyamotoi OR Coxiella burnetii OR Francisella tularensis subsp novicida OR Rickettsia conorii OR Rickettsia slovaca OR Rickettsia massiliae OR Rickettsia aeschlimannii OR Rickettsia raoultii OR Rickettsia africae OR Rickettsia amblyommii OR Tick-borne encephalitis virus OR Tacheng Tick Virus 2 OR Powassan virus ORCrimean-Congo hemorrhagic fever virus OR Crimean-Congo hemorrhagic fever OR Xinjiang hemorrhagic fever virus OR Xinjiang hemorrhagic fever OR	绵羊无形体 or 噬吞噬细胞无形体 or 人粒细胞无形体 or 牛无形体 or 反刍动物埃立克体 or 查菲埃立克体 or 犬埃立克体 or 鼠埃立克体 or 双芽巴贝斯虫 or 牛巴贝斯虫 or 莫氏巴贝斯虫 or 伯氏疏螺旋体 or 莱姆病 or 克氏疏螺旋体 or 宫本疏螺旋体 or 贝氏柯克斯体 or Q 热 or 土拉热弗朗西斯菌新凶手亚种 or 康氏立克次体 or 钮扣热 or 马赛立克次体 or 斯洛伐克立克次体 or 埃氏立克次体 or 劳氏立克次体 or 非洲立克次体 or 钝缘蜱立克次体 or 蜱传脑炎 or 森林脑炎 or 塔城蜱病毒 2 型 or 波瓦桑病毒 or 克里米亚刚果出血热病毒 or 新疆出血热病毒 or 新疆出血热
#2	Morocco OR Algeria OR Sudan OR Egypt OR Ethiopia OR Somalia OR France OR Bulgaria OR Greece OR Russia OR Israel OR Saudi Arabia OR Yemen OR Oman OR Iraq OR Turkey OR Iran OR Kazakhstan OR Tajikistan OR Turkmenistan OR Afghanistan OR Uzbekistan OR Kyrgyzstan OR Nepal OR Pakistan OR India OR Bangladesh OR China OR United Arab Emirates OR Lebanon OR Cyprus OR United States of America	
#3	human OR case OR zoonoses OR zoonotic OR patients	人 OR 病例 OR 患者 OR 人畜共患
#4	#1 AND #2 AND #3	#1 AND #3

Text S2: References for Human Infection

From the listed studies, we extract data including the primary author, year of publication, study type, confirmation date of infected individuals or cases, geographical information, and the number of individuals testing positive for specific pathogens.

[1] Centers for Disease Control and Prevention (CDC). Arboviral Disease—United States, 1994. Mmwr-morbidity And Mortality Weekly Report. 1995;44(35):641-4.

[2] Centers for Disease Control and Prevention (CDC). Lyme Disease—United States, 1994. Mmwr-morbidity And Mortality Weekly Report. 1995;44(24):459-62.

[3] Centers for Disease Control and Prevention (CDC). Lyme Disease—United States, 1995. Archives of Dermatology. 1996;132(11).

[4] Centers for Disease Control and Prevention (CDC). Lyme Disease—United States, 2003-2005. JAMA. 2007;298(3).

[5] Centers for Disease Control and Prevention (CDC). West Nile Virus and Other Arboviral Diseases—United States, 2012. Mmwr-morbidity And Mortality Weekly Report. 2013;62(25):513-7.

[6] Centers for Disease Control and Prevention (CDC). Three Sudden Cardiac Deaths Associated With Lyme Carditis—United States, November 2012 to July 2013. Mmwr-morbidity And Mortality Weekly Report. 2013;62(49):993-6.

[7] Aarthi P, Bagyalakshmi R, Mohan KR, Krishna M, Nitin M, Madhavan HN, et al. First case series of emerging Rickettsial neonatal sepsis identified by polymerase chain reaction-based deoxyribonucleic acid sequencing. Indian Journal of Medical Microbiology. 2013;31(4):343-8.

[8] Abadoglu O, Engin A. The frequency of self-reported allergic diseases in patients with crimean-congo haemorrhagic fever. Allergol Immunopathol (Madr). 2009;37(5):234-8.

[9] Abbass H, Selim SAK, Sobhy MM, El-Mokhtar MA, Elhariri M, Abd-Elhafeez HH. High prevalence of Coxiella burnetii infection in humans and livestock in Assiut, Egypt: A serological and molecular survey. Veterinary World. 2020;13(12):2578-86.

[10] Abbott KC, Vukelja SJ, Smith CE, McAllister CK, Konkol KA, O'Rourke TJ, et al. Hemophagocytic syndrome: A cause of pancytopenia in human ehrlichiosis. American Journal of Hematology. 2006;38(3):230-4.

[11] Abbott KC, Vukelja SJ, Smith CE, McAllister CK, Konkol KA, Orourke TJ, et al. Hemophagocytic syndrome: a cause of pancytopenia in human ehrlichiosis. American Journal of Hematology. 1991;38(3):230-4.

[12] Abdiyeva K, Turebekov N, Dmitrovsky A, Tukhanova N, Shin A, Yeraliyeva L, et al. Seroepidemiological and molecular investigations of infections with Crimean-Congo haemorrhagic fever virus in Kazakhstan. International Journal of Infectious Diseases. 2019;78:121-7.

[13] Abernathy H, Alejo A, Arahirwa V, Mansour O, Brown-Marusiak A, Giandomenico D, et al. "Leopards do not change their spots:" tick borne disease symptomology case report. Bmc Infectious Diseases. 2022;22(1).

[14] Abraham, Vasantha K, Abraham. Brief, recurrent, and spontaneous episodes of loss of consciousness in a healthy young male. International Medical Case Reports Journal. 2010:71–6.

[15] Abu Rmeileh A, Khoury T, Meir K, Drori A, Shalit M, Benenson S, et al. Familial Q fever clustering with variable manifestations imitating infectious and autoimmune disease. Clinical Microbiology and Infection. 2015;21(5):459-63.

[16] Abuova G, Pshenichnaya N, Irsimbetova N, Apsatarov Z. Clinical and epidemiological aspects of Crimean-Congo hemorrhagic fever in pregnant women in South Kazakhstan. International Journal of Infectious Diseases. 2012;16:E66-E.

[17] Abuova GN, Berdaliyeva FA, Polukchi TV, Aliyev DS, Raymkulov GS, Kulemin MV, et al. Seroprevalence of Crimean-Congo hemorrhagic fever virus in the population of Turkestan region. Le infezioni in medicina. 2024;32(1):83-9.

[18] Abushahba MFN, Abdelbaset AE, Rawy MS, Ahmed SO. Cross-sectional study for determining the prevalence of Q fever in small ruminants and humans at El Minya Governorate, Egypt. BMC research notes. 2017;10(1):538-.

[19] Afrasiabian S, Esmaeili S, Hajibagheri K, Hadizadeh N, Lotfi G, Veysi A. Endocarditis Caused by Coxiella burnetii: A Case Report in Western Iran. Journal of Arthropod-Borne Diseases. 2024.

[20] Ağargün M, Karahocagil M, Karsen H, Beşiroğlu L, Akdeniz H. Lyme neuroborreliosis presenting chiefly with neuropsychiatric symptoms displaying difficulties in diagnosis: Report of two cases. Journal of Pediatric Infectious Diseases. 2015;01(03):177-83.

[21] Aguero-Rosenfeld ME, Donnarumma L, Zentmaier L, Jacob J, Frey M, Noto R, et al. Seroprevalence of antibodies that react with Anaplasma phagocytophila, the agent of human granulocytic ehrlichiosis, in different populations in Westchester County, New York. Journal of Clinical Microbiology. 2002;40(7):2612-5.

[22] Aguero-Rosenfeld ME, Nowakowski J, Bittker S, Cooper D, Nadelman RB, Wormser GP. Evolution of the serologic response to Borrelia burgdorferi in treated patients with culture-confirmed erythema migrans. Journal of Clinical Microbiology. 1996;34(1):1-9.

[23] Ahmed A, Ali Y, Salim B, Dietrich I, Zinsstag J. Epidemics of Crimean-Congo Hemorrhagic Fever (CCHF) in Sudan between 2010 and 2020. Microorganisms. 2022;10(5).

[24] Ahmed AA, McFalls JM, Hoffmann C, Filone CM, Stewart SM, Paragas J, et al. Presence of broadly reactive and group-specific neutralizing epitopes on newly described isolates of <i>Crimean-Congo hemorrhagic fever virus</i>. Journal of General Virology. 2005;86:3327-36.

[25] Ahn B, Kim GB, Lee HJ, Choi EH. A Case of Lyme Disease Complicated with Atrioventricular Block in a 13-year-old Boy. Pediatric Infection and Vaccine. 2020;27(3):184-9.

[26] Ai CX, Zhang WF, Zhao JH. Sero-epidemiology of Lyme disease in an endemic area in China. Microbiology and Immunology. 1994;38(7):505-9.

[27] Aijazi I, Al Shama FMA, Shandala Y, Varghese RM. Crimean-Congo haemorrhagic fever presenting with acute compartment syndrome of the extremities (think beyond normal infections). Bmj Case Reports. 2020;13(2).

[28] Akamine CM, Perez ML, Lee JH, Ing MB. Q Fever in Southern California: a Case Series of 20 Patients from a VA Medical Center. American Journal of Tropical Medicine and Hygiene. 2019;101(1):33-9.

[29] Akar N, Caliskan E, Ozturk CE, Ankarali H, Kilincel O, Oksuz S, et al. Seroprevalence of hantavirus and Borrelia burgdorferi in Düzce (Turkey) forest villages and the relationship with sociodemographic features. Turkish Journal of Medical Sciences. 2019;49(2):483-9.

[30] Akbarian Z, Ziay G, Schauwers W, Noormal B, Saeed I, Qanee AH, et al. Brucellosis and Coxiella

burnetii Infection in Householders and Their Animals in Secure Villages in Herat Province, Afghanistan: A Cross-Sectional Study. Plos Neglected Tropical Diseases. 2015;9(10).

[31] Ake JA, Massung RF, Whitman TJ, Gleeson TD. Difficulties in the diagnosis and management of a US servicemember presenting with possible chronic Q fever. Journal of Infection. 2010;60(2):175-7.

[32] Aksoy HZ, Yilmaz G, Aksoy F, Koksal I. Crimean-Congo haemorrhagic fever presenting as epididymo-orchitis. Journal of Clinical Virology. 2010;48(4):282-4.

[33] Aktas F, Aktas T. The pulmonary findings of Crimean-Congo hemorrhagic fever patients with chest X-ray assessments. Radiologia Medica. 2019;124(9):826-32.

[34] Aktaş T, Aktaş F, Özmen Z, Altunkaş A, Kaya T, Demir O. Thorax CT findings in patients with Crimean-Congo hemorrhagic fever (CCHF). Springerplus. 2016;5(1):1823.

[35] Al Dabal LM, Shahmirzadi MRR, Baderldin S, Abro A, Zaki A, Dessi Z, et al. Crimean-Congo Hemorrhagic Fever in Dubai, United Arab Emirates, 2010: Case Report. Iranian Red Crescent Medical Journal. 2016;18(8).

[36] Al Mughaizwi T, Al Rawahi H, Elamin N, Al Hinai Z, Al Muharrmi Z, Al Yazidi LS. Ten-year-old Omani Girl with Lyme Arthritis. Oman medical journal. 2022;37(6):e446-e.

[37] Al Salihi KA, Younise MH, Mahmoud ZZ, Hussain T. The 2022 Crimean-Congo Hemorrhagic Fever outbreak in Iraq. Austral Journal of Veterinary Sciences. 2024;56(1):35-40.

[38] Al-Abri SS, Hewson R, Al-Kindi H, Al-Abaidani I, Al-Jardani A, Al-Maani A, et al. Clinical and molecular epidemiology of Crimean-Congo hemorrhagic fever in Oman. Plos Neglected Tropical Diseases. 2019;13(4).

[39] Al-Araimi HA, Al-Alawi K, Al-Jardani AK, Paul G, Al-Sukaiti N, Al-Farqani A, et al. Chronic Q Fever Endocarditis in an Omani Child: The First Pediatric Case Report from Oman. Oman medical journal. 2020;35(5):e180-e.

[40] Al-Kindi N, Al-Yaaqoubi M, Al-Rashdi Y, Al-Rashdi A, Al-Ajmi A, Al-Maani A. The First Confirmed Pediatric Chronic Osteomyelitis due to Coxiella Burnetii in Oman. Oman medical journal. 2022;37(6):e449-e.

[41] Al-Zadjali M, Al-Hashim H, Al-Ghilani M, Balkhiar A. A Case of Crimean-Congo Hemorrhagic Fever in Oman. Oman Medical Journal. 2013;28(3):210-2.

[42] Alabdely MH, Mukhtar N, Alshaikh A, Halim M, Mohammed S, Pragliola C, et al. Q-fever prosthetic valve endocarditis in a patient with SLE and antiphospholipid antibody syndrome. J Infect Public Health. 2020;13(5):821-3.

[43] Alam MM, Khurshid A, Sharif S, Shaukat S, Rana MS, Angez M, et al. Genetic analysis and epidemiology of Crimean Congo hemorrhagic fever viruses in Baluchistan province of Pakistan. BMC Infectious Diseases. 2013;13(1).

[44] Alavi-Naini R, Moghtaderi A, Koohpayeh H-R, Sharifi-Mood B, Naderi M, Metanat M, et al. Crimean-Congo hemorrhagic fever in Southeast of Iran. Journal of Infection. 2006;52(5):378-82.

[45] Albayrak A, Alay H, Yilmaz SI. Comorbidity of Crimean-Congo Hemorrhagic Fever and COVID-19. Rev Soc Bras Med Trop. 2021;54:e0429.

[46] Alexiou-Daniel S, Tea A, Ilonidis G, Antoniadis A. A case of pleurisy associated with antibodies to Rickettsia conorii. Clinical Microbiology and Infection. 2003;9(5):437-40.

[47] Alhetheel A, Binkhamis K, Somily A, Barry M, Shakoor Z. Screening for Q fever. A tertiary care hospital-based experience in central Saudi Arabia. Saudi Medical Journal. 2018;39(12):1195-9.

[48] Alhilfi RA, Khaleel HA, Raheem BM, Mahdi SG, Tabche C, Rawaf S. Large outbreak of Crimean-Congo haemorrhagic fever in Iraq, 2022. Ijid Regions. 2023;6:76-9.

[49] Ali S, Saeed U, Rizwan M, El-Adawy H, Mertens-Scholz K, Neubauer H. Serological Prevalence of and Risk Factors for Coxiella burnetti Infection in Women of Punjab Province, Pakistan. International Journal of Environmental Research and Public Health. 2022;19(8).

[50] Allan-Blitz L-T, Sakona A, Wallace WD, Klausner JD. Coxiella burnetii Endocarditis and Meningitis, California, USA, 2017. Emerging Infectious Diseases. 2018;24(8):1555-7.

[51] Almayahi ZK, Kindi HA, Jabri IA, Shaqsi NA, Hattali NA, Hattali AA, et al. Challenges in Diagnosis of Crimean-Congo Hemorrhagic Fever A Case Report on Patient With Self-Referral. Infectious Diseases in Clinical Practice. 2022;30(2).

[52] Almogren A, Shakoor Z, Hasanato R, Adam MH. Q fever: a neglected zoonosis in Saudi Arabia. Annals of Saudi Medicine. 2013;33(5):464-8.

[53] Aloizos S, Gourgiotis S, Oikonomou K, Stakia P. Recurrent Jarisch-Herxheimer reaction in a patient with Q fever pneumonia: a case report. Cases J. 2008;1(1):360.

[54] Altaf A, Luby S, Ahmed AJ, Zaidi N, Khan AJ, Mirza S, et al. Outbreak of Crimean-Congo haemorrhagic fever in Quetta, Pakistan: contact tracing and risk assessment. Trop Med Int Health. 1998;3(11):878-82.

[55] Altay FA, Elaldi N, Senturk GC, Altin N, Gozel MG, Albayrak Y, et al. Serum sTREM-1 Level Is Quite Higher in Crimean Congo Hemorrhagic Fever, a Viral Infection. Journal of Medical Virology. 2016;88(9):1473-8.

[56] Altin N, Altay FA, Albayrak M, Sahingoz SO, Sencan I. Serum thrombin-activatable fibrinolysis inhibitor levels and its relation with pathogenesis and bleeding and prognosis in patients with Crimean Congo hemorrhagic fever. Journal of Medical Virology. 2023;95(1).

[57] Altuntaş EE, Kaya A, Uysal I, Cevit Ö, Içağasioğlu D, Müderris S. Anterior rhinomanometry and determination of nasal mucociliary clearance time with the saccharin test in children with Crimean-Congo hemorrhagic fever. J Craniofac Surg. 2013;24(3):e239-42.

[58] Alzahrani A, Alqarni T, Alsalmi M, Ashi A, Waggass R. Q Fever Endocarditis in a Saudi Child: A Case Report and Literature Review. Cureus Journal of Medical Science. 2019;11(12).

[59] Amin S, Rahim F, Mahmood A, Gul H, Noor M, Zia A, et al. Crimean-Congo Hemorrhagic Fever Case Series: a Chronology of Biochemical and Hematological Parameters. Cureus Journal of Medical Science. 2022;14(9).

[60] Amitai Z, Bromberg M, Bernstein M, Raveh D, Keysary A, David D, et al. A Large Q Fever Outbreak in an Urban School in Central Israel. Clinical Infectious Diseases. 2010;50(11):1433-8.

[61] Ananthan D, Shah S, Haseer-Koya H, Patel A. Powasson virus causing tick-borne encephalitis: a diagnostic dilemma. Qjm-an International Journal of Medicine. 2014;107(11):909-10.

[62] Anderson AD, Baker TR, Littrell AC, Mott RL, Niebuhr DW, Smoak BL. Seroepidemiologic survey for Coxiella burnetii among hospitalized US troops deployed to Iraq. Zoonoses and Public Health. 2011;58(4):276-83.

[63] Anderson AD, Kruszon-Moran D, Loftis AD, McQuillan G, Nicholson WL, Priestley RA, et al. Seroprevalence of Q Fever in the United States, 2003-2004. American Journal of Tropical Medicine and Hygiene. 2009;81(4):691-4.

[64] Anderson JF, Armstrong PM. Seroepidemiologic survey for Coxiella burnetii among hospitalized US troops deployed to Iraq. American Journal of Tropical Medicine and Hygiene. 2012;87(4):754-

9.
[65] Angelakis E, Mediannikov O, Socolovschi C, Mouffok N, Bassene H, Tall A, et al. Coxiella burnetii-positive PCR in febrile patients in rural and urban Africa. International Journal of Infectious Diseases. 2014;28:107-10.
[66] Angelakis E, Million M, D'Amato F, Rouli L, Richet H, Stein A, et al. Q fever and pregnancy: disease, prevention, and strain specificity. European Journal of Clinical Microbiology & Infectious Diseases. 2013;32(3):361-8.
[67] Angelakis E, Thiberville S-D, Million M, Raoult D. Sternoclavicular joint infection caused by Coxiella burnetii: a case report. Journal of medical case reports. 2016;10(1):139-.
[68] Annen K, Friedman K, Eshoa C, Horowitz M, Gottschall J, Straus T. Two cases of transfusion-transmitted Anaplasma phagocytophilum. American Journal of Clinical Pathology. 2012;137(4):562-5.
[69] Antoniou M, Economou I, Wang XY, Psaroulaki A, Spyridaki I, Papadopoulos B, et al. Fourteen-year seroepidemiological study of zoonoses in a Greek village. American Journal of Tropical Medicine and Hygiene. 2002;66(1):80-5.
[70] Antony SJ, Dummer JS, Hunter E. Human Ehrlichiosis in a Liver Transplant Recipient. Transplantation. 1995;60(8):879-80.
[71] Aradaib IE, Erickson BR, Karsany MS, Khristova ML, Elageb RM, Mohamed MEH, et al. Multiple Crimean-Congo Hemorrhagic Fever Virus Strains Are Associated with Disease Outbreaks in Sudan, 2008-2009. Plos Neglected Tropical Diseases. 2011;5(5).
[72] Aradaib IE, Erickson BR, Mustafa ME, Khristova ML, Saeed NS, Elageb RM, et al. Nosocomial Outbreak of Crimean-Congo Hemorrhagic Fever, Sudan. Emerging Infectious Diseases. 2010;16(5):837-9.
[73] Arav-Boger R, Knepp JH, Walls JJ, Dumler SJ. Human Monocytic Ehrlichiosis in a Child with Leukemia. The Pediatric Infectious Disease Journal. 2000;19(2):173-5.
[74] Ardalan MR, Tubbs RS, Chinikar S, Shoja MM. Crimean-Congo haemorrhagic fever presenting as thrombotic microangiopathy and acute renal failure. Nephrology Dialysis Transplantation. 2006;21(8):2304-7.
[75] Argov O, Weintraub M, Charach G. Doughnut granulomas from erythema nodosum in acute Q fever. Israel Medical Association Journal. 2008;10(3):241-2.
[76] Arslan M, Yilmaz G, Mentese A, Yilmaz H, Karahan SC, Koksal I. Importance of endothelial dysfunction biomarkers in patients with Crimean-Congo hemorrhagic fever. Journal of Medical Virology. 2017;89(12):2084-91.
[77] Aslani D, Salehi-Vaziri M, Baniasadi V, Jalali T, Azad-Manjiri S, Mohammadi T, et al. Crimean-Congo hemorrhagic fever among children in Iran. Archives of Virology. 2017;162(3):721-5.
[78] Assi MA, Yao JDC, Walker RC. Lyme disease followed by human granulocytic anaplasmosis in a kidney transplant recipient. Transplant Infectious Disease. 2007;9(1):66-72.
[79] Athanassopoulou P, Liatsos G, Pirounaki M, Skounakis M, Moulakakis A. Disseminated intravascular coagulation as the laboratory hallmark of acute Q fever. Diagnostic Microbiology and Infectious Disease. 2011;69(2):210-2.
[80] Atlas E, Novak SN, Duray PH, Steere AC. Lyme Myositis: Muscle Invasion by Borrelia burgdorferi. Annals of Internal Medicine. 1988;109(3):245-6.
[81] Atwan Z, Alhilfi R, Mousa AK, Rawaf S, Torre JDL, Hashim A, et al. Alarming update on

incidence of Crimean-Congo hemorrhagic fever in Iraq in 2023. Ijid Regions. 2024;10:75-9.
[82] Aubin A, Eldin C, Zemali N, Jaubert J, Koumar Y, Moiton M-P, et al. Clinical and Epidemiological Aspects of Acute Q Fever in Reunion Island over Fourteen Years: A Retrospective Cohort Study. Microorganisms. 2023;11(10).
[83] Avitabile CM, Harris MA, Chowdhury D. Cardiac Magnetic Resonance Characterizes Myocarditis in a 16-Year-Old Female With Lyme Disease. World J Pediatr Congenit Heart Surg. 2016;7(3):394-6.
[84] Ayatollahi J, Shahcheraghi SH, Mirjalili M. Report of nine cases of Crimean-Congo haemorrhagic fever From Iran. Nigerian medical journal : journal of the Nigeria Medical Association. 2015;56(2):156-9.
[85] Aydin N, Icagasioglu DF. Longitudinal Extensive Transverse Myelitis Secondary to Lyme Disease. Bezmialem Science. 2022;10(5):652-4.
[86] Aydin ZGG, Tanir G, Metin O, Teke TA, Bayhan GI, Oz FN, et al. Transient sinus bradycardia during the course of Crimean-Congo hemorrhagic fever in children. Ticks and Tick-Borne Diseases. 2015;6(2):185-8.
[87] Azouzi F, Olagne L, Edouard S, Cammilleri S, Magnan P-E, Fournier P-E, et al. Coxiella burnetii Femoro-Popliteal Bypass Infection: A Case Report. Microorganisms. 2023;11(9).
[88] Bababeygy SR, Quiros PA. Isolated trochlear palsy secondary to Lyme neuroborreliosis. Int Ophthalmol. 2011;31(6):493-5.
[89] Babu K, Murthy KR, Bhagya M, Murthy PR, Puttamallesh VN, Ravi V. Seroprevalence of Lymes disease in the Nagarahole and Bandipur forest areas of South India. Indian Journal of Ophthalmology. 2020;68(1):100-3.
[90] Backliwal G, Hildinger M, Chenuet S, DeJesus M, Wurm FM. Coexpression of acidic fibroblast growth factor enhances specific productivity and antibody titers in transiently transfected HEK293 cells. New Biotechnology. 2008;25(2-3):162-6.
[91] Badarni K, Blich M, Atiya-Nasagi Y, Ghanem-Zoabi N. Acute Q Fever with Atrioventricular Block, Israel. Emerging Infectious Diseases. 2022;28(9):1886-9.
[92] Baker A, Wang H-H, Mogg M, Derouen Z, Borski J, Grant WE. Increasing Incidence of Anaplasmosis in the United States, 2012 Through 2016. Vector-Borne and Zoonotic Diseases. 2020;20(11):855-9.
[93] Bakir M, Bakir S, Sari I, Celik VK, Gozel MG, Engin A. Evaluation of the relationship between serum levels of VEGF and sVEGFR1 with mortality and prognosis in patients with Crimean-Congo hemorrhagic fever. J Med Virol. 2013;85(10):1794-801.
[94] Bakir M, Engin A, Gozel MG, Kilickap S, Cinar Z. A new perspective to determine the severity of cases with Crimean-Congo hemorrhagic fever. Journal of Vector Borne Diseases. 2012;49(2):105-10.
[95] Bakir M, Engin A, Kuskucu MA, Bakir S, Gundag O, Midilli K. Relationship of plasma cell-free DNA level with mortality and prognosis in patients with Crimean-Congo hemorrhagic fever. Journal of Medical Virology. 2016;88(7):1152-8.
[96] Bakir M, Ugurlu M, Dokuzoguz B, Bodur H, Tasyaran MA, Vahaboglu H, et al. Crimean-Congo haemorrhagic fever outbreak in Middle Anatolia: a multicentre study of clinical features and outcome measures. Journal of Medical Microbiology. 2005;54(4):385-9.
[97] Balakrishnan N, Menon T, Fournier P-E, Raoult D. Bartonella quintanaandCoxiella burnetiias

Causes of Endocarditis, India. Emerging Infectious Diseases. 2008;14(7):1168-9.
[98] Balasundaram MB, Manjunath M, Baliga G, Kapadi F. Ocular manifestations of Rickettsia conorii in South India. Indian Journal of Ophthalmology. 2018;66(12):1840-+.
[99] Baltadzhiev I, Kevorkyan A, Popivanova N. Mediterranean spotted fever in child and adult patients: investigation from an endemic region in Bulgaria. Central European Journal of Public Health. 2020;28(3):187-92.
[100] Baltadzhiev I, Zaprianov Z, Baltadjiev A. Renal Involvement in Mediterranean Spotted Fever: Clinical and Histopathological Data. Med Princ Pract. 2021;30(4):369-75.
[101] Baltadzhiev IG, Popivanova NI. Some epidemiological features of the mediterranean spotted fever re-emerging in Bulgaria. Folia Medica. 2012;54(1):36-43.
[102] Bariola JR, Bradsher RW. Severe Ehrlichia chaffeensis Infection in a Patient Receiving Etanercept, a Tumor Necrosis Factor Inhibitor. Infectious Diseases in Clinical Practice. 2008;16(2):124-6.
[103] Barton LL, Dawson JE, Letson GW, Luisiri A, Scalzo AJ. Simultaneous ehrlichiosis and Lyme disease. The Pediatric Infectious Disease Journal. 1990;9(2):127-8.
[104] Baseri N, Salehi-Vaziri M, Mostafavi E, Amiri FB, Latifian M, Stenos J, et al. Investigation of Rickettsia conorii in Patients Suspected of Having Crimean-Congo Hemorrhagic Fever. Pathogens. 2022;11(9).
[105] Bayard-Mc Neeley M, Bansal A, Chowdhury I, Girao G, Small CB, Seiter K, et al. In vivo and in vitro studies on Anaplasma phagocytophilum infection of the myeloid cells of a patient with chronic myelogenous leukaemia and human granulocytic ehrlichiosis. Journal of Clinical Pathology. 2004;57(5):499-503.
[106] Baymakova M, Pekova L, Plochev K, Parousheva P. Severe clinical forms of Mediterranean Spotted Fever: A case series from an endemic area in Bulgaria. International Journal of Infectious Diseases. 2016;53:150-1.
[107] Baymakova M, Popov GT, Andonova R, Kovaleva V, DiKov I, Plochev K. Fever of unknown origin and Q-fever: a case series in a Bulgarian hospital. Caspian Journal of Internal Medicine. 2019;10(1):102-6.
[108] Bayram Y, Parlak M, Ozkacmaz A, Cikman A, Guducuoglu H, Kilic S, et al. Seroprevalence of Crimean-Congo Hemorrhagic Fever in Turkey's Van Province. Japanese Journal of Infectious Diseases. 2017;70(1):65-8.
[109] Bazer DA, Orwitz M, Koroneos N, Syritsyna O, Wirkowski E. Powassan Encephalitis: A Case Report from New York, USA. Case Reports in Neurological Medicine. 2022;2022.
[110] Beauté J, Spiteri G, Warns-Petit E, Zeller H. Tick-borne encephalitis in Europe, 2012 to 2016. Euro Surveill. 2018;23(45).
[111] Behera SP, Singh R, Deval H, Bhardwaj P, Zaman K, Misra BR, et al. Molecular detection of spotted fever group of Rickettsiae in acute encephalitis syndrome cases from eastern Uttar Pradesh region of India. Zoonoses and Public Health. 2023;70(5):403-10.
[112] Belet N, Top A, Terzi O, Arslan HN, Baysal K, Sensoy G. Evaluation of Children with Crimean-Congo Hemorrhagic Fever in the Central Blacksea Region. Pediatric Infectious Disease Journal. 2014;33(8):E194-E7.
[113] Belman AL. Cerebrospinal Fluid Findings in Children With Lyme Disease-Associated Facial Nerve Palsy. Arch Pediatr Adolesc Med. 1997;151(12).

[114] Belman AL, Coyle PK, Roque C, Cantos E. MRI findings in children infected by Borrelia burgdorferi. Pediatric Neurology. 1992;8(6):428-31.

[115] Belongia EA, Reed KD, Mitchell PD, Chyou PH, Mueller-Rizner N, Finkel MF, et al. Clinical and Epidemiological Features of Early Lyme Disease and Human Granulocytic Ehrlichiosis in Wisconsin. Clinical Infectious Diseases. 1999;29(6):1472-7.

[116] Benabdellah A, Mouffok N, Bensaad M, Kouied AB, Razik F, Raoult D. Mediterranean-spotted fever: clinical and laboratory characteristics of 34 children in Oran (Algeria). Pathologie Biologie. 2007;55(10):539-42.

[117] Benach JL, Coleman JL, Habicht GS, MacDonald A, Grunwaldt E, Giron JA. Serological Evidence for Simultaneous Occurrences of Lyme Disease and Babesiosis. Journal of Infectious Diseases. 1985;152(3):473-7.

[118] Benslimani A, Fenollar F, Lepidi H, Raoult D. Bacterial Zoonoses and Infective Endocarditis, Algeria. Emerging Infectious Diseases. 2005;11(2):216-24.

[119] Bentov Y, Sheiner E, Kenigsberg S, Mazor M. Mediterranean spotted fever during pregnancy: case presentation and literature review. European Journal of Obstetrics & Gynecology and Reproductive Biology. 2003;107(2):214-6.

[120] Berger BW, Johnson RG. Clinical and microbiologic findings in six patients with erythema migrans of Lyme disease. Journal of the American Academy of Dermatology. 1989;21(6):1188-91.

[121] Bernard E, Carles M, Laffont C, Durant J, Dellamonica P. Guillain-Barré syndrome associated with acute Q fever. European Journal of Clinical Microbiology & Infectious Diseases. 1994;13(8):658-9.

[122] Bernit E, Pouget J, Janbon F, Dutronc H, Martinez P, Brouqui P, et al. Neurological Involvement in Acute Q Fever. Archives of Internal Medicine. 2002;162(6).

[123] Beştepe Dursun Z, Korkmaz S, Türe Z, Kaynar L, Dursun A, Çelik İ. Efficacy of therapeutic plasma exchange in patients with Crimean-Congo hemorrhagic fever. J Clin Apher. 2021;36(3):390-7.

[124] Bilgin G, Ataman Hatipoglu C, Altun S, Bulut C, Kinikli S, Demiroz AP. An investigation of pulmonary findings of Crimean-Congo haemorrhagic fever patients. Turkish Journal of Medical Sciences. 2014;44(1):162-7.

[125] Binder WD, Gupta R. African Tick-Bite Fever in a Returning Traveler. Journal of Emergency Medicine. 2015;48(5):562-5.

[126] Biswal M, Zaman K, Suri V, Gopi S, Kumar A, Gopi T, et al. Molecular confirmation & characterization of Rickettsia conorii in north India: A report of three cases. Indian Journal of Medical Research. 2020;151(1):59-64.

[127] Bitsori M, Galanakis E, Papadakis CE, Sbyrakis S. Facial nerve palsy associated with Rickettsia conorii infection. Archives of Disease in Childhood. 2001;85(1):54-5.

[128] Bitsori M, Vergadi E, Germanakis I, Raissaki M, Galanakis E. Case Report: A Case of Endocarditis and Embolic Stroke in a Child, Suggestive of Acute Q Fever Infection. American Journal of Tropical Medicine and Hygiene. 2020;103(4):1435-8.

[129] Bjork A, Marsden-Haug N, Nett RJ, Kersh GJ, Nicholson W, Gibson D, et al. First Reported Multistate Human Q Fever Outbreak in the United States, 2011. Vector-Borne and Zoonotic Diseases. 2014;14(2):111-7.

[130] Bodur H, Akinci E, Ascioglu S, Onguru P, Uyar Y. Subclinical Infections with Crimean-Congo Hemorrhagic Fever Virus, Turkey. Emerging Infectious Diseases. 2012;18(4):640-2.

[131] Bodur H, Akinci E, Onguru P, Carhan A, Uyar Y, Tanrici A, et al. Detection of Crimean-Congo hemorrhagic fever virus genome in saliva and urine. International Journal of Infectious Diseases. 2010;14(3):E247-E9.

[132] Bodur H, Akinci E, Onguru P, Uyar Y, Basturk B, Gozel MG, et al. Evidence of vascular endothelial damage in Crimean-Congo hemorrhagic fever. International Journal of Infectious Diseases. 2010;14(8):E704-E7.

[133] Bolling T, Ritter AS, Gupte AA. An Unusual Case of Ehrlichiosis Manifesting With Hyponatremia, Acute Encephalopathy, and Hemophagocytic Lymphohistiocytosis. Cureus. 2022;14(7):e26943.

[134] Bonifay T, Beillard E, Daniel M, Schiemsky V, Vierendeels E, Demar M, et al. High incidence of acute Q fever among incarcerated people in Cayenne, French Guiana. Revista Do Instituto De Medicina Tropical De Sao Paulo. 2022;64.

[135] Botelho-Nevers E, Fournier PE, Richet H, Fenollar F, Lepidi H, Foucault C, et al. Coxiella burnetii infection of aortic aneurysms or vascular grafts: report of 30 new cases and evaluation of outcome. European Journal of Clinical Microbiology & Infectious Diseases. 2007;26(9):635-40.

[136] Botelho-Nevers E, Gagneux-Brunon A, Velay A, Guerbois-Galla M, Grard G, Bretagne C, et al. Tick-Borne Encephalitis in Auvergne-Rhone-Alpes Region, France, 2017-2018. Emerging Infectious Diseases. 2019;25(10):1944-8.

[137] Bouchaib H, Amrane A, Sevestre J, Bitam I, Parola P. Mediterranean spotted fever and peripheral facial nerve palsy: a rare neurological complication. Int J Infect Dis. 2022;117:15-7.

[138] Boudebouch N, Sarih M, Socolovschi C, Fatihi T, Chakib A, Amarouch H, et al. Spotted fever group rickettsioses documented in Morocco. Clinical Microbiology and Infection. 2009;15:257-8.

[139] Bower H, El Karsany M, Alzain M, Gannon B, Mohamed R, Mahmoud I, et al. Detection of Crimean-Congo Haemorrhagic Fever cases in a severe undifferentiated febrile illness outbreak in the Federal Republic of Sudan: A retrospective epidemiological and diagnostic cohort study. Plos Neglected Tropical Diseases. 2019;13(7).

[140] Breton G, Yahiaoui Y, Deforges L, Lebrun A, Michel M, Godeau B. Psoas abscess: An unusual manifestation of Q fever. Eur J Intern Med. 2007;18(1):66-8.

[141] Brett M, Doppalapudi A, Respicio-Kingry LB, Myers D, Husband B, Pollard K, et al. Francisella novicida bacteremia after a near-drowning accident. Journal of Clinical Microbiology. 2012;50(8):2826-9.

[142] Brett ME, Respicio-Kingry LB, Yendell S, Ratard R, Hand J, Balsamo G, et al. Outbreak of Francisella novicida bacteremia among inmates at a louisiana correctional facility. Clinical Infectious Diseases. 2014;59(6):826-33.

[143] Briggs B. Tick-Borne Encephalitis Virus, Kyrgyzstan. Emerging Infectious Diseases. 2011:876-9.

[144] Brouqui P. Chronic Q fever. Ninety-two cases from France, including 27 cases without endocarditis. Archives of Internal Medicine. 1993;153(5):642-8.

[145] Brouqui P, Badiaga S, Raoult D. Q Fever Outbreak in Homeless Shelter. Emerging Infectious Diseases. 2004;10(7):1297-9.

[146] Brouqui P, Dumler JS, Raoult D. Immunohistologic demonstration of coxiella burnetii in the valves of patients with Q fever endocarditis. The American Journal of Medicine. 1994;97(5):451-8.

[147] Brouqui P, Dupont HT, Drancourt M, Bourgeade A, Raoult D. Spotless Boutonneuse Fever. Clinical Infectious Diseases. 1992;14(1):114-6.

[148] Brummitt SI, Kjemtrup AM, Harvey DJ, Petersen JM, Sexton C, Replogle A, et al. Borrelia burgdorferi and Borrelia miyamotoi seroprevalence in California blood donors. PLoS ONE. 2020;15(12).

[149] Bucak O, Kocoglu ME, Tas T, Mengeloglu FZ. Evaluation of Borrelia burgdorferi sensu lato seroprevalencein the province of Bolu, Turkey. Turkish Journal of Medical Sciences. 2016;46(3):727-32.

[150] Bucher B, Poupard J, Parra J-P, Vernant J-C, Buisson G, Koproswki H, et al. Borrelia burgdorferi and tropical spastic paraparesis. The Lancet. 1990;336(8723).

[151] Buitrago Martha I, Ijdo Jacob W, Rinaudo P, Simon H, Copel J, Gadbaw J, et al. Human Granulocytic Ehrlichiosis During Pregnancy Treated Successfully with Rifampin. Clinical Infectious Diseases. 1998;27(1):213-5.

[152] Bush V, Chaudhary J, Manu D, Hyman C. A rare occurrence of Anaplasma-associated peritonitis. Laboratory Medicine. 2023;54(5):E152-E6.

[153] Buzzard SL, Bissell BD, Thompson Bastin ML. Ehrlichiosis presenting as severe sepsis and meningoencephalitis in an immunocompetent adult. JMM case reports. 2018;5(9):e005162-e.

[154] Cagatay A, Kapmaz M, Karadeniz A, Basaran S, Yenerel M, Yavuz S, et al. Haemophagocytosis in a patient with Crimean-Congo haemorrhagic fever. Journal of Medical Microbiology. 2007;56(8):1126-8.

[155] Cai X, Li Y, Wu Z. Rapid Detection of Acute Respiratory Virus and Atypical Bacteria Infections in Children. Jundishapur Journal of Microbiology. 2013;6(5).

[156] Camacci ML, Panganiban RP, Pattison Z, Haghayeghi K, Daly A, Ojevwe C, et al. Severe Human Granulocytic Anaplasmosis With Significantly Elevated Ferritin Levels in an Immunocompetent Host in Pennsylvania: A Case Report. J Investig Med High Impact Case Rep. 2018;6:2324709618758350.

[157] Carcopino X, Raoult D, Bretelle F, Boubli L, Stein A. Managing Q fever during pregnancy: The benefits of long-term cotrimoxazole therapy. Clinical Infectious Diseases. 2007;45(5):548-55.

[158] Carpenter Christopher F, Gandhi Tejal K, Kong Li K, Corey GR, Chen SM, Walker David H, et al. The Incidence of Ehrlichial and Rickettsial Infection in Patients with Unexplained Fever and Recent History of Tick Bite in Central North Carolina. The Journal of Infectious Diseases. 1999;180(3):900-3.

[159] Carrieri MP, Tissot-Dupont H, Rey D, Brousse P, Renard H, Obadia Y, et al. Investigation of a slaughterhouse-related outbreak of Q fever in the French Alps. European Journal of Clinical Microbiology & Infectious Diseases. 2002;21(1):17-21.

[160] Carter N, Miller NR. Fourth Nerve Palsy Caused by Ehrlichia chaffeensis. Journal of Neuro-Ophthalmology. 1997;17(1).

[161] Carvounis PE, Mehta AP, Geist CE. Orbital myositis associated with Borrelia burgdorferi (Lyme disease) infection. Ophthalmology. 2004;111(5):1023-8.

[162] Casalta JP, Gouriet F, Richet H, Thuny F, Habib G, Raoult D. Prevalence of Coxiella burnetii and

Bartonella species as cases of infective endocarditis in Marseilles (1994-2007). Clinical Microbiology and Infection. 2009;15:152-3.

[163] Cassarino DS, Quezado MM, Ghatak NR, Duray PH. Lyme-Associated Parkinsonism: A Neuropathologic Case Study and Review of the Literature. Arch Pathol Lab Med. 2003;127(9):1204-6.

[164] Celik VK, Sari I, Engin A, Gürsel Y, Aydin H, Bakir S. Determination of serum adenosine deaminase and xanthine oxidase levels in patients with crimean-congo hemorrhagic fever. Clinics (Sao Paulo). 2010;65(7):697-702.

[165] Celikbaş A, Ergönül O, Dokuzoğuz B, Eren S, Baykam N, Polat-Düzgün A. Crimean Congo hemorrhagic fever infection simulating acute appendicitis. J Infect. 2005;50(4):363-5.

[166] Celikbas AK, Dokuzoguz B, Baykam N, Gok SE, Eroglu MN, Midilli K, et al. Crimean-Congo Hemorrhagic Fever among Health Care Workers, Turkey. Emerging Infectious Diseases. 2014;20(3):477-9.

[167] Çetinkaya B, Kalender H, Ertas HB, Muz A, Arslan N, Ongor H, et al. Seroprevalence of coxiellosis in cattle, sheep and people in the east of Turkey. Veterinary Record. 2000;146(5):131-6.

[168] Chao LL, Chen YJ, Shih CM. First isolation and molecular identification of Borrelia burgdorferi sensu stricto and Borrelia afzelii from skin biopsies of patients in Taiwan. Int J Infect Dis. 2011;15(3):e182-7.

[169] Chaumentin G, Zénone T, Bibollet C, Denoyel GA, Boibieux A, Biron F, et al. Malignant boutonneuse fever and polymyalgia rheumatica: A coincidental association? Infection. 1997;25(5):320-2.

[170] Chaves TdSS, Mascheretti M, Alves JR, Boulos M, Lopes MH. Travel medicine in the state of São Paulo, Brazil. Travel Medicine and Infectious Disease. 2012;10(5-6):283-4.

[171] Chen R, Kou Z, Xu L, Cao J, Liu Z, Wen X, et al. Analysis of epidemiological characteristics of four natural-focal diseases in Shandong Province, China in 2009-2017: A descriptive analysis. PLoS ONE. 2019;14(8).

[172] Chengxu AI, Yuxin WEN, Yongguo Z, Shaoshan W, Quicheng QIU, Zhixue SHI, et al. Clinical Manifestations and Epidemiological Characteristics of Lyme Disease in Hailin County, Heilongjiang Province, China. Annals of the New York Academy of Sciences. 2006;539(1):302-13.

[173] Chenouard R, Hoppé E, Lemarié C, Talha A, Ducellier F, Ferchaud F, et al. A rare case of Prosthetic Joint Infection associated with Coxiella burnetii. Int J Infect Dis. 2019;87:166-9.

[174] Cherry CC, Nichols Heitman K, Bestul NC, Kersh GJ. Acute and chronic Q fever national surveillance - United States, 2008-2017. Zoonoses Public Health. 2022;69(2):73-82.

[175] Chinikar S, Ghiasi SM, Moradi M, Goya MM, Reza Shirzadi M, Zeinali M, et al. Phylogenetic analysis in a recent controlled outbreak of Crimean-Congo haemorrhagic fever in the south of Iran, December 2008. Eurosurveillance. 2010;15(47).

[176] Chinikar S, Ghiasi SM, Moradi M, Goya MM, Shirzadi MR, Zeinali M, et al. Geographical Distribution and Surveillance of Crimean-Congo Hemorrhagic Fever in Iran. Vector-Borne and Zoonotic Diseases. 2010;10(7):705-8.

[177] Chinikar S, Ghiasi SM, Naddaf S, Piazak N, Moradi M, Razavi MR, et al. Serological Evaluation of Crimean-Congo Hemorrhagic Fever in Humans with High-Risk Professions Living in Enzootic

Regions of Isfahan Province of Iran and Genetic Analysis of Circulating Strains. Vector-Borne and Zoonotic Diseases. 2012;12(9):733-8.
[178] Chinikar S, Shayesteh M, Khakifirouz S, Jalali T, Varaie FSR, Rafigh M, et al. Nosocomial infection of Crimean-Congo haemorrhagic fever in eastern Iran: Case report. Travel Medicine and Infectious Disease. 2013;11(4):252-5.
[179] Chochlakis D, Papaeustathiou A, Minadakis G, Psaroulaki A, Tselentis Y. A serosurvey of Anaplasma phagocytophilum in blood donors in Crete, Greece. European Journal of Clinical Microbiology & Infectious Diseases. 2008;27(6):473-5.
[180] Chochlakis D, Psaroulaki A, Kokkini S, Kostanatis S, Arkalati E, Karagrannaki E, et al. First evidence of Anaplasma infection in Crete, Greece. Report of six human cases. Clinical Microbiology and Infection. 2009;15:8-9.
[181] Choi EEJ, Taylor RA. A case of Powassan viral hemorrhagic encephalitis involving bilateral thalami. Clinical Neurology and Neurosurgery. 2012;114(2):172-5.
[182] Chowdri HR, Gugliotta JL, Berardi VP, Goethert HK, Molloy PJ, Sterling SL, et al. Borrelia miyamotoi infection presenting as human granulocytic anaplasmosis: a case report. Annals of Internal Medicine. 2013;159(1):21-+.
[183] Christenson M, Lee X, Larson S, Johnson DH, Jensen J, Meller M, et al. Occurrence of Amblyomma americanum (Acari: Ixodidae) and Human Infection With Ehrlichia chaffeensis in Wisconsin, 2008-2015. Journal of Medical Entomology. 2017;54(3):752-6.
[184] Christova I, Panayotova E, Tchakarova S, Taseva E, Trifonova I, Gladnishka T. A nationwide seroprevalence screening for West Nile virus and Tick-borne encephalitis virus in the population of Bulgaria. Journal of Medical Virology. 2017;89(10):1875-8.
[185] Christova I, Younan R, Taseva E, Gladnishka T, Trifonova I, Ivanova V, et al. Hemorrhagic Fever with Renal Syndrome and Crimean-Congo Hemorrhagic Fever as Causes of Acute Undifferentiated Febrile Illness in Bulgaria. Vector-Borne and Zoonotic Diseases. 2013;13(3):188-92.
[186] Cikman A, Aydin M, Gulhan B, Karakecili F, Demirtas L. Geographical Features and Seroprevalence of Borrelia burgdorferi in Erzincan, Turkey. Journal of Arthropod-borne Diseases. 2018;12(4):378-86.
[187] Cikman A, Aydin M, Gulhan B, Karakecili F, Kesik OA, Ozcicek A, et al. Seroprevalence of Crimean-Congo Hemorrhagic Fever Virus in Erzincan Province, Turkey, Relationship with Geographic Features and Risk Factors. Vector-Borne and Zoonotic Diseases. 2016;16(3):199-204.
[188] Cikman A, Aydin M, Gulhan B, Karakecili F, Ozcicek A, Kesik OA, et al. The seroprevalence of Coxiella burnetii in Erzincan, Turkey: Identification of the risk factors and their relationship with geographical features. Journal of Vector Borne Diseases. 2017;54(2):157-63.
[189] Citil R, Egri M, onder Y, Duygu F, Bulut YE, Yasayancan O, et al. Determination of Seroprevalence and Risk Factors of Crimean-Congo Haemorrhagic Fever (CCHF) in the Endemic Region in Turkey: A Population-Based Cross-Sectional Study. Journal of Tropical Medicine. 2021;2021.
[190] Clark KLL, Herman-Giddens MEE. Investigation of a Symptomatic Tick Bite Patient Confirms Borrelia burgdorferi in Ixodes scapularis and White-Footed Mice in Ashe County, North Carolina. Vector-Borne and Zoonotic Diseases. 2023;23(2):81-4.
[191] Cohen J, Lasri Y, Land Z. Mediterranean Spotted Fever in Pregnancy. Scandinavian Journal of

Infectious Diseases. 2009;31(2):202-3.
[192] Cohen R, Finn T, Babushkin F, Paran Y, Ben Ami R, Atamna A, et al. Spotted Fever Group Rickettsioses in Israel, 2010-2019. Emerging Infectious Diseases. 2021;27(8):2117-26.
[193] Comer JA, Nicholson WL, Olson JG, Childs JE. Serologic Testing for Human Granulocytic Ehrlichiosis at a National Referral Center. Journal of Clinical Microbiology. 1999;37(3):558-64.
[194] Comer JA, Nicholson WL, Sumner JW, Olson JG, Childs JE. Diagnosis of Human Ehrlichiosis by PCR Assay of Acute-Phase Serum. Journal of Clinical Microbiology. 1999;37(1):31-4.
[195] Cosgun Y, Aydemir A, Hedef H, Kamiloglu AO, Klemens O, Lattwein E, et al. Evaluation of Nucleoprotein-Based Enzyme-Linked Immunosorbent Assay for Serodiagnosis of Acute Crimean-Congo Hemorrhagic Fever Virus Infections in a Turkish Population. Vector-Borne and Zoonotic Diseases. 2023;23(1):44-53.
[196] Cosiquien RJS, Stojiljkovic N, Nordstrom CW, Amadi E, Lutwick L, Dumic I. Anaplasma phagocytophilum Encephalitis: A Case Report and Literature Review of Neurologic Manifestations of Anaplasmosis. Infectious Disease Reports. 2023;15(4):354-9.
[197] Coste Mazeau P, Hantz S, Eyraud JL, Donadel L, Lacorre A, Rogez S, et al. Q fever and pregnancy: experience from the Limoges Regional University Hospital. Arch Gynecol Obstet. 2016;294(2):233-8.
[198] Cove-Smith A, Klein JL. Gonococcal endocarditis: forgotten but not quite gone. Scandinavian Journal of Infectious Diseases. 2009;38(8):696-7.
[199] Cunha BA, Chandrankunnel JG, Hage JE. Ehrlichia chaffeensis human monocytic ehrlichiosis with pancytopenia. Scandinavian Journal of Infectious Diseases. 2012;44(1/12):473-4.
[200] Cunha BA, Petelin A, Hage JE. Ehrlichia chaffeensis presenting with bilateral anterior thigh pain (Louria's sign). Travel Med Infect Dis. 2012;10(5-6):267-9.
[201] D'Angelo LJ, Baker EF, Schlosser W. Q Fever in the United States, 1948-1977. Journal of Infectious Diseases. 1979;139(5):613-5.
[202] Dabaja MF, Greco G, Villari S, Bayan A, Vesco G, Gargano V, et al. The First Serological Study of Q Fever in Humans in Lebanon. Vector-Borne and Zoonotic Diseases. 2018;18(3):138-43.
[203] Dąbrowski JM, Urbanska K, Arnaut LG, Pereira MM, Abreu AR, Simões S, et al. Biodistribution and Photodynamic Efficacy of a Water-Soluble, Stable, Halogenated Bacteriochlorin against Melanoma. Chemmedchem. 2011;6(3):465-75.
[204] Dahlgren FS, Mandel EJ, Krebs JW, Massung RF, McQuiston JH. Increasing incidence of Ehrlichia chaffeensis and Anaplasma phagocytophilum in the United States, 2000-2007. American Journal of Tropical Medicine and Hygiene. 2011;85(1):124-31.
[205] Dahlgren FS, McQuiston JH, Massung RF, Anderson AD. Q Fever in the United States: Summary of Case Reports from Two National Surveillance Systems, 2000-2012. American Journal of Tropical Medicine and Hygiene. 2015;92(2):247-55.
[206] Dandashi JA, Nizamutdinov D. Texas Occurrence of Lyme Disease and Its Neurological Manifestations. Journal of Neuroinfectious Diseases. 2016;7(2).
[207] Daniel SA, Manika K, Arvanitidou M, Diza E, Symeonidis N, Antoniadis A. Serologic Evidence of Human Granulocytic Ehrlichiosis, Greece. Emerging Infectious Diseases. 2002;8(6):643-4.
[208] Darwish MA, Hoogstraal H, Roberts TJ, Ghazi R, Amer T. A sero-epidemiological survey for Bunyaviridae and certain other arboviruses in Pakistan. Transactions of the Royal Society of Tropical Medicine and Hygiene. 1983;77(4):446-50.

[209] Das S, Ninan GA, Jasper S, George M, Iyadurai R. Spotted fever rickettsiosis presenting with bilateral anterior uveitis and retinitis: A case report. Journal of Family Medicine and Primary Care. 2020;9(2):1236-9.

[210] De Martino SJ, Carlyon JA, Fikrig E. Coinfection withBorrelia burgdorferiand the Agent of Human Granulocytic Ehrlichiosis. New England Journal of Medicine. 2001;345(2):150-1.

[211] De Micco C, Raoult D, Benderitter T, Gallais H, Toga M. Immune complex vasculitis associated with mediterranean spotted fever. Journal of Infection. 1987;14(2):163-5.

[212] Defer G, Levy R, Brugiéres P, Postic D, Degos JD. Lyme disease presenting as a stroke in the vertebrobasilar territory: MRI. Neuroradiology. 1993;35(7):529-31.

[213] Deibel R, Osterhout G, Culver J. Immune Globulins to Coxiella Burneti in Man Determined by Radiolsotope Precipitation Technic1. American Journal of Epidemiology. 1969;90(3):262-8.

[214] des Vaux CdLP, Sainte-Rose V, Le Turnier P, Djossou F, Nacher M, Zappa M, et al. Chest CT findings in community-acquired pneumonia due to Coxiella burnetii (Q fever) compared to Streptococcus pneumoniae, a cross sectional study in French Guiana, 2013-2017. Travel Medicine and Infectious Disease. 2024;57.

[215] Desai R, Esposito DH, Lees C, Goodin K, Harris M, Blostein J, et al. Rotavirus-coded Deaths in Children, United States, 1999–2007. Pediatric Infectious Disease Journal. 2011;30(11):986-8.

[216] Deveci K, ouml, ksal, Uysal EB, Kaya A, Sancakdar E, et al. Evaluation of Renal Involvement in Children with Crimean-Congo Hemorrhagic Fever. Japanese Journal of Infectious Diseases. 2013;66(6):493-6.

[217] Dhand A, Nadelman RB, Aguero-Rosenfeld M, Haddad FA, Stokes DP, Horowitz HW. Human granulocytic anaplasmosis during pregnancy: case series and literature review. Clin Infect Dis. 2007;45(5):589-93.

[218] Didona D, Cunha T, Viti G, Juratli HA, Hertl M. Concidence of cotrimoxazole induced toxic epidermal necrolysis and chronic Q Fever in an Afghan shepherd. Journal of the European Academy of Dermatology and Venereology. 2022;36(11):E939-E40.

[219] Dilber E, Cakir M, Acar EA, Orhan F, Yaris N, Bahat E, et al. Crimean-Congo haemorrhagic fever among children in north-eastern Turkey. Annals of Tropical Paediatrics. 2009;29(1):23-8.

[220] Dinerman H, Steere AC. Lyme Disease Associated with Fibromyalgia. Annals of Internal Medicine. 1992;117(4):281-5.

[221] Doddananjayya R. Incidence of Q Fever in Eastern Washington: A Serological Survey. Public Health Reports (1896-1970). 1949;64(39).

[222] Dong Z, Yang M, Wang Z, Zhao S, Xie S, Yang Y, et al. Human Tacheng Tick Virus 2 Infection, China, 2019. Emerging Infectious Diseases. 2021;27(2):594-8.

[223] Drancourt M, Raoult D, Xeridat B, Milandre L, Nesri M, Dano P. Q fever nieningoencephalitis in five patients. European Journal of Epidemiology. 1991;7(2).

[224] Dredla B, Freeman WD. Ehrlichia Meningitis Mimicking Aneurysmal Subarachnoid Hemorrhage. The Neurohospitalist. 2015;6(2):76-9.

[225] Duan L, Zhang L, Hou X, Bao Z, Zeng Y, He L, et al. Surveillance of tick-borne bacteria infection in ticks and forestry populations in Inner Mongolia, China. Frontiers in Public Health. 2024;12.

[226] Dülger AC, Yakarişik M, Uzun YE, Şahin AM. Treatment of Crimean-Congo Haemorrhagic Fever by Favipiravir in a Patient with Novel Coronavirus Co-Infection. Eur J Case Rep Intern Med. 2020;7(12):002042.

[227] Dumic I, Madrid C, Vitorovic D. Unusual cause at an unusual time-Powassan virus rhombencephalitis. International Journal of Infectious Diseases. 2021;103:88-90.
[228] Dumic I, Person E, Igandan O, Adetimehin O, Nordstrom CW, Williams C, et al. Anaplasma phagocytophilum Community-Acquired Pneumonia: Case Report and Literature Review. Microorganisms. 2023;11(6).
[229] Dumler JS, Dey C, Meier F, Lewis LL. Human Monocytic Ehrlichiosis: A Potentially Severe Disease in Children. Arch Pediatr Adolesc Med. 2000;154(8).
[230] Dupeyron A, Lecocq J, Jaulhac B, Isner-Horobeti ME, Vautravers P, Cohen-Solal J, et al. Sciatica, disk herniation, and neuroborreliosis. A report of four cases. Joint Bone Spine. 2004;71(5):433-7.
[231] Dupont H, Raoult D, Brouqui P, Janbon F, Peyramond D, Weiller P-J, et al. Epidemiologic features and clinical presentation of acute Q fever in hospitalized patients: 323 French cases. The American Journal of Medicine. 1992;93(4):427-34.
[232] Durupt S, Puget M, Lega JC, Durieu I, Reynaud Q. Coxiella burnetii infection (Q fever) mimicking systemic lupus erythematosus: two cases. Lupus. 2017;27(6):1027-9.
[233] Dutasta F, Richaud C, Michon A, Ragone E, Podglajen I, Mainardi J-L. Use of 18F-FDG PET/CT for diagnosis of vascular graft infection with spread to sternum caused by <i>Coxiella burnetii</i>. Infectious Diseases. 2016;48(10):769-71.
[234] Duygu F, Kaya T, Baysan P. Re-Evaluation of 400 Crimean-Congo Hemorrhagic Fever Cases in an Endemic Area: Is Ribavirin Treatment Suitable? Vector-Borne and Zoonotic Diseases. 2012;12(9):812-6.
[235] Duygu F, Sari T, Gunal O, Barut S, Atay A, Aytekin F. Cutaneous Findings of Crimean-Congo Hemorrhagic Fever: a Study of 269 Cases. Japanese Journal of Infectious Diseases. 2018;71(6):408-12.
[236] Duygu F, Sari T, Kaya T, Bulut N. Brucellosis in Patients with Crimean-Congo Hemorrhagic Fever. Journal of Arthropod-borne Diseases. 2017;11(4):463-8.
[237] E.Alieva E, T.Gafarova M, I.Bondarenko E, A.Dovgan I, I.Osiptchuk I, E.Eremeeva M. Use of eschar swab DNA to diagnose Rickettsia conorii subspecies conorii infection in Crimea:A case report. 感染医学（英文）. 2023(4):338-42.
[238] Edouard S, Koebel C, Goehringer F, Socolovschi C, Jaulhac B, Raoult D, et al. Emergence of human granulocytic anaplasmosis in France. Ticks and Tick-Borne Diseases. 2012;3(5-6):402-4.
[239] Edouard S, Million M, Royer G, Giorgi R, Grisoli D, Raoult D. Reduction in incidence of Q fever endocarditis: 27 years of experience of a national reference center. Journal of Infection. 2014;68(2):141-8.
[240] Edwards MS, Jones JE, Leass DL, Whitmore JW, Dawson JE, Fishbein DB. Childhood infection caused by Ehrlichia canis or a closely related organism. The Pediatric Infectious Disease Journal. 1988;7(9):651-4.
[241] El Khoury L, Furie R. Inflammatory arthritis: a unique presentation of human anaplasmosis. Clin Rheumatol. 2019;38(1):257-9.
[242] El Sayed Zaki M, Goda T. Clinico-pathological study of atypical pathogens in community-acquired pneumonia: a prospective study. The Journal of Infection in Developing Countries. 2009;3(03).
[243] El-Azazy OME, Scrimgeour EM. Crimean-Congo haemorrhagic fever virus infection in the

Western Province of Saudi Arabia. Transactions of the Royal Society of Tropical Medicine and Hygiene. 1997;91(3):275-8.

[244] El-Mahallawy HS, Kelly P, Zhang J, Yang Y, Wei L, Tian L, et al. Serological and molecular evidence of Coxiella burnetii in samples from humans and animals in China. Annals of Agricultural and Environmental Medicine. 2016;23(1):87-91.

[245] El-Mokhtar MA, Sayed IM, Kamel AM, Mesalam AA, Elgohary EA, Khalaf KAB, et al. The First Report of Coxiella burnetii as a Potential Neglected Pathogen of Acute Hepatitis of Unknown Causes in Egypt. Microorganisms. 2022;10(11).

[246] Elevli M, Ozkul AA, Civilibal M, Midilli K, Gargili A, Duru NS. A newly identified Crimean-Congo hemorrhagic fever virus strain in Turkey. International Journal of Infectious Diseases. 2010;14:E213-E6.

[247] Elyan DS, Moustafa L, Noormal B, Jacobs JS, Aziz MA, Hassan KS, et al. Serological evidence of Flaviviruses infection among acute febrile illness patients in Afghanistan. Journal of Infection in Developing Countries. 2014;8(9):1176-80.

[248] Elzein FE, Alsherbeeni N, Alnajashi K, Alsufyani E, Akhtar MY, Albalawi R, et al. Ten-year experience of Q fever endocarditis in a tertiary cardiac center in Saudi Arabia. International Journal of Infectious Diseases. 2019;88:21-6.

[249] Emiroglu M, Celebi B, Alkan G, Yilmaz Y. The first human case of Rickettsia slovaca from Turkey. Ticks and Tick-Borne Diseases. 2021;12(5).

[250] Engin A, Erdogan H, Ozec AV, Elaldi N, Toker MI, Bakir M, et al. Ocular Findings in Patients with Crimean-Congo Hemorrhagic Fever. American Journal of Ophthalmology. 2009;147(4):634-8.

[251] Epelboin L, Mahamat A, Bonifay T, Demar M, Abboud P, Walter G, et al. Q Fever as a Cause of Community-Acquired Pneumonia in French Guiana. American Journal of Tropical Medicine and Hygiene. 2022;107(2):407-15.

[252] Ergas D, Keysari A, Edelstein V, Sthoeger ZM. Acute Q fever in Israel: Clinical and laboratory study of 100 hospitalized patients. Israel Medical Association Journal. 2006;8(5):337-41.

[253] Ergunay K, Saygan MB, Aydogan S, Litzba N, Sener B, Lederer S, et al. Confirmed Exposure to Tick-Borne Encephalitis Virus and Probable Human Cases of Tick-Borne Encephalitis in Central/Northern Anatolia, Turkey. Zoonoses and Public Health. 2011;58(3):220-7.

[254] Erickson TA, Mayes B, Murray KO, Gunter SM. The epidemiology of human ehrlichiosis in Texas, 2008-2017. Ticks and Tick-Borne Diseases. 2021;12(6).

[255] Ertugrul B, Kirdar S, Ersoy OS, Ture M, Erol N, Ozturk B, et al. The seroprevalence of Crimean-Congo haemorrhagic fever among inhabitants living in the endemic regions of Western Anatolia. Scandinavian Journal of Infectious Diseases. 2012;44(4):276-81.

[256] Ertugrul B, Uyar Y, Yavas K, Turan C, Oncu S, Saylak O, et al. An outbreak of Crimean-Congo hemorrhagic fever in western Anatolia, Turkey. International Journal of Infectious Diseases. 2009;13(6):E431-E6.

[257] Esen B, Gozalan A, Coplu N, Tapar FS, Uzun R, Aslan T, et al. The presence of tick-borne encephalitis in an endemic area for tick-borne diseases, Turkey. Tropical Doctor. 2008;38(1):27-8.

[258] Esmaeili S, Amiri FB, Mokhayeri H, Kayedi MH, Maurin M, Rohani M, et al. Seroepidemiological study of Q fever, brucellosis and tularemia in butchers and slaughterhouses

workers in Lorestan, western of Iran. Comparative Immunology Microbiology and Infectious Diseases. 2019;66.

[259] Esmaeili S, Golzar F, Ayubi E, Naghili B, Mostafavi E. Acute Q fever in febrile patients in northwestern of Iran. Plos Neglected Tropical Diseases. 2017;11(4).

[260] Esmaeili S, Latifian M, Khalili M, Farrokhnia M, Stenos J, Shafiei M, et al. Fatal Case of Mediterranean Spotted Fever Associated with Septic Shock, Iran. Emerging Infectious Diseases. 2022;28(2):485-8.

[261] Esmaeili S, Naddaf SR, Pourhossein B, Shahraki AH, Amiri FB, Gouya MM, et al. Seroprevalence of Brucellosis, Leptospirosis, and Q Fever among Butchers and Slaughterhouse Workers in South-Eastern Iran. PLoS ONE. 2016;11(1).

[262] Etienne J, Delahaye F, Raoult R, Frieh JP, Loire R, Delaye J. Acute heart failure due to Q fever endocarditis. European Heart Journal. 1988;9(8):923-6.

[263] Fagre AC, Lyons S, Staples JE, Lindsey N. West Nile Virus and Other Nationally Notifiable Arboviral Diseases — United States, 2021. MMWR Morbidity and Mortality Weekly Report. 2023;72(34):901-6.

[264] Farrington M, Elenz J, Ginsberg M, Chiu CY, Miller S, Pangonis SF. Powassan Virus Infection Detected by Metagenomic Next-Generation Sequencing, Ohio, USA. Emerging Infectious Diseases. 2023;29(4):838-41.

[265] Farrokhnia M, Yousefi Ghalejoogh Z, Rohani M, Ghasemi A, Esmaeili S, Mostafavi E. Cases of Mediterranean spotted fever in southeast of Iran. Iranian Journal of Microbiology. 2020.

[266] Faruque LI, Zaman RU, Gurley ES, Massung RF, Alamgir ASM, Galloway RL, et al. Prevalence and clinical presentation of Rickettsia, Coxiella, Leptospira, Bartonella and chikungunya virus infections among hospital-based febrile patients from December 2008 to November 2009 in Bangladesh. BMC Infectious Diseases. 2017;17(1).

[267] Fateh BA, Golah HA, Qudari AYA, Al Garadi MA, Alhothy HA. Detection of Coxiella burnetti Antibodies among Workers and Butchers at Dhamar Slaughter House, Yemen. International Journal of Current Microbiology and Applied Sciences. 2019;8(03):361-5.

[268] Fatima H, Qadir TF, Ahmed S, Moin A. Congo fever rears its head again in Pakistan. J Infect Public Health. 2017;10(3):366-7.

[269] Fazlalipour M, Baniasadi V, Mirghiasi SM, Jalali T, Khakifirouz S, Azad-Manjiri S, et al. Crimean-Congo Hemorrhagic Fever Due to Consumption of Raw Meat: Case Reports From East-North of Iran. Japanese Journal of Infectious Diseases. 2016;69(3):270-1.

[270] Ferrante MA, Dolan MJ. Q Fever Meningoencephalitis in a Soldier Returning from the Persian Gulf War. Clinical Infectious Diseases. 1993;16(4):489-96.

[271] Figueroa R, Bracero LA, Aguero-Rosenfeld M, Beneck D, Coleman J, Schwartz I. Confirmation ofBorrelia burgdorferiSpirochetes by Polymerase Chain Reaction in Placentas of Women with Reactive Serology for Lyme Antibodies. Gynecologic and Obstetric Investigation. 1996;41(4):240-3.

[272] Fine AB, Sweeney JD, Nixon CP, Knoll BM. Transfusion-transmitted anaplasmosis from a leukoreduced platelet pool. Transfusion. 2016;56(3):699-704.

[273] Finn T, Babushkin F, Geller K, Alexander H, Paikin S, Lellouche J, et al. Epidemiological, clinical and laboratory features of acute Q fever in a cohort of hospitalized patients in a regional hospital, Israel, 2012-2018. Plos Neglected Tropical Diseases. 2021;15(7).

[274] Fisgin NT, Fisgin T, Tanyel E, Doganci L, Tulek N, Guler N, et al. Crimean-Congo hemorrhagic fever: Five patients with hemophagocytic syndrome. American Journal of Hematology. 2008;83(1):73-6.
[275] Foucault C, Lepidi H, Poujet-Abadie JF, Granel B, Roblot F, Ariga T, et al. Q fever and lymphadenopathy: report of four new cases and review. European Journal of Clinical Microbiology & Infectious Diseases. 2004;23(10):759-64.
[276] Fournier PE, Etienne J, Harle JR, Habib G, Raoult D. Myocarditis, a Rare but Severe Manifestation of Q Fever: Report of 8 Cases and Review of the Literature. Clinical Infectious Diseases. 2001;32(10):1440-7.
[277] Franck M, Ghozzi R, Pajaud J, Lawson-Hogban NE, Mas M, Lacout A, et al. <i>Borrelia miyamotoi</i>: 43 Cases Diagnosed in France by Real-Time PCR in Patients With Persistent Polymorphic Signs and Symptoms. Frontiers in Medicine. 2020;7.
[278] Freylikhman O, Kiselev A, Kazakov S, Sergushichev A, Panferova Y, Tokarevich N, et al. Draft Genome Sequence ofCoxiella burnetiiHistorical Strain Leningrad-2, Isolated from Blood of a Patient with Acute Q Fever in Saint Petersburg, Russia. Genome Announcements. 2018;6(3).
[279] Galy A, Decousser JW, El-Anbassi S, Nebbad B, Belzunce C, Cochennec F, et al. Psoas abscess and chronic Q fever: a contiguous or hematogenous complication? A case report and literature review. Infectious Diseases. 2016;48(8):626-31.
[280] Gao Y, Che L, Wang Z, Niu J, Wei W, Song M, et al. A case report of autochthonous Q fever with pneumonia and hepatitis in northeastern China. Biosafety and Health. 2021;3(3):179-82.
[281] Gao Y, Lv X-L, Han S-Z, Wang W, Liu Q, Song M. First detection of Borrelia miyamotoi infections in ticks and humans from the northeast of Inner Mongolia, China. Acta Tropica. 2021;217.
[282] Gaowa, Wulantuya, Yin X, Cao M, Guo S, Ding C, et al. Case of Human Infection with Anaplasma phagocytophilum in Inner Mongolia, China. Japanese Journal of Infectious Diseases. 2018;71(2):155-7.
[283] Gargili A, Midilli K, Ergonul O, Ergin S, Alp HG, Vatansever Z, et al. Crimean-Congo Hemorrhagic Fever in European Part of Turkey: Genetic Analysis of the Virus Strains from Ticks and a Seroepidemiological Study in Humans. Vector-Borne and Zoonotic Diseases. 2011;11(6):747-52.
[284] Gavina K, Whitacre BE, Meyer TL, Van Benten K, Glazier M, Emery CL, et al. The Brief Case: Suspicious Gram-Negative Coccobacilli—Francisella tularensis subsp.novicida Isolated from an Immunocompromised Patient. Journal of Clinical Microbiology. 2023;61(6).
[285] Gayretli Aydin ZG, Yesilbas O, Reis GP, Guven B. The first pediatric case of hemophagocytic lymphohistiocytosis secondary to Crimean-Congo haemorrhagic fever successfully treated with therapeutic plasma exchange accompanying ribavirin and intravenous immunoglobulin. J Clin Apher. 2021;36(5):780-4.
[286] Gazi H, Özkütük N, Ecemis Ö, Atasoylu G, Köroglu G, Kurutepe S, et al. Seroprevalence of West Nile virus, Crimean-Congo hemorrhagic fever virus, Francisella tularensis and Borrelia burgdorferi in rural population of Manisa, western Turkey. Journal of Vector Borne Diseases. 2016;53(2).
[287] Gellis SE, Stadecker MJ, Steere AC. Spirochetes in atrophic skin lesions accompanied by minimal host response in a child with Lyme disease. Journal of the American Academy of

Dermatology. 1991;25(2):395-7.
[288] Genova-Kalou P, Vladimirova N, Stoitsova S, Krumova S, Kurchatova A, Kantardjiev T. Q fever in Bulgaria: Laboratory and epidemiological findings on human cases and outbreaks, 2011 to 2017. Eurosurveillance. 2019;24(37).
[289] Georghiou GP, Hirsch R, Vidne BA, Raanani E. Coxiella burnetii infection of an aortic graft: surgical view and a word of caution. Interact Cardiovasc Thorac Surg. 2004;3(2):333-5.
[290] Georgilis K, Noring R, Steere AC, Klempner MS. Neutrophil chemotactic factors in synovial fluids of patients with Lyme disease. Arthritis Rheum. 1991;34(6):770-5.
[291] Gergova I, Kamarinchev B. Seroprevalence of Crimean-Congo Hemorrhagic Fever in Southeastern Bulgaria. Japanese Journal of Infectious Diseases. 2014;67(5):397-8.
[292] Germanakis A, Chochlakis D, Angelakis E, Tselentis Y, Psaroulaki A. Rickettsia aeschlimanniiInfection in a Man, Greece. Emerging Infectious Diseases. 2013;19(7):1176-7.
[293] Germanakis A, Psaroulaki A, Gikas A, Tselentis Y. Mediterranean spotted fever in Crete, Greece - Clinical and therapeutic data of 15 consecutive patients. In: Hechemy KE, Oteo JA, Raoult DA, Silverman DJ, Blanco JR, editors. Century of Rickettsiology: Emerging, Reemerging Rickettsioses, Molecular Diagnostics, and Emerging Veterinary Rickettsioses. Annals of the New York Academy of Sciences. 10782006. p. 263-9.
[294] Gewirtz AS, Cornbleet PJ, Vugia DJ, Traver C, Niederhuber J, Kolbert CP, et al. Human Granulocytic Ehrlichiosis: Report of a Case in Northern California. Clinical Infectious Diseases. 1996;23(3):653-4.
[295] Ghanem-Zoubi N, Karram T, Kagna O, Merhav G, Keidar Z, Paul M. Q fever vertebral osteomyelitis among adults: a case series and literature review. Infectious Diseases. 2021;53(4):231-40.
[296] Ghanem-Zoubi N, Paul M, Szwarcwort M, Agmon Y, Kerner A. Screening for Q Fever in Patients Undergoing Transcatheter Aortic Valve Implantation, Israel, June 2018-May 2020. Emerging Infectious Diseases. 2021;27(8):2205-7.
[297] Ghaoui H, Achour N, Saad-Djaballah A, Belacel SI, Bitam I, Fournier PE. Q Fever in Unexplained Febrile Illness in Northern Algeria. Microbiology Research. 2023;14(4):1589-95.
[298] Ghaoui H, Bitam I, Ait-Oudhia K, Achour N, Saad-Djaballah A, Saadnia FZ, et al. Coxiella burnetii infection with women's febrile spontaneous abortion reported in Algiers. New microbes and new infections. 2018;26:8-14.
[299] Gharabaghi MA, Chinikar S, Ghiasi SM, Morady M, Ahmadinejhad T, Paydary K. Severe Crimean-Congo haemorrhagic fever presented with massive retroperitoneal haemorrhage that recovered without antiviral treatment. BMJ Case Rep. 2011;2011.
[300] Ghassemi M, Agger William A, Vanscoy Robert E, Howe Gerald B. Chronic Sternal Wound Infection and Endocarditis withCoxiella burnetii. Clinical Infectious Diseases. 1999;28(6):1249-51.
[301] Ghera P, Kasirye Y, Choudhry MW, Shaw GR, Ejercito VS. Acute transient sensorineural hearing loss due to Anaplasma phagocytophilum. Wmj : Official Publication of The State Medical Society of Wisconsin. 2011;110(6):288-90.
[302] Gibaud M, Pauvert O, Gueden S, Durigneux J, Van Bogaert P. Opsoclonus in a child with neuroborreliosis: Case report and review of the literature. Arch Pediatr. 2019;26(2):118-9.
[303] Gikas A, Kofteridis D, Bouros D, Voloudaki A, Tselentis Y, Tsaparas N. Q Fever Pneumonia:

Appearance on Chest Radiographs. Radiology. 1999;210(2):339-43.
[304] Girard YA, Fedorova N, Lane RS. Genetic diversity of Borrelia burgdorferi and detection of B. bissettii-like DNA in serum of north-coastal California residents. Journal of Clinical Microbiology. 2011;49(3):945-54.
[305] Goel R, Westblade LF, Kessler DA, Sfeir M, Slavinski S, Backenson B, et al. Death from Transfusion-Transmitted Anaplasmosis, New York, USA, 2017. Emerging Infectious Diseases. 2018;24(8):1548-50.
[306] Goldberg S, Katz BZ. Lyme Disease Presenting as Ptosis, Conjunctivitis, and Photophobia. Clinical Pediatrics. 2012;51(2):186-7.
[307] Goldstein MD, Schwartz BS, Friedmann C, Maccarillo B, Borbi M, Tuccillo R. Lyme disease in New Jersey outdoor workers: a statewide survey of seroprevalence and tick exposure. American Journal of Public Health. 1990;80(10):1225-9.
[308] Gönen I, Ermiş F. Crimean-Congo hemorrhagic fever presenting with gastrointestinal manifestations: two cases. Turk J Gastroenterol. 2014;25(1):120-1.
[309] Goodman JL, Jurkovich P, Kramber JM, Johnson RC. Molecular detection of persistent Borrelia burgdorferi in the urine of patients with active Lyme disease. Infection and Immunity. 1991;59(1):269-78.
[310] Gouriet F, Rolain J-M, Raoult D. Rickettsia slovacaInfection, France. Emerging Infectious Diseases. 2006;12(3):521-3.
[311] Gozalan A, Esen B, Fitzner J, Tapar FS, Ozkan AP, Georges-Courbot M-C, et al. Crimean-Congo haemorrhagic fever cases in Turkey. Scandinavian Journal of Infectious Diseases. 2007;39(4):332-6.
[312] Gozalan A, Rolain JM, Ertek M, Angelakis E, Coplu N, Basbulut EA, et al. Seroprevalence of Q fever in a district located in the west Black Sea region of Turkey. European Journal of Clinical Microbiology & Infectious Diseases. 2010;29(4):465-9.
[313] Gozdas HT. Evaluation of Crimean-Congo hemorrhagic fever suspected cases admitted to a secondary care hospital in Kastamonu, Turkey between 2014-2017. African Health Sciences. 2019;19(1):1433-40.
[314] Graham CJ, Yamauchi T, Rountree P. Q fever in animal laboratory workers: An outbreak and its investigation. American Journal of Infection Control. 1989;17(6):345-8.
[315] Graham JV, Baden L, Tsiodras S, Karchmer AW. Q Fever Endocarditis Associated with Extensive Serological Cross-Reactivity. Clinical Infectious Diseases. 2000;30(3):609-10.
[316] Grant AC, Hunter S, Partin WC. A case of acute monocytic ehrlichiosis with prominent neurologic signs. Neurology. 1997;48(6):1619-23.
[317] Grant L, Mohamedy I, Loertscher L. One man, three tick-borne illnesses. BMJ Case Rep. 2021;14(4).
[318] Granter SR. Identification of Borrelia burgdorferi in diffuse fasciitis with peripheral eosinophilia: borrelial fasciitis. JAMA: The Journal of the American Medical Association. 1994;272(16):1283-5.
[319] Gu M, Mo X, Tang Z, Tang J, Wang W. Case Report: Diagnosis of Acute Q Fever With Aseptic Meningitis in a Patient by Using Metagenomic Next-Generation Sequencing. Front Med (Lausanne). 2022;9:855020.
[320] Guardado KE, Sergent S. Pediatric unilateral knee swelling: a case report of a complicated

differential diagnosis and often overlooked cause. J Osteopath Med. 2022;122(2):105-9.
[321] Guet-Revillet H, Levy C, Vallet C, Maghraoui-Slim V, Dommergues MA, Hentgen V, et al. Lyme neuroborreliosis in children: Report of nine cases and a review of the literature. Archives De Pediatrie. 2019;26(3):133-7.
[322] Gugliotta JL, Goethert HK, Berardi VP, Telford SR, III. Meningoencephalitis from Borrelia miyamotoi in an immunocompromised patient. New England Journal of Medicine. 2013;368(3):240-5.
[323] Gul I, Kaya A, Guven AS, Karapinar H, Kucukdurmaz Z, Yılmaz A, et al. Cardiac findings in children with Crimean-Congo hemorrhagic fever. Medical Science Monitor. 2011;17(8):CR456-CR9.
[324] Gülhan B, Kanık-Yüksek S, Çetin İ, Özkaya-Parlakay A, Tezer H. Myocarditis in a Child with Crimean-Congo Hemorrhagic Fever. Vector Borne Zoonotic Dis. 2015;15(9):565-7.
[325] Guner R, Hasanoglu I, Yapar D, Tasyaran MA. A case of Crimean Congo hemorrhagic fever complicated with acalculous cholecystitis and intraabdominal abscess. Journal of Clinical Virology. 2011;50(2):162-3.
[326] Gunes T, Engin A, Poyraz O, Elaldi N, Kaya S, Dokmetas I, et al. Crimean-Congo Hemorrhagic Fever Virus in High-Risk Population, Turkey. Emerging Infectious Diseases. 2009;15(3):461-4.
[327] Güneş T, Erturk R, Poyraz Ö. Serosurvey of Coxiella burnetii in high risk population in Turkey, endemic to Crimean-Congo haemorrhagic fever virus. Journal of Vector Borne Diseases. 2017;54(4).
[328] Gunes T, Poyraz O, Atas M, Turgut NH. The seroprevalence of Anaplasma phagocytophilum in humans from two different climatic regions of Turkey and its co-seroprevalence rate with Borrelia burgdorferi. Turkish Journal of Medical Sciences. 2011;41(5):903-8.
[329] Gunes T, Poyraz O, Atas M, Turgut NH. The seroprevalence of Rickettsia conorii in humans living in villages of Tokat Province in Turkey, where Crimean-Congo hemorrhagic fever virus is endemic, and epidemiological similarities of both infectious agents. Turkish Journal of Medical Sciences. 2012;42(3):441-8.
[330] Gunn TM, Raz GM, Turek JW, Farivar RS. Cardiac manifestations of Q fever infection: case series and a review of the literature. J Card Surg. 2013;28(3):233-7.
[331] Gupta N, Chaudhry R, Valappil VE, Soneja M, Ray A, Kumar U, et al. Lyme arthritis: A prospective study from India. Journal of Family Medicine and Primary Care. 2019;8(12):4046-7.
[332] Gupta PK, Patel R, Bhatti MT. Neuroretinitis secondary to concurrent infection with cat scratch disease and lyme disease. Eye. 2009;23(7):1607-.
[333] Gurbuz E, EkIcI A, Unlu AH, Yilmaz H. Evaluation of seroprevalence and clinical and laboratory findings of patients admitted to health institutions in Gumushane with suspicion of Crimean-Congo hemorrhagic fever. Turkish Journal of Medical Sciences. 2021;51(4):1825-32.
[334] Gurbuz Y, Sencan I, Ozturk B, Tutuncu E. A case of nosocomial transmission of Crimean-Congo hemorrhagic fever from patient to patient. International journal of infectious diseases : IJID : official publication of the International Society for Infectious Diseases. 2009;13(3):e105-7.
[335] Guven FMK, Aydin H, Yildiz G, Engin A, Celik VK, Bakir D, et al. The importance of myeloperoxidase enzyme activity in the pathogenesis of Crimean-Congo haemorrhagic fever. Journal of Medical Microbiology. 2013;62:441-5.
[336] Habibzadeh S, Mohammadshahi J, Bakhshzadeh A, Moradi-Asl E. The First Outbreak of

Crimean-Congo Hemorrhagic Fever Disease in Northwest of Iran. Acta Parasitologica. 2021;66(3):1086-8.

[337] Haddad O, Gillinov M, Fraser T, Shrestha N, Pettersson GB. Mitral Valve Endocarditis: A Rare Manifestation of Lyme Disease. Annals of Thoracic Surgery. 2019;108(2):E85-E6.

[338] Halasz CLG, Niedt GW, Kurtz CP, Scorpio DG, Bakken JS, Dumler JS. A Case of Sweet Syndrome Associated With Human Granulocytic Anaplasmosis. Archives of Dermatology. 2005;141(7).

[339] Halperin J, Luft BJ, Volkman DJ, Dattwyler RJ. Lyme Neuroborreliosis: Peripheral Nervous System Manifestations. Brain. 1990;113(4):1207-21.

[340] Hamilton KS, Standaert SM, Kinney MC. Characteristic peripheral blood findings in human ehrlichiosis. Mod Pathol. 2004;17(5):512-7.

[341] Hammill WW, Wilson MB, Reigart JR, Flick JT, Laver J. Ehrlichia canis Infection in a Child in South Carolina. Clinical Pediatrics. 1992;31(7):432-4.

[342] Han X, Hsu J, Miao Q, Zhou B-T, Fan H-W, Xiong X-L, et al. Retrospective Examination of Q Fever Endocarditis. Chinese Medical Journal. 2017;130(1):64-70.

[343] Hansmann Y, Gut JP, Remy V, Martinot M, Witz MA, Christmann D. Tick-borne encephalitis in eastern France. Scandinavian Journal of Infectious Diseases. 2006;38(6-7):520-6.

[344] Hanson D, Walter AW, Powell J. Ehrlichia-induced hemophagocytic lymphohistiocytosis in two children. Pediatr Blood Cancer. 2011;56(4):661-3.

[345] Hao Q, Geng Z, Hou XX, Tian Z, Yang XJ, Jiang WJ, et al. Seroepidemiological Investigation of Lyme Disease and Human Granulocytic Anaplasmosis among People Living in Forest Areas of Eight Provinces in China. Biomedical And Environmental Sciences. 2013;26(3):185-9.

[346] Hardalo CJ, Quagliarello V, Dumler JS. Human Granulocytic Ehrlichiosis in Connecticut: Report of a Fatal Case. Clinical Infectious Diseases. 1995;21(4):910-4.

[347] Hariri S, Joseph S, Forsythe SJ. Cronobacter sakazakiiST4 Strains and Neonatal Meningitis, United States. Emerging Infectious Diseases. 2013;19(1):175-7.

[348] Harkess JR, Conrad ME. Ehrlichiosis: A cause of bone marrow hypoplasia in humans. American Journal of Hematology. 2006;30(4):265-6.

[349] Harkess JR, Ewing SA, Crulcher JM, Kudlac J, McKee G, Istre GR. Human Ehrlichiosis in Oklahoma. The Journal of Infectious Diseases. 1989;159(3):576-9.

[350] Hartzell JD, Peng SW, Wood-Morris RN, Sarmiento DM, Collen JF, Robben PM, et al. Atypical Q Fever in US Soldiers. Emerging Infectious Diseases. 2007;13(8):1247-9.

[351] Hasan Z, Mahmood F, Jamil B, Atkinson B, Mohammed M, Samreen A, et al. Crimean-Congo hemorrhagic fever nosocomial infection in a immunosuppressed patient, Pakistan: Case report and virological investigation. Journal of Medical Virology. 2013;85(3):501-4.

[352] Hassan W, Talwar M, Balaraman V, Molnar MZ. Ehrlichiosis infection mimicking thrombotic microangiopathy syndrome early after kidney transplantation. Transplant Infectious Disease. 2020;22(5).

[353] Hassanein KM, El-Azazy OME, Yousef HM. Detection of Crimean-Congo haemorrhagic fever virus antibodies in humans and imported livestock in Saudi Arabia. Transactions of the Royal Society of Tropical Medicine and Hygiene. 1997;91(5):536-7.

[354] Hatami H, Qaderi S, Omid AM. Investigation of Crimean-Congo hemorrhagic Fever in Patients Admitted in Antani Hospital, Kabul, Afghanistan, 2017-2018. International Journal of Preventive

Medicine. 2019;10.
[355] Hatipoglu CA, Bulut C, Yetkin MA, Ertem GT, Erdinc FS, Kilic EK, et al. Evaluation of clinical and laboratory predictors of fatality in patients with Crimean-Congo haemorrhagic fever in a tertiary care hospital in Turkey. Scandinavian Journal of Infectious Diseases. 2010;42(6-7):516-21.
[356] Havens NS, Kinnear BR, Mato S. Fatal Ehrlichial Myocarditis in a Healthy Adolescent: A Case Report and Review of the Literature. Clinical Infectious Diseases. 2012;54(8):E113-E4.
[357] Herpe B, Schuffenecker I, Pillot J, Malvy D, Clouzeau B, Bui N, et al. Tickborne Encephalitis, Southwestern France. Emerging Infectious Diseases. 2007;13(7):1114-6.
[358] Heydari AA, Mostafavi E, Heidari M, Latifian M, Esmaeili S. Q Fever Endocarditis in Northeast Iran. Case Reports in Infectious Diseases. 2021;2021.
[359] Hilton E, DeVoti J, Benach JL, Halluska ML, White DJ, Paxton H, et al. Seroprevalence and seroconversion for tick-borne diseases in a high-risk population in the northeast United States. The American Journal of Medicine. 1999;106(4):404-9.
[360] Holmgren AR, Matteson EL. Lyme myositis. Arthritis Rheum. 2006;54(8):2697-700.
[361] Hong DK, Tremoulet AH, Burns JC, Lewis DB. Cross-Reactive Neutralizing Antibody against Pandemic 2009 H1n1 Influenza a Virus in Intravenous Immunoglobulin Preparations. Pediatric Infectious Disease Journal. 2011;30(1):67-9.
[362] Horowitz HW, Dworkin B, Forseter G, Nadelman RB, Connolly C, Luciano BB, et al. Liver Function in Early Lyme Disease. Hepatology. 1996;23(6):1412-7.
[363] Horowitz HW, Kilchevsky E, Haber S, Aguero-Rosenfeld M, Kranwinkel R, James EK, et al. Perinatal Transmission of the Agent of Human Granulocytic Ehrlichiosis. New England Journal of Medicine. 1998;339(6):375-8.
[364] Horowitz HW, Sanghera K, Goldberg N, Pechman D, Kamer R, Duray P, et al. Dermatomyositis Associated with Lyme Disease: Case Report and Review of Lyme Myositis. Clinical Infectious Diseases. 1994;18(2):166-71.
[365] Horowitz R, Freeman PR. Improvement of common variable immunodeficiency using embryonic stem cell therapy in a patient with lyme disease: a clinical case report. Clin Case Rep. 2018;6(6):1166-71.
[366] Hosseini-Vasoukolaei N, Oshaghi MA, Shayan P, Vatandoost H, Babamahmoudi F, Yaghoobi-Ershadi MR, et al. Anaplasma Infection in Ticks, Livestock and Human in Ghaemshahr, Mazandaran Province, Iran. Journal of Arthropod-borne Diseases. 2014;8(2):204-11.
[367] Houpikian P, Habib G, Mesana T, Raoult D. Changing Clinical Presentation of Q Fever Endocarditis. Clinical Infectious Diseases. 2002;34(5):e28-e31.
[368] Houpikian P, Raoult D. Blood culture-negative endocarditis in a reference center - Etiologic diagnosis of 348 cases. Medicine. 2005;84(3):162-73.
[369] Huang M, Ma J, Jiao J, Li C, Chen L, Zhu Z, et al. The epidemic of Q fever in 2018 to 2019 in Zhuhai city of China determined by metagenomic next-generation sequencing. Plos Neglected Tropical Diseases. 2021;15(7).
[370] Hussein ZJ, Al Jalboubi AS, Al Busaidi I. Acute Q fever in an Omani presenting with a febrile movement disorder - A Case Report. IDCases. 2023;33:e01861.
[371] Idris MA, Ruppel A, Petney T. Antibodies against rickettsia in humans and potential vector ticks from Dhofar, Oman. Journal For Scientific Research Medical Sciences. 2000;2(1):7-10.

[372] Ince Y, Yasa C, Metin M, Sonmez M, Meram E, Benkli B, et al. Crimean-Congo hemorrhagic fever infections reported by ProMED. International Journal of Infectious Diseases. 2014;26:44-6.

[373] Ingram D, Joseph B, Hawkins S, Spain J. Anaplasmosis in Pennsylvania: Clinical Features, Diagnosis, and Outcomes of Patients Diagnosed With Anaplasma phagocytophilum Infection at Hershey Medical Center From 2008 to 2021. Open Forum Infectious Diseases. 2023;10(4).

[374] Izadi S, Naieni KH, Madjdzadeh SR, Nadim A. Crimean-Congo hemorrhagic fever in Sistan and Baluchestan Province of Iran, a case-control study on epidemiological characteristics. International Journal of Infectious Diseases. 2004;8(5):299-306.

[375] Izcı F, Ture Z, Sagiroglu P, Temel S, Yildiz O. A case of Crimean-Congo hemorrhagic fever with the bacteremia of Clostridium perfringens. J Med Virol. 2021;93(6):3929-33.

[376] Jabbari A, Besharat S, Abbasi A, Moradi A, Kalavi K. Crimean-Congo hemorrhagic fever: case series from a medical center in Golestan province, Northeast of Iran (2004-2006). Indian Journal of Medical Sciences. 2006;60(8):327-9.

[377] Jacobs C. Services overseas. Public Health. 1982;96(6).

[378] Jacobson A, Sutthiwan P. Myocarditis: A rare manifestation of acute Q fever infection. J Cardiol Cases. 2019;20(2):45-8.

[379] Jahfari S, Herremans T, Platonov AE, Kuiper H, Karan LS, Vasilieva O, et al. High seroprevalence of Borrelia miyamotoi antibodies in forestry workers and individuals suspected of human granulocytic anaplasmosis in the Netherlands. New Microbes and New Infections. 2014;2(5):144-9.

[380] Jairath V, Sehrawat M, Jindal N, Jain VK, Aggarwal P. Lyme disease in Haryana, India. Indian Journal of Dermatology Venereology & Leprology. 2014;80(4):320-3.

[381] Jamil B, Hasan RS, Sarwari AR, Burton J, Hewson R, Clegg C. Crimean-Congo hemorrhagic fever: experience at a tertiary care hospital in Karachi, Pakistan. Transactions of the Royal Society of Tropical Medicine and Hygiene. 2005;99(8):577-84.

[382] Janbon F, Raoult D, Reynes J, Bertrand A. Concomitant Human Infection Due to Rickettsia conorii and Coxiella burnetii. Journal of Infectious Diseases. 1989;160(2):354-5.

[383] Jares TM, Mathiason MA, Kowalski TJ. Functional outcomes in patients with Borrelia burgdorferi reinfection. Ticks and Tick-Borne Diseases. 2014;5(1):58-62.

[384] Jaubert J, Atiana L, Larrieu S, De Vos P, Somon-Payet C, Porcherat S, et al. Q fever seroprevalence in parturient women: the EQRUN cross-sectional study on Reunion Island. Bmc Infectious Diseases. 2020;20(1).

[385] Jaubert J, Naze F, Camuset G, Larrieu S, Pascalis H, Guernier V, et al. Seroprevalence of Coxiella burnetii (Q fever) Exposure in Humans on Reunion Island. Open Forum Infect Dis. 2019;6(7):ofz227.

[386] Jawanda J. Rare case of severe rhabdomyolysis secondary to human granulocytic anaplasmosis. Am J Emerg Med. 2020;38(7):1543.e1-.e2.

[387] Jha P, Pereira SGR, Thakur A, Jhaj G, Bhandari S. A Case of Optic Neuritis Secondary to Lyme Disease. Wmj : Official Publication of The State Medical Society of Wisconsin. 2018;117(2):83-7.

[388] Jia N, Liu H-B, Zheng Y-C, Shi W-Q, Wei R, Chu Y-L, et al. Cutaneous Immunoprofiles of Three Spotted Fever Group Rickettsia Cases. Infection and Immunity. 2020;88(4).

[389] Jia N, Zheng Y-C, Ma L, Huo Q-B, Ni X-B, Jiang B-G, et al. Human Infections with Rickettsia raoultii, China. Emerging Infectious Diseases. 2014;20(5):866-8.

[390] Jiang B-G, Jia N, Jiang J-F, Zheng Y-C, Chu Y-L, Jiang R-R, et al. Borrelia miyamotoi Infections in Humans and Ticks, Northeastern China. Emerging Infectious Diseases. 2018;24(2):236-41.

[391] Jobe DA, Lovrich SD, Oldenburg DG, Kowalski TJ, Callister SM. Borrelia miyamotoiInfection in Patients from Upper Midwestern United States, 2014–2015. Emerging Infectious Diseases. 2016;22(8):1471-3.

[392] Johnson DKH, Schiffman EK, Davis JP, Neitzel DF, Sloan LM, Nicholson WL, et al. Human Infection with Ehrlichia muris like Pathogen, United States, 2007-2013. Emerging Infectious Diseases. 2015;21(10):1794-9.

[393] Johnson DKH, Staples JE, Sotir MJ, Warshauer DM, Davis JP. Tickborne Powassan virus infections among Wisconsin residents. Wmj : Official Publication of The State Medical Society of Wisconsin. 2010;109(2):91-7.

[394] Kadyrov A, Shermukhamedova D, Komilov N, Umurzakov S, Bryanseva E, Nazarbekova M. Reappearance of CCHF and other tick-borne arboviruses in the Syrdarya region of the Republic of Uzbekistan. International Journal of Antimicrobial Agents. 2007;29:S280-S.

[395] Kaell AT. Positive Lyme Serology in Subacute Bacterial Endocarditis. JAMA. 1990;264(22).

[396] Kagan S, Levy I, Ashkenazi-Hoffnung L, Lowenthal A, Goldstein RE, Landau D, et al. Q Fever and Kingella kingae Endocarditis in a Toddler: A Rare Coinfection Case. Pediatric Infectious Disease Journal. 2019;38(12):e336-e7.

[397] Kaltenboeck B, Zhang L, Wang G, Liu Q, Chen C, Li J, et al. Molecular Analysis of Anaplasma phagocytophilum Isolated from Patients with Febrile Diseases of Unknown Etiology in China. PLoS ONE. 2013;8(2).

[398] Karabay O, Gozdas HT, Ozturk G, Tuna N, Utku AC. A Q fever case mimicking Crimean-Congo haemorrhagic fever. Indian Journal of Medical Microbiology. 2011;29(4):418-9.

[399] Karabay O, Kocoglu E, Baysoy G, Konyalioglu S. Coxiella burnetii seroprevalence in the rural part of Bolu, Turkey. Turkish Journal of Medical Sciences. 2009;39(4):641-5.

[400] Karawan B, Zeev W, Moran S-C, Nelly Z-B, Mical P, Nesrin G-Z. Q fever screening among pregnant women with pre-term delivery in northern Israel: An observational study. Zoonoses and Public Health. 2023;70(2):160-5.

[401] Kayal N, Ghosh R, Mazumdar PS, Das S, Ghosh S, Pandit A, et al. Bilateral Facial Nerve Palsy in a Young Woman From West Bengal: Do Not Forget Lyme Neuroborreliosis. Neurology India. 2021;69(4):997-1001.

[402] Kerget F, Demirdogen SO, Kerget B. Case Report: A Rare Case of Crimean-Congo Hemorrhagic Fever Associated with Epididymo-Orchitis. American Journal of Tropical Medicine and Hygiene. 2021;104(3):1055-7.

[403] Kersh GJ, Fitzpatrick K, Pletnikoff K, Brubaker M, Bruce M, Parkinson A. Prevalence of serum antibodies to Coxiella burnetii in Alaska Native Persons from the Pribilof Islands. Zoonoses and Public Health. 2020;67(1):89-92.

[404] Keysary A, Amram L, Keren G, Sthoeger Z, Potasman I, Jacob A, et al. Serologic Evidence of Human Monocytic and Granulocytic Ehrlichiosis in Israel. Emerging Infectious Diseases. 1999;5(6):775-8.

[405] Khaki M, Ghaznavi-Rad E, Ghazavi A, Ghasami K, Mosayeb G. Elevated antibody titer against

Borrelia burgdorferi in new case of multiple sclerosis patients. African Journal of Microbiology Research. 2011;5(30):5354-7.

[406] Khameneie MK, Asadi J, Khalili M, Abiri Z. The First Serological Study of Coxiella burnetii among Pregnant Women in Iran. Iranian Journal of Public Health. 2016;45(4):523-30.

[407] Khan AM, Shahzad SR, Ashraf MF, Naseer U. Powassan virus encephalitis, severe babesiosis and lyme carditis in a single patient. BMJ Case Rep. 2019;12(11).

[408] Khatat SE, Daminet S, Kachani M, Leutenegger CM, Duchateau L, El Amri H, et al. Anaplasma spp. in dogs and owners in north-western Morocco. Parasites & Vectors. 2017;10.

[409] Khatat SE, Sahibi H, Hing M, Moustain IA, El Amri H, Benajiba M, et al. Human Exposure to Anaplasma phagocytophilum in Two Cities of Northwestern Morocco. PLoS ONE. 2016;11(8).

[410] Khatri A, Lloji A, Doobay R, Wang G, Knoll B, Dhand A, et al. Anaplasma phagocytophilum presenting with orchitis in a renal transplant recipient. Transplant Infectious Disease. 2019;21(4).

[411] Khera KD, Southerland DM, Miller NE, Garrison GM. A Case of Anaplasmosis during a Warm Minnesota Fall. Journal of Primary Care and Community Health. 2021;12.

[412] Kidder I, Kobayashi T, Ford B, Sekar P. Hip periprosthetic joint infection due to Coxiella burnetii in an adult male. IDCases. 2023;31:e01661.

[413] Kimball SA. Complete heart block as the sole presentation of Lyme disease. Archives of Internal Medicine. 1989;149(8):1897-8.

[414] King LA, Goirand L, Tissot-Dupont H, Giunta B, Giraud C, Colardelle C, et al. Outbreak of Q fever, Florac, Southern France, Spring 2007. Vector-Borne and Zoonotic Diseases. 2011;11(4):341-7.

[415] Kirmizis D, Efstratiadis G, Economidou D, Diza-Mataftsi E, Leontsini M, Memmos D. MPGN secondary to Lyme disease. Am J Kidney Dis. 2004;43(3):544-51.

[416] Kirsch M. Fatal adult respiratory distress syndrome in a patient with Lyme disease. JAMA: The Journal of the American Medical Association. 1988;259(18):2737-9.

[417] Knust B, Medetov ZB, Kyraubayev KB, Bumburidi Y, Erickson BR, MacNeil A, et al. Crimean-Congo Hemorrhagic Fever, Kazakhstan, 2009-2010. Emerging Infectious Diseases. 2012;18(4):643-5.

[418] Koebel C, Kern A, Edouard S, Anh Thu H, Celestin N, Hansmann Y, et al. Human granulocytic anaplasmosis in eastern France: clinical presentation and laboratory diagnosis. Diagnostic Microbiology and Infectious Disease. 2012;72(3):214-8.

[419] Koester TM, Meece JK, Fritsche TR, Frost HM. Infectious Mononucleosis and Lyme Disease as Confounding Diagnoses: A Report of 2 Cases. Clin Med Res. 2018;16(3-4):66-8.

[420] Koester TM, Timothy P, Meece JK, Osborn RA, Frost HM. Suspected Neuro-invasive Powassan Virus Infection in a Pediatric Patient. Clin Med Res. 2020;18(2-3):95-8.

[421] Kofteridis DP, Mazokopakis EE, Tselentis Y, Gikas A. Neurological complications of acute Q fever infection. European Journal of Epidemiology. 2004;19(11):1051-4.

[422] Koksal I, Yilmaz G, Aksoy F, Erensoy S, Aydin H. The seroprevalance of Crimean-Congo haemorrhagic fever in people living in the same environment with Crimean-Congo haemorrhagic fever patients in an endemic region in Turkey. Epidemiology and Infection. 2014;142(2):239-45.

[423] Koksal I, Yilmaz G, Iskender S, Arslan M, Yavuz I, Aksoy F, et al. The First Crimean-Congo Hemorrhagic Fever Case in the Winter Season from Turkey. Intervirology. 2011;54(3):144-5.

[424] Komitova R, Lakos A, Aleksandrov A, Christova I, Murdjeva M. A case of tick-transmitted

lymphadenopathy in Bulgaria associated with Rickettsia slovaca. Scandinavian Journal of Infectious Diseases. 2003;35(3):213-.

[425] Korkmaz S, Elaldi N, Kayatas M, Sencan M, Yildiz E. Unusual manifestations of acute Q fever: autoimmune hemolytic anemia and tubulointerstitial nephritis. Ann Clin Microbiol Antimicrob. 2012;11:14.

[426] Kornmehl EW, Lesser RL, Jaros P, Rocco E, Steere AC. Bilateral Keratitis in Lyme Disease. Ophthalmology. 1989;96(8):1194-7.

[427] Koster FT, Williams JC, Goodwin JS. Cellular Immunity in Q Fever: Specific Lymphocyte Unresponsiveness in Q Fever Endocarditis. Journal of Infectious Diseases. 1985;152(6):1283-9.

[428] Kostopoulou V, Chochlakis D, Kanta C, Katsanou A, Rossiou K, Rammos A, et al. A Case of Human Infection by Rickettsia slovaca in Greece. Japanese Journal of Infectious Diseases. 2016;69(4):335-7.

[429] Kounatidis D, Skourtis A, Grivakou E, Vourlakou C, Geladari E, Jahaj E, et al. Multiple mononeuritis with peripheral blood eosinophilia in a patient with Q fever: An unusual presentation: Case report and review of the literature. Journal of Infection and Chemotherapy. 2021;27(9):1357-9.

[430] Kováčová E, Sixl W, Stünzner D, Ürvögyi J, Kazár J. Serological examination of human and animal sera from six countries of three continents for the presence of rickettsial antibodies. European Journal of Epidemiology. 1996;12(1):85-9.

[431] Kowalski TJ, Tata S, Berth W, Mathiason MA, Agger WA. Antibiotic Treatment Duration and Long-Term Outcomes of Patients with Early Lyme Disease from a Lyme Disease-Hyperendemic Area. Clinical Infectious Diseases. 2010;50(4):512-20.

[432] Krause PJ, Carroll M, Fedorova N, Brancato J, Dumouchel C, Akosa F, et al. Human Borrelia miyamotoi infection in California: Serodiagnosis is complicated by multiple endemic Borrelia species. PLoS ONE. 2018;13(2).

[433] Krause PJ, Corrow CL, Bakken JS. Successful Treatment of Human Granulocytic Ehrlichiosis in Children Using Rifampin. Pediatrics. 2003;112(3):e252-e3.

[434] Krause PJ, Narasimhan S, Wormser GP, Barbour AG, Platonov AE, Brancato J, et al. Borrelia miyamotoi sensu lato Seroreactivity and Seroprevalence in the Northeastern United States. Emerging Infectious Diseases. 2014;20(7):1183-90.

[435] Krause PJ, Narasimhan S, Wormser GP, Rollend L, Fikrig E, Lepore T, et al. Human Borrelia miyamotoi Infection in the United States. New England Journal of Medicine. 2013;368(3):291-3.

[436] Krause PJ, Schwab J, Narasimhan S, Brancato J, Xu G, Rich SM. Hard Tick Relapsing Fever Caused by Borrelia miyamotoi in a Child. Pediatric Infectious Disease Journal. 2016;35(12):1352-4.

[437] Kreisel F. Doughnut Ring—Shaped Epithelioid Granulomas in the Bone Marrow of a Patient With Q Fever. International Journal of Surgical Pathology. 2007;15(2):172-3.

[438] Krol V, Kogan V, Cunha BA. Q fever bioprosthetic aortic valve endocarditis (PVE) successfully treated with doxycycline monotherapy. Heart Lung. 2008;37(2):157-60.

[439] Krow-Lucal ER, Lindsey NP, Fischer M, Hills SL. Powassan Virus Disease in the United States, 2006-2016. Vector-Borne and Zoonotic Diseases. 2018;18(6):286-90.

[440] Kuenen JG. Anammox bacteria: from discovery to application. Nature Reviews Microbiology. 2008;6(4):320-6.

[441] Kuloglu F, Rolain JM, Akata F, Eroglu C, Celik AD, Parola P. Mediterranean spotted fever in the Trakya region of Turkey. Ticks and Tick-Borne Diseases. 2012;3(5-6):297-303.

[442] Kuloglu F, Rolain JM, Aydoslu B, Akata F, Tugrul M, Raoult D. Prospective evaluation of rickettsioses in the Trakya (European) region of Turkey and atypic presentations of <i>Rickettsia conorii</i>. In: Hechemy KE, Oteo JA, Raoult DA, Silverman DJ, Blanco JR, editors. Century of Rickettsiology: Emerging, Reemerging Rickettsioses, Molecular Diagnostics, and Emerging Veterinary Rickettsioses. Annals of the New York Academy of Sciences. 10782006. p. 173-5.

[443] Kumar M, Singh R, Yadav M. Indian Tick Typhus Presenting with Gangrene: A Case Report from an Urban Slum of Delhi. Indian Journal of Pediatrics. 2014;81(1):95-7.

[444] Kurt YG, Cayci T, Onguru P, Akgul EO, Yaman H, Aydin I, et al. Serum chitotriosidase enzyme activity in patients with Crimean-Congo hemorrhagic fever. Clinical Chemistry and Laboratory Medicine. 2009;47(12):1543-7.

[445] Kváč M, Květoňová D, Sak B, Ditrich O. CryptosporidiumPig Genotype II in Immunocompetent Man. Emerging Infectious Diseases. 2009;15(6):982-3.

[446] Ladzinski AT, Baker M, Dunning K, Patel PP. Human Granulocytic Anaplasmosis presenting as Subacute Abdominal Pain and Hyponatremia. IDCases. 2021;25:e01183.

[447] Laidoudi Y, Rousset E, Dessimoulie A-S, Prigent M, Raptopoulo A, Huteau Q, et al. Tracking the Source of Human Q Fever from a Southern French Village: Sentinel Animals and Environmental Reservoir. Microorganisms. 2023;11(4).

[448] Landais C, Fenollar F, Constantin A, Cazorla C, Guilyardi C, Lepidi H, et al. Q fever osteoarticular infection: four new cases and a review of the literature. Eur J Clin Microbiol Infect Dis. 2007;26(5):341-7.

[449] Landais C, Fenollar F, Thuny F, Raoult D. From acute Q fever to endocarditis: Serological follow-up strategy. Clinical Infectious Diseases. 2007;44(10):1337-40.

[450] Landau Z, Feld S, Kunichezky S, Grinspan M, Gorbacz M. Thrombosis of the Mesenteric Vein as a Complication of Mediterranean Spotted Fever. Clinical Infectious Diseases. 1992;15(6):1070-1.

[451] Lane RS, Lavoie PE. Lyme borreliosis in California. Acarological, clinical, and epidemiological studies. Annals of the New York Academy of Sciences. 1988;539:192-203.

[452] Lantos PM, Branda JA, Boggan JC, Chudgar SM, Wilson EA, Ruffin F, et al. Poor Positive Predictive Value of Lyme Disease Serologic Testing in an Area of Low Disease Incidence. Clinical Infectious Diseases. 2015;61(9):1374-80.

[453] Lantos PM, Nigrovic LE, Auwaerter PG, Fowler VG, Ruffin F, Brinkerhoff RJ, et al. Geographic Expansion of Lyme Disease in the Southeastern United States, 2000–2014. Open Forum Infectious Diseases. 2015;2(4).

[454] Lee S, Khankhanian P, Salama C, Brown M, Lieber J. Pseudo-Pelger-Huët anomaly and granulocytic dysplasia associated with human granulocytic anaplasmosis. Int J Hematol. 2015;102(1):129-33.

[455] Martin GS, Christman BW, Standaert SM. Rapidly Fatal Infection withEhrlichia chaffeensis. New England Journal of Medicine. 1999;341(10):763-4.

[456] Mehdipanah R. Recensión bibliográfica. Gaceta Sanitaria. 2012;26(6).

[457] Meissner JD, Seregin SS, Seregin SV, Yakimenko NV, Vyshemirskii OI, Netesov SV, et al. Complete L segment coding-region sequences of Crimean Congo hemorrhagic fever virus strains

from the Russian Federation and Tajikistan. Archives of Virology. 2006;151(3):465-75.
[458] Miller NR. Predicting visual recovery following optic nerve decompression for chronic optic neuropathy. Acta Neurochirurgica. 2009;151(12):1729-.
[459] Mogg M, Wang H-H, Baker A, Derouen Z, Borski J, Grant WE. Increased Incidence of Ehrlichia chaffeensis Infections in the United States, 2012 Through 2016. Vector-Borne and Zoonotic Diseases. 2020;20(7):547-50.
[460] Molloy PJ, Telford SR, III, Chowdri HR, Lepore TJ, Gugliotta JL, Weeks KE, et al. Borrelia miyamotoi Disease in the Northeastern United States A Case Series. Annals of Internal Medicine. 2015;163(2):91-+.
[461] Mowla SJ, Drexler NA, Cherry CC, Annambholta PD, Kracalik IT, Basavaraju SV. Ehrlichiosis and Anaplasmosis among Transfusion and Transplant Recipients in the United States. Emerging Infectious Diseases. 2021;27(11):2768-75.
[462] N BD, Tasha S, J VA, Elisabeth L, Alisa D, L ST, et al. Francisella tularensis subsp. novicida isolated from a human in Arizona. BMC Research Notes. 2009;2(1).
[463] Ng CP, Chung CH. Persistent Fever and Exanthema in a Family: A Case Report of Mediterranean Spotted Fever. Hong Kong Journal of Emergency Medicine. 2017;12(2):91-4.
[464] Oizumi K, Ono R, Konno K, Numazaki Y. Isolation of cytomegalovirus from the lung tissue of patients with intrapulmonary neoplasma. The Tohoku Journal of Experimental Medicine. 1989;158(4):335-6.
[465] Parola P, Jourdan J, Raoult D. Tick-Borne Infection Caused byRickettsia africaein the West Indies. New England Journal of Medicine. 1998;338(19):1391-2.
[466] Prevention(CDC) CfDCa. Lyme Disease—United States, 1996. JAMA: The Journal of the American Medical Association. 1997;278(2).
[467] Prevention(CDC) CfDCa. From the Centers for Disease Control and Prevention. Outbreak of Powassan encephalitis--Maine and Vermont, 1999-2001. Jama-journal of The American Medical Association. 2001;286(16):1962-3.
[468] Raoult D, Tissot-Dupont H, Foucault C, Gouvernet J, Fournier PE, Bernit E, et al. Q Fever 1985-1998: Clinical and Epidemiologic Features of 1,383 Infections. Medicine. 2000;79(2):109-23.
[469] Saha. BK, Chieng. H, Itty. R, Bonnier. A, Shkolnik. B, Beegle. S. Previously Unreported Presentation of Anaplasmosis in an Endemic Area: A Report of 2 Cases. Infectious Diseases in Clinical Practice. 2020;28(1).
[470] Salehi-Vaziri M, Fazlalipour M, Baniasadi V, Pouriayevali M, Jalali T, Mohammadi T, et al. Crimean-Congo hemorrhagic fever virus Asia 2 genotype in Qeshm Island, southern Iran: A case report. Journal of Vector Borne Diseases. 2019;56(3).
[471] Samy AM, Chen X, Li F, Yin Q, Liu W, Fu S, et al. Epidemiology of tick-borne encephalitis in China, 2007- 2018. PLoS ONE. 2019;14(12).
[472] Scott H, Shihab N, Raimondi F. Canine wobbler syndrome study. Veterinary Record. 2013;173(17):428-.
[473] Seneviratne JK, Blair JE, Smith BE. Brachial plexopathy associated with Q fever: Case report and review of the literature. Muscle Nerve. 2008;38(6):1644-8.
[474] Sköldenberg B, Stiernstedt G. Lyme Borreliosis 1990: Proceedings of the IV International Conference on Lyme Borreliosis Held in Stockholm, Sweden June 18–21, 1990. Scandinavian Journal of Infectious Diseases. 2015;23(sup77):1-157.

[475] Syed MA, Siddiqui MI, Memon IH, Jehandad K, Baloch NN, Jamal H, et al. Risk Factors of Crimean-Congo Haemorrhagic Fever in Sindh Province, Pakistan. International Journal of Infectious Diseases. 2024;146.

[476] Szer IS, Taylor E, Steere AC. The Long-Term Course of Lyme Arthritis in Children. New England Journal of Medicine. 1991;325(3):159-63.

[477] Szonyi B, Srinath I, Esteve-Gassent M, Lupiani B, Ivanek R. Exploratory spatial analysis of Lyme disease in Texas -what can we learn from the reported cases? BMC Public Health. 2015;15:924.

[478] Tabassum S, Naeem A, Khan MZ, Mumtaz N, Gill S, Ohadi L. Crimean-Congo hemorrhagic fever outbreak in Pakistan, 2022: A warning bell amidst unprecedented floods and COVID 19 pandemic. Health Science Reports. 2023;6(1).

[479] Talbot TR, Comer JA, Bloch KC. Ehrlichia chaffeensisInfections among HIV-infected Patients in a Human Monocytic Ehrlichiosis–Endemic Area. Emerging Infectious Diseases. 2003;9(9):1123-7.

[480] Talsness SR, Shukla SK, Mazza JJ, Yale SH. Rhabdomyolysis-induced acute kidney injury secondary to Anaplasma phagocytophilum and concomitant statin use. Wmj : Official Publication of The State Medical Society of Wisconsin. 2011;110(2):82-4.

[481] Tande AJ, Cunningham SA, Raoult D, Sim FH, Berbari EF, Patel R. A case of Q fever prosthetic joint infection and description of an assay for detection of Coxiella burnetii. J Clin Microbiol. 2013;51(1):66-9.

[482] Tang Q, Saijo M, Zhang Y, Asiguma M, Tianshu D, Han L, et al. A patient with Crimean-Congo hemorrhagic fever serologically diagnosed by recombinant nucleoprotein-based antibody detection systems. Clin Diagn Lab Immunol. 2003;10(3):489-91.

[483] Tariq S, Niaz F, Safi Vahidy A, Qidwai M, Ishaq M, Abbasher Hussien Mohamed Ahmed K, et al. Crimean-Congo Hemorrhagic Fever (CCHF) in Pakistan: The Daunting Threat of an Outbreak as Eid-ul-Azha Approaches. Disaster Medicine and Public Health Preparedness. 2023;17.

[484] Tavora F, Burke A, Li L, Franks TJ, Virmani R. Postmortem confirmation of Lyme carditis with polymerase chain reaction. Cardiovascular Pathology. 2008;17(2):103-7.

[485] Taylor L, Condon T, Destrampe EM, Brown JA, McGavic J, Gould CV, et al. Powassan Virus Infection Likely Acquired Through Blood Transfusion Presenting as Encephalitis in a Kidney Transplant Recipient. Clin Infect Dis. 2021;72(6):1051-4.

[486] Tekin S, Barut S, Bursali A, Aydogan G, Yuce O, Demir F, et al. Seroprevalence of Crimean-Congo haemorrhagic fever (CCHF) in risk groups in Tokat Province of Turkey. African Journal of Microbiology Research. 2010;4(3):214-7.

[487] Telford SR, Lepore TJ, Snow P, Warner CK, Dawson JE. Human Granulocytic Ehrlichiosis in Massachusetts. Annals of Internal Medicine. 1995;123(4):277-9.

[488] Temocin F, Kose H, Sari T, Duygu F, Sahin RO. Seroprevalence of Crimean-Congo hemorrhagic fever among health care workers in a hospital in an endemic region of Turkey. Journal of Infection in Developing Countries. 2018;12(7):587-91.

[489] Ternovoi VA, Kurzhukov GP, Sokolov YV, Ivanov GY, Ivanisenko VA, Loktev AV, et al. Tick-Borne Encephalitis with Hemorrhagic Syndrome, Novosibirsk Region, Russia, 1999. Emerging Infectious Diseases. 2003;9(6):743-6.

[490] Ternovoi VA, Protopopova EV, Chausov EV, Novikov DV, Leonova GN, Netesov SV, et al. Novel Variant of Tickborne Encephalitis Virus, Russia. Emerging Infectious Diseases.

2007;13(10):1574-8.
[491] Tevatia P, Ahmad S, Gupta N, Shirazi N. Lyme disease in north India: a case for concern. Tropical Doctor. 2018;48(4):352-5.
[492] Tezer H, Sucakli IA, Sayli TR, Celikel E, Yakut I, Kara A, et al. Crimean-Congo hemorrhagic fever in children. Journal of Clinical Virology. 2010;48(3):184-6.
[493] Tezer H, Tavil B, Sucakli IA, Korukluoglu G, Uyar Y, Dincer E, et al. Concurrent Crimean-Congo Hemorrhagic Fever and Visceral Leishmaniasis in a Turkish Girl. Vector-Borne and Zoonotic Diseases. 2011;11(6):743-5.
[494] Thibon M, Villiers V, Souque P, Dautry-Varsat A, Duquesnel R, Ojcius DM. High incidence of Coxiella burnetii markers in a rural population in France. European Journal of Epidemiology. 1996;12(5):509-13.
[495] Thill P, Eldin C, Dahuron L, Berlioz-Artaud A, Demar M, Nacher M, et al. High endemicity of Q fever in French Guiana: A cross sectional study (2007-2017). Plos Neglected Tropical Diseases. 2022;16(5).
[496] Thomas LD, Hongo I, Bloch KC, Tang YW, Dummer S. Human ehrlichiosis in transplant recipients. American Journal of Transplantation. 2007;7(6):1641-7.
[497] Thomas R, Mathew F, Louis EM, Valsan C, Priyanka R, Thomas J, et al. Contact Tracing for an Imported Case of Crimean-Congo Hemorrhagic Fever - Experience from a Tertiary Care Center in Kerala, South India. Indian Journal of Community Medicine. 2019;44(3):285-7.
[498] Tissot-Dupont H, Amadei M-A, Nezri M, Raoult D. Wind in November, Q Fever in December. Emerging Infectious Diseases. 2004;10(7):1264-9.
[499] Tissot-Dupont H, Torres S, Nezri M, Raoult D. Hyperendemic Focus of Q Fever Related to Sheep and Wind. American Journal of Epidemiology. 1999;150(1):67-74.
[500] Tissot-Dupont H, Vaillant V, Rey S, Raoult D. Role of Sex, Age, Previous Valve Lesion, and Pregnancy in the Clinical Expression and Outcome of Q Fever after a Large Outbreak. Clinical Infectious Diseases. 2007;44(2):232-7.
[501] Titenko AM, Andaev EI, Botvinkin AD. Tick-borne encephalitis epidemiology in some provinces of Siberia. Zentralblatt für Bakteriologie. 1999;289(5-7):595-604.
[502] Todd CS, Mansoor GF, Buhler C, Rahimi H, Zekria R, Fernandez S, et al. Prevalence of Zoonotic and Vector-Borne Infections Among Afghan National Army Recruits in Afghanistan. Vector-Borne and Zoonotic Diseases. 2016;16(8):501-6.
[503] Tonnetti L, Marcos LA, Mamone L, Spitzer ED, Jacob M, Townsend RL, et al. A case of transfusion-transmission Anaplasma phagocytophilum from leukoreduced red blood cells. Transfusion. 2024;64(4):751-4.
[504] Townsend RL, Moritz ED, Fialkow LB, Berardi V, Stramer SL. Probable transfusion-transmission of Anaplasma phagocytophilum by leukoreduced platelets. Transfusion. 2014;54(11):2828-32.
[505] Treadwell TL. Mediterranean Spotted Fever in Children Returning From France. Arch Pediatr Adolesc Med. 1990;144(9).
[506] Tripathi S, Bhati R, Gopalakrishnan M, Bohra GK, Tiwari S, Panda S, et al. Clinical profile and outcome of patients with Crimean Congo haemorrhagic fever: a hospital based observational study from Rajasthan, India. Transactions of the Royal Society of Tropical Medicine and Hygiene. 2020;114(9):650-6.
[507] Tsai K-H, Chung L-H, Chien C-H, Tung Y-J, Wei H-Y, Yen T-Y, et al. Human granulocytic

anaplasmosis in Kinmen, an offshore island of Taiwan. Plos Neglected Tropical Diseases. 2019;13(9).

[508] Tselentis Y, Gikas A, Kofteridis D, Kyriakakis E, Lydataki N, Bouros D, et al. Q Fever in the Greek Island of Crete: Epidemiologic, Clinical, and Therapeutic Data from 98 Cases. Clinical Infectious Diseases. 1995;20(5):1311-6.

[509] Tsukahara T, Ezaki T, Moriguchi J, Furuki K, Ukai H, Okamoto S, et al. Effects of Iron-Deficiency Anemia on Cadmium Uptake or Kidney Dysfunction Are Essentially Nil among Women in General Population in Japan. The Tohoku Journal of Experimental Medicine. 2002;197(4):243-7.

[510] Tufan ZK, Yigit H, Kacar M, Bulut C, Canpolat G, Hatipoglu CA, et al. Sonographic Findings in Patients With Crimean-Congo Hemorrhagic Fever. Journal of Ultrasound in Medicine. 2014;33(11):1999-2003.

[511] Tumturk A. Crimean-Congo haemorrhagic fever in a middle Anatolian city: five years of experience. Tropical Doctor. 2020;50(2):115-7.

[512] Ture Z, Kilic AU, Celik I, Tok T, Yagci-Caglayik D. Crimean-Congo hemorrhagic fever with hyperbilirubinemia and ascites: An unusual presentation. Journal of Medical Virology. 2016;88(1):159-62.

[513] Turtle L, Wey E, James E. Severe pneumonia caused by ciprofloxacin resistant panton-valentine leukocidin producing community acquired meticillin resistant Staphylococcus aureus. Journal of Infection. 2009;58(1):86-7.

[514] Tuygun N, Tanir G, Caglayik DY, Uyar Y, Korukluoglu G, Cenesiz F. Pediatric cases of Crimean-Congo hemorrhagic fever in Turkey. Pediatrics International. 2012;54(3):402-6.

[515] Twizeyimana E, Pichard E, Lunel-Fabiani F, Fanello S, De Martino SJ. Impact of serodiagnosis on the management of Lyme borreliosis at Angers University Hospital. Medecine Et Maladies Infectieuses. 2014;44(9):429-32.

[516] Tzavella K, Hatzizisis IS, Vakali A, Mandraveli K, Zioutas D, Alexiou-Daniel S. Severe case of Mediterranean spotted fever in Greece with predominantly neurological features. Journal of Medical Microbiology. 2006;55(3):341-3.

[517] Umair M, Khurshid A, Alam MM, Akhtar R, Salman M, Ikram A. Genetic diversity and phylogenetic analysis of Crimean-Congo Hemorrhagic Fever viruses circulating in Pakistan during 2019. Plos Neglected Tropical Diseases. 2020;14(6).

[518] Umair M, Rehman Z, Haider SA, Ali Q, Hakim R, Bibi S, et al. Whole-genome sequencing of Crimean-Congo hemorrhagic fever virus circulating in Pakistan during 2022. Journal of Medical Virology. 2023;95(3).

[519] Umair M, Rehman Z, Whitmer S, Mobley M, Fahim A, Ikram A, et al. Crimean-Congo Hemorrhagic Fever Virus Diversity and Reassortment, Pakistan, 2017-2020. Emerging Infectious Diseases. 2024;30(4):654-64.

[520] Ungar SP, Varkey J, Pierro J, Raetz E, Ratner AJ. Do Not Forget About the Ticks: An Unusual Cause of Fever, GI Distress, and Cytopenias in a Child With ALL. Journal of Pediatric Hematology/Oncology. 2022;44(5):e901-e4.

[521] Vahey GM, Mathis S, Martin SW, Gould CV, Staples JE, Lindsey NP. West Nile Virus and Other Domestic Nationally Notifiable Arboviral Diseases — United States, 2019. MMWR Morbidity and Mortality Weekly Report. 2021;70(32):1069-74.

[522] Vandenesch A, Turbelin C, Couturier E, Arena C, Jaulhac B, Ferquel E, et al. Incidence and hospitalisation rates of Lyme borreliosis, France, 2004 to 2012. Eurosurveillance. 2014;19(34).

[523] Vaughn MF, Delisle J, Johnson J, Daves G, Williams C, Reber J, et al. Seroepidemiologic Study of Human Infections with Spotted Fever Group Rickettsiae in North Carolina. Journal of Clinical Microbiology. 2014;52(11):3960-6.

[524] Vaziri S, Navabi J, Afsharian M, Sayad B, Mansouri F, Janbakhsh A, et al. Crimean congo hemorrhagic fever infection simulating thrombotic thrombocytopenic purpura. Indian Journal of Hematology and Blood Transfusion. 2008;24(1):35-8.

[525] Vázquez M, Sparrow SS, Shapiro ED. Long-Term Neuropsychologic and Health Outcomes of Children With Facial Nerve Palsy Attributable to Lyme Disease. Pediatrics. 2003;112(2):e93-e7.

[526] Velay A, Janssen-Langenstein R, Kremer S, Laugel E, Lutz M, Pierson AL, et al. Tick-Borne Encephalitis in Pregnant Woman and Long-Term Sequelae. Emerging Infectious Diseases. 2023;29(3):669-71.

[527] Velay A, Solis M, Kack-Kack W, Gantner P, Maquart M, Martinot M, et al. A new hot spot for tick-borne encephalitis (TBE): A marked increase of TBE cases in France in 2016. Ticks and Tick-Borne Diseases. 2018;9(1):120-5.

[528] Vinayaraj EV, Gupta N, Sreenath K, Thakur CK, Gulati S, Anand V, et al. Clinical and laboratory evidence of Lyme disease in North India, 2016-2019. Travel Medicine and Infectious Disease. 2021;43.

[529] Vinayaraj EV, Thakur CK, Negi P, Sreenath K, Upadhyay P, Verma N, et al. Epidemiological, clinical, and laboratory characteristics of human granulocytic anaplasmosis in North India. Journal of Clinical Microbiology. 2024;62(3).

[530] Volchkova E, Umbetova K, Karan L, Konnova Y, Gorobchenko A, Belaia O, et al. Clinical case of spotted fever group rickettsiae. Int J Infect Dis. 2019;89:27-9.

[531] Volynkina A, Lisitskaya Y, Kolosov A, Shaposhnikova L, Pisarenko S, Dedkov V, et al. Molecular epidemiology of Crimean-Congo hemorrhagic fever virus in Russia. PLoS ONE. 2022;17(5).

[532] Vranakis I, Kokkini S, Chochlakis D, Sandalakis V, Pasparaki E, Minadakis G, et al. Serological survey of Q fever in Crete, southern Greece. Comparative Immunology Microbiology and Infectious Diseases. 2012;35(2):123-7.

[533] Vranakis I, Kokkini S, Yachnakis E, Tselentis Y, Chochlakis D, Psaroulaki A. Q fever in Greece: Findings of a 13 years surveillance study. Comparative Immunology Microbiology and Infectious Diseases. 2020;69.

[534] Vural T, Ergin Ç, Sayin F. Investigation ofRickettsia conorii antibodies in the Antalya area. Infection. 1998;26(3):170-2.

[535] Wald M, Merisor S, Zachary P, Augereau O, Gravier S, Jaulhac B, et al. Microbiological Outcomes Associated With Low Leukocyte Counts in Cerebrospinal Fluid. Open Forum Infectious Diseases. 2021;8(2).

[536] Wallace JW, Nicholson WL, Perniciaro JL, Vaughn MF, Funkhouser S, Juliano JJ, et al. Incident Tick-Borne Infections in a Cohort of North Carolina Outdoor Workers. Vector-Borne and Zoonotic Diseases. 2016;16(5):302-8.

[537] Walsh MG. Assessing Q fever in a representative sample from the United States population: identification of a potential occupational hazard. Epidemiology and Infection. 2012;140(1):42-6.

[538] Wang D, Zhang L, Cai Z, Liu Y. Diagnosis of Acute Q Fever in a Patient by Using Metagenomic

Next-Generation Sequencing: A Case Report. Infection and Drug Resistance. 2023;16:1923-30.
[539] Wang F, Ma M, Luo S, Yan M, Tao L, Liu A, et al. Seroprevalence of Tick-Borne Anaplasma phagocytophilum Infection in Healthy Adult Population and Patients with Acute Undifferentiated Fever from the Yunnan Province of China. Vector-Borne and Zoonotic Diseases. 2019;19(8):576-81.
[540] Wang TJ, Liang MH, Sangha O, Phillips CB, Lew RA, Wright EA, et al. Coexposure to Borrelia burgdorferi and Babesia microti Does Not Worsen the Long-Term Outcome of Lyme Disease. Clinical Infectious Diseases. 2000;31(5):1149-54.
[541] Wang W, Chen O, Liu W, Gan L, Li X, Ma Q, et al. Coxiella burnetii and Bartonella Endocarditis Diagnosed by Metagenomic Next-Generation Sequencing. J Clin Med. 2022;11(23).
[542] Wang X, Zou G, Wang Q, Li J. A case report of Vancomycin in the treatment of Q fever endocarditis. Frontiers in Cellular and Infection Microbiology. 2024;14.
[543] Warner RD, Jemelka ED, Jessen AE. An outbreak of tick-bite-associated illness among military personnel subsequent to a field training exercise. Journal of the American Veterinary Medical Association. 1996;209(1):78-81.
[544] Watt G, Lacroix A, Pachirat O, Baggett HC, Raoult D, Fournier P-E, et al. Prospective Comparison of Infective Endocarditis in Khon Kaen, Thailand and Rennes, France. American Journal of Tropical Medicine and Hygiene. 2015;92(4):871-4.
[545] Webber BJ, Burganowski RP, Colton L, Escobar JD, Pathak SR, Gambino-Shirley KJ. Lyme disease overdiagnosis in a large healthcare system: a population-based, retrospective study. Clinical Microbiology and Infection. 2019;25(10):1233-8.
[546] Weinberger M, Keysary A, Sandbank J, Zaidenstein R, Itzhaki A, Strenger C, et al. FatalRickettsia conoriisubsp.israelensisInfection, Israel. Emerging Infectious Diseases. 2008;14(5):821-4.
[547] Weinstein ER, Rebman AW, Aucott JN, Johnson-Greene D, Bechtold KT. Sleep quality in well-defined Lyme disease: a clinical cohort study in Maryland. Sleep. 2018;41(5).
[548] Weiss NL, Sadock VA, Sigal LH, Phillips M, Merryman PF, Abramson SB. False positive seroreactivity to Borrelia burgdorferi in systemic lupus erythematosus: the value of immunoblot analysis. Lupus. 1995;4(2):131-7.
[549] Weiss T, Zhu P, White H, Posner M, Wickiser JK, Washington MA, et al. Latent Lyme Disease Resulting in Chronic Arthritis and Early Career Termination in a United States Army Officer. Military Medicine. 2019;184(7-8):E368-E70.
[550] Weitzner E, Visintainer P, Wormser GP. Comparison of males versus females with culture-confirmed early Lyme disease at presentation and at 11-20 years after diagnosis. Diagnostic Microbiology and Infectious Disease. 2016;85(4):493-5.
[551] Wendling D, Sevrin P, Bouchaud-Chabot A, Chabroux A, Toussirot E, Bardin T, et al. Parsonage-Turner syndrome revealing Lyme borreliosis. Joint Bone Spine. 2009;76(2):202-4.
[552] Wenjun LI, Mouffok N, Rovery C, Parola P, Raoult D. Genotyping Rickettsia conorii detected in patients with Mediterranean spotted fever in Algeria using multispacer typing (MST). Clinical Microbiology and Infection. 2009;15:281-3.
[553] White B, Brooks T, Seaton RA. Q fever in military and paramilitary personnel in conflict zones: Case report and review. Travel Medicine and Infectious Disease. 2013;11(2):134-7.
[554] White DJ. The Geographic Spread and Temporal Increase of the Lyme Disease Epidemic. JAMA: The Journal of the American Medical Association. 1991;266(9).

[555] Whitney EAS, Massung RF, Candee AJ, Ailes EC, Myers LM, Patterson NE, et al. Seroepidemiologic and Occupational Risk Survey for Coxiella burnetii Antibodies among US Veterinarians. Clinical Infectious Diseases. 2009;48(5):550-7.

[556] Wiberg K, Birnbaum A, Gradon J. Causes and Presentation of Meningitis in a Baltimore Community Hospital 1997–2006. Southern Medical Journal. 2008;101(10):1012-6.

[557] Wiley Z, Reddy S, Jacobs Slifka KM, Brandon DC, Jernigan J, Kersh GJ, et al. Chronic Q Fever with Vascular Involvement: Progressive Abdominal Pain in a Patient with Aortic Aneurysm Repair in the United States. Case Rep Infect Dis. 2019;2019:5369707.

[558] Williams CL, Curran AS, Lee AC, Sousa VO. Lyme disease: epidemiologic characteristics of an outbreak in Westchester County, NY. American Journal of Public Health. 1986;76(1):62-5.

[559] Wills AB, Spaulding AB, Adjemian J, Prevots DR, Turk S-P, Williams C, et al. Long-term Follow-up of Patients With Lyme Disease: Longitudinal Analysis of Clinical and Quality-of-life Measures. Clinical Infectious Diseases. 2016;62(12):1546-51.

[560] Wilske B, Schierz G, Preac-Mursic V, von Buscb K, Kuhbeck R, Pfister HW, et al. Intrathecal Production of Specific Antibodies against Borrelia burgdorferi in Patients with Lymphocytic Meningoradiculitis (Bannwarth's Syndrome). Journal of Infectious Diseases. 1986;153(2):304-14.

[561] Winward KE, Smith JL, Culbertson WW, Parishamelin A. Ocular Lyme borreliosis. American Journal of Ophthalmology. 1989;108(6):651-7.

[562] Wittwer B, Pelletier S, Ducrocq X, Maillard L, Mione G, Richard S. Cerebrovascular Events in Lyme Neuroborreliosis. Journal of Stroke & Cerebrovascular Diseases. 2015;24(7):1671-8.

[563] Wong SJ, Brady GS, Dumler JS. Serological responses to Ehrlichia equi, Ehrlichia chaffeensis, and Borrelia burgdorferi in patients from New York State. Journal of Clinical Microbiology. 1997;35(9):2198-205.

[564] Wormser GP. Use of a novel technique of cutaneous lavage for diagnosis of Lyme disease associated with erythema migrans. JAMA: The Journal of the American Medical Association. 1992;268(10):1311-3.

[565] Wormser GP, Aguero-Rosenfeld ME, Cox ME, Nowakowski J, Nadelman RB, Holmgren D, et al. Differences and Similarities between Culture-Confirmed Human Granulocytic Anaplasmosis and Early Lyme Disease. Journal of Clinical Microbiology. 2013;51(3):954-8.

[566] Wormser GP, Brady KC, Cho MS, Scavarda CA, McKenna D. Efficacy of a 14-day course of amoxicillin for patients with erythema migrans. Diagnostic Microbiology and Infectious Disease. 2019;94(2):192-4.

[567] Wormser GP, Horowitz HW, Nowakowski J, McKenna D, Stephen Dumler J, Varde S, et al. Positive Lyme Disease Serology in Patients With Clinical and Laboratory Evidence of Human Granulocytic Ehrlichiosis. American Journal of Clinical Pathology. 1997;107(2):142-7.

[568] Wormser Gary P, Liveris D, Nowakowski J, Nadelman Robert B, Cavaliere LF, McKenna D, et al. Association of Specific Subtypes ofBorrelia burgdorferiwith Hematogenous Dissemination in Early Lyme Disease. The Journal of Infectious Diseases. 1999;180(3):720-5.

[569] Wormser GP, McKenna D, Carlin J, Nadelman RB, Cavaliere LF, Holmgren D, et al. Brief Communication: Hematogenous Dissemination in Early Lyme Disease. Annals of Internal Medicine. 2005;142(9):751-5.

[570] Wormser GP, McKenna D, Jacobson E, Shanker EM, Shaffer KD, Scavarda C, et al. Patients with

Erythema Migrans: Characterizing the Impact of Initiation of Antibiotic Therapy Prior to Study Enrollment. Antimicrobial Agents and Chemotherapy. 2021;65(1).

[571] Wormser GP, McKenna D, Morgan T, Scavarda C, Cooper D, Visintainer P. A Prospective Study to Characterize Symptoms and Symptom Severity in Adult Patients with Extracutaneous Manifestations of Lyme Disease. American Journal of Medicine. 2023;136(7):702-6.

[572] Wormser GP, McKenna D, Shaffer KD, Silverman JH, Scavarda C, Visintainer P. Evaluation of selected variables to determine if any had predictive value for, or correlated with, residual symptoms at approximately 12 months after diagnosis and treatment of early Lyme disease. Diagnostic Microbiology and Infectious Disease. 2021;100(3).

[573] Wormser GP, Ramanathan R, Nowakowski J, McKenna D, Holmgren D, Visintainer P, et al. Duration of Antibiotic Therapy for Early Lyme Disease. Annals of Internal Medicine. 2003;138(9):697-704.

[574] Wormser GP, Schneider LM, Joseph JT, Fish D. Surveillance data from 2011-2020 indicate a lower risk of Lyme disease in the USA in even-numbered years. Wiener Klinische Wochenschrift. 2024;136(21-22):636-8.

[575] Wormser GP, Sudhindra P, Lopez E, Patel L, Rezai S, Brumbaugh AD, et al. Fatigue in patients with erythema migrans. Diagn Microbiol Infect Dis. 2016;86(3):322-6.

[576] Wormser GP, Weitzner E, McKenna D, Nadelman RB, Scavarda C, Molla I, et al. Long-term Assessment of Health-Related Quality of Life in Patients With Culture-Confirmed Early Lyme Disease. Clinical Infectious Diseases. 2015;61(2):244-7.

[577] Wright WF, Oliverio JA. First Case of Lyme Arthritis Involving a Prosthetic Knee Joint. Open Forum Infect Dis. 2016;3(2):ofw096.

[578] Wroblewski D, Gebhardt L, Prusinski MA, Meehan LJ, Halse TA, Musser KA. Detection of Borrelia miyamotoi and other tick-borne pathogens in human clinical specimens and Ixodes scapularis ticks in New York State, 2012-2015. Ticks and Tick-Borne Diseases. 2017;8(3):407-11.

[579] Wu CS, Chang KY, Lee CS, Chen TJ. Acute Q fever hepatitis in Taiwan. J Gastroenterol Hepatol. 1995;10(1):112-5.

[580] Wu Z, Li Y, Gu J, Zheng H, Tong Y, Wu Q. Detection of viruses and atypical bacteria associated with acute respiratory infection of children in Hubei, China. Respirology. 2014;19(2):218-24.

[581] Xanthos. T, Lelovas. P, Kantsos. H, Dontas. I, Perrea. D, Kouskouni. E. Lyme carditis: complete atrioventricular dissociation with need for temporary pacing. Hellenic Journal of Cardiology. 2006;47(5):313-6.

[582] Xia H, Li P, Yang J, Pan L, Zhao J, Wang Z, et al. Epidemiological survey of Crimean-Congo hemorrhagic fever virus in Yunnan, China, 2008. International Journal of Infectious Diseases. 2011;15(7):E459-E63.

[583] Xie CB, Cowper S, Odell ID. Morphea after Borrelia-induced facial nerve palsy. J Scleroderma Relat Disord. 2021;6(1):111-3.

[584] Xing F, Ye H, Deng C, Sun L, Yuan Y, Lu Q, et al. Diverse and atypical manifestations of Q fever in a metropolitan city hospital: Emerging role of next-generation sequencing for laboratory diagnosis of <i>Coxiella burnetii</i>. Plos Neglected Tropical Diseases. 2022;16(4).

[585] Xu N, Gai W, Zhang Y, Wang W, Wang G, Dasch GA, et al. Confirmation of Rickettsia conorii Subspecies indica Infection by Next-Generation Sequencing, Shandong, China. Emerging

Infectious Diseases. 2021;27(10):2691-4.
[586] Xu N, Liu H, Qu C, Wen S, Zou W, Chang C, et al. The presence of foci of Rickettsia conorii infection in China. Infectious Medicine. 2023;2(4):334-7.
[587] Xuefei D, Qin H, Xiaodi G, Zhen G, Wei L, Xuexia H, et al. Epidemiological and Clinical Features of Three Clustered Cases Co-Infected with Lyme Disease and Rickettsioses. Zoonoses and Public Health. 2013;60(7):487-93.
[588] Yadav JP, Malik SVS, Dhaka P, Kumar A, Kumar M, Bhoomika S, et al. Coxiella burnetii in cattle and their human contacts in a gaushala (cattle shelter) from India and its partial com 1 gene sequence-based phylogenetic analysis. Animal Biotechnology. 2022;33(7):1449-58.
[589] Yadav PD, Cherian SS, Zawar D, Kokate P, Gunjikar R, Jadhav S, et al. Genetic characterization and molecular clock analyses of the Crimean-Congo hemorrhagic fever virus from human and ticks in India, 2010-2011. Infection Genetics and Evolution. 2013;14:223-31.
[590] Yadav PD, Gurav YK, Mistry M, Shete AM, Sarkale P, Deoshatwar AR, et al. Emergence of Crimean-Congo hemorrhagic fever in Amreli District of Gujarat State, India, June to July 2013. International Journal of Infectious Diseases. 2014;18:97-100.
[591] Yadav PD, Pardeshi PG, Patil DY, Shete AM, Mourya DT. Persistence of IgG antibodies in survivors of Crimean Congo hemorrhagic fever virus infection, India. J Infect Public Health. 2019;12(4):598-9.
[592] Yadav PD, Patil DY, Mourya DT. Positivity of dengue and chikungunya among Crimean-Congo hemorrhagic fever-negative cases in India: 2013-2016. J Infect Public Health. 2018;11(6):900-1.
[593] Yadav PD, Patil DY, Shete AM, Kokate P, Goyal P, Jadhav S, et al. Nosocomial infection of CCHF among health care workers in Rajasthan, India. Bmc Infectious Diseases. 2016;16.
[594] Yadav PD, Raut CG, Mourya DT. Re-occurrence of Crimean-Congo haemorrhagic fever in Ahmedabad, Gujarat, India (2012): a fatal case report. Indian Journal of Medical Research. 2013;138(6):1027-8.
[595] Yadav PD, Thacker S, Patil DY, Jain R, Mourya DT. Crimean-Congo Hemorrhagic Fever in Migrant Worker Returning from Oman to India, 2016. Emerging Infectious Diseases. 2017;23(6):1005-8.
[596] Yagci-Caglayik D, Kayaaslan B, Yapar D, Kocagul-Celikbas A, Ozkaya-Parlakay A, Emek M, et al. Monitoring Crimean-Congo haemorrhagic fever virus RNA shedding in body secretions and serological status in hospitalised patients, Turkey, 2015. Eurosurveillance. 2020;25(10):37-43.
[597] Yagci-Caglayik D, Korukluoglu G, Uyar Y. Seroprevalence and Risk Factors of Crimean-Congo Hemorrhagic Fever in Selected Seven Provinces in Turkey. Journal of Medical Virology. 2014;86(2):306-14.
[598] Yaghmaie F, Esmaeili S, Francis SA, Mostafavi E. Q fever endocarditis in Iran: A case report. Journal of Infection and Public Health. 2015;8(5):498-501.
[599] Yagupsky P. Mortality in Serologically Unconfirmed Mediterranean Spotted Fever. The Journal of Infectious Diseases. 2000;181(2):809-10.
[600] Yahşi A. Severe pneumonia in a child caused by Coxiella burnetii. J Paediatr Child Health. 2023;59(6):840-2.
[601] Yaldiz M, Erdem T, Dilek FH. Three cases of early-stage localised Lyme disease. Hong Kong Med J. 2017;23(2):204-6.
[602] Yang S, Xue B, Hu X, Zhou W, Zhang M, Zhao M. Spinal infection caused by Coxiella burnetii.

Bmc Infectious Diseases. 2023;23(1).
[603] Yang Y, Shi Q, Jin Q, Yang Z, Li W, Han J, et al. Case Report: Metagenomic Next-Generation Sequencing Clinches the Diagnosis of Acute Q Fever and Verified by Indirect Immunofluorescence Assay. Frontiers in Medicine. 2022;9.
[604] Yao M, Liu Z, Aijun W. Accurate and approximate evaluations of asynchronous tunable-wavelength-converter sharing schemes in optical burst-switched networks. Journal of Lightwave Technology. 2005;23(10):2807-15.
[605] Yapar D, Akdoğan Ö, Boyacı H, Yılmaz YA, Topçu H, Arslan S, et al. COVID-19 and Crimean-Congo Hemorrhagic Fever: Is there any Similarity in Chest Radiology? Infect Dis Clin Microbiol. 2022;4(1):1-6.
[606] Yaqub T, Oneeb M, Mukhtar N, Tahir Z, Shahid F, Subhan S, et al. Crimean-Congo Haemorrhagic Fever: Case study analysis of a sporadic outbreak from Chakwal, Pakistan. Zoonoses and Public Health. 2019;66(7):871-3.
[607] Yashina L, Vyshemirskii O, Seregin S, Petrova I, Samokhvalov E, Lvov D, et al. Genetic analysis of Crimean-Congo hemorrhagic fever virus in Russia. Journal of Clinical Microbiology. 2003;41(2):860-2.
[608] Yesilbag Z, Karadeniz A, Koculu S, Kayhan CB. Epidemiological characteristics, clinical and laboratory findings supporting preliminary diagnosis of Crimean-Congo hemorrhagic fever in an endemic region in Turkey. Wiener Klinische Wochenschrift. 2020;132(19-20):581-8.
[609] Yevich SJ, Sanchez JL, DeFraites RF, Rives CC, Dawson JE, Uhaa IJ, et al. Seroepidemiology of Infections Due to Spotted Fever Group Rickettsiae and Ehrlichia Species in Military Personnel Exposed in Areas of the United States where Such Infections Are Endemic. Journal of Infectious Diseases. 1995;171(5):1266-73.
[610] Yildirmak T, Tulek N, Bulut C. Crimean-Congo haemorrhagic fever: transmission to visitors and healthcare workers. Infection. 2016;44(5):687-9.
[611] Yilmaz G, Koksal I, Topbas M, Yilmaz H, Aksoy F. The effectiveness of routine laboratory findings in determining disease severity in patients with Crimean-Congo hemorrhagic fever: Severity prediction criteria. Journal of Clinical Virology. 2010;47(4):361-5.
[612] Yilmaz G, Mentese A, Kaya S, Uzun A, Karahan SC, Koksal I. The diagnostic and prognostic significance of soluble urokinase plasminogen activator receptor in Crimean-Congo hemorrhagic fever. Journal of Clinical Virology. 2011;50(3):209-11.
[613] Yilmaz G, Mentese A, Yilmaz H, Koksal I. Importance of Serum Adipokine and Ghrelin Levels in Patients With Crimean-Congo Hemorrhagic Fever. Journal of Medical Virology. 2015;87(2):310-4.
[614] Yilmaz G, Yilmaz H, Arslan M, Kostakoğlu U, Menteşe A, Karahan SC, et al. The prognostic significance of serum TGF-β1 levels in patients with Crimean-Congo hemorrhagic fever. J Med Virol. 2017;89(3):413-6.
[615] Yilmaz H, Barut K, Karakullukcu A, Kasapcopur O, Kocazeybek B, Altan E, et al. Serological Evidence of Tick-Borne Encephalitis and West Nile Virus Infections Among Children with Arthritis in Turkey. Vector-Borne and Zoonotic Diseases. 2019;19(6):446-9.
[616] Yilmaz H, Kostakoglu U, Demir S, Aksoy F, Mentese A, Karahan SC, et al. Carbonic anhydrase I-II autoantibodies and oxidative status in long-term follow-up of patients with Crimean-Congo haemorrhagic fever. Archives of Physiology and Biochemistry. 2018;124(1):69-74.

[617] Yilmaz H, Yilmaz G, Kostakoğlu U, Yaman H, Örem A, Köksal İ. The prognostic significance of serum troponin T levels in Crimean-Congo hemorrhagic fever patients. J Med Virol. 2017;89(3):408-12.
[618] Yilmaz H, Yilmaz G, Menteşe A, Kostakoğlu U, Karahan SC, Köksal İ. Prognostic impact of platelet distribution width in patients with Crimean-Congo hemorrhagic fever. J Med Virol. 2016;88(11):1862-6.
[619] Yilmaz M, Aydin K, Akdogan E, Sucu N, Sonmez M, Omay SB, et al. Peripheral blood natural killer cells in Crimean-Congo hemorrhagic fever. Journal of Clinical Virology. 2008;42(4):415-7.
[620] Yilmaz M, Elaldi N, Bagci B, Sari I, Gümüs E, Yelkovan I. Effect of tumour necrosis factor-alpha and interleukin-6 promoter polymorphisms on course of Crimean-Congo hemorrhagic fever in Turkish patients. Journal of Vector Borne Diseases. 2015;52(1).
[621] Yilmaz R, Karaaslan E, Albayrak SE, Gul A, Kasap T. Analysis of Pediatric Intensive Care Unit Admissions for Crimean-Congo Hemorrhagic Fever in Turkey. Journal of Pediatric Infectious Diseases. 2020;15(05):242-7.
[622] Yilmaz R, Kundak AA, Ozer S, Esmeray H. Successful treatment of severe Crimean-Congo hemorrhagic fever with supportive measures without ribavirin and hypothermia. Journal of Clinical Virology. 2009;44(2):181-2.
[623] Yilmaz S, Yilmaz SI, Alay H, Kosan Z, Eren Z. Temporal tendency, seasonality and relationship with climatic factors of Crimean-Congo Hemorrhagic Fever cases (East of Turkey: 2012-2021). Heliyon. 2023;9(9).
[624] Yin X, Guo S, Ding C, Cao M, Kawabata H, Sato K, et al. Spotted Fever Group Rickettsiae in Inner Mongolia, China, 2015–2016. Emerging Infectious Diseases. 2018;24(11):2105-7.
[625] Yılmaz G, Öztürk B, Memikoğlu O, Coşkun B, Yalçı A, Metin Ö, et al. An Unusual Manifestation of Q Fever: Peritonitis. J Infect Public Health. 2015;8(4):373-6.
[626] Yoon EC, Vail E, Kleinman G, Lento PA, Li S, Wang G, et al. Lyme disease: a case report of a 17-year-old male with fatal Lyme carditis. Cardiovascular Pathology. 2015;24(5):317-21.
[627] Yoshii K, Hayasaka D, Goto A, Obara M, Araki K, Yoshimatsu K, et al. Enzyme-linked immunosorbent assay using recombinant antigens expressed in mammalian cells for serodiagnosis of tick-borne encephalitis. J Virol Methods. 2003;108(2):171-9.
[628] Yoshinari NH, Reinhardt BN, Steere AC. T cell responses to polypeptide fractions of Borrelia burgdorferi in patients with lyme arthritis. Arthritis Rheum. 2005;34(6):707-13.
[629] Young RP, Ip M, Bassett DCJ. Fatal Rickettsial Meningitis in Hong Kong: A Need for Rapid Laboratory Diagnosis. Scandinavian Journal of Infectious Diseases. 2009;27(5):527-8.
[630] Yu M, Fu X, Zhu Y, Li M, Wu M, Zhou E, et al. TaqMan qPCR and IgM Detection in Samples of Patients with Tick-Borne Encephalitis Virus Infection in Northeast China. International Journal of General Medicine. 2024;17:3745-53.
[631] Yu Q, Matkovic E, Reagan-Steiner S, Denison AM, Osborn R, Salamat SM. A Fatal Case of Powassan Virus Encephalitis. Journal of Neuropathology and Experimental Neurology. 2020;79(11):1239-43.
[632] Yurchenko OO, Dubyna DO, Vynograd NO, Rogovskyy AS. Phylogenetic analysis of tick -borne encephalitis virus strains found in an engorged tick and traveler returning from Russia. Ticks and Tick-Borne Diseases. 2021;12(3).

[633] Zahraei B, Hashemzadeh MS, Najarasl M, Zahiriyeganeh S, Tat M, Metanat M, et al. Novel, In-House, SYBR Green Based One-Step rRT-PCR: Rapid and Accurate Diagnosis of Crimean-Congo Hemorrhagic Fever Virus in Suspected Patients From Iran. Jundishapur Journal of Microbiology. 2016;9(1).

[634] Zaibaq J, Welden C, Mansour W, Axley P, Weber F. Cholestatic Jaundice: A Rare Presentation of Coxiella burnetii. American Journal of Gastroenterology. 2017;112.

[635] Zakeri A, Montaseri M, Shekarforoush SS. Prevalence and risk factors associated with Q fever infection in slaughterhouse workers in Fars province, Iran. International Archives of Occupational and Environmental Health. 2023;96(4):597-605.

[636] Zanchi AC, Gingold AR, Theise ND, Min AD. Necrotizing granulomatous hepatitis as an unusual manifestation of Lyme disease. Digestive Diseases and Sciences. 2007;52(10):2629-32.

[637] Zhai Y. Q fever represented as multiple pulmonary nodules: a case report. J Int Med Res. 2023;51(6):3000605231183553.

[638] Zhang J, Hao Y, Wang Z, Yang Q. Diagnosis of Coxiella burnetii infection via metagenomic next-generation sequencing: a case report. BMC Infect Dis. 2022;22(1):373.

[639] Zhang L. Nosocomial Transmission of Human Granulocytic Anaplasmosis in China. JAMA. 2008;300(19).

[640] Zhang L, Cui F, Wang L, Zhang L, Zhang J, Wang S, et al. Investigation of anaplasmosis in Yiyuan County, Shandong Province, China. Asian Pacific Journal of Tropical Medicine. 2011;4(7):568-72.

[641] Zhang L, Liu H, Xu B, Zhang Z, Jin Y, Li W, et al. Rural Residents in China Are at Increased Risk of Exposure to Tick-Borne Pathogens <i>Anaplasma phagocytophilum and Ehrlichia chaffeensis</i>. Biomed Research International. 2014;2014.

[642] Zhang L, Shan A, Mathew B, Yin J, Fu X, Zhang J, et al. Rickettsial Seroepidemiology among Farm Workers, Tianjin, People's Republic of China. Emerging Infectious Diseases. 2008;14(6):938-40.

[643] Zhang L, Zhu X, Hou X, Li H, Yang X, Chen T, et al. Prevalence and prediction of Lyme disease in Hainan province. Plos Neglected Tropical Diseases. 2021;15(3).

[644] Zhang L-j, Fu X-p, Zhang J-s. Q fever endocarditis with multi-organ complication: a case report. Chinese Medical Journal. 2006;119(18):1580-2.

[645] Zhang L-j, Li X-m, Zhang D-r, Zhang J-s, Di Y, Luan M-c, et al. Molecular epidemic survey on co-prevalence of scrub typhus and marine typhus in Yuxi city, Yunnan province of China. Chinese Medical Journal. 2007;120(15):1314-8.

[646] Zhang Liu L, Hou Xue X, Geng Z, Lou Yong L, Wan Kang L, Hao Q. Combination of Loop-Mediated Isothermal Amplification Assay and Nested PCR for Detection of <i>Borrelia burgdorferi sensu lato</i> in Human Serum Samples. Biomedical and Environmental Sciences. 2015;28(4):312-5.

[647] Zhang M, Lu D, Sun H, Zheng H, Cang M, Du Y. Serum Metabolomics of Tick-Borne Encephalitis Based on Orbitrap-Mass Spectrometry. International Journal of General Medicine. 2021;14:7995-8005.

[648] Zhang X, Chen H, Han D, Wu W. Clinical usefulness of metagenomic next-generation sequencing for Rickettsia and Coxiella burnetii diagnosis. European Journal of Clinical Microbiology & Infectious Diseases. 2023;42(6):681-9.

[649] Zhang X, Fan H, Jiao Y, Huang X. Defining the clinical characteristics of Q fever endocarditis: A case-control study in China. Journal of Infection in Developing Countries. 2022;16(8):1329-35.
[650] Zhang Y, Jiang L, Yang Y, Xie S, Yuan W, Wang Y. A tick bite patient with fever and meningitis co-infected with Rickettsia raoultii and Tacheng tick virus 1: a case report. Bmc Infectious Diseases. 2021;21(1).
[651] Zhang YT, Lafontant G, Bonner FJ. Lyme neuroborreliosis mimics stroke: A case report. Archives of Physical Medicine and Rehabilitation. 2000;81(4):519-21.
[652] Zhang. L, Zhu. X, Hou. X, Geng. Z, Chen. H, Hao. Q. Test of 259 serums from patients with arthritis or neurological symptoms confirmed existence of Lyme disease in Hainan province, China. International Journal of Clinical And Experimental Medicine. 2015;8(6):9531–6.
[653] Zhang. QE, Zhang. PH, Li. SQ, Li. RY, Pei. DK. Investigation of Lyme disease in Xinjiang. Chinese medical journal. 1991;104(3):244–6.
[654] Zhao Q, Li X, Zhang W, Chu C, Yao L, Zhang Y, et al. Epidemiological Characteristics and Spatial Analysis of Tick-Borne Encephalitis in Jilin Province, China. American Journal of Tropical Medicine and Hygiene. 2019;101(1):189-97.
[655] Zhioua E, Rodhain F, Binet P, Perez-Eid C. Prevalence of antibodies to Borrelia burgdorferi in forestry workers of Ile de France, France. European Journal of Epidemiology. 1997;13(8):959-62.
[656] Zhuo M, Caev H, Saunders SJ, Li J, Stillman IE, Danziger J. Acute Kidney Injury Associated With Human Granulocytic Anaplasmosis: A Case Report. American Journal of Kidney Diseases. 2019;74(5):696-9.
[657] Zohaib A, Saqib M, Athar MA, Hussain MH, Sial A-u-R, Tayyab MH, et al. Crimean-Congo Hemorrhagic Fever Virus in Humans and Livestock, Pakistan, 2015-2017. Emerging Infectious Diseases. 2020;26(4):773-7.
[658] Annie Lo HY, Cheng M, Chun L, Patel K, Lew W. Open repair of a Coxiella burnetii-associated abdominal aortic endovascular stent graft infection with a cryopreserved allograft using visceral artery pump perfusion. Journal of Vascular Surgery Cases, Innovations and Techniques. 2022;8(1):89-92.
[659] Gaüzère B-A, Malvy D, Filleul L, Ramful D, Jaffar-Bandjee M-C, El Bock M, et al. Intensive Care Unit Admission for Pandemic (H1N1) 2009, Reunion Island, 2009. Emerging Infectious Diseases. 2011;17(1):140-1.
[660] Genova-Kalou P, Vladimirova N, Stoitsova S, Krumova S, Kurchatova A, Kantardjiev T. Q fever in Bulgaria: Laboratory and epidemiological findings on human cases and outbreaks, 2011 to 2017. Eurosurveillance. 2019;24(37):25-31.
[661] Harris C, Kihonda J, Lwetoijera D, Dongus S, Devine G, Majambere S. A simple and efficient tool for trapping gravid Anopheles at breeding sites. Parasites & Vectors. 2011;4(1).
[662] Heitman KN, Dahlgren FS, Drexler NA, Massung RF, Behravesh CB. Increasing Incidence of Ehrlichiosis in the United States: A Summary of National Surveillance of <i>Ehrlichia chaffeensis</i> and <i>Ehrlichia ewingii</i> Infections in the United States, 2008-2012. American Journal of Tropical Medicine and Hygiene. 2016;94(1):52-60.
[663] Hernandez-Ruiz V, Edjolo A, Roubaud-Baudron C, Jaulhac B, Avila-Funes J-A, Dartigues J-F, et al. Association of Seropositivity to Borrelia burgdorferi With the Risk of Neuropsychiatric Disorders and Functional Decline in Older Adults The Aging Multidisciplinary Investigation

Study. Jama Neurology. 2020;77(2):210-4.

[664] Hotez PJ, Parola P, Socolovschi C, Jeanjean L, Bitam I, Fournier P-E, et al. Warmer Weather Linked to Tick Attack and Emergence of Severe Rickettsioses. PLoS Neglected Tropical Diseases. 2008;2(11).

[665] Hynote ED, Mervine PC, Stricker RB. Clinical evidence for rapid transmission of Lyme disease following a tickbite. Diagn Microbiol Infect Dis. 2012;72(2):188-92.

[666] Katsumata U, Sekizawa K, Inoue H, Sasaki H, Takishima T. Inhibitory actions of procaterol, a beta-2 stimulant, on substance P-induced cough in normal subjects during upper respiratory tract infection. The Tohoku Journal of Experimental Medicine. 1989;158(1):105-6.

[667] Laham FR, Jewell AM, Schoonover SL, Demmler GJ, Piedra PA. The Search for Adenovirus 14 in Children in Houston, Texas. Pediatric Infectious Disease Journal. 2008;27(7):653-4.

[668] Lane RS, Lennette ET, Madigan JE. Interlaboratory and intralaboratory comparisons of indirect immunofluorescence assays for serodiagnosis of Lyme disease. Journal of Clinical Microbiology. 1990;28(8):1774-9.

[669] Lantos PM, Tsao J, Nigrovic LE, Auwaerter PG, Fowler VG, Ruffin F, et al. Geographic Expansion of Lyme Disease in Michigan, 2000-2014. Open Forum Infect Dis. 2017;4(1):ofw269.

[670] Laroche C, Lienhardt A, Boulesteix J. Accident ischémique cérébral lié à une neuroborréliose. Archives de Pédiatrie. 1999;6(12):1302-5.

[671] Latifian M, Khalili M, Farrokhnia M, Mostafavi E, Esmaeili S. Rickettsia conorii subsp. israelensis infection: a case report from southeast Iran. Bmc Infectious Diseases. 2022;22(1).

[672] Lawrence CH, Botchlet R, Silberg SL, Flournoy DJ, Guthrie PJ. Prevalence of Lyme disease infection in Oklahoma. Journal of The National Medical Association. 1992;84(9):803-4.

[673] Le Marechal M, Mailles A, Seigneurin A, Tattevin P, Stahl J-P, Epaulard O, et al. A Prospective Cohort Study to Identify Clinical, Biological, and Imaging Features That Predict the Etiology of Acute Encephalitis. Clinical Infectious Diseases. 2021;73(2):264-70.

[674] Leblebicioglu H, Sunbul M, Barut S, Buyuktuna SA, Ozkurt Z, Yapar D, et al. Multi-center prospective evaluation of discharge criteria for hospitalized patients with Crimean-Congo Hemorrhagic Fever. Antiviral Research. 2016;133:9-13.

[675] Leblebicioglu H, Sunbul M, Guner R, Bodur H, Bulut C, Duygu F, et al. Healthcare-associated Crimean-Congo haemorrhagic fever in Turkey, 2002-2014: a multicentre retrospective cross-sectional study. Clinical Microbiology and Infection. 2016;22(4).

[676] Ledue TB, Collins MF, Young J, Schriefer ME. Evaluation of the Recombinant VlsE-Based Liaison Chemiluminescence Immunoassay for Detection of <i>Borrelia burgdorferi</i> and Diagnosis of Lyme Disease. Clinical and Vaccine Immunology. 2008;15(12):1796-804.

[677] Lee SH, Vigliotti JS, Vigliotti VS, Jones W, Shearer DM. Detection of Borreliae in Archived Sera from Patients with Clinically Suspect Lyme Disease. International Journal of Molecular Sciences. 2014;15(3):4284-98.

[678] Lefebvre M, Grossi O, Agard C, Perret C, Le Pape P, Raoult D, et al. Systemic immune presentations of Coxiella burnetii infection (Q Fever). Seminars in Arthritis and Rheumatism. 2010;39(5):405-9.

[679] Leiby DA, Chung APS, Cable RG, Trouern-Trend J, McCullough J, Homer MJ, et al. Relationship between tick bites and the seroprevalence of Babesia microti and Anaplasma phagocytophila (previously Ehrlichia sp.) in blood donors. Transfusion. 2002;42(12):1585-91.

[680] Leikauskas JA, Read JS, Kelso P, Nichols Heitman K, Armstrong PA, Kwit NA. Anaplasmosis-Related Fatality in Vermont: A Case Report. Vector-Borne and Zoonotic Diseases. 2022;22(3):188-90.

[681] Lennette EH, Clark WH, Jensen FW. Q Fever Studies. XII. Certain Observations on the Relationships Between Serologic Tests for Brucellosis, Syphilis and Q Fever. American Journal of Public Health and the Nations Health. 1952;42(1):12-9.

[682] Lenormand C, Jaulhac B, De Martino S, Barthel C, Lipsker D. Species of Borrelia burgdorferi complex that cause borrelial lymphocytoma in France. British Journal of Dermatology. 2009;161(1):174-6.

[683] Lenormand C, Jaulhac B, Debarbieux S, Dupin N, Granel-Brocard F, Adamski H, et al. Expanding the clinicopathological spectrum of late cutaneous Lyme borreliosis (acrodermatitis chronica atrophicans ACA): A prospective study of 20 culture-and/or polymerase chain reaction (PCR)-documented cases. Journal of the American Academy of Dermatology. 2016;74(4):685-92.

[684] Leone M, Iqbal A, Hugo Bonatti JR, Anwar S, Feaga C. A Patient with SIADH, Urinary Retention, Constipation, and Bell's Palsy following a Tick Bite. Case reports in nephrology. 2022;2022:5937131-.

[685] Lepidi H, Coulibaly B, Casalta JP, Raoult D. Autoimmunohistochemistry: A New Method for the Histologic Diagnosis of Infective Endocarditis. The Journal of Infectious Diseases. 2006;193(12):1711-7.

[686] Lepidi H, Houpikian P, Liang Z, Raoult D. Cardiac Valves in Patients with Q Fever Endocarditis: Microbiological, Molecular, and Histologic Studies. The Journal of Infectious Diseases. 2003;187(7):1097-106.

[687] Lesser RL, Kornmehl EW, Pachner AR, Kattah J, Hedges TR, Newman NM, et al. Neuro-Ophthalmologic Manifestations of Lyme Disease. Ophthalmology. 1990;97(6):699-706.

[688] Letrilliart L, Ragon B, Hanslik T, Flahault A. Lyme disease in France: a primary care-based prospective study. Epidemiology and Infection. 2005;133(5):935-42.

[689] Levy M, Abi-Warde MT, Rameau AC, Fafi-Kremer S, Hansmann Y, Fischbach M, et al. Tick-borne encephalitis in a child in a nonendemic country: A case report. Archives De Pediatrie. 2016;23(10):1055-8.

[690] Levy PY, Carrieri P, Raoult D. Coxiella burnetii Pericarditis: Report of 15 Cases and Review. Clinical Infectious Diseases. 1999;29(2):393-7.

[691] Levy PY, Drancourt M, Etienne J, Auvergnat JC, Beytout J, Sainty JM, et al. Comparison of different antibiotic regimens for therapy of 32 cases of Q fever endocarditis. Antimicrobial Agents and Chemotherapy. 1991;35(3):533-7.

[692] Li H, Zhang P-H, Huang Y, Du J, Cui N, Yang Z-D, et al. Isolation and Identification of Rickettsia raoultii in Human Cases: A Surveillance Study in 3 Medical Centers in China. Clinical Infectious Diseases. 2018;66(7):1109-15.

[693] Li H, Zhou Y, Wang W, Guo D, Huang S, Jie S. The clinical characteristics and outcomes of patients with human granulocytic anaplasmosis in China. International Journal of Infectious Diseases. 2011;15(12):E859-E66.

[694] Li Y, Wang J, Gao M, Fang L, Liu C, Lyu X, et al. Geographical Environment Factors and Risk Assessment of Tick-Borne Encephalitis in Hulunbuir, Northeastern China. International Journal

of Environmental Research and Public Health. 2017;14(6).

[695] Liddell AM, Sumner JW, Paddock CD, Rikihisa Y, Unver A, Buller RS, et al. Reinfection withEhrlichia chaffeensisin a Liver Transplant Recipient. Clinical Infectious Diseases. 2002;34(12):1644-7.

[696] Lieberman D, Lieberman D, Boldur I, Manor E, Hoffman S, Schlaeffer F, et al. Q-fever pneumonia in the Negev Region of Israel: A review of 20 patients hospitalised over a period of one year. Journal of Infection. 1995;30(2):135-40.

[697] Liegner KB. Disulfiram (Tetraethylthiuram Disulfide) in the Treatment of Lyme Disease and Babesiosis: Report of Experience in Three Cases. Antibiotics-Basel. 2019;8(2).

[698] Lin B, Kidder JM, Noring R, Steere Allen C, Klempner Mark S, Hu Linden T. Differences in Synovial Fluid Levels of Matrix Metalloproteinases Suggest Separate Mechanisms of Pathogenesis in Lyme Arthritis before and after Antibiotic Treatment. The Journal of Infectious Diseases. 2001;184(2):174-80.

[699] Lin S, Shrestha S, Prusinski MA, White JL, Lukacik G, Smith M, et al. The effects of multiyear and seasonal weather factors on incidence of Lyme disease and its vector in New York State. Science of the Total Environment. 2019;665:1182-8.

[700] Lindsey NP, Lehman JA, Staples JE, Fischer M. West Nile Virus and Other Arboviral Diseases— United States, 2013. Mmwr-morbidity And Mortality Weekly Report. 2014;63(24):521-6.

[701] Lindsey NP, Lehman JA, Staples JE, Fischer M. West Nile Virus and Other Nationally Notifiable Arboviral Diseases — United States, 2014. MMWR Morbidity and Mortality Weekly Report. 2015;64(34):929-34.

[702] Lipsett SC, Branda JA, McAdam AJ, Vernacchio L, Gordon CD, Gordon CR, et al. Evaluation of the C6 Lyme Enzyme Immunoassay for the Diagnosis of Lyme Disease in Children and Adolescents. Clinical Infectious Diseases. 2016;63(7):922-8.

[703] Lipsker D, Antoni-Bach N, Hansmann Y, Jaulhac B. Long-term prognosis of patients treated for erythema migrans in France. British Journal of Dermatology. 2002;146(5):872-6.

[704] Lipsker D, Boeckler P, Cribier B. Tick-borne lymphadenopathy/dermacentor-borne necrosis erythema lymphadenopathy: an infectious cause of cicatricial alopecia. Clinical and Experimental Dermatology. 2008;33(4):518-9.

[705] Little EAH, Anderson JF, Stafford KC, III, Eisenb L, Eisen RJ, Molaei G. Predicting spatiotemporal patterns of Lyme disease incidence from passively collected surveillance data for <i>Borrelia burgdorferi</i> sensu lato-infected <i>Ixodes scapularis</i> ticks. Ticks and Tick-Borne Diseases. 2019;10(5):970-80.

[706] Liu J, Ai H, Xiong Y, Li F, Wen Z, Liu W, et al. Prevalence and Correlation of Infectious Agents in Hospitalized Children with Acute Respiratory Tract Infections in Central China. PLoS ONE. 2015;10(3).

[707] Liu W, Liu Hui X, Zhang L, Hou Xue X, Wan Kang L, Hao Q. Evaluation of Six Recombinant Proteins for Serological Diagnosis of Lyme Borreliosis in China. Biomedical and Environmental Sciences. 2016;29(5):323-30.

[708] Liveris D, Wang GQ, Girao G, Byrne DW, Nowakowski J, McKenna D, et al. Quantitative detection of Borrelia burgdorferi in 2-millimeter skin samples of erythema migrans lesions: correlation of results with clinical and laboratory findings. Journal of Clinical Microbiology. 2002;40(4):1249-53.

[709] Lochhead RB, Arvikar SL, Aversa JM, Sadreyev RI, Strle K, Steere AC. Robust interferon signature and suppressed tissue repair gene expression in synovial tissue from patients with postinfectious, <i>Borrelia burgdorferi</i>-induced Lyme arthritis. Cellular Microbiology. 2019;21(2).

[710] Lochhead RB, Strle K, Kim ND, Kohler MJ, Arvikar SL, Aversa JM, et al. MicroRNA Expression Shows Inflammatory Dysregulation and Tumor-Like Proliferative Responses in Joints of Patients With Postinfectious Lyme Arthritis. Arthritis & Rheumatology. 2017;69(5):1100-10.

[711] Loukaides F, Hadjichristodoulou C, Soteriades ES, Kolonia V, Ioannidou MC, Psaroulaki A, et al. Active surveillance of Q fever in human and animal population of Cyprus. Bmc Infectious Diseases. 2006;6.

[712] Lovrich SD, Jobe DA, Kowalski TJ, Policepatil SM, Callister SM. Expansion of the Midwestern Focus for Human Granulocytic Anaplasmosis into the Region Surrounding La Crosse, Wisconsin. Journal of Clinical Microbiology. 2011;49(11):3855-9.

[713] Lu M, Chen Q, Qin X, Lyu Y, Teng Z, Li K, et al. Anaplasma bovis Infection in Fever and Thrombocytopenia Patients - Anhui Province, China, 2021. China CDC Weekly. 2022;4(12):249-53.

[714] Lu M, Li F, Liao Y, Shen J-J, Xu J-M, Chen Y-Z, et al. Epidemiology and Diversity of Rickettsiales Bacteria in Humans and Animals in Jiangsu and Jiangxi provinces, China. Scientific Reports. 2019;9.

[715] Lundy P, Arnold P, Hance K. Coxiella burnetii infection of the spine requiring neurosurgical intervention. Surg Neurol Int. 2019;10:182.

[716] Luo S, Yang S, Liu A, Wu H, Gao L, Wu X, et al. Serological and molecular epidemiological investigation of Mediterranean spotted fever in Yunnan Province, China. Infection Genetics and Evolution. 2024;118.

[717] Lupoglazoff JM, Brouqui P, Magnier S, Hvass U, Casasoprana A. Q fever tricuspid valve endocarditis. Archives of Disease in Childhood. 1997;77(5):448-9.

[718] Magnarelli LA, Dumler JS, Anderson JF, Johnson RC, Fikrig E. Coexistence of antibodies to tick-borne pathogens of babesiosis, ehrlichiosis, and Lyme borreliosis in human sera. Journal of Clinical Microbiology. 1995;33(11):3054-7.

[719] Magnarelli LA, Ijdo JW, Anderson JF, Padula SJ, Flavell RA, Fikrig E. Human Exposure to a Granulocytic Ehrlichia and Other Tick-Borne Agents in Connecticut. Journal of Clinical Microbiology. 1998;36(10):2823-7.

[720] Magnaval J-F, Leparc-Goffart I, Gibert M, Gurieva A, Outreville J, Dyachkovskaya P, et al. A Serological Survey About Zoonoses in the Verkhoyansk Area, Northeastern Siberia (Sakha Republic, Russian Federation). Vector-Borne and Zoonotic Diseases. 2016;16(2):103-9.

[721] Mah A, Viola GM, Ariza Heredia E, Rezvani K, Kebriaei P, Bhatti MM, et al. Graft loss attributed to possible transfusion-transmitted ehrlichiosis following cord blood stem cell transplant. Transpl Infect Dis. 2018;20(4):e12899.

[722] Mailles A, Argemi X, Biron C, Fillatre P, De Broucker T, Buzele R, et al. Changing profile of encephalitis: Results of a 4-year study in France. Infectious Diseases Now. 2022;52(1):1-6.

[723] Majeed B, Dicker R, Nawar A, Badri S, Noah A, Muslem H. Morbidity and mortality of Crimean-Congo hemorrhagic fever in Iraq: cases reported to the National Surveillance System, 1990-2010. Transactions of the Royal Society of Tropical Medicine and Hygiene. 2012;106(8):480-3.

[724] Makwana D, Yadav PD, Kelaiya A, Mourya DT. First confirmed case of Crimean-Congo haemorrhagic fever from Sirohi district in Rajasthan State, India. Indian Journal of Medical Research. 2015;142:489-91.

[725] Maltezou HC, Papa A, Tsiodras S, Dalla V, Maltezos E, Antoniadis A. Crimean-Congo hemorrhagic fever in Greece: a public health perspective. International Journal of Infectious Diseases. 2009;13(6):713-6.

[726] Maltezou HC, Papa A, Ventouri S, Tseki C, Pervanidou D, Pavli A, et al. A case of Crimean-Congo haemorrhagic fever imported in Greece: Contact tracing and management of exposed healthcare workers. Journal of infection prevention. 2019;20(4):171-8.

[727] Mandelcwajg A, Menager C, Cheron G. Mediterranean spotted fever in a 3-year-old child. Archives De Pediatrie. 2014;21(4):396-8.

[728] Maraspin V, Bogovič P, Ogrinc K, Rojko T, Ružić-Sabljić E, Kastrin A, et al. Are Differences in Presentation of Early Lyme Borreliosis in Europe and North America a Consequence of a More Frequent Spirochetemia in American Patients? J Clin Med. 2021;10(7).

[729] Marcos LA, Smith K, Reardon K, Weinbaum F, Spitzer ED. Presence of Borrelia miyamotoi infection in a highly endemic area of Lyme disease. Annals of Clinical Microbiology and Antimicrobials. 2020;19(1).

[730] Marguet C, Rouillier-Saas M, Mallet E, Meunier M, Jeannot E, Boulloche J, et al. Maladie de Lyme chez l'enfant en Haute-Normandie: à propos d'une enquête hospitalière. Archives de Pédiatrie. 2000;7:S517-S22.

[731] Marques AR, Stock F, Gill V. Evaluation of a New Culture Medium forBorrelia burgdorferi. Journal of Clinical Microbiology. 2000;38(11):4239-41.

[732] Marshall GS. Ehrlichia chaffeensis Seroprevalence Among Children in the Southeast and South-Central Regions of the United States. Arch Pediatr Adolesc Med. 2002;156(2).

[733] Martinez-Balzano C, Hess M, Malhotra A, Lenox R. Severe babesiosis and Borrelia burgdorferi co-infection. Qjm-an International Journal of Medicine. 2015;108(2):141-3.

[734] Marty AM, Dumler JS, Imes G, Brusman HP, Smrkovski LL, Frisman DM. Ehrlichiosis mimicking thrombotic thrombocytopenic purpura. Case report and pathological correlation. Human Pathology. 1995;26(8):920-5.

[735] Martzolff L, Bouhala M, Dukic R, Saraceni O, Wilhelm JM, Bombaron P, et al. Recurrent nerve palsy due to Lyme disease: Report of two cases. Revue De Medecine Interne. 2010;31(3):229-31.

[736] Marx GE, Leikauskas J, Lindstrom K, Mann E, Reagan-Steiner S, Matkovic E, et al. Fatal Lyme Carditis in New England: Two Case Reports. Annals of Internal Medicine. 2020;172(3):222-+.

[737] Massengo SA, Bonnet F, Braun C, Vital A, Beylot J, Bastard J. Severe neuroborreliosis: The benefit of prolonged high-dose combination of antimicrobial agents with steroids--an illustrative case. Diagn Microbiol Infect Dis. 2005;51(2):127-30.

[738] Masters E, Granter S, Duray P, Cordes P. Physician-Diagnosed Erythema Migrans and Erythema Migrans–like Rashes Following Lone Star Tick Bites. Archives of Dermatology. 1998;134(8).

[739] Maukayeva S, Karimova S. Tick-Borne Encephalitis in Kazakhstan: A Case Report. Erciyes Medical Journal. 2020;42(2):226-8.

[740] Mboussou Y, Jaubert J, Larrieu S, Atiana L, Naze F, Folio C, et al. Pregnancy outcomes of Q fever: prospective follow-up study on Reunion island. Bmc Infectious Diseases. 2019;19(1).

[741] McDonald E, Martin SW, Landry K, Gould CV, Lehman J, Fischer M, et al. West Nile Virus and

Other Domestic Nationally Notifiable Arboviral Diseases — United States, 2018. MMWR Morbidity and Mortality Weekly Report. 2019;68(31):673-8.

[742] Mehmood Q, Tahir MJ, Jabbar A, Siddiqi AR, Ullah I. Crimean–Congo hemorrhagic fever outbreak in Turkey amid the coronavirus disease 2019 (COVID-19) pandemic; a debacle for the healthcare system of Turkey. Infection Control & Hospital Epidemiology. 2021;43(11):1726-7.

[743] Melenotte C, Bart G, Kraeber-Bodere F, Cammilleri S, Le Goff B, Raoult D. Isolation of Coxiella burnetii from an acromioclavicular infection with low serological titres. Int J Infect Dis. 2018;73:27-9.

[744] Melenotte C, Epelboin L, Million M, Hubert S, Monsec T, Djossou F, et al. Acute Q Fever Endocarditis: A Paradigm Shift Following the Systematic Use of Transthoracic Echocardiography During Acute Q Fever. Clin Infect Dis. 2019;69(11):1987-95.

[745] Melenotte C, Loukil A, Rico A, Lepidi H, Raoult D. Blood Culture-Negative Cardiovascular Infection in a Patient With Multiple Sclerosis. Open Forum Infect Dis. 2019;6(10):ofz429.

[746] Melenotte C, Mezouar S, Ben Amara A, Benatti S, Chiaroni J, Devaux C, et al. A transcriptional signature associated with non-Hodgkin lymphoma in the blood of patients with Q fever. PLoS ONE. 2019;14(6):e0217542.

[747] Melenotte C, Million M, Audoly G, Gorse A, Dutronc H, Roland G, et al. B-cell non-Hodgkin lymphoma linked to Coxiella burnetii. Blood. 2016;127(1):113-21.

[748] Melenotte C, Million M, Hartung O, Botelho-Nevers E, Claudel M, Craighero F, et al. Query rectal bleeding. Lancet. 2012;380(9839):446.

[749] Melenotte C, Protopopescu C, Million M, Edouard S, Carrieri MP, Eldin C, et al. Clinical Features and Complications of Coxiella burnetii Infections From the French National Reference Center for Q Fever. Jama Network Open. 2018;1(4).

[750] Meltzer E, Paran Y, Lustig Y, Stienlauf S, Weinberger M, Schwartz E. Travel-Related Tick-Borne Encephalitis, Israel, 2006-2014. Emerging Infectious Diseases. 2017;23(1):119-21.

[751] Memish ZA, Albarrak A, Almazroa MA, Al-Omar I, Alhakeem R, Assiri A, et al. Seroprevalence of Alkhurma and Other Hemorrhagic Fever Viruses, Saudi Arabia. Emerging Infectious Diseases. 2011;17(12):2316-8.

[752] Memon A, Abdelghany A, Abusuliman M, Eldesouki M, Fatima M, Abdelhalim O, et al. Altered Mental Status on Top of Anaplasmosis-Induced Severe Rhabdomyolysis: A Rare Clinical Presentation. Cureus. 2023;15(9):e45020.

[753] Mendoza MA, Hass RM, Vaillant J, Johnson DR, Theel ES, Toledano M, et al. Powassan Virus Encephalitis: A Tertiary Center Experience. Clinical Infectious Diseases. 2024;78(1):80-9.

[754] Mentese A, Yilmaz G, Sumer A, Arslan M, Karahan SC, Koksal I. The diagnostic and prognostic significance of SCUBE1 levels in Crimean-Congo hemorrhagic fever. International Journal of Infectious Diseases. 2013;17(11):E1042-E5.

[755] Merati M, Rucker JC, McKeon A, Frucht SJ, Hu J, Balcer LJ, et al. A Case of Opsoclonus-Myoclonus-Ataxia With Neuronal Intermediate Filament IgG Detected in Cerebrospinal Fluid. J Neuroophthalmol. 2022;42(2):278-81.

[756] Merhej V, Cammilleri S, Piquet P, Casalta J-P, Raoult D. Relevance of the positron emission tomography in the diagnosis of vascular graft infection with <i>Coxiella burnetii</i>. Comparative Immunology Microbiology and Infectious Diseases. 2012;35(1):45-9.

[757] Meriglier E, Sunder A, Elsendoorn A, Canoui E, Rammaert B, Million M, et al. Osteoarticular

manifestations of Q fever: a case series and literature review. Clin Microbiol Infect. 2018;24(8):912-3.

[758] Mesana TG, Collart F, Caus T, Salamand A. Q fever endocarditis: A surgical view and a word of caution. Journal of Thoracic and Cardiovascular Surgery. 2003;125(1):217-8.

[759] Meslin P, Renoux MC, Manin C, Wendremaire P, Rosselini D, Tambat A, et al. A 2-month-old baby with Mediterranean spotted fever. Archives De Pediatrie. 2014;21(7):772-5.

[760] Metanat M, Mood BS, Salehi M, Moghaddam AA, Rakhshani M, Rad NS. Viral Load as a Predictor of Severity in Crimean-Congo Hemorrhagic Fever. Archives of Clinical Infectious Diseases. 2017;12(1).

[761] Miailhes P, Conrad A, Sobas C, Laurent F, Lustig S, Ferry T. Coxiella burnetti prosthetic joint infection in an immunocompromised woman: iterative surgeries, prolonged ofloxacin-rifampin treatment and complex reconstruction were needed for the cure. Arthroplasty. 2021;3(1):43.

[762] Michalski B, Umpierrez De Reguero A. Lyme Carditis Buried Beneath ST-Segment Elevations. Case Rep Cardiol. 2017;2017:9157625.

[763] Middelveen MJ, Bandoski C, Burke J, Sapi E, Filush KR, Wang Y, et al. Exploring the association between Morgellons disease and Lyme disease: identification of Borrelia burgdorferi in Morgellons disease patients. Bmc Dermatology. 2015;15.

[764] Middelveen MJ, Burke J, Sapi E, Bandoski C, Filush KR, Wang Y, et al. Culture and identification of Borrelia spirochetes in human vaginal and seminal secretions. F1000Research. 2014;3.

[765] Middelveen MJ, Sapi E, Burke J, Filush KR, Franco A, Fesler MC, et al. Persistent Borrelia Infection in Patients with Ongoing Symptoms of Lyme Disease. Healthcare (Basel). 2018;6(2).

[766] Middelveen MJ, Shah JS, Fesler MC, Stricker RB. Relapsing fever Borrelia in California: a pilot serological study. International Journal of General Medicine. 2018;11:373-82.

[767] Midilli K, Gargili A, Ergonul O, Elevli M, Ergin S, Turan N, et al. The first clinical case due to AP92 like strain of Crimean-Congo Hemorrhagic Fever virus and a field survey. Bmc Infectious Diseases. 2009;9.

[768] Midilli K, Gargili A, Ergonul O, Sengoz G, Ozturk R, Bakar M, et al. Imported Crimean-Congo hemorrhagic fever cases in Istanbul. Bmc Infectious Diseases. 2007;7.

[769] Miernyk KM, Bruden D, Parkinson AJ, Hurlburt D, Klejka J, Berner J, et al. Human Seroprevalence to 11 Zoonotic Pathogens in the US Arctic, Alaska. Vector-Borne and Zoonotic Diseases. 2019;19(8):563-75.

[770] Miller HK, Stoddard RA, Dawsey SM, Nasrollahzadeh D, Abnet CC, Etemadi A, et al. Association Between Serological Responses to Two Zoonotic Ruminant Pathogens and Esophageal Squamous Cell Carcinoma. Vector-Borne and Zoonotic Diseases. 2021;21(2):125-7.

[771] Million M, Bardin N, Bessis S, Nouiakh N, Douliery C, Edouard S, et al. Thrombosis and antiphospholipid antibody syndrome during acute Q fever A cross-sectional study. Medicine. 2017;96(29).

[772] Mitchell PD, Reed KD, Hofkes JM. Immunoserologic evidence of coinfection with Borrelia burgdorferi, Babesia microti, and human granulocytic Ehrlichia species in residents of Wisconsin and Minnesota. Journal of Clinical Microbiology. 1996;34(3):724-7.

[773] Mofleh J, Ahmad AZ. Crimean–Congo haemorrhagic fever outbreak investigation in the Western Region of Afghanistan in 2008. Eastern Mediterranean Health Journal. 2012;18(5):522-6.

[774] Mohamad Alahmad MA, Hammoud KA. Inpatient Q Fever Frequency Is on the Rise. Canadian

Journal of Infectious Diseases & Medical Microbiology. 2023;2023.
[775] Mohareb E, Christova I, Soliman A, Younan R, Kantardjiev T. Tick-borne encephalitis in Bulgaria, 2009 to 2012. Eurosurveillance. 2013;18(46).
[776] Mokrani K, Tebbal S, Raoult D, Fournier P-E. Human rickettsioses in the Batna area, eastern Algeria. Ticks and Tick-Borne Diseases. 2012;3(5-6):363-5.
[777] Molloy PJ, Weeks KE, Todd B, Wormser GP. Seroreactivity to the C6 Peptide in Borrelia miyamotoi Infections Occurring in the Northeastern United States. Clinical Infectious Diseases. 2018;66(9):1407-10.
[778] Moradnejad P, Esmaeili S, Maleki M, Sadeghpour A, Kamali M, Rohani M, et al. Q Fever Endocarditis in Iran. Scientific Reports. 2019;9.
[779] Morand A, Angelakis E, Ben Chaabane M, Parola P, Raoult D, Gautret P. Seek and Find! PCR analyses of skin infections in West-European travelers returning from abroad with an eschar. Travel Medicine and Infectious Disease. 2018;26:32-6.
[780] Mosel MR, Rebman AW, Carolan HE, Montenegro T, Lovari R, Schutzer SE, et al. Molecular Microbiological and Immune Characterization of a Cohort of Patients Diagnosed with Early Lyme Disease. J Clin Microbiol. 2020;59(1).
[781] Moss WJ, Dumler JS. Simultaneous infection with Borrelia burgdorferi and human granulocytic ehrlichiosis. The Pediatric Infectious Disease Journal. 2003;22(1):91-2.
[782] Mostafavi E, Chinikar S, Bokaei S, Haghdoost A. Temporal modeling of Crimean-Congo hemorrhagic fever in eastern Iran. International Journal of Infectious Diseases. 2013;17(7):E524-E8.
[783] Mostafavi E, Haghdoost A, Khakifirouz S, Chinikar S. Spatial Analysis of Crimean Congo Hemorrhagic Fever in Iran. American Journal of Tropical Medicine and Hygiene. 2013;89(6):1135-41.
[784] Mostafavi E, Molaeipoor L, Esmaeili S, Ghasemi A, Kamalizad M, Behzadi MY, et al. Seroprevalence of Q fever among high-risk occupations in the Ilam province, the west of Iran. PLoS ONE. 2019;14(2).
[785] Mostafavi E, Pourhossein B, Esmaeili S, Amiri FB, Khakifirouz S, Shah-Hosseini N, et al. Seroepidemiology and risk factors of Crimean-Congo Hemorrhagic Fever among butchers and slaughterhouse workers in southeastern Iran. International Journal of Infectious Diseases. 2017;64:85-9.
[786] Mouffok N, Parola P, Abdennour D, Aouati A, Razik F, Benabdellah A, et al. Mediterranean spotted fever in Algerian children. Clinical Microbiology and Infection. 2009;15:290-1.
[787] Mouffok N, Parola P, Lepidi H, Raoult D. Mediterranean spotted fever in Algeria - new trends. International Journal of Infectious Diseases. 2009;13(2):227-35.
[788] Mourya DT, Viswanathan R, Jadhav SK, Yadav PD, Basu A, Chadha MS. Retrospective analysis of clinical information in Crimean-Congo haemorrhagic fever patients: 2014-2015, India. Indian Journal of Medical Research. 2017;145:672-7.
[789] Mourya DT, Yadav PD, Gurav YK, Pardeshi PG, Shete AM, Jain R, et al. Crimean Congo hemorrhagic fever serosurvey in humans for identifying high-risk populations and high-risk areas in the endemic state of Gujarat, India. Bmc Infectious Diseases. 2019;19.
[790] Mourya DT, Yadav PD, Shete AM, Gurav YK, Raut CG, Jadi RS, et al. Detection, Isolation and Confirmation of Crimean-Congo Hemorrhagic Fever Virus in Human, Ticks and Animals in

Ahmadabad, India, 2010-2011. Plos Neglected Tropical Diseases. 2012;6(5).
[791] Mousapour M, Oveisi A, Key YA, Mikaeili E, Rahimi F, Shademan B, et al. First Serological & Molecular Study of Coxiella burnetii in Stray, Domestic Cats, and Their Owners in Iran. Topics in Companion Animal Medicine. 2020;41.
[792] Muehlenbachs A, Bollweg BC, Schulz TJ, Forrester JD, Carnes MD, Molins C, et al. Cardiac Tropism of <i>Borrelia burgdorferi An Autopsy Study of Sudden Cardiac Death Associated with Lyme Carditis</i>. American Journal of Pathology. 2016;186(5):1195-205.
[793] Mullholand JB, Tolman N, De Obaldia A, Hennrikus E. Central nervous system involvement of anaplasmosis. BMJ Case Rep. 2021;14(12).
[794] Musso D, Raoult D. Coxiella burnetii blood cultures from acute and chronic Q-fever patients. Journal of Clinical Microbiology. 1995;33(12):3129-32.
[795] Mustafa ML, Ayazi E, Mohareb E, Yingst S, Zayed A, Rossi CA, et al. Crimean-Congo Hemorrhagic Fever, Afghanistan, 2009. Emerging Infectious Diseases. 2011;17(10):1940-1.
[796] Mwirigi NW, Rodriguez-Porcel M. 31-year-old man with fever, palpitations, and generalized rash. Mayo Clin Proc. 2010;85(4):e13-6.
[797] Myers F, Mishra PE, Cortez D, Schleiss MR. Chest palpitations in a teenager as an unusual presentation of Lyme disease: case report. Bmc Infectious Diseases. 2020;20(1).
[798] Nachamkin I, Riddle DL, Feldman M, Edelstein PH. Utilization of tests for Lyme disease antibody at a university hospital. Clinical Diagnostic Laboratory Immunology. 1996;3(3):287-9.
[799] Naderi H, Sheybani F, Bojdi A, Khosravi N, Mostafavi I. Short Report: Fatal Nosocomial Spread of Crimean-Congo Hemorrhagic Fever with Very Short Incubation Period. American Journal of Tropical Medicine and Hygiene. 2013;88(3):469-71.
[800] Nadjm B, Van Tulleken C, Macdonald D, Chiodini PL. East African Trypanosomiasis in a Pregnant Traveler. Emerging Infectious Diseases. 2009;15(11):1866-7.
[801] Naeem M, Enos D, Shah S, Patel N, Fisher T. Management of Chronic Symptoms of Lyme Disease With Intravenous Ceftriaxone. Cureus. 2021;13(7):e16354.
[802] Nayak SU, Simon GL. Myocarditis after Trimethoprim/Sulfamethoxazole Treatment for Ehrlichiosis. Emerging Infectious Diseases. 2013;19(12):1975-7.
[803] Newman ENC, Johnstone P, Bridge H, Wright D, Jameson L, Bosworth A, et al. Seroconversion for Infectious Pathogens among UK Military Personnel Deployed to Afghanistan, 2008-2011. Emerging Infectious Diseases. 2014;20(12):2015-22.
[804] Niazi A-u-R, Jawad MJ, Amirnajad A, Durr PA, Williams DT. Crimean-Congo Hemorrhagic Fever, Herat Province, Afghanistan, 2017. Emerging Infectious Diseases. 2019;25(8):1596-8.
[805] Nigrovic LE, Neville DN, Chapman L, Balamuth F, Levas MN, Thompson AD, et al. Multiplex High-Definition Polymerase Chain Reaction Assay for the Diagnosis of Tick-borne Infections in Children. Open Forum Infect Dis. 2023;10(4):ofad121.
[806] Nikiforova MA, Kuznetsova NA, Shchetinin AM, Butenko AM, Kozlova AA, Larichev VP, et al. Arboviruses in the Astrakhan region of Russia for 2018 season: The development of multiplex PCR assays and analysis of mosquitoes, ticks, and human blood sera. Infection Genetics and Evolution. 2021;88.
[807] Nili S, Khanjani N, Jahani Y, Bakhtiari B. The effect of climate variables on the incidence of Crimean Congo Hemorrhagic Fever (CCHF) in Zahedan, Iran. BMC Public Health. 2020;20(1).
[808] Nokhodian Z, Ataei B, Moradi A, Yaran M, Hoseini SG, Feizi A, et al. Seroprevalence and risk

factors of Coxiella burnetii infection among high-risk population in center of Iran, a neglected health problem. Acta Tropica. 2017;169:107-11.

[809] Nokhodian Z, Feizi A, Khalili M, Ataei B, Moradi A. Molecular evidence and risk factors of Coxiella burnetii among seropositive high-risk individuals in the center of Iran. Comparative Immunology Microbiology and Infectious Diseases. 2018;61:34-7.

[810] Nur YA, Groen J, Yusuf MA, Osterhaus ADME. IgM antibodies in hospitalized children with febrile illness during an inter-epidemic period of measles, in Somalia. Journal of Clinical Virology. 1999;12(1):21-5.

[811] O'Connor C, Prusinski MA, Jiang S, Russell A, White J, Falco R, et al. A Comparative Spatial and Climate Analysis of Human Granulocytic Anaplasmosis and Human Babesiosis in New York State (2013-2018). Journal of Medical Entomology. 2021;58(6):2453-66.

[812] Oaks JB, Lasam G, LaCapra G. Mediterranean Spotted Fever: A Rare Non-Endemic Disease in the USA. Cureus Journal of Medical Science. 2017;9(1).

[813] Obafemi AI, Le J. Perioperative Occupational Exposure to Coxiella burnetii-Infected Thoracic Endovascular Aneurysm Stent Graft. International Journal of Occupational and Environmental Medicine. 2017;8(1):46-9.

[814] Oelschlaeger S, Gabriel M, Schmidt-Chanasit J, Meyer M, Osborn E, Conger NG, et al. Complete sequence and phylogenetic characterisation of Crimean-Congo hemorrhagic fever virus from Afghanistan. Journal of Clinical Virology. 2011;50(1):90-2.

[815] Oktay AA, Dibs SR, Friedman H. Sinus Pause in Association with Lyme Carditis. Texas Heart Institute Journal. 2015;42(3):248-50.

[816] Olano JP, Hogrefe W, Seaton B, Walker DH. Clinical manifestations, epidemiology, and laboratory diagnosis of human monocytotropic ehrlichiosis in a commercial laboratory setting. Clinical and Diagnostic Laboratory Immunology. 2003;10(5):891-6.

[817] Olano JP, Masters E, Hogrefe W, Walker DH. Human Monocytotropic Ehrlichiosis, Missouri. Emerging Infectious Diseases. 2003;9(12):1579-86.

[818] Orloski KA, Hayes EB, Campbell BS, Dennis DT. Surveillance for Lyme disease--United States, 1992-1998. Mmwr Surveillance Summaries. 2000;49(3):1-11.

[819] Ostapchuk YO, Dmitrovskiy AM, Pak EA, Perfilyeva YV. A case of combined infection with tick-borne encephalitis and lyme borreliosis with severe meningoencephalitis and complete recovery. Journal of Global Infectious Diseases. 2023;15(2):81-3.

[820] Ostapchuk YO, Perfilyeva YV, Zhigailov AV, Maltseva ER, Neupokoyeva AS, Bissenbay AO, et al. Monitoring of pathogenic Borrelia burgdorferi sensu lato in the Almaty oblast, Kazakhstan. Ticks and Tick-Borne Diseases. 2021;12(4).

[821] Osterholm MT, Forfang JC, Kuritsky JN. Lyme disease in Minnesota: epidemiologic and serologic findings. Yale Journal of Biology And Medicine. 1984;57(4):677-83.

[822] Owolabi T, Simpson I. An annular rash. American Family Physician. 2014;89(7):581-2.

[823] Ozgen EK, Kilicoglu Y, Yanmaz B, Ozmen M, Ulucan M, Bagatir PS, et al. Molecular epidemiology of Coxiella burnetii detected in humans and domestic ruminants in Turkey. Veterinary Microbiology. 2022;273.

[824] Ozkurt Z, Ozden K, Kiki I, Usanmaz M. Prognostic significance of antithrombin activity in patients with crimean-congo hemorrhagic Fever. Eurasian J Med. 2011;43(2):83-6.

[825] Ozsoy S, Gokmen A, Ozdemir M, Akduman B, Korkusuz I, Javan GT. Medical examiners and

Crimean-Congo hemorrhagic fever contamination risk. Journal of Forensic and Legal Medicine. 2015;36:32-6.
[826] Ozturk B, Tutuncu E, Kuscu F, Gurbuz Y, Sencan I, Tuzun H. Evaluation of factors predictive of the prognosis in Crimean-Congo hemorrhagic fever: new suggestions. International Journal of Infectious Diseases. 2012;16(2):E89-E93.
[827] Pacheco A, Rutler O, Valenzuela I, Feldman D, Eskin B, Allegra JR. Positive Tests for Lyme Disease and Emergency Department Visits for Bell's Palsy Patients. Journal of Emergency Medicine. 2020;59(6):820-7.
[828] Paddock CD, Sumner JW, Shore GM, Bartley DC, Elie RC, McQuade JG, et al. Isolation and characterization of Ehrlichia chaffeensis strains from patients with fatal ehrlichiosis. Journal of Clinical Microbiology. 1997;35(10):2496-502.
[829] Pagni S, Dempsey A, Austin EH, 3rd. Tricuspid and aortic valve and ventricular septal defect endocarditis: an unusual presentation of acute Q fever. Ann Thorac Surg. 2009;88(6):2027-9.
[830] Pan L, Zhang L, Fan D, Zhang X, Liu H, Lu Q, et al. Rapid, Simple and Sensitive Detection of Q Fever by Loop-Mediated Isothermal Amplification of the <i>htpAB</i> Gene. Plos Neglected Tropical Diseases. 2013;7(5).
[831] Panda S, Sharma S, Bajpai N, Gopalakrishnan M, Kombade SP, Nag VL. A Rare Fatal Cause of Acute Areflexic Quadriparesis in the Tropics. Neurol India. 2020;68(5):1196-200.
[832] Panou F, Papadopoulos C, Kolokathis F, Gannitsioti E, Tsiodras S, Giamarellou E, et al. Infective aortic valve endocarditis from Coxiella burnetii. Hellenic Journal of Cardiology. 2007;48(3):177-80.
[833] Papa A, Chaligiannis I, Kontana N, Sourba T, Tsioka K, Tsatsaris A, et al. A novel AP92-like Crimean-Congo hemorrhagic fever virus strain, Greece. Ticks and Tick-Borne Diseases. 2014;5(5):590-3.
[834] Papa A, Christova I, Papadimitriou E, Antoniadis A. Crimean-Congo Hemorrhagic Fever in Bulgaria. Emerging Infectious Diseases. 2004;10(8):1465-7.
[835] Papa A, Dalla V, Papadimitriou E, Kartalis GN, Antoniadis A. Emergence of Crimean-Congo haemorrhagic fever in Greece. Clinical Microbiology and Infection. 2010;16(7):843-7.
[836] Papa A, Dalla V, Petala A, Maltezou HC, Maltezos E. Fatal Mediterranean spotted fever in Greece. Clinical Microbiology and Infection. 2010;16(6):589-92.
[837] Papa A, Sidira P, Kallia S, Ntouska M, Zotos N, Doumbali E, et al. Factors associated with IgG positivity to Crimean-Congo hemorrhagic fever virus in the area with the highest seroprevalence in Greece. Ticks and Tick-Borne Diseases. 2013;4(5):417-20.
[838] Papakonstantinou PE, Samonis G, Andrianaki AM, Christofaki M, Dimopoulou D, Papadakis J, et al. Epidemiology, Microbiological and Clinical Features, Treatment, and Outcomes of Infective Endocarditis in Crete, Greece. Infection and Chemotherapy. 2018;50(1):21-8.
[839] Pape M, Mandraveli K, Arvanitidou-Vagiona M, Nikolaidis P, Alexion-Daniel S. Q fever in northern Greece: epidemiological and clinical data from 58 acute and chronic cases. Clinical Microbiology and Infection. 2009;15:150-1.
[840] Pape M, Mandraveli K, Nikolaidis P, Alexiou-Daniel S, Arvanitidou-Vagiona M. Seroprevalence of Coxiella burnetii in a healthy population from northern Greece. Clinical Microbiology and Infection. 2009;15:148-9.
[841] Pape M, Xanthis A, Hatzitolios A, Mandraveli K, Savopoulos C, Alexiou-Daniel S. Acute

hepatitis associated with Q fever in a man in Greece: a case report. Journal of medical case reports. 2007;1:154-.
[842] Paraense WL. The schistosome vectors in the Americas. Memórias do Instituto Oswaldo Cruz. 2001;96(suppl):7-16.
[843] Parkinson M, Vuyyuru S, Patel J, Animalu C. Challenges of Diagnosing Severe Ehrlichiosis in Orthotopic Liver Transplant Recipients. Case Rep Transplant. 2021;2021:8285326.
[844] Parlak E, Birdal O, Ceviz M. Nosocomial Valve Endocarditis after Crimean-Congo Hemorrhagic Fever. Revista Da Sociedade Brasileira De Medicina Tropical. 2022;55.
[845] Parlak M, Bayram Y, ÇIkman A, Ceylan N, BerktaŞ M. Seropositivity of Borrelia burgdorferi in Risky Groups in Van Region, Turkey. Mikrobiyoloji Bulteni. 2015;49(3):439-45.
[846] Parola P, Fenollar F, Badiaga S, Brouqui P, Raoult D. First Documentation ofRickettsia conoriiInfection (Strain Indian Tick Typhus) in a Traveler. Emerging Infectious Diseases. 2001;7(5):909-10.
[847] Parola P, Rovery C, Rolain JM, Brouqui P, Davoust B, Raoult D. Rickettsia slovaca and R. raoultii in tick-borne Rickettsioses. Emerging Infectious Diseases. 2009;15(7):1105-8.
[848] Patel AA, Dalal YD, Parikh A, Gandhi R, Shah A. Crimean-Congo Hemorrhagic Fever: An Emerging Viral Infection in India, Revisited and Lessons Learned. Cureus Journal of Medical Science. 2023;15(8).
[849] Patel KM, Johnson J, Zacharioudakis IM, Boxerman JL, Flanigan TP, Reece RM. First confirmed case of Powassan neuroinvasive disease in Rhode Island. IDCases. 2018;12:84-7.
[850] Patel TP, Beck P, Chairman D, Regunath H. Ehrlichiosis Presenting as Hemophagocytic Lymphohistiocytosis in an Immunocompetent Adult. IDCases. 2020;20:e00813.
[851] Pavia CS, Plummer MM, Varantsova A. An Unusual Case of Serologically Confirmed Post-Partum Lyme Disease Following an Asymptomatic <i>Borrelia burgdorferi</i> Infection Acquired during Pregnancy and Lacking Vertical Transmission in Utero. Pathogens. 2024;13(3).
[852] Pavletic AJ, Marques AR. Early Disseminated Lyme Disease Causing False-Positive Serology for Primary Epstein-Barr Virus Infection: Report of 2 Cases. Clinical Infectious Diseases. 2017;65(2):336-7.
[853] Pence R, Johnston B. Syncope: A rare presentation of lyme disease. Journal of Emergency Medicine. 2023;65(1):E23-E6.
[854] Penev DG, Laurent E, Baron S, Diot E, Bastides F, de Gialluly C, et al. Lyme borreliosis: Census of adult patients hospitalized in Indre-et-Loire (France), from the Hospital Discharge Data (1999-2006). Revue D Epidemiologie Et De Sante Publique. 2010;58(5):339-47.
[855] Perk O, Emeksiz S, Ozcan S, Meral G. Crimean-Congo hemorrhagic fever: A pediatric case responding to plasmapheresis treatment. Transfus Apher Sci. 2021;60(6):103215.
[856] Perthame E, Chartier L, George J-C, Varloud M, Ferquel E, Choumet V. Case presentation and management of Lyme disease patients: a 9-year retrospective analysis in France. Frontiers in Medicine. 2024;10.
[857] faff F, François A, Hommel D, Jeanne I, Margery J, Guillot G, et al. Q Fever in French Guiana: New Trends. Emerging Infectious Diseases. 1998;4(1):131-2.
[858] Piantadosi A, Kanjilal S, Ganesh V, Khanna A, Hyle EP, Rosand J, et al. Rapid Detection of Powassan Virus in a Patient With Encephalitis by Metagenomic Sequencing. Clinical Infectious Diseases. 2018;66(5):789-92.

[859] Pick N, Potasman I, Strenger C, Keysary A, Schwartz I. Ehrlichiosis associated vasculitis. Journal of Internal Medicine. 2000;247(6):674-8.

[860] Pinar O, Ozgur AE, Esragul A, Halil Y, Gulcan KY, Ayse E, et al. High serum levels of neopterin in patients with Crimean-Congo hemorrhagic fever and its relation with mortality. Journal of Infection. 2008;56(5):366-70.

[861] Platonov AE, Karan LS, Kolyasnikova NM, Makhneva NA, Toporkova MG, Maleev VV, et al. Humans infected with relapsing fever spirochete Borrelia miyamotoi, Russia. Emerging Infectious Diseases. 2011;17(10):1816-23.

[862] Popivanova N, Hristova D, Hadjipetrova E. Guillain-Barré Polyneuropathy Associated with Mediterranean Spotted Fever: Case Report. Clinical Infectious Diseases. 1998;27(6):1549-.

[863] Poponnikova TV. Specific clinical and epidemiological features of tick-borne encephalitis in Western Siberia. International Journal of Medical Microbiology. 2006;296:59-62.

[864] Porath A, Schlaeffer F, Lieberman D. The epidemiology of community-acquired pneumonia among hospitalized adults. Journal of Infection. 1997;34(1):41-8.

[865] Pouquet M, Bareille N, Guatteo R, Moret L, Beaudeau F. Coxiella burnetii infection in humans: to what extent do cattle in infected areas free from small ruminants play a role? . Epidemiology and Infection. 2020;148.

[866] Pradeep J, Kumar S, Stephen S, Kamboj DV, Gunasekaran D, Hanifah M. Detection of acute Q fever human cases by indirect immunofluorescence real-time polymerase chain reaction in a tertiary care hospital in Puducherry. Indian Journal of Medical Research. 2018;148(4):449-52.

[867] Pradeep J, Stephen S, Ambroise S, Gunasekaran D. Diagnosis of Acute Q Fever by Detection of Coxiella burnetii DNA using Real-Time PCR, Employing a Commercial Genesig Easy Kit. Journal of Clinical and Diagnostic Research. 2017;11(9):DC10-DC3.

[868] Primus S, Akoolo L, Schlachter S, Gedroic K, Rojtman AD, Parveen N. Efficient detection of symptomatic and asymptomatic patient samples for Babesia microti and Borrelia burgdorferi infection by multiplex qPCR. PLoS ONE. 2018;13(5):e0196748.

[869] Pritt BS, Fernholz EC, Replogle AJ, Kingry LC, Sciotto MP, Petersen JM. Borrelia mayonii - A cause of Lyme borreliosis that can be visualized by microscopy of thin blood films. Clin Microbiol Infect. 2022;28(6):823-4.

[870] Pritt BS, Mead PS, Johnson DKH, Neitzel DF, Respicio-Kingry LB, Davis JRP, et al. Identification of a novel pathogenic Borrelia species causing Lyme borreliosis with unusually high spirochaetaemia: a descriptive study. Lancet Infectious Diseases. 2016;16(5):556-64.

[871] Pritt BS, Sloan LM, Johnson DKH, Munderloh UG, Paskewitz SM, McElroy KM, et al. Emergence of a New Pathogenic Ehrlichia Species, Wisconsin and Minnesota, 2009. New England Journal of Medicine. 2011;365(5):422-9.

[872] Psaroulaki A, Chochlakis D, Ioannou I, Florentia A, Gikas A, Tselentis Y. Acute anaplasmosis in humans in Cyprus. Clinical Microbiology and Infection. 2009;15:10-1.

[873] Psevdos G, Khoo T, Chow R, Romano CL, Campbell S. Epidemiology of Lyme disease among US Veterans in Long Island, New York. Ticks and Tick-Borne Diseases. 2019;10(2):407-11.

[874] Pshenichnaya NY, Nenadskaya SA. Probable Crimean-Congo hemorrhagic fever virus transmission occurred after aerosol-generating medical procedures in Russia: nosocomial cluster. International Journal of Infectious Diseases. 2015;33:120-2.

[875] Pshenichnaya NY, Sydenko IS, Klinovaya EP, Romanova EB, Zhuravlev AS. Possible sexual

transmission of Crimean-Congo hemorrhagic fever. Int J Infect Dis. 2016;45:109-11.
[876] Puges M, Berard X, Caradu C, Ducours M, Eldin C, Carrer M, et al. Polymicrobial Infections Among Patients with Vascular Q Fever, France, 2004-2020. Emerging Infectious Diseases. 2021;27(7):1961-3.
[877] Pun SB, Agrawal S, Jha S, Bhandari LN, Chalise BS, Mishra A, et al. First report of Lyme disease in Nepal. JMM case reports. 2018;5(3):e005128-e.
[878] Qaderi S, Hatami H, Omid AM, Sayad J. Vaginal bleeding as a sign of Crimean-Congo hemorrhagic fever infection: a case report. Journal of Medical Case Reports. 2022;16(1).
[879] Qaderi S, Mardani M, Shah A, Shah J, Bazgir N, Sayad J, et al. Crimean-Congo Hemorrhagic Fever (CCHF) in Afghanistan: A retrospective single center study. International Journal of Infectious Diseases. 2021;103:323-8.
[880] Qin S, Zhang W, Chen F, Luo F, Zhou Q, Ke P, et al. Antibodies against atypical pathogens and respiratory viruses detected by Pneumoslide IgM test in adults with community-acquired pneumonia in Guangzhou City. Journal of Clinical Laboratory Analysis. 2020;34(9).
[881] Qorbani A, Khalili M, Nourollahifard S, Mostafavi E, Farrokhnia M, Esmaeili S. An update on spotted fever group serology in Kerman Province, Iran. Comparative Immunology Microbiology and Infectious Diseases. 2022;88.
[882] Rachman M, Garfield DAS. Lyme disease and secondary depression: Universal lessons from an uncommon case. Psychosomatics. 1998;39(3):301-2.
[883] Raffetin A, Schemoul J, Chahour A, Nguala S, Caraux-Paz P, Paoletti G, et al. Multidisciplinary Management of Suspected Lyme Borreliosis: Clinical Features of 569 Patients, and Factors Associated with Recovery at 3 and 12 Months, a Prospective Cohort Study. Microorganisms. 2022;10(3).
[884] Rafik R, Hachimi M, Ouarssani A, Haouri M, Rouimi A. [Acute polyradiculoneuropathy and Rickettsia conorii infection]. Med Mal Infect. 2011;41(10):553-5.
[885] Rahden P, Adam A, Mika A, Jassoy C. Elevated Human Crimean-Congo Hemorrhagic Fever Virus Seroprevalence in Khashm el Girba, Eastern Sudan. American Journal of Tropical Medicine and Hygiene. 2019;100(6):1549-51.
[886] Rainey JJ, Siesel C, Guo X, Yi L, Zhang Y, Wu S, et al. Etiology of acute febrile illnesses in Southern China: Findings from a two-year sentinel surveillance project, 2017-2019. PLoS ONE. 2022;17(6).
[887] Ramsey AH, Belongia EA, Gale CM, Davis JP. Outcomes of Treated Human Granulocytic Ehrlichiosis Cases. Emerging Infectious Diseases. 2002;8(4):398-401.
[888] Randazzo JP, DiSpaltro FX, Cottrill C, Klainer AS, Steere AC, Bisaccia E. Successful treatment of a patient with chronic Lyme arthritis with extracorporeal photochemotherapy. Journal of the American Academy of Dermatology. 1994;30(5):908-10.
[889] Raoult D, Casalta JP, Richet H, Khan M, Bernit E, Rovery C, et al. Contribution of systematic serological testing in diagnosis of infective endocarditis. Journal of Clinical Microbiology. 2005;43(10):5238-42.
[890] Raoult D, Fenollar F, Stein A. Q Fever During Pregnancy. Archives of Internal Medicine. 2002;162(6).
[891] Raoult D, Fournier P-E, Abboud P, Caron F. First Documented HumanRickettsia aeschlimanniiInfection. Emerging Infectious Diseases. 2002;8(7):748-9.

[892] Raoult D, Fournier PE, Fenollar F, Jensenius M, Prioe T, de Pina JJ, et al. Rickettsia africae,a Tick-Borne Pathogen in Travelers to Sub-Saharan Africa. New England Journal of Medicine. 2001;344(20):1504-10.

[893] Rasikh AS, Aram MM, Noory AT. Clinical and Epidemiological Characteristics of 30 Fatal Cases of Crimean-Congo Hemorrhagic Fever in Kabul, Afghanistan: A Retrospective Observational Study. Infection and Drug Resistance. 2023;16:3469-76.

[894] Rau A, Munoz-Zanzi C, Schotthoefer AM, Oliver JD, Berman JD. Spatio-Temporal Dynamics of Tick-Borne Diseases in North-Central Wisconsin from 2000-2016. International Journal of Environmental Research and Public Health. 2020;17(14).

[895] Rawlins ML, Gerstner C, Hill HR, Litwin CM. Evaluation of a western blot method for the detection of Yersinia antibodies: evidence of serological cross-reactivity between Yersinia outer membrane proteins and Borrelia burgdorferi. Clinical and Diagnostic Laboratory Immunology. 2005;12(11):1269-74.

[896] Rehman K, Bettani MAK, Veletzky L, Afridi S, Ramharter M. Outbreak of Crimean-Congo haemorrhagic fever with atypical clinical presentation in the Karak District of Khyber Pakhtunkhwa, Pakistan. Infectious Diseases of Poverty. 2018;7.

[897] Reisfeld S, Mhamed SH, Stein M, Chowers M. Epidemiological, clinical and laboratory characteristics of acute Q fever in an endemic area in Israel, 2006-2016. Epidemiology and Infection. 2019;147.

[898] Renard C, Marignier S, Gillet Y, Roure-Sobas C, Guibaud L, Portes VD, et al. Acute hemiparesis revealing a neuroborreliosis in a child. Archives De Pediatrie. 2008;15(1):41-4.

[899] Rey D, Obadia Y, Tissot-Dupont H, Raoult D. Seroprevalence of antibodies to Coxiella burnetti among pregnant women in South Eastern France. European Journal of Obstetrics & Gynecology and Reproductive Biology. 2000;93(2):151-6.

[900] Riegodedios AJ, Ajene A, Malakooti MA, Gaydos JC, MacIntosh VH, Bohnker BK. Comparing Diagnostic Coding and Laboratory Results. Emerging Infectious Diseases. 2005;11(7):1151-3.

[901] Rigaud E, Jaulhac B, Garcia-Bonnet N, Hunfeld KP, Femenia F, Huet D, et al. Seroprevalence of seven pathogens transmitted by the Ixodes ricinus tick in forestry workers in France. Clinical Microbiology and Infection. 2016;22(8).

[902] Rohrbach BW, Harkess JR, Ewing SA, Kudlac J, McKee GL, Istre GR. Epidemiologic and clinical characteristics of persons with serologic evidence of E. canis infection. American Journal of Public Health. 1990;80(4):442-5.

[903] Rose CD, Fawcett PT, Klein JD, Eppes SC, Caputo GM, Doughty RA. Reinfection in paediatric Lyme borreliosis. Annals of the Rheumatic Diseases. 1993;52(9):695-6.

[904] Rosenberg R, Lindsey NP, Fischer M, Gregory CJ, Hinckley AF, Mead PS, et al. Vital Signs: Trends in Reported Vectorborne Disease Cases — United States and Territories, 2004–2016. MMWR Morbidity and Mortality Weekly Report. 2018;67(17):496-501.

[905] Rovery C, Granel B, Casalta JP, Lepidi H, Habib G, Raoult D. Coinfection with Coxiella burnetii in infectious endocarditis. Clinical Microbiology and Infection. 2009;15:190-1.

[906] Royal J, Riddle MS, Mohareb E, Monteville MR, Porter CK, Faix DJ. Seroepidemiologic survey for Coxiella burnetii among US military personnel deployed to Southwest and Central Asia in 2005. American Journal of Tropical Medicine and Hygiene. 2013;89(5):991-5.

[907] Rubio LA, Kjemtrup AM, Marx GE, Cronan S, Kilonzo C, Saunders MEM, et al. Borrelia

miyamotoi Infection in Immunocompromised Man, California, USA, 2021. Emerging Infectious Diseases. 2023;29(5):1011-4.

[908] Rudenko N, Golovchenko M, Vancova M, Clark K, Grubhoffer L, Oliver JH, Jr. Isolation of live Borrelia burgdorferi sensu lato spirochaetes from patients with undefined disorders and symptoms not typical for Lyme borreliosis. Clinical Microbiology and Infection. 2016;22(3).

[909] Russell A, Prusinski M, Sommer J, O'Connor C, White J, Falco R, et al. Epidemiology and Spatial Emergence of Anaplasmosis, New York, USA, 2010.2018. Emerging Infectious Diseases. 2021;27(8):2154-62.

[910] Ryan ME, Still MM, Fleetwood MK, Bross J, Wood GC, Frey CM. Prevalence of Antibody to Borrelia Burgdorferi in an Outpatient Setting in Central and Northeastern Pennsylvania. Infectious Diseases in Clinical Practice. 1999;8(7):341-5.

[911] Sabzevari S, Shoraka H, Seyyedin M. Seroepidemiological survey of brucellosis and Q fever among high-risk occupations in northeast of Iran for first time. Iranian Journal of Microbiology. 2021.

[912] Sadeghi M, Asgharzadeh SA, Bayani M, Alijanpour E, Javaniyan M, Jabbari A. Crimean congo hemorrhagic fever appearance in the north of Iran. Caspian Journal of Internal Medicine. 2013;4(1):617-20.

[913] Saglam B, Albayrak M, Acar A, Yildiz A, Maral S, Tiglioglu M, et al. Q fever as a rare cause of hemophagocytic lymphohistiocytosis: Case report. Transfusion and Apheresis Science. 2020;59(4).

[914] Sah R, Mohanty A, Mehta V, Chakraborty S, Chakraborty C, Dhama K. Crimean-Congo haemorrhagic fever (CCHF) outbreak in Iraq: Currently emerging situation and mitigation strategies - Correspondence. International Journal of Surgery. 2022;106.

[915] Saha A, Browning C, Dandamudi R, Barton K, Graepel K, Cullity M, et al. Donor-derived Ehrlichiosis: 2 Clusters Following Solid Organ Transplantation. Clinical Infectious Diseases. 2022;74(5):918-23.

[916] Sahak MN, Arifi F, Saeedzai SA. Descriptive epidemiology of Crimean-Congo Hemorrhagic Fever (CCHF) in Afghanistan: Reported cases to National Surveillance System, 2016-2018. International Journal of Infectious Diseases. 2019;88:135-40.

[917] Sahay RR, Dhandore S, Yadav PD, Chauhan A, Bhatt L, Garg V, et al. Detection of African genotype in Hyalomma tick pools during Crimean Congo hemorrhagic fever outbreak, Rajasthan, India, 2019. Virus Research. 2020;286.

[918] Sahay RR, Shete AM, Yadav PD, Patil S, Majumdar T, Jain R, et al. Sequential determination of viral load, humoral responses and phylogenetic analysis in fatal and non-fatal cases of Crimean-Congo hemorrhagic fever patients from Gujarat, India, 2019. Plos Neglected Tropical Diseases. 2021;15(8).

[919] Sahu R, Kale SB, Vergis J, Dhaka P, Kumar M, Choudhary M, et al. Apparent prevalence and risk factors associated with occurrence of Coxiella burnetii infection in goats and humans in Chhattisgarh and Odisha, India. Comparative Immunology Microbiology and Infectious Diseases. 2018;60:46-51.

[920] Saleem J, Usman M, Nadeem A, Sethi SA, Salman M. Crimean-Congo hemorrhagic fever: a first case from Abbottabad, Pakistan. International journal of infectious diseases : IJID : official publication of the International Society for Infectious Diseases. 2009;13(3):e121-3.

[921] Salehi-Vaziri M, Pouriayevali MH, Azad-Manjiri S, Vasmehjani AA, Baniasadi V, Fazlalipour M. The Seroprevalence of Tick-Borne Encephalitis in Rural Population of Mazandaran Province, Northern Iran (2018 - 2019). Archives of Clinical Infectious Diseases. 2020;15(1).

[922] Salehi-Vaziri M, Salmanzadeh S, Baniasadi V, Jalali T, Mohammadi T, Azad-Manjiri S, et al. An Outbreak of Crimean-Congo Hemorrhagic Fever in the SouthWest of Iran. Jundishapur Journal of Microbiology. 2017;10(1).

[923] Salih N, Baig KS, Jan MA, Ihtisham M, Ahmad F, Ghani N, et al. Crimean-Congo Hemorrhagic Fever Presented in Dengue Epidemic: A Case Report. Cureus Journal of Medical Science. 2023;15(5).

[924] Sargianou M, Panos G, Tsatsaris A, Gogos C, Papa A. Crimean-Congo hemorrhagic fever: seroprevalence and risk factors among humans in Achaia, western Greece. International Journal of Infectious Diseases. 2013;17(12):E1160-E5.

[925] Sari İ, Bakır S, Engin A, Aydın H, Poyraz Ö. Some acute phase reactants and cholesterol levels in serum of patient with Crimean-Congo haemorrhagic fever. Bosnian Journal of Basic Medical Sciences. 2013;13(1).

[926] Sarksyan DS, Platonov AE, Karan LS, Shipulin GA, Sprong H, Hovius JWR. Probability of SpirocheteBorrelia miyamotoiTransmission from Ticks to Humans. Emerging Infectious Diseases. 2015;21(12):2273-4.

[927] Sartin JS, Oettel KR. A morphealike skin condition caused by Borrelia burgdorferi in an immunocompromised patient. Mayo Clinic Proceedings. 2006;81(9):1259-+.

[928] Sathi S, Kim D, Duplan P, Kim P, Shenkamn C. Ischemic Stroke With Hemorrhagic Conversion in a Case of Lyme Neuroborreliosis. Cureus. 2022;14(8):e28028.

[929] Sauer A, Speeg-Schatz C, Hansmann Y. Two cases of orbital myositis as a rare feature of lyme borreliosis. Case Rep Infect Dis. 2011;2011:372470.

[930] Sayfullin RF, Perekopskaya NE, Karan LS, Zvereva NN, Sayfullin MA. Autochthonous Case of Rickettsia slovaca Infection in Russia. Emerging Infectious Diseases. 2021;27(10):2736-8.

[931] Schick S, Quigley R, Koenig ZA, McCarthy R. Jaw Pain and Profound Bradycardia - An Atypical Presentation of Lyme Carditis. Cureus. 2020;12(11):e11607.

[932] Schmulewitz L, Moumile K, Patey-Mariaud de Serre N, Poirée S, Gouin E, Mechaï F, et al. Splenic Rupture and Malignant Mediterranean Spotted Fever. Emerging Infectious Diseases. 2008;14(6):995-7.

[933] Schotthoefer AM, Meece JK, Ivacic LC, Bertz PD, Zhang K, Weiler T, et al. Comparison of a Real-Time PCR Method with Serology and Blood Smear Analysis for Diagnosis of Human Anaplasmosis: Importance of Infection Time Course for Optimal Test Utilization. Journal of Clinical Microbiology. 2013;51(7):2147-53.

[934] Schutzer SE, Luan J, Coyle PK. Detection of Lyme Disease after OspA Vaccine. New England Journal of Medicine. 1997;337(11):794-5.

[935] Schwartz AM, Hinckley AF, Mead PS, Hook SA, Kugeler KJ. Surveillance for Lyme Disease — United States, 2008–2015. MMWR Surveillance Summaries. 2017;66(22):1-12.

[936] Schwartz BS, Goldstein MD, Childs JE. Antibodies to Borrelia burgdorferi and tick salivary gland proteins in New Jersey outdoor workers. American Journal of Public Health. 1993;83(12):1746-8.

[937] Scribner J, Wu B, Lamyaithong A, Arcega V, Villanueva DD. Anaplasmosis-Induced

Hemophagocytic Lymphohistiocytosis: A Case Report and Review of the Literature. Open Forum Infect Dis. 2023;10(5):ofad213.

[938] Scrimgeour EM, Johnston WJ, Al Dhahry SHS, El-Khatim HS, John V, Musa M. First Report of Q Fever in Oman. Emerging Infectious Diseases. 2000;6(1):74-9.

[939] Scrimgeour EM, Zaki A, Mehta FR, Abraham AK, Al-Busaidy S, El-Khatim H, et al. Crimean-Congo haemorrhagic fever in Oman. Transactions of the Royal Society of Tropical Medicine and Hygiene. 1996;90(3):290-1.

[940] Seinost G, Golde WT, Berger BW, Dunn JJ, Qiu D, Dunkin DS, et al. Infection With Multiple Strains of Borrelia burgdorferi Sensu Stricto in Patients With Lyme Disease. Archives of Dermatology. 1999;135(11).

[941] Selim MME, Elbashier AM, Awad AI, Borgio F. Erythema Migrans - Case Report From Dammam Central Hospital. Annals of Saudi Medicine. 1994;14(6):521-2.

[942] Septfons A, Goronflot T, Jaulhac B, Roussel V, De Martino S, Guerreiro S, et al. Epidemiology of Lyme borreliosis through two surveillance systems: the national Sentinelles GP network and the national hospital discharge database, France, 2005 to 2016. Eurosurveillance. 2019;24(11):6-16.

[943] Serindağ HC, Çoban E, Kaykı Y, Soysal A. A Neuroborreliosis Case Presenting with Asymmetric Painful Radiculoneuritis. Turkish Journal Of Neurology. 2018;24(4):334-6.

[944] Sessa C, Vokrri L, Porcu P, Maurin M, Stahl JP, Magne JL. Abdominal aortic aneurysm and Coxiella burnetii infection: report of three cases and review of the literature. J Vasc Surg. 2005;42(1):153-8.

[945] Seuge L, Fischbach M, Laugel V, Lipsker D. Children Lyme Borreliosis: retrospective study of 16 cases. Presse medicale (Paris, France : 1983). 2011;40(9 Pt 1):e359-64.

[946] Sexton DJ, Corey GR, Carpenter C, Kong LQ, Gandhi T, Breitschwerdt E, et al. Dual Infection with Ehrlichia chaffeensis and a Spotted Fever Group Rickettsia: A Case Report. Emerging Infectious Diseases. 1998;4(2):311-6.

[947] Shah JS, Cruz ID, Ward S, Harris NS, Ramasamy R. Development of a sensitive PCR-dot blot assay to supplement serological tests for diagnosing Lyme disease. European Journal of Clinical Microbiology & Infectious Diseases. 2018;37(4):701-9.

[948] Shah V, Vaidya V, Bang V, Shah I. Spotted fever in a child in Mumbai, India. Journal of Vector Borne Diseases. 2009;46(4):310-2.

[949] Shahhosseini N, Chinikar S, Shams E, Nowotny N, Fooks AR. Crimean-Congo hemorrhagic fever cases in the North of Iran have three distinct origins. Virusdisease. 2017;28(1):50-3.

[950] Shahid MF, Shabbir MZ, Ashraf K, Ali M, Yaqub S, Ul-Rahman A, et al. Sero-Epidemiological Survey of Crimean-Congo Hemorrhagic Fever among the Human Population of the Punjab Province in Pakistan. Virologica Sinica. 2020;35(4):486-9.

[951] Shaker DA, Abd MT, Alsalih NJ, Mahdi SG, Alsaadawi M, Aakef IR, et al. A retrospective study of Crimean-Congo hemorrhagic fever in Iraq. African Health Sciences. 2024;24(1):59-68.

[952] Shapiro ED. Long-Term Outcomes of Persons with Lyme Disease. Vector-Borne and Zoonotic Diseases. 2002;2(4):279-81.

[953] Sharabi S, Sagi O, Ben-Shimol S. Serologic Diagnosis of Acute Rickettsiosis in Children in Southern Israel. Pediatric Infectious Disease Journal. 2021;40(12):E521-E3.

[954] Sharifi-Mood B, Metanat M, Alavi-Naini R. Prevalence of crimean-congo hemorrhagic Fever

among high risk human groups. International journal of high risk behaviors & addiction. 2014;3(1):e11520-e.
[955] Sharififard M, Alavi SM, Salmanzadeh S, Safdari F, Kamali A. Epidemiological Survey of Crimean-Congo Hemorrhagic Fever (CCHF), a Fatal Infectious Disease in Khuzestan Province, Southwest Iran, During 1999-2015. Jundishapur Journal of Microbiology. 2016;9(5).
[956] Shayan S, Bokaean M, Shahrivar MR, Chinikar S. Crimean-Congo Hemorrhagic Fever. Laboratory Medicine. 2015;46(3):180-9.
[957] Shazberg G, Moise J, Terespolsky N, Hurvitz H. Family Outbreak of Rickettsia conorii Infection. Emerging Infectious Diseases. 1999;5(5):723-4.
[958] Sheikh AS, Sheikh AA, Sheikh NS, Rafi US, Asif M, Afridi F, et al. Bi-annual surge of Crimean-Congo haemorrhagic fever (CCHF): a five-year experience. International Journal of Infectious Diseases. 2005;9(1):37-42.
[959] Shih C-M, Wang J-C, Chao L-L, Wu T-N. Lyme Disease in Taiwan: First Human Patient with Characteristic Erythema Chronicum Migrans Skin Lesion. Journal of Clinical Microbiology. 1998;36(3):807-8.
[960] Shin A, Tukhanova N, Ndenkeh J, Jr., Shapiyeva Z, Yegemberdiyeva R, Yeraliyeva L, et al. Tick-borne encephalitis virus and West-Nile fever virus as causes of serous meningitis of unknown origin in Kazakhstan. Zoonoses and Public Health. 2022;69(5):514-25.
[961] Shirley JD, Ngo TT, Patel JA, Pritt BS, Gaensbauer JT, Theel ES, et al. The Brief Case: An unexpected cause of meningoencephalitis in an infant. Journal of Clinical Microbiology. 2023;61(11).
[962] Sidira P, Maltezou HC, Haidich AB, Papa A. Seroepidemiological study of Crimean-Congo haemorrhagic fever in Greece, 2009-2010. Clinical Microbiology and Infection. 2012;18(2):E16-E9.
[963] Sidira P, Nikza P, Danis K, Panagiotopoulos T, Samara D, Maltezou H, et al. Prevalence of Crimean-Congo hemorrhagic fever virus antibodies in Greek resi dents in the area where the AP92 strain was isolated. Hippokratia. 2013;17(4):322-5.
[964] Sieg C. Bester Weiterbildungsbeitrag prämiert. Der Internist. 2010;51(8):1070-2.
[965] Sinha P, Oberoi B, Sirohi YS, Sood A, Bhattacharjee S. A Case Report of Early Disseminated Lyme Disease. Neurol India. 2020;68(4):916-8.
[966] Sisman A. Epidemiologic Features and Risk Factors of Crimean-Congo Hemorrhagic Fever in Samsun Province, Turkey. Journal of Epidemiology. 2013;23(2):95-102.
[967] Smith RP, Jr., Elias SP, Borelli TJ, Missaghi B, York BJ, Kessler RA, et al. Human Babesiosis, Maine, USA, 1995-2011. Emerging Infectious Diseases. 2014;20(10):1727-30.
[968] Smith RP, Jr., Elias SP, Cavanaugh CE, Lubelczyk CB, Lacombe EH, Brancato J, et al. Seroprevalence of Borrelia burgdorferi, B. miyamotoi, and Powassan Virus in Residents Bitten by Ixodes Ticks, Maine, USA. Emerging Infectious Diseases. 2019;25(4):804-7.
[969] Solomon IH, Spera KM, Ryan SL, Helgager J, Andrici J, Zaki SR, et al. Fatal Powassan Encephalitis (Deer Tick Virus, Lineage II) in a Patient With Fever and Orchitis Receiving Rituximab. JAMA Neurol. 2018;75(6):746-50.
[970] Song D, Almas T, Abdelghffar M, Jain S, Geetha HS, Shah V, et al. A rare case of delayed anaplasma phagocytophilum-induced pancytopenia: A diagnostic conundrum. Ann Med Surg (Lond). 2022;75:103366.

[971] Sonsoz MR, Bali EA, Aydogan M, Mercanoglu F, Yavuz SS. Q fever endocarditis: is it always subacute or chronic? Turk Kardiyoloji Dernegi Arsivi-Archives of the Turkish Society of Cardiology. 2020;48(1):72-6.

[972] Spyridaki I, Gikas A, Kofteridis D, Psaroulaki A, Tselentis Y. Q Fever in the Greek Island of Crete: Detection, Isolation, and Molecular Identification of Eight Strains of Coxiella burnetii from Clinical Samples. Journal of Clinical Microbiology. 1998;36(7):2063-7.

[973] Stähelin-Massik J, Zimmermann H, Gnehm HE. Tick-Borne Encephalitis in Swiss Children 2000–2004. Pediatric Infectious Disease Journal. 2008;27(6):555-7.

[974] Standaert SM, Dawson JE, Schaffner W, Childs JE, Biggie KL, Singleton J, et al. Ehrlichiosis in a Golf-Oriented Retirement Community. New England Journal of Medicine. 1995;333(7):420-5.

[975] Steere AC, McHugh G, Damle N, Sikand VK. Prospective study of serologic tests for lyme disease. Clin Infect Dis. 2008;47(2):188-95.

[976] Stephen S, Gunasekaran D, Pradeep J, Broise SA, Sarangapani K. Serological Diagnosis of Indian Tick Typhus in and around Puducherry: Application of Indirect Immunofluorescence Assay. Journal of Clinical and Diagnostic Research. 2019;13(5):DC6-DC9.

[977] Straily A, Dahlgren FS, Peterson A, Paddock CD. Surveillance for Q Fever Endocarditis in the United States, 1999-2015. Clinical Infectious Diseases. 2017;65(11):1872-7.

[978] Strickland GT, Trivedi L, Watkins S, Clothier M, Grant J, Morgan J, et al. Cluster of Lyme Disease Cases at a Summer Camp in Kent County, Maryland. Emerging Infectious Diseases. 1996;2(1):44-6.

[979] Sudhindra P, Wang G, Schriefer ME, McKenna D, Jian Z, Krause PJ, et al. Insights into Borrelia miyamotoi infection from an untreated case demonstrating relapsing fever, monocytosis and a positive C6 Lyme serology. Diagnostic Microbiology and Infectious Disease. 2016;86(1):93-6.

[980] Sun R-X, Lai S-J, Yang Y, Li X-L, Liu K, Yao H-W, et al. Mapping the distribution of tick-borne encephalitis in mainland China. Ticks and Tick-Borne Diseases. 2017;8(4):631-9.

[981] Sun S, Dai X, Aishan M, Wang X, Meng W, Feng C, et al. Epidemiology and Phylogenetic Analysis of Crimean-Congo Hemorrhagic Fever Viruses in Xinjiang, China. Journal of Clinical Microbiology. 2009;47(8):2536-43.

[982] Sun W-W, Cong W, Li M-H, Wang C-F, Shan X-F, Qian A-D. Coxiella burnetii Seroprevalence and Risk Factors in Cattle Farmers and Farm Residents in Three Northeastern Provinces and Inner Mongolia Autonomous Region, China. Biomed Research International. 2016;2016.

[983] Sundheim KM, Levas MN, Balamuth F, Thompson AD, Neville DN, Garro AC, et al. Seasonality of Acute Lyme Disease in Children. Tropical Medicine and Infectious Disease. 2021;6(4).

[984] Sung S, Wurcel AG, Whittier S, Kulas K, Kramer LD, Flam R, et al. Powassan Meningoencephalitis, New York, New York, USA. Emerging Infectious Diseases. 2013;19(9):1504-6.

[985] Surgers L, Belkadi G, Foucard A, Lalande V, Girard PM, Hennequin C. Babesiosis and Lyme disease co-infection in a female patient returning from the United States. Medecine Et Maladies Infectieuses. 2015;45(11-12):490-2.

[986] Sweeney CJ, Ghassemi M, Agger WA, Persing DH. Coinfection with Babesia microti and Borrelia burgdorferi in a western Wisconsin resident. Mayo Clin Proc. 1998;73(4):338-41.

[987] Trobe JD. Thieme Atlas of Anatomy: Head and Neuroanatomy. Journal of Neuro-Ophthalmology. 2007;27(4).

[988] Van Deynze A, Stoffel K, Buell CR, Kozik A, Liu J, van der Knaap E, et al. Diversity in conserved genes in tomato. BMC Genomics. 2007;8(1).

[989] Wang J, Gao S, Zhang S, He X, Liu J, Liu A, et al. Rapid detection of Babesia motasi responsible for human babesiosis by cross-priming amplification combined with a vertical flow. Parasites & Vectors. 2020;13(1).

[990] He LF, Hou XX, Chen T, Zhang L, Wen S, Miao GQ, et al. Serological study of Lyme disease antibody in 2 311 patients with arthritis symptoms in Hainan Province. Zhonghua yu fang yi xue za zhi [Chinese journal of preventive medicine]. 2021;55(3):379-85.

[991] Weiss MD, Wasdell MB, Bomben MM, Rea KJ, Freeman RD. Sleep Hygiene and Melatonin Treatment for Children and Adolescents With ADHD and Initial Insomnia. J Am Acad Child Adolesc Psychiatry. 2006;45(5):512-9.

[992] Centers for Disease Control and Prevention (CDC). Human granulocytic ehrlichiosis--New York, 1995. MMWR Morb Mortal Wkly Rep. 1995;44(32):593-5.

[993] Centers for Disease Control and Prevention (CDC). Human ehrlichiosis--Maryland, 1994. MMWR Morb Mortal Wkly Rep. 1996;45(37):798-802.

[994] Centers for Disease Control and Prevention (CDC). Statewide surveillance for ehrlichiosis--Connecticut and New York, 1994-1997. MMWR Morb Mortal Wkly Rep. 1998;47(23):476-80.

[995] From the Centers for Disease Control and Prevention. Outbreak of Powassan encephalitis--Maine and Vermont, 1999-2001. JAMA. 2001;286(16):1962-3.

[996] Centers for Disease Control and Prevention (CDC). Q fever--California, Georgia, Pennsylvania, and Tennessee, 2000-2001. MMWR Morb Mortal Wkly Rep. 2002;51(41):924-7.

[997] Centers for Disease Control and Prevention (CDC). Anaplasmosis and ehrlichiosis - Maine, 2008. MMWR Morb Mortal Wkly Rep. 2009;58(37):1033-6.

[998] Centers for Disease Control and Prevention (CDC). Tick-borne encephalitis among U.S. travelers to Europe and Asia - 2000-2009. MMWR Morb Mortal Wkly Rep. 2010;59(11):335-8.

[999] Centers for Disease Control and Prevention (CDC). Notes from the field: Q fever outbreak associated with goat farms--Washington and Montana, 2011. MMWR Morb Mortal Wkly Rep. 2011;60(40):1393.

[1000] Centers for Disease Control and Prevention (CDC). West Nile virus disease and other arboviral diseases--United States, 2010. MMWR Morb Mortal Wkly Rep. 2011;60(30):1009-13.

[1001] Bental T, Fejgin M, Keysary A, Rzotkiewicz S, Oron C, Nachum R, et al. Chronic Q fever of pregnancy presenting as Coxiella burnetii placentitis: successful outcome following therapy with erythromycin and rifampin. Clin Infect Dis. 1995;21(5):1318-21.

[1002] Centers for Disease Control and Prevention (CDC). Outbreak of Powassan encephalitis--Maine and Vermont, 1999-2001. MMWR Morb Mortal Wkly Rep. 2001;50(35):761-4.

[1003] Centers for Disease Control and Prevention (CDC). Lyme disease--United States, 2001-2002. MMWR Morb Mortal Wkly Rep. 2004;53(17):365-9.

[1004] Arav-Boger R, Crawford T, Steere AC, Halsey NA. Cerebellar ataxia as the presenting manifestation of Lyme disease. Pediatr Infect Dis J. 2002;21(4):353-6.

[1005] Bacon RM, Kugeler KJ, Mead PS; Centers for Disease Control and Prevention (CDC). Surveillance for Lyme disease--United States, 1992-2006. MMWR Surveill Summ. 2008;57(10):1-9.

[1006] Halperin J, Luft BJ, Volkman DJ, Dattwyler RJ. Lyme neuroborreliosis. Peripheral nervous

system manifestations. Brain. 1990;113 (Pt 4):1207-21.
[1007] Centers for Disease Control and Prevention (CDC). Lyme disease--United States, 2003-2005. MMWR Morb Mortal Wkly Rep. 2007;56(23):573-6.
[1008] Centers for Disease Control and Prevention (CDC). Lyme disease--United States, 1994. MMWR Morb Mortal Wkly Rep. 1995;44(24):459-62.
[1009] Centers for Disease Control and Prevention (CDC). Lyme disease--United States, 1995. MMWR Morb Mortal Wkly Rep. 1996;45(23):481-4.
[1010] Centers for Disease Control and Prevention (CDC). Lyme disease -- United States, 1996. MMWR Morb Mortal Wkly Rep. 1997;46(23):531-5.
[1011] Centers for Disease Control and Prevention (CDC). Lyme disease--United States, 1999. MMWR Morb Mortal Wkly Rep. 2001;50(10):181-5.
[1012] Centers for Disease Control and Prevention (CDC). Lyme disease--United States, 2000. MMWR Morb Mortal Wkly Rep. 2002;51(2):29-31.
[1013] 阿不力提甫·阿不力孜, 努尔比亚·吾不力阿西木, 塔依尔·吾不力. 2003 年巴楚县 2 例新疆出血热病例诊治报告. 地方病通报. 2007(05):63.
[1014] 阿日棍通拉嘎, 张晓光, 韩淑祯. 蒙西医结合治疗重症森林脑炎 1 例报告. 中国民族民间医药. 2016;25(14):11-2.
[1015] 艾承绪, 温玉欣, 张永国, 邱贵城, 李得友, 史志学, 等. 莱姆病面神经麻痹. 中华神经精神科杂志. 1989;22(01):41-3.
[1016] 艾承绪, 温玉欣, 张永国. 莱姆病在我国的首次报告. 山东医科大学学报. 1987(02):1-4.
[1017] 敖玉霞. 森林脑炎患者心理护理干预的研究 [硕士]: 内蒙古民族大学; 2015.
[1018] 白海梅, 白乙拉. 蒙西医结合治疗森林脑炎. 中国民族医药杂志. 2007(03):27.
[1019] 白海燕. 森林脑炎患者气管切开后的护理 30 例分析. 中国保健营养. 2017;27(009):219-20.
[1020] 白洁, 李培奋, 何灵, 高晨, 哈小琴, 张全华. 9 项呼吸道病原体血清 IgM 抗体检测在呼吸道感染中的临床价值. 国际检验医学杂志. 2016;37(18):2573-5.
[1021] 白雪燕. 森林脑炎 1 例. 中国误诊学杂志. 2006(06):1208.
[1022] 包益平, 于学英. 3 例人粒细胞无形体病的护理. 中华护理杂志. 2009;44(7):662-3.
[1023] 鲍红霞. 1 例人粒细胞无形体病人的护理. 护理研究. 2010;24(33):3101.
[1024] 毕德增，陈振光，宋秀萍，赵晶，贺金荣，苑明远. 我国钮扣热感染的血清学证据. 疾病监测. 1995(08):237-9.
[1025] 边才, 柳朝阳, 张涛, 王琨, 李晓丹. 蜱传疾病 森林脑炎、莱姆病及斑点热多重感染病例分析. 世界最新医学信息文摘. 2017(17):137.
[1026] 蔡增林, 鲁志新, 胡玲美, 赵占林, 何亦祥. 东北地区人血清虫媒病毒抗体调查. 中国人兽共患病杂志. 1993(06):60-1.
[1027] 蔡增林，鲁志新，胡玲美，赵占林，金显涛，何亦祥. 东北三省部份地区森林脑炎疫源地流行病学调查. 微生物学杂志. 1996(01):19-22.
[1028] 曹磊, 许庆梅, 张悦. 早期心肌酶学监测对重症森林脑炎的预警研究. 世界最新医学信息文摘. 2017;17(45):97-8.
[1029] 曹明华, 刘红, 张永根, 史永林, 王俊, 张丽娟. 安徽省不同地区人群和家畜蜱源立克次体病血清流行病学调查. 安徽预防医学杂志. 2010;16(05):342-4.
[1030] 曾蓓伦, 张格庆, 吕铮, 崔久嵬. 临床确诊慢性 Q 热并发多系统功能损害一例. 中华传染病杂志. 2019;37(2):2.
[1031] 曾涛. 2019 年某院儿童 9 项呼吸道病原体 IgM 抗体检测结果分析. 医学信息.

2021;34(6):150-3.
[1032] 柴程良, 陆群英, 孙继民, 姜理平, 凌锋, 张丽娟, 等. 浙江省人和家畜蜱媒传染病血清流行病学调查. 中华流行病学杂志. 2010(10):4.
[1033] 产美英, 蒋惠荷, 陈艺林, 李英欣, 马登宏, 朱德明. 莱姆病周围性面瘫 9 例报告. 中国人兽共患病杂志. 1995;11(3):54-5.
[1034] 常爱娜, 李晨, 王海涛, 郑谊梅. 人粒细胞无形体病二例. 中华临床医师杂志(电子版). 2011;5(04):1234-5.
[1035] 常丙功, 张健之, 田小东, 蔡军, 陆振豸. 海南省琼中地区三农场斑点热血清流行病学研究. 中国媒介生物学及控制杂志. 1999(03):41-3.
[1036] 常利涛, 刀志宏, 梁长威, 李娟, 李云德, 赵景波, 等. 云南省人和家畜立克次体病血清流行病学调查. 中国人兽共患病学报. 2010;26(02):189-92+97.
[1037] 常素静, 韩澎湃, 韩淑祯. 森林脑炎颈肌瘫痪一例肌电图观察. 华西医学. 2009;24(11):3032.
[1038] 沈博, 柳鸿敏, 许爽, 吴东林, 李静, 李响, 等. 2012 年吉林省森林脑炎病例检验结果与分析. 中国卫生工程学. 2013;12(05):428-9+31.
[1039] 沈壮, 王劲, 张凤琴, 田兴宽, 高云鹏, 宋安乔. 北京西南部山区莱姆病调查. 中国公共卫生. 1993(S1):11-3.
[1040] 陈恒, 江立千, 李亚东, 万芳, 柯茂彬. 儿童急性呼吸道感染 9 种病原体的 IgM 抗体检测结果分析. 检验医学与临床. 2016;13(12):1665-6.
[1041] 陈红霞, 马玲敏, 黄艳, 王贞斐. 台州地区儿童呼吸道感染非典型病原体检出情况分析. 中国卫生检验杂志. 2014;24(09):1339-40+43.
[1042] 陈华俊. 小儿急性呼吸道感染 9 项病原体的感染情况及其分布特点. 广西医学. 2018;40(11):1185-8+99.
[1043] 陈炯然, 陈思东, 伍碧雯, 连建华, 林大能. 广东 Q 热调查研究. 广东医学. 1990(01):47-8.
[1044] 陈宁. 延边地区森林脑炎流行及临床表现分析. 中国当代医药. 2021;28(19):198-200+78.
[1045] 陈平, 吴文兵, 苟惠, 任思冲. 外周血宏基因组二代测序辅助诊断 Q 热血流感染 1 例. 中国医药导报. 2023;20(17):186-8+96.
[1046] 陈若雷. 莱姆病早期出现二期损伤病例报告及分析 2 例. 中国实用医药. 2010;5(26):188-9.
[1047] 陈素爱, 冯芳波, 杨应龙, 张微芬, 张首印, 徐继才, 等. 北京西北郊果树专业工人中的莱姆病. 北京医学. 1993(03):137-9.
[1048] 陈香蕊, 胥照平, 张永国, 汪民, 石建时, 徐毛华. 我国部分地区立克次体血清学的调查研究. 解放军预防医学杂志. 1988(04):15-8.
[1049] 陈兴, 胡嘉颖, 彭丹, 黄坚尧, 邓玉华. 急性呼吸道感染患儿病原学特点分析. 中国实用医药. 2019;14(17):47-9.
[1050] 陈映, 林永梅, 李洪. 1934 例呼吸道感染患者 9 种呼吸道病原体特征分析. 华南国防医学杂志. 2019;33(03):173-6.
[1051] 陈永亮, 杨育松, 贾丽丽, 耿利彬, 王娅琼, 郑钫华. 北京市密云县莱姆病血清流行病学调查. 首都公共卫生. 2016;10(02):83-5.
[1052] 陈勇. 山东省人粒细胞无形体病 4 例报道. 中国病原生物学杂志. 2009;4(11):796.
[1053] 陈育. 森林脑炎肝功能损伤的临床研究 [硕士]: 内蒙古民族大学; 2012.
[1054] 陈育, 张晓光, 韩淑祯. 森林脑炎并肝功能损伤 93 例临床观察. 中国现代药物应用. 2012;6(07):51-2.
[1055] 陈振光，毕德增，宋秀萍，潘亮，于恩庶，范明远. 福建宁化县斑点热的发现与研究. 中

国人兽共患病杂志. 1995(03):52-4.
[1056] 成守金. 间接免疫荧光法检测呼吸道九项病原体相关抗体及其临床价值的研究 [硕士]2016.
[1057] 成守金, 冯光安, 陈文洁. 间接免疫荧光法测定呼吸道 9 种病原体 IgM 结果分析. 中国临床研究. 2014;27(07):866-8.
[1058] 成守金, 许贺春, 赵丽萍. 11376 例呼吸道感染 9 种病原体检测结果分析. 实验与检验医学. 2020;38(3):533-6.
[1059] 程仕虎, 刘又宁, 李朝霞, 赵铁梅. 44 例被误诊为普通性肺炎的 Q 热柯克斯体肺炎病人的回顾性分析. 科学技术与工程. 2007(10):2335-9.
[1060] 程周祥, 杨小祥, 李群, 何建刚, 豆正东, 吴家兵, 等. 皖南地区 2006 年一起人传"粒细胞无形体病"疫情的流行病学特征. 中华疾病控制杂志. 2009;13(01):4-7.
[1061] 从日照. 森林脑炎患者血清 NSE 和 TNF-α 检测的临床意义 [硕士]: 内蒙古民族大学; 2012.
[1062] 丛日照, 韩淑祯, 孙亚男, 张晓光. 森林脑炎患者血清神经元特异性烯醇化酶检测的临床意义. 中国医学创新. 2012;9(12):83-5.
[1063] 丛日照, 孙亚男, 韩淑祯. 森林脑炎并发脓毒血症 1 例. 中外医学研究. 2012;10(12):155-6.
[1064] 崔峰, 王玲, 张玲, 杨淑霞, 张寿峰, 王加坤, 等. 山东省沂源县人粒细胞无形体病现场流行病学调查. 疾病监测. 2009;24(06):412-5.
[1065] 崔君兆. 广西人,畜 Q 热血清流行病学首次调查报告. 中国人兽共患病杂志. 1989(04):56.
[1066] 崔威武, 邓晶, 施世锋, 黄仁杰, 许珂, 黄诚孝, 等. 杭州市莱姆病血清流行病学调查. 中国预防医学杂志. 2007(04):382-6.
[1067] 崔伟华. 山东昌乐呼吸道感染常见病原体的检测与分析 [硕士]: 青岛大学; 2020.
[1068] 崔文治. 眼莱姆病例报告. 实用眼科杂志. 1993(06):50.
[1069] 崔晓鸣. 嗜吞噬细胞无形体及其复合感染的调查与实验研究 [博士]2016.
[1070] 崔宇晖, 王艳红, 宣群, 杨志芬, 何瑞云, 柳爱华, 等. 云南省某大学新生中嗜吞噬细胞无形体感染的血清流行病学调查. 中国医药科学. 2015;5(07):214-6.
[1071] 单咏梅, 周宏, 杨凡, 万海英. 呼吸道非典型病原体抗体实验室检测及病原分析. 国际检验医学杂志. 2013;34(17):2297-9.
[1072] 党秋菊, 崔家幸, 王茜, 田艳荣, 王立东, 张玉霞. 呼吸道病原体九联检在儿科呼吸道疾病中的临床应用. 中国医学工程. 2015;23(01):32-3.
[1073] 邓立权, 沈博, 李亚明. 2010～2016 年吉林省森林脑炎流行特征分析. 中国生物制品学杂志. 2017;30(10):1059-62.
[1074] 翟励敏, 王碧玉, 初礼巍, 赵丽春. 广西桂林地区儿童 9 种呼吸道病原体感染的检测结果分析. 现代检验医学杂志. 2020;35(5):147-50.
[1075] 丁大伟, 付维明, 黄玉明, 梁慧杰, 王延禄, 杨春江, 等. 黑龙江林区出入境人员嗜吞噬细胞无形体血清抗体检测. 中国国境卫生检疫杂志. 2010;33(04):221-3.
[1076] 董景红, 朱建华, 尹凤茹. 内蒙古大兴安岭林区森林脑炎、莱姆病流行病学调查研究. 中国预防医学杂志. 2007(06):718-9.
[1077] 董敏, 张晓军, 周厚清. 非典型呼吸道感染病原体检测在儿童急性呼吸道感染中的意义. 医药论坛杂志. 2012;33(05):15-6.
[1078] 杜红阳. 蜱传疾病多重感染病例分析. 兽医导刊. 2017(22):1.
[1079] 杜娟. 发热伴血小板减少综合征疫源地中斑点热群立克次体的调查研究 [硕士]2018.
[1080] 杜微. 人粒细胞无形体病 16 例临床护理. 齐鲁护理杂志. 2011;17(06):71-2.
[1081] 杜文军. 1 起人粒细胞无形体病的流行病学调查报告. 安徽预防医学杂志. 2008(05):372+92.

[1082] 杜勇. 黑龙江省苇河林区莱姆病调查报告. 中国人兽共患病杂志. 1991(04):36-7.
[1083] 范德生, 徐琪毅, 禹惠兰, 彭勇, 王誓闻, 张丽娟. 新疆伊犁地区人及家畜贝氏柯克斯体血清学调查. 疾病监测. 2011;26(01):15-7.
[1084] 范理铭. 以脑膜炎及脑膜脑炎为表现的莱姆病 2 例报告. 中国神经精神疾病杂志. 1992(05):289.
[1085] 冯崇慧. 抗体捕获 ELISA 检测 IgM 抗体在新疆出血热病人早期诊断中的应用. 地方病通报. 2004(S1):96-8.
[1086] 冯方波，张薇芬，周国萍，张首印，袁玉民，吴宜恕. 北京地区莱姆病的发现及临床研究. 中华流行病学杂志. 1994(01):10-3.
[1087] 冯梅, 蒋喜凤. 614 例住院患儿急性呼吸道感染病原学分析. 吉林医学. 2013;34(36):7611-2.
[1088] 付锦娴, 皮赛男, 陈友鹏. 深圳市慢性 Q 热心内膜炎 2 例. 中华传染病杂志. 2023;41(8):538-9.
[1089] 付宗强, 刘豆豆, 张汇征, 牛小斌, 李永伟. 郑州地区单中心住院患者呼吸道感染病原体的流行特征. 河南医学研究. 2020;29(01):1-5.
[1090] 高东旗, 曹务春, 张习坦, 赵秋敏, 朱建华. 内蒙古大兴安岭林区人埃立克体病自然疫源地的调查. 传染病信息. 2001(04):168-70.
[1091] 高东旗, 曹务春, 张习坦, 赵秋敏, 朱建华, 陈山虎,等. 大兴安岭地区人群埃立克体感染的调查. 中华流行病学杂志. 2001(02):57-61.
[1092] 高平, 刘银红. 莱姆病神经系统表现. 脑与神经疾病杂志. 1999(06):353-5.
[1093] 高雪. 对重症森林脑炎患者施以注射用人免疫球蛋白治疗方案的疗效研究. 中国保健营养. 2020;30(13):109-10.
[1094] 高原. 辽宁省新发蜱媒传染病的流行病学调查、分子生物学研究和临床特征分析 [硕士]2023.
[1095] 葛才荣, 齐玉琴. Q 热的多脏器损害. 金陵医院院刊. 1990(03):263-4.
[1096] 葛才荣, 齐玉琴. 江苏地区 Q 热一例报告. 1991 年 12 卷 1 期 19 页 MEDLINE ISTIC PKU CSCD. 2020.
[1097] 葛显成. 信阳市浉河区 2010 年人粒细胞无形体病流行病学分析. 中国公共卫生管理. 2012;28(03):304-5.
[1098] 耿娅萍, 崔红萍, 尹春琼, 白志瑶. 某院 2051 例患者呼吸道病原体九联检结果分析. 实用检验医师杂志. 2019;11(4):200-2.
[1099] 耿震, 侯学霞, 郭建华, 黄鑫, 王春生, 王博, 等. 吉林省长白及通化县莱姆病流行病学调查. 中国媒介生物学及控制杂志. 2010;21(06):572-5.
[1100] 耿震, 侯学霞, 郝琴, 胡桂兰, 万康林. 827 例拟诊莱姆病患者抗伯氏疏螺旋体抗体检查结果分析. 中国媒介生物学及控制杂志. 2007(03):219-21.
[1101] 耿震, 侯学霞, 万康林, 郝琴. 105 例拟诊莱姆病患者抗伯氏疏螺旋体抗体检查结果分析. 中国媒介生物学及控制杂志. 2010;21(01):65-7.
[1102] 耿震, 李国华, 侯学霞, 张琳, 郝琴. 山西省首例莱姆病病例分析. 中国媒介生物学及控制杂志. 2014;25(4):318-9,22.
[1103] 龚慧, 杜冀晖, 高灵莉, 李一凡, 麦丽文, 周蓓, 等. 深圳市 5918 例呼吸道感染患者 9 种呼吸道病原体 IgM 抗体检测分析. 国际检验医学杂志. 2017;38(19):2657-9+62.
[1104] 谷存国, 李雅江, 潘宝山. 应用 ELISA 技术检测黑河林区人群莱姆病螺旋体抗体. 黑龙江医药科学. 2003;26(1):63-.
[1105] 归巧娣, 宫艳艳, 范芸, 武建财. 7363 例呼吸道九项病原体 IgM 抗体检测结果分析. 医学

动物防制. 2021;37(02):146-8.
[1106] 郭玲, 郝凯军, 张增梅. 1例人粒细胞无形体伴多脏器功能衰竭患者的护理. 中国医学创新. 2012;9(29):75-6.
[1107] 郭锐, 徐少泽, 许庆梅, 王晶. 养血清脑颗粒治疗森林脑炎抑郁病人头痛的疗效观察. 世界最新医学信息文摘（连续型电子期刊）. 2019;19(29):143-4.
[1108] 郭万申, 李林村, 张彦平, 韩唤霞, 李广伟, 夏占国, 等. 新安县莱姆病血清及病原学初探. 中国卫生检验杂志. 1995(06):355-6.
[1109] 郭伟, 戚丽君, 王菲, 王凤爽, 王红, 刘亚娇. 1 例森林脑炎气管切开术后患者的护理体会. 黑龙江医学. 2000(06):49.
[1110] 郭衍, 万康林, 许世锷, 张哲夫, 谢霖崇, 陈慎奔, 等. 粤东莱姆病疫源地的发现与研究. 中国人兽共患病杂志. 2000(02):42-5.
[1111] 韩丹, 黄义恒, 孙丽丽, 徐少泽. 浅谈森林脑炎患者的饮食护理. 医学信息. 2015;000(024):124-.
[1112] 韩辉, 伍波, 贾娇娇, 宋悦谦. 2022 年 5 月全球传染病疫情研判分析. 疾病监测. 2022;37(6):716-9.
[1113] 韩辉, 伍波, 贾娇娇, 宋悦谦. 2022 年 2 月全球传染病疫情概要. 疾病监测. 2022;37(3):290-2.
[1114] 韩辉, 伍波, 贾娇娇, 宋悦谦. 2023 年 5 月全球传染病疫情概要. 疾病监测. 2023;38(6):628-30.
[1115] 韩辉, 伍波, 尚士进, 贾娇娇, 宋悦谦. 2023 年 9 月全球传染病疫情概要. 疾病监测. 2023;38(10):1154-6.
[1116] 韩辉, 伍波, 邵忠阁, 贾娇娇, 宋悦谦. 2023 年 6 月全球传染病疫情概要. 疾病监测. 2023;38(7):762-4.
[1117] 韩辉, 伍波, 吴海磊, 张瑾, 贾娇娇. 2021 年 5 月全球传染病疫情概要. 疾病监测. 2021;36(6):514-6.
[1118] 韩磊, 唐青, 赵秀芹, 西条政幸, 陶晓霞. 巴楚县 2001 年新疆出血热疫情的血清学证实. 中华流行病学杂志. 2002(03):24-6.
[1119] 韩宁, 邝璐, 朱冰, 王长兵, 赵明奇. 广州地区患儿呼吸道感染病原学研究. 中华医院感染学杂志. 2016;26(05):1135-7.
[1120] 韩淑祯. 重症森林脑炎死亡病例分析. 中外医疗. 2009;28(06):69-70.
[1121] 韩淑祯, 高晓馨. 森林脑炎病人呼吸衰竭临床分析. 内蒙古医学杂志. 2009;41(03):325-7.
[1122] 韩淑祯, 张晓光. 森林脑炎肌肉瘫痪与肌电图异常关系. 中国伤残医学. 2009;17(01):18-9.
[1123] 韩淑祯, 钱乌兰. 森林脑炎伴不自主运动 12 例分析. 内蒙古民族大学学报(自然科学版). 2004(02):217-8.
[1124] 韩桃利, 孙灵利, 王英. 阿富汗主要传染病流行概况. 疾病监测. 2021;36(6):554-60.
[1125] 韩威, 吕首旭, 姚春红, 邵海华. 森林脑炎病人 BAEP 与 SEP 的研究. 中外女性健康研究. 2022(4):49-50,86.
[1126] 韩玉芳, 冯艳广, 宋予娟, 王淑娟, 吴园园, 葛廷, 等. 九项呼吸道感染病原体 IgM 检测结果分析. 中国微生态学杂志. 2013;25(07):824-5+9.
[1127] 阚甸华, 孙迎春. 小儿森林脑炎 18 例临床分析. 黑龙江医药. 1982(01):9-10.
[1128] 阚甸华, 孙迎春. 婴儿森林脑炎 2 例报告. 新医学. 1986(05):252.
[1129] 何凡, 徐建华. 以关节炎伴皮肤红斑为首发的莱姆病 1 例. 安徽医学. 2012;33(05):583-4.
[1130] 何辉, 田虹. 贵阳航空口岸首次莱姆病血清流行病学调查. 贵州医药. 1995(06):349-50.

[1131] 何剑峰 黎薇, 董新民, 罗会明, 毕德增, 常丙功. 广东省人群 Q 热感染现状. 中国人兽共患病杂志. 2001(03):97-64.
[1132] 何军, 潘光明. 50 例森林脑炎患者治疗体会. 中国医药指南. 2011;9(34):23-4.
[1133] 何益新, 赵武, 王勇. 一例人粒细胞无形体病的调查与处理. 疾病监测. 2010;25(09):752-3.
[1134] 贺金荣, 张健之, 郭衍, 潘林祥, 罗敬平. 广东省大埔县斑点热群立克次体血清学调查. 中国媒介生物学及控制杂志. 1998(06):54-6.
[1135] 赫兢, 张丽娟, 周越塑, 禹惠兰, 孟玉华, 王誓文, 等. 无形体病 11 例. 中华传染病杂志. 2012;30(4):3.
[1136] 洪俊, 黎丹. 湖北地区儿童呼吸道病原体的流行病学分析. 职业与健康. 2015;31(19):2683-6.
[1137] 侯宗柳, 黄文丽, 自登云, 张海林, 施华芳, 龚正达, 等. 云南蜱媒病毒的血清流行病学研究. 中国媒介生物学及控制杂志. 1992(03):173-6.
[1138] 胡述松, 张大荣, 王承杰, 吴传家, 万正东, 魏强, 等. 安徽省六安地区莱姆病调查. 中国人兽共患病杂志. 1999(04):113-4.
[1139] 胡伟盛. 35 例森林脑炎的临床诊疗分析. 中外医疗. 2009;28(02):36.
[1140] 华满堂, 金兆清, 林涛, 宫占威, 何成, 刘长林. 中俄、中哈边境地区莱姆病自然疫源地调查研究. 解放军预防医学杂志. 1999(06):402-5.
[1141] 华满堂, 林涛, 刘长林, 金兆清, 张哲夫, 何成, 等. 新疆阿勒泰地区人(畜)莱姆病血清流行病学调查. 中国媒介生物学及控制杂志. 1998(04):36-8.
[1142] 化冰, 李庆棣, 王赋敏, 艾承绪, 罗慰慈. 伯氏疏螺旋体感染可能是结节病的病因. 中华内科杂志. 1991;30(10):631-3.
[1143] 化冰, 张哲夫. 经节病患者血清抗伯氏疏螺旋体抗体的检测及分析. 中华内科杂志. 1997;036(2):130.
[1144] 化冰, 李庆棣, 王赋敏, 刘群英, 张哲夫, 万康林, 等. 64 例结节病人血清抗伯氏疏螺旋体抗体的检测及分析. 海军总医院学报. 1996(03):159-61.
[1145] 黄煌, 郭皖北, 刘汉胜, 刘庆武, 钟录英. 中西医结合治疗莱姆病的临床研究. 实用预防医学. 2005(02):236-7.
[1146] 黄鹏. 西藏自然疫源性疾病及医学昆虫. 旅行医学科学. 1997(04):156-7+61.
[1147] 黄育敏, 涂志华, 王洁, 吴维学. 海口地区呼吸道感染患儿非典型性病原体分析. 中国儿童保健杂志. 2013;21(04):425-8.
[1148] 黄振宇, 侯学霞, 万康林. 湖南省平江县莱姆病调查研究. 中国媒介生物学及控制杂志. 2001(04):293-4.
[1149] 惠国隆. 黑龙江省小兴安岭莱姆病调查研究. 哈尔滨医药. 1997(01):24-6.
[1150] 霍秋波，万康林，张哲夫，尚振忠，朱桂凤. 蜱传脑炎疑似患者中抗莱姆病螺旋体抗体的调查. 中国媒介生物学及控制杂志. 1996(02):127-8.
[1151] 霍云燕, 许金波, 胡立先, 韩亚平, 艾承绪. 菌必治治愈莱姆病继发红皮病一例. 中华皮肤科杂志. 1992;25(6):406.
[1152] 季云, 王玉月, 史伟峰, 沈丽. 常州地区儿童急性呼吸道感染病原体的流行病学研究. 检验医学. 2013;28(07):599-601.
[1153] 冀昌龙. 宝格达山阿拉坦合力地区蜱传森林脑炎疫源地调查. 中西医结合心血管病电子杂志. 2017;5(20):82-3.
[1154] 贾文春，万康林，张淑琴，张哲夫，宋鸿章，张晓杰，等. 从两例莱姆病患者血液中分离出莱姆病螺旋体. 中国媒介生物学及控制杂志. 1996(02):125-6.

[1155] 贾月萍, 周国萍, 张晓芳, 和娟惠, 范丽华, 谷存国, 等. 黑河地区小兴安岭林场首次分离培养出伯氏疏螺旋体. 中国预防医学杂志. 2003;4(1):54-5.
[1156] 姜双应, 易虎, 巩天祥, 杨维成, 李红, 贾月萍. 青海省同德县莱姆病血清流行病学调查分析. 青海医药杂志. 2001;31(12):51-2.
[1157] 金光, 赵黎明, 陈宏宇, 吕首旭. 森林脑炎 56 例临床分析. 感染、炎症、修复. 2010;11(03):178.
[1158] 金世文, 郝琴, 田桢, 侯学霞, 耿震, 蒋毅, 等. 1998-2003 年河南石油勘探局新疆探区人群莱姆病监测结果分析. 中国人兽共患病杂志. 2005(06):501-2+14.
[1159] 金涛, 邓晖, 于雪凡, 陆璐, 孙莉. 莱姆病神经系统损害附 2 例报告. 中风与神经疾病杂志. 2010;27(5):454-5.
[1160] 靳庆娥, 苏建荣, 乌姗娜, 辛德莉. 2015 年北京地区成人急性呼吸道感染 9 种病原体 IgM 抗体检测分析. 现代检验医学杂志. 2017;32(02):157-9.
[1161] 孔昭敏, 周新荣. 新疆人群 Q 热血清流行病学调查及病原分离. 西北国防医学杂志. 1991(01):43-4.
[1162] 赖学琴, 孔昭敏. 乌鲁木齐市 Q 热血清学调查. 地方病通报. 1986(02):168.
[1163] 李春喜, 徐健辉, 董航明, 罗网. 急性 Q 热并横纹肌溶解 1 例. 中国感染控制杂志. 2022;21(11):1135-8.
[1164] 李东明, 张硕, 陶春凤, 曾尚娟, 李颖丰, 佘尚扬. 急性呼吸道感染患儿病原体感染状况及流行病学特征. 中华妇幼临床医学杂志(电子版). 2015;11(05):625-8.
[1165] 李芳, 谭华炳, 李儒贵. 成功救治人粒细胞无形体病 1 例. 西南国防医药. 2011;21(04):356.
[1166] 李华, 孙呈祥, 阎大成. 森林脑炎与莱姆病双重感染关系探讨. 中华流行病学杂志. 1996(01):54.
[1167] 李华, 张晓光, 阎大成, 李福宽. 精神病人莱姆病螺旋体感染情况分析. 中国媒介生物学及控制杂志. 1998(04):42-3.
[1168] 李继贤. 第三例森林脑炎报告. 新疆医学院学报. 1978(02):207-9.
[1169] 李佳卿, 袁云雁, 王雨馨, 金江, 张建中. 莱姆病合并 IgA 肾病 1 例. 中国皮肤性病学杂志. 2019;33(4):453-5.
[1170] 李金贵, 肖益民, 杨贵琴, 李长春. 莱姆病 1 例报告. 中国皮肤性病学杂志. 2002;16(2):123-.
[1171] 李娟, 刘翩, 邱菊, 王开金. 1 例 Q 热肺炎诊治报告. 中国人兽共患病学报. 2022;38(02):187-9.
[1172] 李莉莉, 周玉萍, 张爱勤, 王棋, 刘增加, 胡明霞. 蜱传莱姆病一病例报告. 中华卫生杀虫药械. 2023;29(02):186-7.
[1173] 李林村，郭万申，张彦平，夏占国，郭风照，陈康礼，等. 河南省莱姆病血清流行病学调查. 河南医学研究. 1994(02):164-6.
[1174] 李宁霞. 联合检测病原体抗体和炎性标志物对儿童呼吸道感染诊断的意义 [硕士]2017.
[1175] 李芹阶, 职宁, 饶贤才, 余国泉, 俞树荣. 从云南军犬及人群发现埃立克体抗体. 中国人兽共患病杂志. 1993(02):33-4.
[1176] 李庆棣，化冰，刘群英，戴炜，王赋敏，罗慰慈. 伯氏疏螺旋体抗体测定对结节病的诊断价值. 中华结核和呼吸杂志. 1994(03):162-4+90-91.
[1177] 李荣凯, 韩玉芳, 葛廷, 牛琰, 魏巍, 王华. 新乡地区 2011 至 2014 年呼吸道感染患者病原学调查分析. 临床医学. 2020;40(01):1-5.
[1178] 李沙, 张琳, 李欢, 侯学霞, 陈婷, 苗广青, 等. 海南省东北部地区某类就诊患者莱姆病抗体检测结果分析. 实用预防医学. 2020;27(09):1068-71.

[1179] 李莎莎, 姚瑶, 郭雅琪, 杨丹. 3035 例 9 种呼吸道病原体 Ig M 抗体联合检测结果分析. 宁夏医科大学学报. 2019;41(04):391-4.
[1180] 李婷. 脑电图在森林脑炎诊断中的应用价值 [硕士]: 内蒙古民族大学; 2014.
[1181] 李伟, 贺永文. 人埃里克体病 36 例临床分析. 华中科技大学学报(医学版). 2011;40(01):109-12.
[1182] 李霞, 杨兆文, 王威严, 户中丹, 张源潮. 莱姆病 40 例病例资料分析. 慢性病学杂志. 2013;14(09):671-4.
[1183] 李祥舒, 卢天齐, 孙久林, 刘万平, 张志军, 王文远, 等. 伯氏疏螺旋体感染致周围性面瘫的临床研究. 北京中医. 1997(5):47-9.
[1184] 李晓玲. 莱姆病 2 例报告. 中国预防医学杂志. 2000(01):9.
[1185] 李孝容, 沈宏萍, 邵盛春. 临床药师参与 2 例 Q 热会诊的分析与体会. 海峡药学. 2018;30(11):240-2.
[1186] 李烨. 1 例 Q 热患者的护理查房. 当代护士(学术版). 2004(12):49-51.
[1187] 李依萍, 庞众多, 刘增加. 西北地区重要蜱媒疾病的调查研究. 中华卫生杀虫药械. 2016;22(02):180-3.
[1188] 李优良, 郝霁光, 张哲夫, 万康林, 夏先中, 张金声, 等. 四川省南川县莱姆病的调查. 中国媒介生物学及控制杂志. 1991(01):54-6.
[1189] 李兆育, 邢莉, 蔡增林. 东北地区人血清五种虫媒病毒抗体调查. 大连大学学报. 1998(04):61-5+9.
[1190] 李振, 陈秀兰, 崔凡, 汪丙松, 钱增堃, 刘福荣, 等. 芜湖市某医院 9 种常见呼吸道感染病原体的 IgM 抗体结果分析. 实用预防医学. 2023;30(06):747-9.
[1191] 李志刚, 马福海, 吴美云, 马莉, 胡熊, 林涛, 等. 宁夏六盘山地区莱姆病调查研究. 中国媒介生物学及控制杂志. 1997(06):441-3.
[1192] 李志清, 宫占威, 费晋秀, 石胜刚, 刘涛, 冯晓妍, 等. 陕西省部分林区莱姆病血清流行病学调查研究. 中国人兽共患病学报. 2010;26(9):879-80.
[1193] 李志清, 刘增加, 费晋秀, 许诺, 郭宏林, 张峰, 等. 陕西省莱姆病自然疫源地调查研究. 中华卫生杀虫药械. 2010;16(05):356-9.
[1194] 李忠, 丁淑军, 吕慧, 陈勇, 侯配强, 王显军. 山东省 1 例人粒细胞无形体病调查. 传染病信息. 2009;22(03):165-8.
[1195] 李忠, 孙桐, 黄捷通, 冯开军. 鲁南地区首次 Q 热和斑点热抗体检测报告. 中国公共卫生学报. 1997(01):15-6.
[1196] 梁晨, 魏伟. 2008 至 2017 年某市职业性森林脑炎流行病学与职业特征和临床表现. 中华劳动卫生职业病杂志. 2018;36(8):3.
[1197] 梁华忠, 赖初麟, 方锦嵩. 莱姆病两例报告. 广西医学. 1992(04):275-7.
[1198] 梁劲松, 夏贞莲. 2754 例南宁市呼吸道感染患者九种血清病毒流行病学的调查研究. 广西中医药大学学报. 2014;17(02):42-4.
[1199] 梁瑶, 吴祥林, 王晓川, 郭志强, 蓝健, 温杰翔. 宏基因组二代测序辅助诊断 Q 热立克次体肺炎 1 例. 中国感染与化疗杂志. 2022;22(01):91-4.
[1200] 梁长威, 张颖, 赵景波, 张之伦, 禹惠兰, 阴杰莹, 等. 天津高危人群蜱源立克次体病流行病学监测. 中国公共卫生. 2011;27(06):719-20.
[1201] 廖芳宇, 黄小娟, 刘斌, 张华. 先天性心脏病合并社区获得性肺炎患儿呼吸道病毒及特殊病原体相关分析. 西部医学. 2021;33(05):701-4+8.
[1202] 林碧瑚. 应用 DEB—ELISA 试验检测种特异性立克次体抗体. 海南医学院学报.

1996(02):49-51.
[1203] 林成虎. 长白山区（二道白河）莱姆病的流行病学调查 [硕士]2005.
[1204] 林成虎. 长白山林区莱姆病的血清学调查. 延边大学医学学报. 2008;31(04):272-4.
[1205] 林光宇, 张守印, 沈江建, 谢克锦, 郑嘉华, 彭兰英. 2 例临床诊断粒细胞无形体病回顾性调查. 海峡预防医学杂志. 2009;15(05):31-2.
[1206] 林光宇, 张守印, 谢克锦, 沈江建, 郑嘉华, 严延生, 等. 福建省武夷山林区人群人粒细胞无形体血清流行病学调查. 中华流行病学杂志. 2008;29(11):2.
[1207] 林鸿. 莱姆病四例报告. 中国疗养医学. 2010;19(02):178-9.
[1208] 林孔翊, 李忠, 孙桐, 黄捷通, 冯开军. 山东省陵县 Q 热和斑点热的血清流行病学调查. 预防医学文献信息. 1996(2):122-3.
[1209] 林正方. 广州地区儿童血清 EV71-IgG 阳性率与中和抗体水平的比较及急性呼吸道感染的病原学分析 [硕士]: 广州医科大学; 2015.
[1210] 凌锋, 陆群英, 翁卫东, 姜理平, 赵江灵, 金利胜. 浙江首例人粒细胞无形体病患者病原学分析. 国际流行病学传染病学杂志. 2011;38(6):4.
[1211] 刘朝阳, 蒋嫣, 龙雅琴, 赵明伟, 周伟东, 胡晓雯, 等. 二代测序诊断慢性 Q 热并发腰肌脓肿 1 例. 中国矫形外科杂志. 2023;31(5):479-80.
[1212] 刘芳, 黄志刚. mNGS 辅助诊断急性 Q 热 1 例. 岭南急诊医学杂志. 2023;28(01):89-90.
[1213] 刘洪波. 蜱感染病原体的分离鉴定与监测研究 [博士]2018.
[1214] 刘洪涛. 森林脑炎致精神障碍 1 例. 临床心身疾病杂志. 2008;14(3):252-.
[1215] 刘惠民. 森林脑炎并不安腿综合症一例报告. 石河子医学院学报. 1993(03):198.
[1216] 刘洁, 何美琳, 邵冬华, 曹清芸. 3151 例九种呼吸道病原体 IgM 检测结果分析. 海南医学. 2015(4):537-9.
[1217] 刘俊晓, 王孟丽, 张艳. 住院患儿 9 种呼吸道病原体检测结果分析. 河南科技大学学报(医学版). 2018;36(03):222-4.
[1218] 刘敏, 黄义恒. 森林脑炎头痛病人的护理体会. 世界最新医学信息文摘. 2015;15(08):204-5.
[1219] 刘庆武, 李俊华, 张立新, 万康林, 杨烈, 陶学永, 等. 湖南省郴州地区莱姆病调查. 中国媒介生物学及控制杂志. 1997(02):125-6.
[1220] 刘晓清, 盛瑞媛, 王爱霞, 秦树林. 莱姆病的诊断和治疗(附 6 例报告). 中国医学科学院学报. 2000(04):398-9.
[1221] 刘义, 耿震, 陈继永, 侯学霞, 宋春英, 郝琴, 等. 天津市蓟县多发性神经炎患者血莱姆病螺旋体分离调查. 天津医药. 2008(05):345.
[1222] 刘义, 陈继永, 宋春英, 于凤太, 贾艳合, 丁健青. 首次报导天津市蓟县莱姆病的调查研究. 医学动物防制. 2001(09):485-7.
[1223] 刘远恒, 柴君杰, 肖成恩, 李文惠. 新疆出血热 140 例流行病学分析. 地方病通报. 2004(S1):47-9.
[1224] 刘增加, 华满堂, 石淑珍, 杨银书, 罗远琼, 宫占威. 西北部分地区莱姆病与人、家养动物、啮齿动物关系的研究. 医学动物防制. 2000(06):298-301.
[1225] 刘增加, 康新民, 粟多寿, 石淑珍, 杨俭. 祁连山北麓铧尖地区莱姆病自然疫源地调查研究. 中国兽医科技. 1993(11):13-4+48.
[1226] 龙健, 林涛, 李文斌, 张哲夫. 江西上高县人和动物莱姆病流行病学调查. 中国媒介生物学及控制杂志. 1999(01):51-3.
[1227] 龙江, 牛晓珊, 文军, 冒文娟, 仲婷, 李红燕, 等. 乌鲁木齐地区献血者伯氏疏螺旋体感染率调查. 中国媒介生物学及控制杂志. 2017;28(03):280-2.

[1228] 卢宏霞, 刘增柱. 森林脑炎 30 例临床资料分析及报告. 中国保健营养. 2017;27(11).
[1229] 陆敬民. 以神经系统表现为主的莱姆病 17 例临床分析. 医学临床研究. 2003(11):831-3.
[1230] 吕红. 人粒细胞无形体病的临床观察及护理体会. 滨州医学院学报. 2013;36(06):468-9.
[1231] 吕天增, 冯方波, 张建武, 谢明, 周国萍, 唐兰. 驻莱姆病高发地区某部莱姆病流行病学调查. 解放军预防医学杂志. 1997(04):42-3.
[1232] 吕小龙. 内蒙古大兴安岭林区森林脑炎住院病人的回顾性研究 [硕士]: 内蒙古民族大学; 2014.
[1233] 吕小龙, 张晓光, 韩淑祯. 森林脑炎所致呼吸肌麻痹 1 例. 大家健康(学术版). 2014;8(10):223-4.
[1234] 吕燕宁, 窦相峰, 陈丽娟, 孙玉兰, 张秀春, 关增智, 等. 北京东北部山区人群嗜吞噬细胞无形体血清流行病学调查. 中国人兽共患病学报. 2016;32(10):861-4+70.
[1235] 吕燕宁, 窦相峰, 甘亚弟, 张勇, 张秀春, 关增智, 等. 北京市首次网络直报莱姆病 1 例报告. 首都公共卫生. 2010;4(06):277-9.
[1236] 罗娇, 和倩, 黄丽华, 顾伟, 蒲艳琳, 陈会桥, 等. mNGS 诊断急性 Q 热二例并文献复习. 临床医学进展. 2023;13(7):12005-11.
[1237] 罗锦鸿. 南中国海 Q 热流行病学调查研究 [硕士]2023.
[1238] 罗云杰, 赵丽萍, 许贺春. 2608 例呼吸道感染九项病原体 IgM 检测结果分析. 中国城乡企业卫生. 2014;29(04):97-9.
[1239] 罗宗初, 唐群兰, 陈丰, 张电, 鲁霞. 某区小儿急性呼吸道感染九种病原体 IgM 抗体检测的意义. 中国医药指南. 2013;11(04):227-9.
[1240] 骆福余, 张建华, 岳武. 森林脑炎 40 例临床分析. 哈尔滨医药. 1989(02):14-6.
[1241] 马海滨, 杨文映, 杨向东, 张哲夫, 万康林, 张知德. 云南丽江、孟连莱姆病血清流行病学调查报告. 云南医药. 1991(06):384-5.
[1242] 马慧, 沈永明, 司萍, 彭林. 急性呼吸道感染儿童 9 种呼吸道病原体 IgM 抗体检测分析及其与空气污染的相关性探讨. 中国免疫学杂志. 2018;34(04):576-82.
[1243] 马兰. 人感染新斑点热群立克次体的发现及自然疫源地调查研究 [硕士]2015.
[1244] 马岚云, 王锋, 王翠兰. 列车上一起确诊人粒细胞无形体病例的调查分析. 疾病监测与控制. 2012;6(08):479-81.
[1245] 马仕金, 卢登明, 张有植. 西藏林芝地区森林脑炎血清抗体调查. 中华预防医学杂志. 1996(06):28.
[1246] 马新秋, 王晶. 莱姆病导致肝损伤 25 例报告. 临床肝胆病杂志. 2007;23(4):298-.
[1247] 马颖, 张峰波, 季萍, 夏宇, 贾斌, 胡金伟, 等. 新疆非典型呼吸道感染病原体检测结果分析. 新疆医科大学学报. 2016;39(01):87-9.
[1248] 马哲, 许银花, 吴光. 延边地区森林脑炎流行病学调查研究. 中国保健营养. 2016;26(33).
[1249] 毛贝, 籍希平, 张玉珲, 李如森. 新疆出血热的病理学观察及与临床联系的探讨. 地方病通报. 2004(S1):37-43+121-4.
[1250] 毛丽君, 赵金垣, 徐希娴, 孙淑云. 380 例森林脑炎临床分析. 中国工业医学杂志. 2002(03):137-40.
[1251] 毛晓霞, 杜荣. 乌鲁木齐市某中医院 2018-2020 年门诊部就诊 1000 例呼吸道感染患者病原体检测结果分析. 新疆医学. 2023;53(06):712-4+49.
[1252] 孟庆学, 夏侯玉发, 王军峰. 人粒细胞无形体病的临床及影像学表现. 中国中西医结合影像学杂志. 2012;10(01):51-2+73.
[1253] 孟昭鹏. Q 热心内膜炎. 传染病信息. 1995(01):40.

[1254] 莫伟平, 张泳仪, 莫海兴, 郭振添, 罗进通. 手足口病患儿呼吸道病原体 IgM 抗体检测结果分析. 深圳中西医结合杂志. 2017;27(06):61-2.
[1255] 牟路萌. 莱姆病、布鲁菌病诊断方法的建立和流行病学调查 [硕士]2016.
[1256] 牟艳，王瑞春. 蜱叮咬致森林脑炎一例临床报告. 工业卫生与职业病. 1995(05):300.
[1257] 娜仁, 张嘉懿, 司萍, 沈永明. 天津市急性呼吸道感染住院患儿非细菌性病原体感染及与空气质量关系的初步研究. 环境与健康杂志. 2019;36(10):898-901.
[1258] 娜仁, 张嘉懿, 司萍, 崔小健, 郭文伟, 沈永明. 18252 例儿童急性呼吸道感染常见非细菌病原体的流行特征分析. 中国妇幼保健. 2019;34(15):3490-2.
[1259] 娜仁, 赵全良, 佟艳秋. 莱姆病致外展神经麻痹四例. 中华眼科杂志. 2003;39(9):573.
[1260] 倪慧萍, 季伟, 王永清, 史伟峰, 陈海霞. 2011—2013 年常州地区急性呼吸道感染住院儿童病原学研究. 实用临床医药杂志. 2014;18(17):199-202.
[1261] 倪林仙, 吴倩, 樊茂, 宋顺祺, 陈祝. 昆明地区小儿非典型肺炎病原体临床实验研究. 医学研究通讯. 2004(10):14-6.
[1262] 倪少娟, 陶春凤, 周华辉, 黄丽英, 李明艺. 儿科重症监护室和普通病房小儿下呼吸道感染病原学分析. 中华妇幼临床医学杂志(电子版). 2012;8(06):740-3.
[1263] 倪雪冰. 基于哨点医院的莱姆病临床及病原特征分析 [硕士]2013.
[1264] 倪寅凯, 路喆鑫, 赵金龙, 付亮, 陈宗辉, 励峰. Q 热立克次体感染性心内膜炎一例. 中华传染病杂志. 2020;38(3):2.
[1265] 倪中华. 森林脑炎呼吸肌麻痹患者早期心理干预. 黑龙江医学. 2012;36(02):120-2.
[1266] 倪中华, 郝淑梦. 森林脑炎致呼吸肌麻痹的相关因素分析. 医学动物防制. 2014;30(06):609-11.
[1267] 倪中华, 郝淑梦. 蒙西医结合治疗森林脑炎 32 例临床观察. 中国民族民间医药. 2015;24(03):3-4.
[1268] 聂童. 白介素与森林脑炎患者病情及预后的相关性研究 [硕士]: 内蒙古民族大学; 2011.
[1269] 钮莉春，张姝丽，武贵森，刘兰. 内蒙古健康人群 Q 热抗体水平调查报告. 内蒙古医学杂志. 1996(01):38-9.
[1270] 欧阳建, 张启国, 关朝阳, 徐勇, 许景艳, 陈兵, 等. 人粒细胞无形体病——一例报告并文献复习. 中华血液学杂志. 2009(8):2.
[1271] 潘光明. 森林脑炎患者 70 例临床治疗体会. 吉林医学. 2011;32(34):7342-3.
[1272] 潘家云. 海南岛 Q 热 2 例报告. 广东医学. 1984(02):42.
[1273] 潘家云. Q 热性肝硬化 1 例报告. 实用医学杂志. 1987(01):23.
[1274] 潘亮, 潘敏楠, 严延生, 徐国英, 祝庆余, 杨保安, 等. 福建首次从临床病例中发现东部马脑炎病毒感染者. 海峡预防医学杂志. 2003(04):31-2.
[1275] 潘亮, 于恩庶, 林金瑞, 张哲夫, 曹伯良, 王志坚, 等. 福建省发现莱姆病. 中国人兽共患病杂志. 1990(05):63.
[1276] 潘亮，陈振光，黄耀平，罗肱良，丁景昭. 福建省莱姆病的临床特征分析. 海峡预防医学杂志. 1996(03):3-5.
[1277] 潘小玲, 王刚, 刘建荣. 神经莱姆病 1 例报告与分析. 内科理论与实践. 2009;4(04):316-7.
[1278] 庞卫龙, 崔清荣, 葛君华. 天台县主要蜱媒传染病血清流行病学调查. 浙江预防医学. 2012;24(07):27-9.
[1279] 裴永菊, 谢舒棠, 王曦, 倪明辉, 李江. ICU 内老年重症肺炎患者呼吸道感染的血清流行病学调查研究. 中国实验诊断学. 2021;25(05):661-4.
[1280] 彭秋，张小澍，袁玉民，夏秀华. 以发作性头痛为主要表现的莱姆病两例报道. 中国疼痛

医学杂志. 1996(04):242-4.
[1281] 蒲增惠, 赵茂茂, 于红霞, 林鹏, 王淑华, 毛崎善. 人粒细胞无形体病三例. 中华传染病杂志. 2008;26(10):632.
[1282] 齐侠. 儿童莱姆病 30 例临床特点分析. 中华儿科杂志. 2003(01):33-4.
[1283] 齐月, 王峰. 莱姆病的临床分析(附 11 例报告). 吉林大学学报(医学版). 2005(01):20.
[1284] 钱红姣, 郑锦利, 陈伦, 曾义协. 2017-2018 年闽东地区儿童呼吸道病原体感染流行特征分析. 检验医学与临床. 2020;17(18):2699-701.
[1285] 钱鹏, 李宗州, 李玲. 呼吸道感染病原体 IgM 抗体检测结果分析. 中国保健营养. 2015;25(15):35-6.
[1286] 钱振荣. 中、重型森林脑炎病人的观察与护理. 中华护理杂志. 1990(08):378-9.
[1287] 乔飞, 盛云峰, 高蕾, 汪茂荣, 何长伦, 隋云华, 等. 慢性 Q 热一例. 中华传染病杂志. 2006(05):358.
[1288] 秦笙, 练立婷, 王维亮, 陈富, 李际强, 吴泽, 等. 广州地区下呼吸道感染老年患者非典型病原体与病毒检测及临床特征分析. 中国医药导报. 2017;14(13):120-3.
[1289] 秦笙, 练立婷, 王维亮, 张伟铮, 陈茶, 郑贵星. 广州发热呼吸道症候群成年患者临床特征及病原学检测. 中国热带医学. 2016;16(05):495-8.
[1290] 秦文浩, 李玉芝, 宋彩华. 牙克石地区人群埃里希病流行病学调查. 内蒙古医学杂志. 2006(01):65-6.
[1291] 渠战芬, 牛俊英, 冯方波, 夏秀华. 贝尔麻痹与莱姆病 83 例报告. 中华神经科杂志. 1996(06):61.
[1292] 冉健, 裴元元, 汤菊妹, 魏凤香. 深圳地区 9 种常见呼吸道病原体 IgM 抗体结果. 热带医学杂志. 2016;16(09):1112-4.
[1293] 仁青东珠. 蒙药丹巴-1 联合病毒唑治疗森林脑炎退热作用研究 [硕士]: 内蒙古民族大学; 2017.
[1294] 任超. 儿童下呼吸道感染病原体及感染指标的研究 [硕士]2020.
[1295] 任次早，王以银，刘红，罗兆庄. 安徽省首次发现人群感染克里米亚－刚果出血热. 中华流行病学杂志. 1995(01):13.
[1296] 任建红, 陈霞, 柯辉. 1 例小儿人粒细胞无形体病的护理体会. 湖北医药学院学报. 2013;32(03):275-6.
[1297] 任莲花. 森林脑炎患者的护理 41 例. 中国社区医师(医学专业). 2012;14(20):316-7.
[1298] 任自实, 张维广, 李晓剑. 中医药治疗莱姆病 1 例报告. 中医杂志. 1994(05):288.
[1299] 荣刘涛, 李秀玲, 祝金明, 陈相磊. 2016 年至 2020 年 1～6 岁儿童呼吸道感染病例流行病学特征. 中国医学工程. 2022;30(05):64-8.
[1300] 沙银中, 刘雯, 许爱敏, 李亚东, 彭红梅. 喀什地区儿童呼吸道感染病原体的 IgM 抗体检测结果分析. 临床研究. 2015;23(12):57-8.
[1301] 邵庆花. 森林脑炎患者气管切开后的护理 60 例. 实用护理杂志. 2003(04):53-4.
[1302] 邵庆花, 张英杰, 楮艳丽. 莱姆病性心肌炎病人的护理. 中国伤残医学. 2009;17(02):109-10.
[1303] 盛玉. 人粒细胞无形体病患者的护理. 实用医药杂志. 2012;29(12):1100-1.
[1304] 石冬梅, 宋莉, 孙嫚丽, 李丹丹, 王吉, 邹海梁, 等. 职业性森林脑炎患者神经功能损伤的客观检查. 中国工业医学杂志. 2022;35(04):326-7.
[1305] 石华, 王玥, 韩华, 高晓丽, 张雅明, 张文佳, 等. 牡丹江林业中心医院 92 例蜱媒传染病病例分析. 中国媒介生物学及控制杂志. 2013;24(04):295-6+300.
[1306] 史瑀, 黄珊珊, 王琪, 郑智, 殷翠香. 森林脑炎患者呼吸机相关肺炎危险因素分析及病原

菌分布. 工业卫生与职业病. 2024;50(02):163-5+82.
[1307] 舒静, 陈宇宁, 刘小花, 姜源, 邓剑. 泸州地区儿童急性呼吸道感染病原体检测结果分析. 广东医学. 2016;37(20):3033-6.
[1308] 双丽. 呼伦贝尔鄂温克草原地区蜱传脑炎调查研究 [硕士]: 内蒙古民族大学; 2015.
[1309] 双丽, 张晓光, 韩淑祯. 呼伦贝尔草原蜱传脑炎的特殊症状报告. 大家健康(学术版). 2014;8(24):322.
[1310] 宋文仕, 张海艳, 马海生, 柯君兰. 九项呼吸道病原体检测在儿童呼吸道感染中应用分析. 齐齐哈尔医学院学报. 2015;36(26):3988-9.
[1311] 宋文英, 陈凤玲, 蒋惠荷, 李英欣, 陈艺林. 莱姆病的临床治疗探讨. 中国人兽共患病杂志. 1997(01):38-40.
[1312] 宋兆华, 崔清兰. 森林脑炎病人高热的护理体会. 黑龙江医药科学. 2002(03):117.
[1313] 孙彬, 吴纯, 韩冰, 李妮, 何菊芳, 董梅. 九种呼吸道感染病原体的IgM抗体检测结果分析. 标记免疫分析与临床. 2015;22(07):604-7.
[1314] 孙桂兰, 张昱, 李桂龄, 何振林. 莱姆(Lyme)病的神经系统表现(附 2 例报告). 中风与神经疾病杂志. 1992(02):92-3.
[1315] 孙恒松, 田桢, 耿震, 侯学霞, 郝琴, 蒋毅, 等. 河南油田新疆石油探区莱姆病流行病学调查. 中国媒介生物学及控制杂志. 2005(03):209-11.
[1316] 孙红妮, 任虹旭, 贺永超, 潘辰, 柳笑榆, 林咏梅, 等. 贝氏柯克斯体感染致心内膜炎 1 例. 传染病信息. 2023;36(1):90-3.
[1317] 孙丽丽, 许庆梅. 2 例重症森脑持续植物状态合并丝状角膜炎病人的护理. 世界最新医学信息文摘（连续型电子期刊）. 2015(8):205-6.
[1318] 孙铁流. 慢性 Q 热. 国外医学流行病学传染病学分册. 1994(03):145.
[1319] 孙桐. 山东地区立克次体病调查. 中国公共卫生. 1999(10):24-5.
[1320] 孙伟. 森林脑炎致双相情感障碍患者的血清细胞因子水平研究. 世界最新医学信息文摘（连续型电子期刊）. 2021;21(1):210-1,3.
[1321] 孙雪芹. 儿童呼吸道感染应用呼吸道九项病原体检测的效果. 饮食保健. 2023(2):29-32.
[1322] 孙亚男. 森林脑炎患者脑脊液抗体水平的监测研究 [硕士]: 内蒙古民族大学; 2012.
[1323] 孙亚男, 韩淑祯, 从日照, 张晓光. 重症森林脑炎 1 例报告. 中外医学研究. 2012;10(11):152-3.
[1324] 孙印旗, 王勇, 姜霞, 姚娜, 钱振宇, 刘晓丽, 等. 河北新发斑点热及人粒细胞无形体病实验室调查分析. 中国媒介生物学及控制杂志. 2015(4):344-8.
[1325] 孙玉今, 金恩浩, 赵志梅, 全松石, 刘慧楠. 森林脑炎的 MRI 表现. 实用放射学杂志. 2011;27(10):3.
[1326] 孙玉兰, 窦相峰, 吕燕宁, 王小梅, 关增智, 王全意. 北京地区畜牧业相关人群Q热血清学调查. 职业与健康. 2013;29(22):2967-8+71.
[1327] 孙长俭, 周连庆, 薛文成, 于笑难. 辽宁省农村地区 Q 热立克次体血清学及虫媒蜱携带病原体状况调查. 华南国防医学杂志. 2016;30(06):362-4.
[1328] 谭明凯, 石亚玲. 小儿呼吸道感染患者中九项病原体 IgM 抗体检测结果及机制分析. 医药前沿. 2020;10(2):2.
[1329] 谭文文, 常亮, 董美华, 周建刚, 张华君, 朱莹莹, 等. 宜兴市一般人群立克次体病血清流行病学调查. 中华疾病控制杂志. 2015;19(01):96-8.
[1330] 谭毓绘, 刘勇, 孙荷, 龙江, 牛晓珊, 于鲁海, 等. 聚合酶链法检测莱姆病患者尿液中伯氏疏螺旋体 DNA(附 17 例报道). 中国临床神经科学. 2012;20(04):372-6.

[1331] 谭毓绘, 刘勇, 孙荷, 于鲁海, 龙江, 牛晓珊, 等. 2000 至 2004 年新疆维吾尔自治区莱姆病的监测. 中国临床神经科学. 2007(02):158-61.
[1332] 谭毓绘, 刘勇, 孙荷, 于鲁海, 曼古努尔, 朱丽, 等. 新疆乌鲁木齐南山莱姆病自然疫源地监测分析. 中国媒介生物学及控制杂志. 2011;22(02):141-3.
[1333] 谭毓绘, 刘勇, 万康林, 郝琴, 孙荷, 于鲁海, 等. 新疆人群感染莱姆病螺旋体分子流行病学调查. 中国媒介生物学及控制杂志. 2013;24(04):297-300.
[1334] 谭毓绘, 刘涌, 孙荷, 贺宇新, 孙岩, 谢荣, 等. 新疆地区神经系统莱姆病的临床特征分析. 中国媒介生物学及控制杂志. 2002(02):93-4.
[1335] 谭兆营, 李亮, 张丽娟. 江苏省几种主要类型人畜立克次体抗体阳性检出情况的现况调查. 苏州大学学报(医学版). 2012;32(04):445-9+593.
[1336] 汤伯明, 游传新, 夏占国. 豫西地区莱姆病血清学及病原学研究. 洛阳医专学报. 2000(01):35-6.
[1337] 唐娇. 呼吸道病原体九联检在小儿急性呼吸道疾病中的应用分析. 世界最新医学信息文摘（连续型电子期刊）. 2020;20(92):64-5.
[1338] 唐青, 韩磊, 赵秀芹, 陶晓霞. 新疆巴楚地区 2001 年克里米亚-刚果出血热现场调查报告. 疾病监测. 2002(02):50.
[1339] 唐学良. 郑州地区 1304 例呼吸道感染病原体检测结果分析. 中国实用医药. 2015;10(02):96-7.
[1340] 陶增珧, 杨成君, 刘希真, 刘国平, 董业志, 王丕基, 等. 吉林省抚松、珲春地区莱姆病调查. 中国公共卫生学报. 1991(01):15-7.
[1341] 田茂芳, 韩亚, 龙文斌, 周瑞其, 王昭孝, 卢大奇. 铁路筑路人群莱姆病血清学调查. 铁道医学. 1999(05):298-9.
[1342] 田思佳, 张婷, 孔乐乐, 周琳, 梁艳. Q 热立克次体感染伴急性肾损伤 1 例. 临床检验杂志. 2022;40(08):637-8.
[1343] 田桢, 陈建, 万康林. 175 例莱姆病患者治疗效果研究. 中国预防医学杂志. 2004(01):58-60.
[1344] 佟艳秋, 刘玲, 赵全良, 孙刚. 林区森林脑炎病毒引起眼部神经损害的临床探讨. 内蒙古医学杂志. 2006(01):87-8.
[1345] 佟艳秋, 孙刚, 马慧蕾, 张晓光. 森林脑炎病毒感染致视神经炎的远期疗效观察. 国际眼科杂志. 2009;9(12):2432-3.
[1346] 佟艳秋, 赵全良, 刘玲. 森林脑炎致眼部病变 44 例分析. 中国实用眼科杂志. 2006(10):1068-9.
[1347] 万清平, 徐能鹏, 陈桃珍, 高祖送, 袁秀兰. 6 例人粒细胞无形体病实验室检测结果分析. 咸宁学院学报(医学版). 2010;24(01):68.
[1348] 王波, 马春萍, 朱文芳, 王凤林. 甘肃部分地区莱姆病血清流行病学及相关因素调查研究. 西南国防医药. 2020;30(10):971-2.
[1349] 王超. 八项呼吸道病原体检测在小儿呼吸道感染中的临床意义. 医疗装备. 2017;30(14):59-60.
[1350] 王超, 许琳, 鄂冰, 麻婷. 1 例重型森林脑炎长期留置尿管患者的并发症及护理. 世界最新医学信息文摘（连续型电子期刊）. 2015(19):121-,7.
[1351] 王春花. 森林脑炎高热病人的护理. 中国疗养医学. 2010;19(05):444.
[1352] 王春花. 森林脑炎合并糖尿病昏迷患者的护理体会. 黑龙江医学. 2015;39(06):706.
[1353] 王春生, 万康林, 杨修军, 王博, 张国芳, 梁小平, 等. 吉林省平原地区莱姆病流行病学调查. 实用预防医学. 2009;16(02):341-2.

[1354] 王春生, 杜占森, 杨修军, 杨红, 万康林, 杨学敏, 等. 长春地区首次发现莱姆病疫源地. 中国卫生工程学. 2005(03):158-60.
[1355] 王春生, 王峰, 王珍, 王艳华, 李凤银, 崔铁娟,等. 血清学检测诊断莱姆病结果分析. 中国卫生工程学. 2003(01):53.
[1356] 王春晓, 高洪丽, 马克光. 森林脑炎 2 例报告及治疗体会. 吉林大学学报(医学版). 2006(06):1133.
[1357] 王芳, 林见敏, 陈辉凤, 王金金, 张建林, 邓燕燕, 等. 上海青浦 2015 年至 2016 年 2241 例呼吸道感染患者 9 项常见病原体检出情况分析. 诊断学理论与实践. 2018;17(02):207-10.
[1358] 王菲, 梅淑慧. 1 例重型森林脑炎的护理. 中国实用护理杂志. 2005(08):46.
[1359] 王峰. 云南省嗜吞噬细胞无形体分布情况调查及无形体表面蛋白 MSP2 原核表达 [博士]2019.
[1360] 王光璐, 张风, 孟淑敏, 艾承绪, 许金波, 霍云燕. 莱姆病的眼底表现及其治疗. 眼科. 1993(02):82-4+126.
[1361] 王桂杰, 张晓光, 孙欣. 森林脑炎并发心肌损害研究. 内蒙古医学杂志. 2012;44(02):176-9.
[1362] 王国栋, 张腾, 李杰, 孔俭. 输血导致莱姆病形成III度房室传导阻滞一例. 中华传染病杂志. 2017;35(4):243.
[1363] 王国华. 人粒细胞无形体病的护理体会. 实用医药杂志. 2011;28(08):732-3.
[1364] 王海涛, 杨桂生, 宋修爱. 人粒细胞无形体病误诊为流行性出血热. 临床误诊误治. 2011;24(03):67-8.
[1365] 王红, 郝爱庆. 气管切开术在抢救森林脑炎中的应用体会. 牡丹江医学院学报. 2002(03):34-5.
[1366] 王化勇, 侯学霞, 李立琴, 耿震, 王全意, 郝琴. 2005 年北京市密云地区人群莱姆病血清流行病学调查研究. 中国预防医学杂志. 2009;10(08):737-9.
[1367] 王津洲, 宁立友. 青阳县莱姆病血清流行病学调查. 中国农村医学. 1992(07):23.
[1368] 王晶, 宝音扎布, 韩淑祯. 森林脑炎临床与脑电图变化分析. 黑龙江医学. 2007(04):276-7.
[1369] 王晶, 王蒙. 急性期森林脑炎患者头颅 CT 影像学观察. 中国地方病学杂志. 2009(4):1.
[1370] 王磊, 杜琨, 栗绍刚, 吴赵永, 齐志群, 邹洋, 等. 仅以发热为首发症状的莱姆病—附 1 例报告. 中国热带医学. 2013;13(01):125-6.
[1371] 王丽娟, 侯学霞, 陈志林, 郝琴, 陶小润, 万康林. 山东省莱姆病地理流行病学研究. 中国媒介生物学及控制杂志. 2007(04):306-8.
[1372] 王丽娟, 万康林, 刘士礼, 陈志林, 冯开军, 侯学霞, 等. 首次调查发现山东省存在莱姆病自然疫源地. 中华流行病学杂志. 2000(04):52-4.
[1373] 王莉莎, 祝开思, 曲建昌, 孙楠楠. 莱姆病关节炎误诊为脊柱关节病 1 例. 中国临床医生. 2010;38(01):77-9.
[1374] 王路. 呼吸道感染九种病原体检出情况分析 [硕士]2015.
[1375] 王路, 刘旻, 程江. 新疆石河子地区上半年呼吸道感染病原体的 IgM 抗体检测结果分析. 国际检验医学杂志. 2015;36(07):895-6.
[1376] 王沛文, 于少飞, 柴少卿. 儿童 Q 热 3 例报道. 新发传染病电子杂志. 2023;8(05):52-5.
[1377] 王鹏, 金哲虎, 韩妤侠, 郑振龙. 莱姆病误诊为蜱螫伤 1 例报告. 吉林医学. 2001(01):30.
[1378] 王倩莹. 黑龙江省边境地区蜱虫传播病原体调查 [硕士]2019.
[1379] 王树桂，曲桂华，王晓川，魏彬，林鹏翥，朱建华，任国松，刘明先. 森林脑炎和莱姆病双重感染 26 例临床分析. 中国媒介生物学及控制杂志. 1995(04):291-3.
[1380] 王双青，占炳东，曹国平，余樟有，张建民. 柯城区老年人群及室内鼠类伯氏疏螺旋体感

染状况. 预防医学. 2018;30(12):1252-4.
[1381] 王爽, 寇增强, 王梅, 任艳艳, 胡彬, 房明, 等. 一起人粒细胞无形体和查菲埃立克体符合感染病例的确认和调查. 疾病监测. 2012;27(08):642-3.
[1382] 王斯丽. 6926 例儿童下呼吸道感染病原体检测与分析. 母婴世界. 2020(9):16-7.
[1383] 王晓峰, 张树林. Q 热并发心内膜炎一例. 中华内科杂志. 2002;41(7):490.
[1384] 王雪云, 张琪然, 秦艳丽, 金嘉琳. 主动脉夹层支架植入术后慢性 Q 热 1 例. 中华传染病杂志. 2024;42(2):114-5.
[1385] 王彦学, 武建英. 复发性 Q 热误诊为上呼吸道感染一例. 临床误诊误治. 1992(02):87.
[1386] 王艳丽. 18 例森林脑炎昏迷患者的护理. 中国医药指南. 2011;9(35):225-6.
[1387] 王雁. 14 例重症森林脑炎死亡病例分析. 医学动物防制. 2006(02):150-1.
[1388] 王长华, 魏镜, 高淑芳. 莱姆病的神经系统表现. 北京医学. 1997(01):7-9.
[1389] 王志超, 张伟, 王月磊, 殷楚强, 沈峰, 曹振鲁, 等. 宏基因组二代测序辅助诊断 Q 热脊柱感染 1 例报道. 实用骨科杂志. 2024;30(03):272-4.
[1390] 王子敏, 万康林, 邱勤, 米庆秀, 梅传安, 吴凤英, 等. 湘西南山区莱姆病的发现. 中国人兽共患病杂志. 1993(06):42-3.
[1391] 魏然. 绵羊无形体自然疫源地调查及其对人致病性研究 [硕士]2017.
[1392] 魏伟, 梁晨. 2006—2020 年呼伦贝尔市新发职业病病例分析. 中国工业医学杂志. 2021;34(06):532-4.
[1393] 魏英, 武伦, 王靳琎, 黄慧敏, 陈琴华. 2018 年湖北省十堰地区某医院呼吸道 9 种病原体检测结果分析. 湖北医药学院学报. 2020;39(04):344-8.
[1394] 温玉欣, 艾承绪, 张永国, 李德荣, 徐在海, 邱贵城, 等. 从莱姆病患者血液分离出螺旋体. 微生物学报. 1988;28(03):275-8.
[1395] 文海军, 谢珊, 岳梦婷, 陈培培, 周绮娴. 呼吸道病原体 IgM 抗体联合检测运用于呼吸道感染诊断的临床分析. 国际医药卫生导报. 2015;21(24):3647-9.
[1396] 文金谦. 呼吸道病原体 IgM 抗体联合检测在 1236 例呼吸道感染中的应用. 河南医学高等专科学校学报. 2017;29(1):70-1.
[1397] 吴伯宪，林萍，吴钦玲，童绎，潘亮. 福建省林区莱姆病的调查与防治措施探讨. 实用预防医学. 1994(03):137-8.
[1398] 吴福清, 曹明华, 刘伯林, 唐永飞. 1 例人粒细胞无形体病流行病学调查. 安徽预防医学杂志. 2010;16(05):393+5.
[1399] 吴卉. 20 例森林脑炎患者的临床分析. 中国实用医药. 2012;7(2):50-1.
[1400] 吴嘉文, 赵倩倩, 马继军, 刘晓雪, 李崇巍. 儿童急性 Q 热一例. 中华传染病杂志. 2021;39(9):569-70.
[1401] 吴茜, 陈祝, 倪林仙, 吴澄清. 社区获得性小儿肺炎病原体组成现状及临床探讨. 小儿急救医学. 2005;12(6):479-82.
[1402] 吴茜, 倪林仙, 樊茂, 赵明波, 高丽. 昆明地区儿童非典型病原体感染病原学回顾性分析. 中国儿童保健杂志. 2009;17(06):708-10.
[1403] 吴翔. 海口地区小儿呼吸道病原体检测结果分析 [硕士]2014.
[1404] 吴宜恕, 张薇芬, 冯方波, 吴际. 莱姆病患者皮肤损害的临床特征. 中华医学杂志. 1997(10):72-3.
[1405] 吴益民, 张志强, 王洪军, 关国坤, 冯立, 王立强, 等. 东北部分地区蜱媒传染病血清学调查. 解放军预防医学杂志. 2006;24(4):300-.
[1406] 吴益民 魏, 刘昕昕,王光,胡玲美,杨青. 绥芬河地区三种蜱媒疾病的血清学调查. 解放军预

防医学杂志. 1997(05):34-6.
[1407] 吴泽刚, 李艳, 祝成亮, 刘映乐. 湖北地区儿童急性呼吸道感染病原体研究. 中华医院感染学杂志. 2011;21(10):2129-31.
[1408] 武建英 王. Q 热复发 1 例. 医学理论与实践. 1997(03):134-5.
[1409] 习宁. 人免疫球蛋白治疗森林脑炎疗效观察 [硕士]2023.
[1410] 习宁, 韩淑祯. 轻型森林脑炎合并急性脑梗死病例分析. 临床医学进展. 2022;12(8):7119-22.
[1411] 夏平松. 人粒细胞无形体病 6 例报道. 国际流行病学传染病学杂志. 2008;35(5):360.
[1412] 肖方震, 李丹萍, 徐国英, 陈阳, 邓艳琴. 福建部分地区人和动物嗜吞噬细胞无形体基因检测和序列分析. 中国人兽共患病学报. 2016;32(03):262-5.
[1413] 肖丽霞. 森林脑炎遗留双上肢瘫痪 1 例报告. 华北煤炭医学院学报. 2001;3(1):123-.
[1414] 肖伟年. 呼吸道感染病原体九联检与痰培养联合的临床应用. 当代医学. 2014;20(16):64-5.
[1415] 谢爱香. 呼吸道九项病原体检测在儿童呼吸道感染中的应用价值. 实验与检验医学. 2018;36(3):443-5.
[1416] 谢光顺, 全锦红, 董刚, 肖明德, 邓成涛, 王迪安. 某连发生一起森林脑炎的调查报告. 人民军医. 1990(05):8-9.
[1417] 谢国艳, 高志生, 秦云, 李星军, 谭永强. 上海崇明地区儿童急性下呼吸道感染的流行特点与临床特征分析. 诊断学理论与实践. 2016;15(04):410-4.
[1418] 谢鹤, 李贵才, 王朋朋, 吴淑庄, 罗昭云, 黄斌, 等. 潮州地区小儿急性下呼吸道感染病原学研究. 中国医学创新. 2013;10(11):116-8.
[1419] 谢红梅, 胡必杰, 马艳, 周春妹, 周昭彦, 黄声雷, 等. 1647 例呼吸道感染病原体的 IgM 抗体检测结果分析. 中华医院感染学杂志. 2012;22(12):2696-8.
[1420] 谢霖崇, 万康林, 郭衍, 许世锷, 张哲夫, 潘林祥, 等. 广东梅州市莱姆病自然疫源地调查. 中国病原生物学杂志. 2009;4(08):575-8.
[1421] 谢松松, 王远志, 鲁晓擘, 郑嵘炅, 周延, 左维泽, 等. 蜱传饶氏立克次体感染致脑膜炎 1 例. 中国感染与化疗杂志. 2018;18(04):431-3.
[1422] 谢晓菲. 海南不明原因发热患者 9 种致病菌分子流行病学调查及恙虫病东方体抗原蛋白表达 [硕士]2023.
[1423] 谢跃文, 辛焰. 武汉地区儿童急性呼吸道感染病原学研究. 临床血液学杂志. 2017;30(04):287-9.
[1424] 邢富昆, 彭志会, 高贵华, 张崛, 王连秀, 侯学霞, 等. 北京市昌平区莱姆病初步调查. 中国人兽共患病杂志. 2002(06):106-10.
[1425] 徐阔, 王婷, 刘静, 王秋霞, 许婷. 老年慢性支气管炎急性发作患者病原体检测结果分析. 标记免疫分析与临床. 2021;28(07):1131-4+45.
[1426] 徐梅, 王东晓, 梅淑芹. 森林脑炎 100 例临床分析. 中国实用内科杂志. 2004;24(6):327.
[1427] 徐能鹏, 熊怡祥, 万禧伟, 夏平松, 吴良俊, 王朝晖, 等. 人粒细胞无形体病六例临床分析. 临床内科杂志. 2008(11):746.
[1428] 徐琪毅, 李宏英, 李飞, 杨国锋, 张丽娟. 新疆伊犁州农村儿童媒介传播立克次体病血清流行病学调查. 中国媒介生物学及控制杂志. 2016;27(01):58-60.
[1429] 徐胜勇, 于学忠. 1 例危重症 Q 热患者诊治经过. 中华危重病急救医学. 2014;26(8):595-6.
[1430] 徐雪华, 尹海英, 张乃杰, 李金萍. 莱州市 1 例人粒细胞无形体病病例调查. 预防医学论坛. 2011;17(04):357-8.
[1431] 徐志强. 森林脑炎疾病的预防及护理措施分析. 养生保健指南. 2019(25):141.

[1432] 许丽. 5 例森林脑炎患者的急救与护理. 吉林医学. 2011;32(1):179.
[1433] 许莉莉, 程邦宁, 刘慧娟, 郝家砚, 江峤. 9693 例呼吸道感染患儿非典型病原体感染病原学检测结果分析. 中华疾病控制杂志. 2014;18(02):178-80.
[1434] 许庆梅, 哈斯额尔敦, 赵孝强, 王晶. 五味沙棘散治疗森林脑炎合并肺炎的理论初探. 中国民族医药杂志. 2015;21(2):68-9.
[1435] 玄德权, 伊世杰, 金昌吉. 森林脑炎临床特点观察及今后治疗的展望——附 82 例临床分析. 延边医学院学报. 1980(03):55-9.
[1436] 薛白, 刘洁, 胡志刚, 邹旭美. 呼吸道感染患者病原学调查分析. 中华医院感染学杂志. 2014;24(02):309-11.
[1437] 阎辉. 小儿莱姆病:90 例患儿的临床与流行病学特征. 国外医学流行病学传染病学分册. 1990(03):142-3.
[1438] 阎西革, 徐进杰, 花琛, 宋明洋, 胡乃宝. 2006-2012 年烟台市 10 种传染病季节性分布研究. 中国医院统计. 2014(3):180-2.
[1439] 杨德林, 王伟明, 曾奕民, 洪思让, 吕文辉, 谢汉瑜, 等. 泉州市莱姆病的发现及蜱媒与宿主的调查研究. 海峡预防医学杂志. 2005(06):12-4.
[1440] 杨发莲, 陈明华, 窦慧芬. 云南部分地区呼吸道感染患者中 Q 热感染调查. 中国人兽共患病杂志. 1994(5):51.
[1441] 杨久宇. 森林脑炎致心肌损害的调查研究 [硕士]: 内蒙古民族大学; 2012.
[1442] 杨娟. 六安地区慢性阻塞性肺疾病急性加重期患者呼吸道非典型病原微生物感染检测分析. 国际检验医学杂志. 2018;39(15):1867-9.
[1443] 杨丽清, 何义菊, 刘婷, 陈恩强. 急性 Q 热继发巨噬细胞活化一例. 华西医学. 2022;37(08):1278-80.
[1444] 杨敏, 刘诚, 李耀军. 儿童急性呼吸道感染 9 种病原体 IgM 抗体检测结果分析. 临床医学研究与实践. 2017;2(05):85-7.
[1445] 杨敏, 马晶, 杨舒婷, 黄国虹, 王昌敏. 宏基因组二代测序诊断急性 Q 热并脑膜脑炎 1 例. 临床检验杂志. 2024;42(01):73-4.
[1446] 杨敏怡, 岑丽莲. 呼吸道病原体九联检联合痰培养在小儿支气管肺炎诊断中的应用价值. 智慧健康. 2022;8(04):13-5.
[1447] 杨淑萍, 陈山虎, 杨森华. 森林脑炎后遗症治验. 中医药信息. 1999(03):53.
[1448] 杨晓萍, 石云, 关碧玮. 红水河龙滩水库拟淹没区人群立克次氏体病血清流行病学调查. 同济医科大学学报. 1991(04):277-9.
[1449] 杨新民，雍晓娥，张彦. 一家 4 例莱姆病报告. 中国人兽共患病杂志. 1995(03):9.
[1450] 杨修军, 王春生, 万康林, 石端平, 葛延辉, 刘文祥, 等. 长白山区莱姆病调查与实验室分析. 中国媒介生物学及控制杂志. 2001(02):115-7.
[1451] 杨秀峰, 张哲夫, 路瑾萍, 张金声, 郑理, 侯学霞, 等. 大兴安岭南段莱姆病的调查. 中国媒介生物学及控制杂志. 1992(01):39-41.
[1452] 杨艳. 森林脑炎患者淋巴细胞亚群分类及细胞因子变化水平的临床研究 [硕士]2017.
[1453] 杨宇琦, 张通, 刘丽旭, 何静杰, 杨凌宇, 公维军, 等. 森林脑炎康复治疗 1 例报道. 中国康复理论与实践. 2011;17(11):1076-7.
[1454] 杨玉芝, 孙成斋, 王振海, 朱礼业, 何素勤, 姜兰清. 淮北地区人畜间 Q 热感染情况调查报告. 中国人兽共患病杂志. 1993(06):19-20.
[1455] 杨育松, 吕燕宁, 陈永亮. 北京市密云区关节炎患者莱姆病血清学调查. 中国媒介生物学及控制杂志. 2017;28(5):490-1.

[1456] 杨湛. 儿童急性下呼吸道感染病原体分布及与年龄气候等因素的相关性分析 [硕士]2018.
[1457] 杨志宁. 某医院下呼吸道感染儿童血清病原体 IgM 抗体结果分析 [硕士]2023.
[1458] 杨重飞, 朱澍, 康飞科, 曹晓瑞, 朱庆生. 贝纳柯克斯体假体周围感染一例报告及系统文献复习. 中华骨科杂志. 2023;43(3):185-90.
[1459] 姚婷, 艾洪武, 余星, 孙红, 伍仕敏, 章晓联. 22 990 例儿童急性呼吸道感染的病原学及流行病学调查. 武汉大学学报（医学版）. 2016;37(6):1018-22,30.
[1460] 叶继斌, 胡传松, 王琦, 陈远山, 方立, 张海涛, 等. 利福霉素钠注射剂治疗人无形体病及临床总结——附 55 例患者临床分型分析. 中华实验和临床感染病杂志（电子版）. 2010;4(3):312-5.
[1461] 叶尚仪, 李香淑, 郑善子. 延边地区 130 例森林脑炎回顾性分析. 延边大学医学学报. 2020;43(03):199-201.
[1462] 叶婷, 刘靳波, 邓剑, 代碧珍. 泸州地区冬春季儿童呼吸道感染病原体研究. 现代医药卫生. 2012;28(24):3687-8,90.
[1463] 叶曦, 王榕峰, 李国伟. 厦门地区部分人群莱姆病感染及蜱媒调查. 预防医学情报杂志. 2007(03):282-3.
[1464] 殷楚强, 王海龙, 任宪锋, 王月磊, 沈峰, 王亭. 宏基因组二代测序技术在脊柱感染病原微生物诊断中的应用. 中国脊柱脊髓杂志. 2022;32(02):141-8.
[1465] 尹海波 尹, 王孝功. 森林脑炎病人心电图改变(附 229 例分析). 黑龙江医学. 2000(11):13.
[1466] 于建武, 孙丽杰, 赵勇华, 康鹏, 高杰, 李树臣. 森林脑炎 79 例流行病学和临床特征回顾性分析. 中华传染病杂志. 2009;27(5):297-300.
[1467] 于莉, 张敏, 李雅慧, 苏显都. 海南西部地区儿童非典型病原体感染病原学分析. 中国热带医学. 2013;13(12):1520-1+4.
[1468] 于永慧, 王涛, 罗声栋, 冯乐, 孙志会, 宋立华. Q 热性心内膜炎 1 例抗I相和II相抗原 IgG 抗体的跟踪监测报道. 军事医学. 2017;41(02):160-1.
[1469] 俞树荣, 邵兰, 李芹阶, 余国泉, 赵蜀崖, 陈允凤, 等. 海南省部分人群抗 Q 热及其他立克次体病抗体的调查. 中华医学杂志. 1989;69(9):534-5.
[1470] 喻艳林, 葛宗成, 杨江华, 涂雄文, 李慧芬, 邵体红, 等. 10 例人粒细胞无形体病暴发流行报告. 中华传染病杂志. 2010;28(3):168-71.
[1471] 詹前美. 呼吸道感染 9 项病原体 IgM 抗体检测分析. 临床和实验医学杂志. 2013;12(09):662-3.
[1472] 詹小妹, 陈小建, 符荣益. 1 594 例下呼吸道感染儿童病例病原学特征分析. 华南预防医学. 2022;48(3):355-7,62.
[1473] 张爱荣, 范秋霞, 许青霞. 2776 例儿童急性呼吸道感染病原学检测. 中国卫生检验杂志. 2016;26(8):1084-6.
[1474] 张鲍虎, 张莉, 杨书才, 周杰. 坪山区 9 种呼吸道病原体 IgM 抗体调查结果分析. 中国医药科学. 2019;9(12):185-8.
[1475] 张大荣, 杭恒贵, 刘红, 顾莉莉, 俞正楚, 罗兆庄. 安徽省人群莱姆病血清流行病学调查. 中华流行病学杂志. 1996(01):24.
[1476] 张大荣, 杭恒贵, 李群. 西藏林芝地区人群莱姆病感染初步调查. 中国人兽共患病杂志. 1997(02):70-16.
[1477] 张当安, 吕家锐. 2009 年卢氏县人和家畜立克次体病血清流行病学调查. 河南预防医学杂志. 2013;24(05):418-21.
[1478] 张方祥, 王凤红. 神经系统莱姆病 28 例临床分析与诊断. 中国媒介生物学及控制杂志.

2011;22(03):285.
[1479] 张芳，刘增加. 我国西北部分地区 Q 热分子流行病学调查. 中国病原生物学杂志. 2011;06(3):183-5,235.
[1480] 张桂林, 刘然, 孙响, 郑旸, 刘晓明, 赵焱, 等. 新疆夏尔希里自然保护区蜱传脑炎疫源地调查.
[1481] 张海莲, 邵兰, 佟忠维, 赵树萱, 卢孝东, 肖友书. 海南岛人群立克次体病的血清抗体检测及其分型研究. 中华流行病学杂志. 1989;10(Z3):12-5.
[1482] 张海林, 张云智, 杨卫红, 章域震, 米竹青, 黄文丽, 袁庆虹, 等. 云南省澜沧江下游地区人及动物血清虫媒病毒抗体调查. 医学动物防制. 2004(04):207-11.
[1483] 张虹霞. 呼吸道病原体九联检在儿童急性呼吸道感染中的临床应用. 甘肃医药. 2017;36(6):477,503.
[1484] 张慧玲, 孙永, 王建军. 庐江县新型布尼亚病毒及人粒细胞无形体疫源地调查. 中华疾病控制杂志. 2015;19(03):273-6.
[1485] 张继军, 刘增加, 张芳, 宫占威, 刘寒. 阿勒泰地区莱姆病螺旋体分子流行病学调查研究. 寄生虫与医学昆虫学报. 2011;18(1):34-7.
[1486] 张健之, 毕德增, 贺金荣, 陈敏, 王士明, 郭衍, 等. 粤东丰溪林场斑点热疫源地. 中国人兽共患病杂志. 1999(02):38-40.
[1487] 张健之, 郭衍, 贺金荣, 许世锷, 潘林祥. 广东省首次发现斑点热群立克次体的感染. 疾病监测. 1998(08):8-11.
[1488] 张君怡，吴东，方卫纲，范洪伟. 慢性 Q 热并发心内膜炎二例. 中华内科杂志. 2010;49(8):707-8.
[1489] 张丽娟, 崔峰, 王玲, 张玲, 张景山, 杨淑霞, 等. 山东省沂源县无形体病实验室调查分析. 传染病信息. 2009;22(01):21-5.
[1490] 张楠. 我国北方部分地区蜱传无形体病的分子流行病学调查 [硕士]2021.
[1491] 张启恩, 艾承绪, 刘玉堂, 徐焕章, 李春明, 李国艾, 等. 新疆西北部地区蜱媒斑点热、Q 热、斑疹伤寒和野兔热的血清学调查. 军事医学科学院院刊. 1982(04):441-6.
[1492] 张启恩, 艾承绪, 刘玉堂, 徐焕章, 李春明, 李国艾, 等. 新疆西北地区蜱媒斑点热、Q 热、斑疹伤寒和土拉弗氏菌病的血清学调查. 中华流行病学杂志. 1983;04(2):69.
[1493] 张倩, 周敬静, 樊茂. 某地区 6 岁以下儿童非典型肺炎病原体感染状况回顾分析. 国际检验医学杂志. 2011;32(14):1627-8.
[1494] 张瑞梅, 王美艳. 一起森林脑炎暴发的调查报告. 疾病监测. 1999;14(4):149.
[1495] 张树林, 王晓峰. Q 热 3 例误诊分析. 中华传染病杂志. 1989;07(3):179-80.
[1496] 张素美, 杨涤. 以言语障碍为主要症状的蜱传森林脑炎一例. 中华实验和临床感染病杂志(电子版). 2011;5(04):480-1.
[1497] 张婷, 牛俊奇, 姜艳芳, 王峰. 吉林省新疆出血热病毒感染的临床研究. 中华实验和临床感染病杂志(电子版). 2007(03):158-60.
[1498] 张卫兴. 塔城蜱病毒 2 的分离鉴定及流行病学研究 [硕士]2019.
[1499] 张晓东, 喻为人, 石成虎, 王孟君, 蒋茨, 张燕妮, 等. 人粒细胞无形体病误诊为心肌梗死 1 例. 内科急危重症杂志. 2014;20(01):67.
[1500] 张晓光, 崔巍, 佟艳秋. 森林脑炎致眼部病 30 例临床报告. 内蒙古医学杂志. 2004(08):581-2.
[1501] 张晓光, 佟艳秋, 娜仁, 孙忠伟. 森林脑炎与莱姆病双重感染致眼部病变的报告. 中国实用眼科杂志. 2004(05):329.

[1502] 张晓鹏，王大虎，粟多寿，罗远琼. 铧尖地区莱姆病调查. 中国兽医科技. 1994(10):15-6.
[1503] 张雄, 钟玉涛. 森林脑炎所致精神障碍 13 例临床观察. 医学动物防制. 2011;27(01):69-70.
[1504] 张秀春, 张立霞, 李伟红, 王誓闻, 孙玉兰, 王圆圆, 等. 北京市农村人群及家畜嗜吞噬无形体和查菲埃立克体感染状况调查. 中华流行病学杂志. 2012;33(5):517-20.
[1505] 张雪晗, 刘慧婷, 王玉, 范洪伟, 王振捷, 焦洋. 慢性 Q 热 12 例临床特征分析. 中华全科医师杂志. 2023;22(10):1062-7.
[1506] 张毅, 李水霞, 郭丽娜. 呼吸道九联检、痰培养及药敏联合检测在社区获得性肺炎诊治中的临床应用. 包头医学院学报. 2015;31(9):72-4.
[1507] 张永根, 史永林, 刘红, 曹明华, 胡万富, 王俊, 等. 安徽省不同地区人群和家畜 Q 热血清流行病学调查. 安徽预防医学杂志. 2010;16(02):87-8+98.
[1508] 张勇, 张金彪, 宋明玉, 王鹏飞, 王晶, 孙超, 等. 以神经系统症状首发的人嗜吞噬细胞无形体病四例. 中国现代神经疾病杂志. 2009;9(04):402-3.
[1509] 张玉马. 关于呼伦贝尔草原地区森林脑炎 1 列特殊症状报告. 世界最新医学信息文摘（连续型电子期刊）. 2015(26):175-.
[1510] 张玉云, 范小莉, 闫博. 人粒细胞无形体病并发弥漫性血管内凝血死亡 1 例. 中华临床感染病杂志. 2009;2(1):51-2.
[1511] 张媛春, 李六九, 雷素娟, 姚云波, 陈良, 朱林, 等. 云南玉溪地区莱姆病调查. 中国人兽共患病杂志. 2000(06):107-8.
[1512] 张钊冠, 赵春燕, 倪朝辉, 王鑫磊, 黄红兰. 长春地区儿童急性呼吸道感染病原体分析. 中国妇幼保健. 2014;29(34):5600-2.
[1513] 张哲夫, 万康林, 冯文祥, 王梦祥, 朱桂凤, 尚振忠, 等. 牡丹江林区莱姆病分布的地理特点. 中华流行病学杂志. 1991;12(03):154-7.
[1514] 张哲夫, 张金声, 朱挂凤, 张知德, 田登安, 万康林, 等. 我国东北林区莱姆病的调查. 中华流行病学杂志. 1989;10(05):261-4.
[1515] 张哲夫, 万康林, 张金声, 朱桂凤, 窦桂兰, 李牧青, 等. 我国莱姆病的流行病学和病原学研究. 中华流行病学杂志. 1997(01):8-11.
[1516] 张之伦, 罗云秋, 张云, 刘祖义, 于长水, 林美惠. 天津市人群中立克次氏体感染状况调查. 中国公共卫生. 1992(06):253-4.
[1517] 张知德, 张哲夫, 尚振忠, 霍秋波, 曹伯良. 从森林脑炎疑似患者血清中检测抗莱姆病螺旋体抗体的调查. 中华流行病学杂志. 1989;10(Z3):333-5.
[1518] 张知德 陈, 梅建华,王东波,林仁卫,吴文有,吕连珍,王聪,石观平. 浙南地区人群莱姆病血清流行病学研究. 中国公共卫生. 2001(11):79-80.
[1519] 张忠龙, 陈凤娇. 福州地区儿童呼吸道感染非典型病原体检测分析. 中外医疗. 2019;38(33):5-7.
[1520] 赵春生，蒋廉华，陈维军，陆振豸. 海南省部分地区人血清中新疆出血热病毒抗体的调查. 中国人兽共患病杂志. 1996(04):6+10.
[1521] 赵浩宏, 谷培云, 方媚, 王超, 栗绍刚, 齐文杰. 骨髓血涂片镜检诊断人粒细胞无形体病 1 例报告. 中国临床医学. 2022;29(2):293-6.
[1522] 赵慧. Q 热立克次体在人群间的分布. 中华预防医学杂志. 1991;25(1):59.
[1523] 赵静, 范志磊, 李铁锋. 九项呼吸道病原体抗体 IgM 联合检测在呼吸道感染疾病中的临床应用. 健康之友. 2020(6):71.
[1524] 赵俏猷, 伍燕青, 黎北信, 许昌, 黄丽霖, 黄梅霞. 茂名地区儿童非典型肺炎病原体流行病学调查. 国际检验医学杂志. 2016;37(9):1226-7.

[1525] 赵全良. 大兴安岭林区森林脑炎致眼部病变临床分析. 中国民族医药杂志. 2008;14(11):12-3.
[1526] 赵荣山. 美国爆发一起由猫引起的 Q 热. 国外医学流行病学传染病学分册. 1992(01):48.
[1527] 赵万辉, 刘义庆, 王泽筠, 亓琳, 耿丽丽, 张炳昌, 等. 2013-2015 年山东某院呼吸道感染患者 9 种病原体血清 IgM 抗体检测情况. 检验医学与临床. 2018;15(15):2211-3.
[1528] 赵孝强, 马永菊, 许庆梅. 机械通气治疗合并呼吸肌麻痹的森林脑炎患者 53 例临床体会. 医学信息. 2015(30):357-.
[1529] 赵延佳, 梁瑛. 1 例 Q 热病人的护理. 护理研究. 2011;25(30):2817-8.
[1530] 赵焱, 刘然, 张桂林, 刘晓明, 孙响, 郑重, 等. 新疆北部某边防部队蜱传疾病血清流行病学调查. 解放军预防医学杂志. 2014;32(04):324-5.
[1531] 赵智强. 蒙药丹巴--1 号治疗森林脑炎疗效观察 [硕士]: 内蒙古民族大学; 2023.
[1532] 郑加田, 张源潮, 潘正论. 莱姆病患者螺旋体 IgM 抗体多次复查升高 1 例. 慢性病学杂志. 2013;14(09):717-8.
[1533] 郑姣, 王文妍, 曹力. 1 例蜱虫咬伤致森林脑炎的护理. 中国急救复苏与灾害医学杂志. 2012;7(12):1172-3.
[1534] 郑龙荣. 九项呼吸道感染病原体 IgM 抗体早期检测的临床应用. 中外医学研究. 2018;16(11):52-3.
[1535] 郑启秋. 平果县海明林场林区莱姆病的调查报告. 右江医学. 1992(03):139-40.
[1536] 郑世玲, 陈凯霞. 人粒细胞无形体病 15 例的护理. 医药世界. 2009;11(08):449-50.
[1537] 郑淑鹏. 美国发生一次与猫相关的 Q 热流行. 国外医学情报. 1992(03):15.
[1538] 郑智武. 延边地区 2012-2021 年 243 例森林脑炎患者临床特征及转归分析 [硕士]2022.
[1539] 郑重, 张桂林, 曾凡本, 孙响, 刘然, 刘晓明, 等. 新疆地区人群森林脑炎血清流行病学调查. 解放军预防医学杂志. 2016;34(06):806-8.
[1540] 钟添长, 李柯莹, 李家春. 多西环素治疗急性 Q 热失败 1 例. 中国感染控制杂志. 2023;22(9):1110-3.
[1541] 钟宣贤, 王笑中. 森林脑炎后遗症高压氧治疗 1 例. 海军医学. 1985(03):73.
[1542] 钟应洪, 许德馨. 拉萨市 Q 热血清学调查. 中华流行病学杂志. 1982;03(1):37.
[1543] 周宝桐, 王焕玲, 范洪伟, 刘晓清, 李太生. Q 热心内膜炎四例并文献复习. 中华内科杂志. 2014;53(3):184-7.
[1544] 周经纬, 夏岥, 崔昌星, 姜涛, 马承泰. 血液宏基因组二代测序检测确诊 Q 热 1 例. 中国感染与化疗杂志. 2022;22(04):484-7.
[1545] 周莲, 符明昌. 海南南部地区儿童急性呼吸道感染病原学特征分析. 中国热带医学. 2016;16(07):713-6.
[1546] 周麟玲, 常爱娜. 人粒细胞无形体病一例. 中华传染病杂志. 2009;27(9):565.
[1547] 周明行, 胡媛媛, 樊成辉, 李惠珍, 叶军, 蔡雄, 等. 上海市区居民立克次体感染初步检测. 中华流行病学杂志. 1989;10(Z3):33-5.
[1548] 周全彦, 赵慕愚, 黄自英, 刘骊生, 王文殊, 万康林. 四川省健康人群莱姆病的血清流行病学调查. 预防医学情报杂志. 1991(01):46-7+38.
[1549] 周伟. 森林脑炎患者血清中 VEGF 和 sICAM-1 水平的研究及其预后相关因素的分析 [硕士]: 内蒙古民族大学; 2017.
[1550] 周晓茵. 1996～2003 年牡丹江地区莱姆病临床及流行病学分析. 中国寄生虫病防治杂志. 2005(02):159.
[1551] 周新荣, 王天祥, 刘栓奎, 吉保新, 窦君, 党荣理, 等. 西藏阿里地区自然疫源性疾病血清

流行病学调查. 中国媒介生物学及控制杂志. 1998(02):51-3.
[1552] 周祖木. 法国首次从病人分离出斯洛伐克立克次体. 国外医学(流行病学传染病学分册). 2003(02):125-6.
[1553] 朱华, 陈宗云, 黄爱兰. 6449 例儿童 9 种呼吸道病原体感染情况分析. 海峡预防医学杂志. 2022;28(2):91-3.
[1554] 朱华民, 林柏杏, 包世杰, 孙志强. 急性 Q 热伴噬血细胞综合征 1 例报道并文献复习. 现代医药卫生. 2020;36(11):1772-4.
[1555] 朱丽芳, 顾大磊. 急性下呼吸道感染患儿非典型病原体检测结果分析. 中国乡村医药. 2015(5):67-8.
[1556] 朱利明, 罗浩元, 刘集鸿, 周潇, 朱少美, 张倩. 急性呼吸道感染患儿非典型病原体特异性 IgM 抗体检测结果分析. 国际检验医学杂志. 2017;38(11):1537-9.
[1557] 朱少美, 钟舒芫. 广东省惠州市呼吸道感染病原体特征分析. 实用医技杂志. 2022;29(12):1250-4.
[1558] 朱淑琴, 洪克晋, 张艳平. 森林脑炎合并莱姆病葡萄膜病变 1 例. 中国媒介生物学及控制杂志. 1999(03):67.
[1559] 朱香花. 一例森林脑炎患者的护理体会. 养生保健指南. 2016(25):215-.
[1560] 朱晓宇, 魏建春, 张慧娟, 俞东征. 一次不明原因发热疫情中病人血清的 Q 热抗体检测. 中国卫生检验杂志. 2008(06):1130-1.
[1561] 朱雄, 侯学霞, 于莉, 张琳, 陈运优, 苗广青, 等. 海南省西部地区莱姆病血清学调查. 中国人兽共患病学报. 2020;36(04):313-6+9.
[1562] 朱雄, 张琳, 侯学霞, 耿震, 陈海, 陈婷, 等. 海南省关节炎和神经系统疾病患者莱姆病调查. 中国人兽共患病学报. 2015;31(04):353-6.
[1563] 朱莹莹. 丙种球蛋白治疗森林脑炎的临床研究 [硕士]: 内蒙古民族大学; 2011.
[1564] 朱有, 韩冰, 李绍民, 贺永文, 冯澜, 代立娟, 等. 人粒细胞无形体病 45 例临床分析. 临床医学. 2010;30(04):47-50.
[1565] 朱玉光 刘. 莱姆氏病误诊蛛网膜下腔出血 1 例报告. 实用放射学杂志. 2001(11):880.
[1566] 宗定国, 蒋岳新, 王连城, 张玉民, 金花, 朱敏杰, 等. 新疆博尔塔拉地区几种自然疫源性疾病的血清学调查. 地方病通报. 1987(03):5-8.
[1567] 宗晓龙, 马利锋, 李真玉, 韩悦, 田雨鑫, 赵琪, 等. 2013—2018 年天津地区住院儿童急性呼吸道感染病原体流行特征分析. 天津医药. 2020;48(4):313-9.
[1568] 邹甜甜, 费安兴, 柯海燕, 陆栋鹏, 李胜. 黄石地区 2520 例儿童呼吸道病原体检测结果分析. 世界最新医学信息文摘（连续型电子期刊）. 2020;20(85):175-6.
[1569] 俎红伟, 郝爱庆, 张贵显. 莱姆病引起周围性面瘫一例. 临床耳鼻咽喉科杂志. 1994(02):112.
[1570] 左玲燕, 徐珊, 谢静. 1 例莱姆病患者的护理. 现代护理. 2006(26):2476-7.
[1571] 左万超, 瞿新, 梁荣鑫, 李莲. 9 项呼吸道病原体联合检测在儿科呼吸道疾病诊断中的应用. 检验医学与临床. 2017;14(01):60-2.
[1572] 俄罗斯联邦爆发克里米亚—刚果出血热. 口岸卫生控制. 1999(04):44.
[1573] 俄罗斯 8 人死于蜱传脑炎. 口岸卫生控制. 2007(04):46.
[1574] K. S, G. S, K. V, 谢琰臣. 运动和感觉多发性神经炎合并远端传导障碍为急性 Rickettsia conorii 感染的罕见并发症. 世界核心医学期刊文摘(神经病学分册). 2005(10):41.
[1575] Stone J.H, DK, Aram G., Dumler J. S., 王亭忠. 人单核细胞埃里希体病. 世界核心医学期刊文摘(心脏病学分册). 2005(04):17-8.

[1576] 刘蔼年, 刘海林, 化冰. Lyme 病眼部表现 30 例分析. 中华眼科杂志. 1993;29(5):271-3.

table S5: The PRISMA Checklist

We followed the Preferred Reporting Items for Systematic Reviews and Meta-Analyses (PRISMA) statement in the conduct of this study.

Section and Topic	Item #	Checklist item	Location where item is reported
**TITLE**			
Title	1	Identify the report as a systematic review.	1
**ABSTRACT**			
Abstract	2	See the PRISMA 2020 for Abstracts checklist.	2
**INTRODUCTION**			
Rationale	3	Describe the rationale for the review in the context of existing knowledge.	3-4
Objectives	4	Provide an explicit statement of the objective(s) or question(s) the review addresses.	3-4
**METHODS**			
Eligibility criteria	5	Specify the inclusion and exclusion criteria for the review and how studies were grouped for the syntheses.	4-5; table S1-S3
Information sources	6	Specify all databases, registers, websites, organisations, reference lists and other sources searched or consulted to identify studies. Specify the date when each source was last searched or consulted.	4; Fig 1;Fig S1; table S1-S3
Search strategy	7	Present the full search strategies for all databases, registers and websites, including any filters and limits used.	4; Fig 1;Fig S1;Table S1

PRISMA

PRISMA 2020 Checklist

Section and Topic	Item #	Checklist item	Location where item is reported
Selection process	8	Specify the methods used to decide whether a study met the inclusion criteria of the review, including how many reviewers screened each record and each report retrieved, whether they worked independently, and if applicable, details of automation tools used in the process.	4; table S2
Data collection process	9	Specify the methods used to collect data from reports, including how many reviewers collected data from each report, whether they worked independently, any processes for obtaining or confirming data from study investigators, and if applicable, details of automation tools used in the process.	4-5
Data items	10a	List and define all outcomes for which data were sought. Specify whether all results that were compatible with each outcome domain in each study were sought (e.g. for all measures, time points, analyses), and if not, the methods used to decide which results to collect.	6
	10b	List and define all other variables for which data were sought (e.g. participant and intervention characteristics, funding sources). Describe any assumptions made about any missing or unclear information.	table S3
Study risk of bias assessment	11	Specify the methods used to assess risk of bias in the included studies, including details of the tool(s) used, how many reviewers assessed each study and whether they worked independently, and if applicable, details of automation tools used in the process.	4-5
Effect measures	12	Specify for each outcome the effect measure(s) (e.g. risk ratio, mean difference) used in the synthesis or presentation of results.	6
Synthesis methods	13a	Describe the processes used to decide which studies were eligible for each synthesis (e.g. tabulating the study intervention characteristics and comparing against the planned groups for each synthesis (item #5)).	6
	13b	Describe any methods required to prepare the data for presentation or synthesis, such as handling of missing summary statistics, or data conversions.	6
	13c	Describe any methods used to tabulate or visually display results of individual studies and syntheses.	7
	13d	Describe any methods used to synthesize results and provide a rationale for the choice(s). If meta-analysis was performed, describe the model(s), method(s) to identify the presence and extent of statistical heterogeneity, and software package(s) used.	6
	13e	Describe any methods used to explore possible causes of heterogeneity among study results (e.g. subgroup analysis, meta-regression).	N/A
	13f	Describe any sensitivity analyses conducted to assess robustness of the synthesized results.	N/A
Reporting bias assessment	14	Describe any methods used to assess risk of bias due to missing results in a synthesis (arising from reporting biases).	N/A
Certainty assessment	15	Describe any methods used to assess certainty (or confidence) in the body of evidence for an outcome.	6
**RESULTS**			

PRISMA

PRISMA 2020 Checklist

Section and Topic	Item #	Checklist item	Location where item is reported
Study selection	16a	Describe the results of the search and selection process, from the number of records identified in the search to the number of studies included in the review, ideally using a flow diagram.	7-8; fig S1
	16b	Cite studies that might appear to meet the inclusion criteria, but which were excluded, and explain why they were excluded.	N/A
Study characteristics	17	Cite each included study and present its characteristics.	8-10; Data 2-4
Risk of bias in studies	18	Present assessments of risk of bias for each included study.	N/A
Results of individual studies	19	For all outcomes, present, for each study: (a) summary statistics for each group (where appropriate) and (b) an effect estimate and its precision (e.g. confidence/credible interval), ideally using structured tables or plots.	8; Fig 4
Results of syntheses	20a	For each synthesis, briefly summarise the characteristics and risk of bias among contributing studies.	N/A
	20b	Present results of all statistical syntheses conducted. If meta-analysis was done, present for each the summary estimate and its precision (e.g. confidence/credible interval) and measures of statistical heterogeneity. If comparing groups, describe the direction of the effect.	8; Fig 4
	20c	Present results of all investigations of possible causes of heterogeneity among study results.	N/A
	20d	Present results of all sensitivity analyses conducted to assess the robustness of the synthesized results.	N/A
Reporting biases	21	Present assessments of risk of bias due to missing results (arising from reporting biases) for each synthesis assessed.	N/A
Certainty of evidence	22	Present assessments of certainty (or confidence) in the body of evidence for each outcome assessed.	8; Fig 4
**DISCUSSION**			
Discussion	23a	Provide a general interpretation of the results in the context of other evidence.	11-12
	23b	Discuss any limitations of the evidence included in the review.	14
	23c	Discuss any limitations of the review processes used.	14
	23d	Discuss implications of the results for practice, policy, and future research.	15

PRISMA

PRISMA 2020 Checklist

Section and Topic	Item #	Checklist item	Location where item is reported
**OTHER INFORMATION**			
Registration and protocol	24a	Provide registration information for the review, including register name and registration number, or state that the review was not registered.	N/A
	24b	Indicate where the review protocol can be accessed, or state that a protocol was not prepared.	N/A
	24c	Describe and explain any amendments to information provided at registration or in the protocol.	N/A
Support	25	Describe sources of financial or non-financial support for the review, and the role of the funders or sponsors in the review.	15-16
Competing interests	26	Declare any competing interests of review authors.	16
Availability of data, code and other materials	27	Report which of the following are publicly available and where they can be found: template data collection forms; data extracted from included studies; data used for all analyses; analytic code; any other materials used in the review.	16

From: Page MJ, McKenzie JE, Bossuyt PM, Boutron I, Hoffmann TC, Mulrow CD, et al. The PRISMA 2020 statement: an updated guideline for reporting systematic reviews. BMJ 2021;372:n71. doi: 10.1136/bmj.n71.

This work is licensed under CC BY 4.0. To view a copy of this license, visit https://creativecommons.org/licenses/by/4.0/

Table S6: Environmental and meteorological variables downloaded for ecological modeling for *H. anatolicum*

Variable	Description	Source
BIO1	Annual mean temperature (°C)	
BIO2	Mean diurnal range (Mean of monthly (max temp-min temp)) (°C)	
BIO3	Isothermality (BIO2/BIO7)(*100)	
BIO4	Temperature seasonality (standard deviation*100)	
BIO5	Max temperature of warmest month (°C)	
BIO6	Min temperature of coldest month (°C)	
BIO7	Annual range of temperature (BIO5- BIO6) (°C)	
BIO8	Mean temperature of wettest quarter (°C)	
BIO9	Mean temperature of driest quarter (°C)	
BIO10	Mean temperature of warmest quarter (°C)	WorldClim database
BIO11	Mean temperature of coldest quarter (°C)	
BIO12	Annual precipitation (mm)	
BIO13	Precipitation of wettest month (mm)	
BIO14	Precipitation of driest month (mm)	
BIO15	Precipitation seasonality(Coefficient of variation)	
BIO16	Precipitation of wettest quarter (mm)	
BIO17	Precipitation of driest quarter (mm)	
BIO18	Precipitation of warmest quarter (mm)	
BIO19	Precipitation of coldest quarter (mm)	
Elevation	Elevation	
Slope	Slope	
Aspect	Aspect	
Percent tree cover	Percent tree cover	Geospatial Information Authority of Japan
Land cover	Land cover	

Legend for Land Cover type

Code	Class Name
1	Broadleaf Evergreen Forest
2	Broadleaf Deciduous Forest
3	Needleleaf Evergreen Forest
4	Needleleaf Deciduous Forest
5	Mixed Forest
6	Tree Open
7	Shrub
8	Herbaceous
9	Herbaceous with Sparse Tree/Shrub
10	Sparse vegetation
11	Cropland
12	Paddy field
13	Cropland/Other Vegetation Mosaic
14	Mangrove
15	Wetland
16	Bare area, consolidated (gravel, rock)
17	Bare area, unconsolidated (sand)
18	Urban
19	Snow/Ice
20	Water bodies

Table S7: Known occurrence locations of *H. anatolicum* used for ecological niche modeling*

Data Source	Refence/ GBIF ID	Country	First-level Administrative Division	Second-level Administrative Division	Longitude	Latitude
Literature	36	Pakistan	Khyber Pakhtunkhwa	Malakand	72.241392	34.666486
Literature	36	Pakistan	Khyber Pakhtunkhwa	Hazara	73.301944	34.534722
Literature	36	Pakistan	Khyber Pakhtunkhwa	Mardan	72.026447	34.118217
Literature	40	Sudan	Al Gezira	Sharg Aj Jazirah	33.516667	14.916667
Literature	40	Sudan	West Kordofan	En Nahud	28.416667	12.700000
Literature	65	Turkey	Kayseri	Kayseri	34.400000	38.933333
Literature	66	Egypt	Beni Suef Governorate	Beni-Suef	31.400000	30.130000
Literature	99	Saudi Arabia	Riyadh Province	Riyadh Governorate	45.500000	24.000000
Literature	99	Saudi Arabia	Qassim Province	Al-Rass	42.866667	25.800000
Literature	112	Pakistan	Khyber Pakhtunkhwa	Dera Ismail Khan	69.444000	32.542300
Literature	112	Pakistan	Khyber Pakhtunkhwa	Dera Ismail Khan	70.163800	32.581000
Literature	128	Iran	Sistan and Baluchestan	Sarbaz	66.994775	30.584808
Literature	128	Iran	Sistan and Baluchestan	Chabahar	61.138600	25.389122
Literature	149	Pakistan	Khyber Pakhtunkhwa	Malakand	71.247501	30.330847
Literature	161	Iran	Golestan	Azadshahr	55.065833	37.163333
Literature	166	Turkey	Manisa	Golmarmara	27.585770	38.391440
Literature	166	Turkey	Manisa	Alasehir	28.254540	38.251370
Literature	166	Turkey	İzmir	Tire	27.38934	38.00247
Literature	166	Turkey	İzmir	Kınık	27.27303	38.57901
Literature	166	Turkey	İzmir	Aliaga	27.03102	38.45697
Literature	166	Turkey	Aydin	Soke	27.313810	37.401380
Literature	166	Turkey	Aydin	Aydin	27.455810	37.488360
Literature	166	Turkey	Aydin	Yenipazar	28.055130	37.459430
Literature	166	Turkey	Aydin	Cine	28.034910	37.301310
Literature	169	India	Punjab	Patiala	75.500000	30.150000
Literature	170	Iran	Ardabil	Pars-abad	47.418639	39.445611
Literature	170	Iran	Ilam	Ilam	46.074139	33.780056
Literature	170	Iran	North Khorasan	Raz and Jargaland	57.104528	37.942333
Literature	170	Iran	Sistan and Baluchestan	Chabahar	61.402778	26.240972
Literature	170	Iran	Zanjan	Ijrud	48.598444	36.100056
Literature	202	Sudan	Sennar	Sennar	33.616667	13.550000
Literature	202	Sudan	Sennar	Dinder	34.200000	13.733333
Literature	202	Sudan	Sennar	Um Banein	33.950000	13.066667
Literature	208	Lebanon	Nabatieh	Bint Jbeil	35.240000	33.011667
Literature	208	Lebanon	Nabatieh	Bint Jbeil	35.570000	33.330000

Literature	231	Iran	Golestan	Bandar-e Gaz	54.115420	36.882340
Literature	231	Iran	Golestan	Bandar-e Gaz	54.080590	36.855620
Literature	231	Iran	Golestan	Gomishan	54.079120	37.077380
Literature	231	Iran	Golestan	Gomishan	54.095800	36.997640
Literature	231	Iran	Golestan	Gomishan	54.085280	36.965030
Literature	231	Iran	Golestan	Bandar-e Gaz	54.049040	36.824610
Literature	231	Iran	Golestan	Kordkuy	54.110330	36.795590
Literature	231	Iran	Golestan	Bandar-e Gaz	54.030550	36.792050
Literature	231	Iran	Golestan	Bandar-e Torkaman	54.275450	36.984840
Literature	231	Iran	Golestan	Bandar-e Torkaman	54.235210	36.972490
Literature	231	Iran	Golestan	Kordkuy	54.176090	36.908650
Literature	231	Iran	Golestan	Bandar-e Gaz	53.938770	36.780690
Literature	231	Iran	Golestan	Bandar-e Gaz	53.908970	36.741840
Literature	231	Iran	Golestan	Bandar-e Gaz	53.867830	36.733400
Literature	231	Iran	Golestan	AqQala	54.618210	37.134870
Literature	231	Iran	Golestan	AqQala	54.665520	37.150190
Literature	231	Iran	Golestan	AqQala	54.645690	37.106860
Literature	231	Iran	Golestan	Gomishan	54.572180	37.413210
Literature	231	Iran	Golestan	Gomishan	54.651350	37.346860
Literature	231	Iran	Golestan	Gomishan	54.515050	37.323880
Literature	231	Iran	Golestan	AqQala	54.612510	37.022470
Literature	231	Iran	Golestan	Gorgan	54.619660	36.982690
Literature	231	Iran	Golestan	AqQala	54.779730	37.180390
Literature	231	Iran	Golestan	Gonbad-e Kavus	54.998010	37.255960
Literature	231	Iran	Golestan	Maraveh Tappeh	55.402270	37.682210
Literature	231	Iran	Golestan	Gomishan	54.515020	37.209740
Literature	231	Iran	Golestan	Gomishan	54.472840	37.108740
Literature	231	Iran	Golestan	Gomishan	54.418490	37.137680
Literature	231	Iran	Golestan	Aq Qala	54.859460	37.133170
Literature	231	Iran	Golestan	Aliabad	54.842840	36.781840
Literature	231	Iran	Golestan	Aliabad	54.942270	36.809290
Literature	231	Iran	Golestan	Gorgan	54.688710	36.904850
Literature	231	Iran	Golestan	Gorgan	54.470000	36.890250
Literature	231	Iran	Golestan	Kordkuy	54.312310	36.801460
Literature	234	Iran	Kurdistan	Bijar	47.583300	35.700000
Literature	234	Iran	Kurdistan	Bijar	47.550000	36.016600
Literature	234	Iran	Kurdistan	Qorveh	47.973300	35.292200
Literature	234	Iran	Kurdistan	Qorveh	47.795100	35.159400
Literature	234	Iran	Kurdistan	Sanandaj	47.010600	35.235500
Literature	234	Iran	Kurdistan	Saqez	46.766600	36.366600
Literature	262	Pakistan	Khyber Pakhtunkhwa	Hazara Division	73.419400	34.556900
Literature	262	Pakistan	Khyber Pakhtunkhwa	Malakand Division	72.292800	34.782900

Literature	262	Pakistan	Khyber Pakhtunkhwa	Mardan Division	71.944100	34.281600
Literature	262	Pakistan	Khyber Pakhtunkhwa	Peshawar Division	71.532500	34.045200
Literature	262	Pakistan	Khyber Pakhtunkhwa	Peshawar Division	71.651500	33.998400
Literature	270	Lebanon	Nabatieh	Marjiiyoun	35.500000	33.500000
GBIF	1	Pakistan	Punjab	Lahore	73.860000	31.220000
GBIF	2	Pakistan	Punjab	Gujranwala	72.730000	30.450000
GBIF	3	Pakistan	Balochistan	Kalat	64.100000	26.970000
GBIF	4	Pakistan	Balochistan	Quetta	67.190000	30.270000
GBIF	5	Pakistan	Punjab	Multan	70.800000	30.500000
GBIF	6	Pakistan	Balochistan	Quetta	68.600000	30.370000
GBIF	7	Pakistan	Sindh	Karachi	67.580000	24.470000
GBIF	8	Pakistan	Balochistan	Quetta	68.540000	30.400000
GBIF	9	Pakistan	Punjab	Lahore	74.130000	32.200000
GBIF	10	Pakistan	Sindh	Karachi	67.050000	24.870000
GBIF	11	Pakistan	Punjab	Bahawalpur	67.720000	26.820000
GBIF	12	Pakistan	Punjab	Lahore	74.340000	31.550000
GBIF	13	Pakistan	Punjab	Lahore	74.400000	31.360000
GBIF	14	Pakistan	Punjab	Mahmood Boati	68.783300	30.400000
GBIF	15	Pakistan	Punjab	Lahore	74.410000	31.650000
GBIF	16	Pakistan	Khyber-Pakhtunkwa	Mardan	72.030000	34.200000
GBIF	17	Pakistan	Punjab	Lahore	68.830000	29.500000
GBIF	18	Pakistan	Khyber-Pakhtunkwa	Malakand	71.930000	34.560000
GBIF	20	Pakistan	Khyber-Pakhtunkwa	Shah Mansur	72.450000	34.070000
GBIF	21	Pakistan	Punjab	Rawalpindi	74.100000	33.900000
GBIF	22	Pakistan	Punjab	Lahore	73.877800	31.190300
GBIF	23	Pakistan	Punjab	Lahore	74.480000	31.590000
GBIF	24	Pakistan	Punjab	Lahore	74.500000	31.510000
GBIF	25	Pakistan	Punjab	Lahore	73.760000	30.970000
GBIF	26	Pakistan	Punjab	Rawalpindi	73.520000	33.650000
GBIF	27	Pakistan	Punjab	Rawalpindi	73.500000	33.450000
GBIF	28	Pakistan	Punjab	Rawalpindi	73.070000	33.600000
GBIF	29	Pakistan	Sindh	Hyderabad	69.730000	25.360000
GBIF	30	Pakistan	Sindh	Hyderabad	70.110000	25.410000
GBIF	31	Pakistan	Punjab	Lahore	74.490000	31.550000
GBIF	32	Pakistan	Sindh	Hyderabad	69.730000	25.370000
GBIF	33	Pakistan	Punjab	Karpa	72.420000	34.710000
GBIF	34	Pakistan	Khyber-Pakhtunkwa	Hazara	73.200000	34.330000
GBIF	35	Pakistan	Punjab	Lahore	75.200000	32.200000
GBIF	36	Pakistan	Punjab	Lahore	74.570000	31.500000
GBIF	37	Pakistan	Punjab	Lahore	73.980000	31.710000
GBIF	38	Pakistan	Punjab	Lahore	74.570000	31.570000
GBIF	39	Pakistan	Punjab	Lahore	73.300000	33.270000

GBIF	40	Pakistan	Punjab	Lahore	74.510000	31.480000
GBIF	42	Pakistan	Sindh	Hyderabad	70.200000	25.700000
GBIF	43	Pakistan	Punjab	Faisalabad	73.130000	31.030000
GBIF	44	Pakistan	Punjab	Lahore	72.160000	31.850000
GBIF	45	Pakistan	Punjab	Sargodha	67.800000	24.490000
GBIF	46	Pakistan	Punjab	Sargodha	73.420000	31.330000
GBIF	47	Pakistan	Sindh	Hyderabad	70.230000	25.470000
GBIF	49	Pakistan	Punjab	Lahore	72.150000	31.330000
GBIF	50	Pakistan	Punjab	Lahore	74.370000	31.640000
GBIF	51	Pakistan	Punjab	Lahore	74.530000	31.590000
GBIF	52	Pakistan	Punjab	Lahore	74.360000	31.390000
GBIF	53	Pakistan	Punjab	Faisalabad	73.080000	31.420000
GBIF	54	Pakistan	Punjab	Lahore	74.510000	31.910000
GBIF	55	Pakistan	Punjab	Lahore	73.970000	31.080000
GBIF	56	Pakistan	Punjab	Lahore	74.260000	31.760000
GBIF	57	Pakistan	Punjab	Gujranwala	74.170000	32.170000
GBIF	58	Pakistan	Punjab	Lahore	72.550000	33.170000
GBIF	59	Pakistan	Punjab	Lahore	73.830000	30.970000
GBIF	60	Pakistan	Punjab	Lahore	74.100000	31.460000
GBIF	61	Pakistan	Punjab	Gujranwala	73.270000	31.900000
GBIF	63	Pakistan	Punjab	Lahore	73.820000	32.780000
GBIF	64	Pakistan	Punjab	Lahore	73.420000	32.120000
GBIF	65	Pakistan	Punjab	Lahore	74.230000	31.620000
GBIF	66	Pakistan	Punjab	Aalus Kalan	74.420000	32.380000
GBIF	69	Pakistan	Punjab	Hafizabad	72.810000	32.420000
GBIF	70	Pakistan	Punjab	Lahore	73.980000	32.820000
GBIF	71	Pakistan	Punjab	Lahore	74.400000	31.060000
GBIF	72	Pakistan	Punjab	Lahore	74.430000	31.310000
GBIF	74	Pakistan	Punjab	Lahore	74.500000	31.440000
GBIF	75	Pakistan	Punjab	Lahore	74.340000	31.470000
GBIF	76	Pakistan	Punjab	Alipur	70.060000	29.380000
GBIF	77	Pakistan	Punjab	Lahore	74.410000	31.560000
GBIF	78	Pakistan	Punjab	Lahore	74.330000	31.430000
GBIF	79	Pakistan	Sindh	Hyderabad	69.120000	27.850000
GBIF	80	Pakistan	Punjab	Lahore	70.430000	29.300000
GBIF	81	Pakistan	Punjab	Lahore	74.620000	32.560000
GBIF	82	Pakistan	Punjab	Lahore	74.590000	31.420000
GBIF	83	Pakistan	Punjab	Lahore	74.170000	32.210000
GBIF	85	Pakistan	Punjab	Lahore	74.060000	31.120000
GBIF	86	Pakistan	Punjab	Lahore	74.030000	30.600000
GBIF	87	Pakistan	Punjab	Lahore	73.820000	30.930000
GBIF	88	Pakistan	Punjab	Lahore	75.170000	32.270000

GBIF	90	Pakistan	Punjab	Lahore	74.320000	30.930000
GBIF	91	Pakistan	Punjab	Lahore	74.560000	32.520000
GBIF	92	Pakistan	Punjab	Wazirabad	74.120000	32.450000
GBIF	93	Pakistan	Punjab	Lahore	74.410000	31.280000
GBIF	95	Pakistan	Punjab	Lahore	73.480000	30.470000
GBIF	97	Pakistan	Punjab	Lahore	74.370000	31.430000
GBIF	98	Pakistan	Punjab	Lahore	73.290000	30.950000
GBIF	100	Pakistan	Punjab	Lahore	74.600000	32.420000
GBIF	101	Pakistan	Punjab	Dial	74.530000	30.600000
GBIF	102	Pakistan	Punjab	Sargodha	71.750000	32.610000
GBIF	103	Pakistan	Sindh	Karachi	66.850000	24.990000
GBIF	104	Pakistan	Punjab	Rawalpindi	73.730000	32.930000
GBIF	105	Pakistan	Punjab	Rawalpindi	73.050000	32.580000
GBIF	106	Pakistan	Punjab	Sargodha	72.830000	33.740000
GBIF	107	Pakistan	Punjab	Rawalpindi	74.250000	31.220000
GBIF	108	Pakistan	Sindh	Sukkur	69.320000	28.020000
GBIF	109	Pakistan	Sindh	Hyderabad	68.750000	25.250000
GBIF	110	Pakistan	Sindh	Hyderabad	69.010000	25.520000
GBIF	111	Pakistan	Punjab	Lahore	74.130000	30.770000
GBIF	112	Pakistan	Punjab	Lahore	74.420000	31.580000
GBIF	113	Pakistan	Sindh	Sukkur	69.730000	28.160000
GBIF	114	Pakistan	Punjab	Lahore	74.500000	31.170000
GBIF	115	Pakistan	Punjab	Lahore	74.410000	31.090000
GBIF	116	Pakistan	Sindh	Hyderabad	67.920000	24.750000
GBIF	117	Pakistan	Punjab	Multan	71.020000	29.350000
GBIF	118	Pakistan	Sindh	Hyderabad	68.650000	25.030000
GBIF	119	Pakistan	Khyber-Pakhtunkwa	Dera Ismail Khan	70.900000	31.830000
GBIF	120	Pakistan	Balochistan	Uthal	66.620000	25.810000
GBIF	121	Pakistan	Azad Kashmir	Dhok	72.680000	33.920000
GBIF	122	Pakistan	Punjab	Nankana Sahib	73.700000	31.450000
GBIF	123	Pakistan	Punjab	Sialkot	71.850000	30.920000
GBIF	124	Pakistan	Azad Kashmir	Muzaffarabad	73.470000	34.370000
GBIF	125	Pakistan	Punjab	Multan	69.740000	28.530000
GBIF	126	Pakistan	Punjab	Bahawalpur	69.920000	28.420000
GBIF	127	Pakistan	Northern Areas	Gilgit	74.290000	35.920000
GBIF	128	Pakistan	Sindh	Hyderabad	69.250000	25.290000
GBIF	129	Pakistan	Punjab	Bahawalpur	72.850000	29.190000
GBIF	130	Pakistan	Balochistan	Kalat	64.140000	26.980000
GBIF	131	Pakistan	Balochistan	Kalat	63.910000	26.020000
GBIF	132	Pakistan	Sindh	Hyderabad	70.750000	24.370000
GBIF	133	Pakistan	Punjab	Lahore	74.324400	31.484400
GBIF	134	Pakistan	Balochistan	Quetta	69.900000	30.050000

GBIF	135	Pakistan	Balochistan	Kalat	66.850000	29.800000
GBIF	136	Pakistan	Balochistan	Kalat	66.260000	28.490000
GBIF	137	Pakistan	Punjab	Multan	71.200000	30.070000
GBIF	138	Pakistan	Punjab	Lahore	74.870000	32.480000
GBIF	139	Pakistan	Khyber-Pakhtunkwa	Peshawar	71.560000	34.000000
GBIF	140	Pakistan	Khyber-Pakhtunkwa	Malakand	71.800000	35.880000
GBIF	141	Pakistan	Balochistan	Quetta	69.450000	31.340000
GBIF	142	Pakistan	Khyber-Pakhtunkwa	Peshawar	71.990000	34.620000
GBIF	143	Pakistan	Khyber-Pakhtunkwa	Malakand	72.350000	34.750000
GBIF	144	Pakistan	Islamabad	Islamabad	73.170000	33.700000
GBIF	145	Pakistan	Sindh	Sukkur	68.420000	26.250000
GBIF	146	Pakistan	Balochistan	Zhob	68.030000	30.880000
GBIF	147	Pakistan	Balochistan	Quetta	66.583900	30.607200
GBIF	148	Pakistan	Punjab	Bahawalpur	72.550000	29.820000
GBIF	149	Pakistan	Azad Kashmir	Kohala	73.480000	34.120000
GBIF	150	Pakistan	Balochistan	Quetta	66.450000	30.930000
GBIF	151	Pakistan	Sindh	Karachi	67.110000	24.920000
GBIF	152	Pakistan	Sindh	Karachi	66.300000	26.230000
GBIF	153	Pakistan	Balochistan	Quetta	66.020000	29.550000
GBIF	154	Pakistan	Balochistan	Karachi	66.600000	25.420000
GBIF	155	Pakistan	Khyber-Pakhtunkwa	Peshawar	72.610000	34.890000
GBIF	156	Pakistan	Khyber-Pakhtunkwa	Peshawar	72.940000	34.000000
GBIF	157	Pakistan	Balochistan	Quetta	67.730000	30.380000
GBIF	158	Pakistan	Punjab	Bahawalpur	71.250000	29.150000
GBIF	159	Pakistan	Gilgit-Baltistan	Chilas	74.080000	35.430000
GBIF	160	Pakistan	Balochistan	Karachi	68.000000	28.000000
GBIF	161	Pakistan	Punjab	Rawalpindi	73.040000	33.610000
GBIF	162	Pakistan	Balochistan	Quetta	66.580000	30.610000
GBIF	163	Pakistan	Sindh	Karachi	67.190000	24.790000
GBIF	164	Pakistan	Balochistan	Quetta	69.820000	30.450000
GBIF	165	Pakistan	Sindh	Sukkur	69.240000	24.880000
GBIF	166	Pakistan	Balochistan	Kalat	70.960000	30.820000
GBIF	167	Pakistan	Khyber-Pakhtunkwa	Malakand	72.580000	35.530000
GBIF	168	Pakistan	Federally Administered Tribal Areas	Khyber Pass	71.150000	34.090000
GBIF	169	Pakistan	Khyber-Pakhtunkwa	Peshawar	71.420000	34.160000
GBIF	170	Pakistan	Punjab	Lahore	73.830000	31.190000
GBIF	171	Pakistan	Punjab	Phagwari	73.500000	33.980000
GBIF	172	Pakistan	Punjab	Rawalpindi	73.400000	33.900000
GBIF	173	Pakistan	Punjab	Lahore	70.940000	30.880000
GBIF	174	Pakistan	Punjab	Lahore	73.970000	31.270000
GBIF	175	Pakistan	Punjab	Lahore	72.920000	33.370000

GBIF	176	Pakistan	Punjab	Lahore	73.450000	32.050000
GBIF	177	Pakistan	Punjab	Lahore	74.080000	32.650000
GBIF	178	Pakistan	Punjab	Sargodha	71.566700	32.966700
GBIF	179	Pakistan	Balochistan	Kalat	66.430000	28.030000
GBIF	180	Pakistan	Punjab	Bahawalpur	72.080000	29.110000
GBIF	181	Pakistan	Punjab	Lahore	74.080000	32.710000
GBIF	182	Pakistan	Balochistan	Quetta	67.000000	30.200000
GBIF	183	Pakistan	Sindh	Sukkur	68.440000	28.280000
GBIF	184	Pakistan	Punjab	Lahore	74.210000	31.920000
GBIF	185	Pakistan	Punjab	Sargodha	71.440000	32.890000
GBIF	186	Pakistan	Punjab	Lahore	74.300000	31.400000
GBIF	187	Pakistan	Punjab	Bahawalpur	71.270000	29.150000
GBIF	188	Pakistan	Punjab	Lahore	74.360000	31.520000
GBIF	189	Pakistan	Sindh	Hyderabad	67.650000	26.420000
GBIF	190	Pakistan	Balochistan	Quetta	69.180000	30.990000
GBIF	191	Pakistan	Punjab	Multan	73.700000	30.450000
GBIF	192	Pakistan	Punjab	Bahawalpur	71.330000	28.770000
GBIF	195	Iraq	Al-Qādisiyah	Al-Sanniya	44.000000	32.000000
GBIF	196	Iraq	Dhi Qar	Fahod	46.000000	30.000000
GBIF	197	Iraq	Al-Muthanna	Rumaitha	45.000000	31.000000
GBIF	198	Iraq	Maysan	Castle Saleh	47.000000	31.000000
GBIF	199	Morocco	Marrakech-Safi	de Safi	-9.233333	32.283333
GBIF	201	France	Occitanie	Pyrenees-Orientales	3.023950	42.478160
GBIF	203	USA	Maryland	Prince George's	-76.800000	39.050000

*The geographic locations presented are all verified sites with precise coordinates, sourced from the literature review and the Global Biodiversity Information Facility (GBIF).

Text S3: Predictive modeling of the potential distribution of H. anatolicum

Predictive Modeling: We utilized MaxEnt (version 3.4.4) to predict the global habitat suitability of *Hyalomma anatolicum* [1–2]. Only occurrence records with precise geographic coordinates were retained, while those derived from centroids (which introduce spatial ambiguity) were excluded. To mitigate sampling bias and avoid model overfitting, spatial rarefaction was performed using ENMTools (version 1.4.4) [3].

Variable Selection: A rigorous screening process was implemented to eliminate multicollinearity among environmental variables. Initially, the Jackknife test was used to evaluate the initial contribution of each variable, followed by Pearson correlation analysis conducted via ENMTools [3]. For highly correlated variable pairs ($|r| > 0.8$), we retained variables with higher predictive power and biological relevance to H. anatolicum, while removing those with negligible contribution (< 0.5%).

Optimization & Validation: Model hyperparameters were optimized using the kuenm package in R software by testing all combinations of regularization multipliers and feature classes (linear = L, quadratic = Q, product = P, threshold = T, and hinge = H). The model with the lowest Akaike Information Criterion corrected for small sample sizes (AICc = 0) was selected to balance model complexity and goodness-of-fit [4]. The final model was run with 10,000 iterations and 25-fold cross-validation; model performance was evaluated using the Area Under the Receiver Operating Characteristic Curve (AUC), and the relative importance of each variable was quantified via the Jackknife test [5].

[1] Phillips SJ, Anderson RP, Schapire RE, Maximum entropy modeling of species geographic distributions, Ecological Modelling. 190(2006) 231-259, https://doi.org/10.1016/j.ecolmodel.2005.03.026.

[2] Elith J, Phillips SJ, Hastie T, Dudík M, Chee YE, Yates CJ, A statistical explanation

of MaxEnt for ecologists, Diversity and Distributions. 17(2011) 43-57, https://doi.org/10.1111/j.1472-4642.2010.00725.x.

[3] Warren DL, Glor RE, Turelli M, ENMTools: A toolbox for comparative studies of environmental niche models, Ecography. 33(2010) 607-611, https:// doi.org/10.1111/j.1600-0587.2009.06142.x.

[4] Rochlin I, Modeling the Asian Longhorned Tick (Acari: Ixodidae) Suitable Habitat in North America, Journal of Medical Entomology. 56(2018) 2, https://doi.org/10.1093/jme/tjy210.

[5] Phillips SJ, Anderson RP, Schapire RE, Maximum entropy modeling of species geographic distributions, Ecological Modelling. 190(2006)231-259, https://doi.org/10.1016/j.ecolmodel.2005.03.026.

Text S4: Data Collection Characteristics

Data Sources and Systematic Review Process

Data for this study were compiled from three primary sources: (1) a systematic literature review; (2) the specialized reference book, "Fauna Sinica-Arachnida Ixodida," from which we extracted authoritative information on the geographic distribution of *H. anatolicum*; and (3) the related website, the Global Biodiversity Information Facility (GBIF).

Our systematic literature search identified 2,728 potentially relevant studies. After 711 duplicate records were removed, the titles and abstracts of the remaining 2,017 studies were screened. Following a full-text review against our inclusion and exclusion criteria, 362 articles were ultimately included in the study. An additional 203 valid coordinate records for *H. anatolicum* were obtained from GBIF.

Geographic Distribution Data

Data integration, cleaning, and deduplication yielded 591 unique geographic records spanning 32 countries. The breakdown of records was as follows: Pakistan (n=250), Iran (n=93), China (n=88), India (n=50), Turkey (n=24), Sudan (n=15), Iraq (n=11), Egypt (n=7), Saudi Arabia (n=7), Kazakhstan (n=6), United Arab Emirates (n=4), Algeria (n=4), and Morocco (n=3), Lebanon (n=3), Tajikistan (n=3), Bangladesh (n=3). The remaining 16 countries (Oman, the United States, France, Israel, Nepal, Turkmenistan, Uzbekistan, Russia, Afghanistan, Bulgaria, Cyprus, Ethiopia, Greece, Somalia, Yemen, and Kyrgyzstan) contributed two or fewer records each.

Host Data

A total of 318 host records were obtained, comprising 19 distinct host species across 7 families The most frequently recorded families were Bovidae (n=245), followed by Camelidae (n=41), Equidae (n=21), Canidae (n=7), Hominidae (n=2), Suidae (n=1), and Leporidae (n=1). At the country level, Pakistan documented the highest host species diversity (11 species), followed by India (8 species), China (8 species), Iran (6 species), and Sudan (6 species).

Pathogen Spectrum Data

Pathogens in *H. anatolicum*: A total of 66 distinct microbial species were identified in *H. anatolicum* samples, categorized as 21 human pathogens, 26 animal pathogens, and 19 microbes of unknown pathogenicity. Sufficient quantitative data (i.e., total sample size and number of positives) were available for 33 of these species to be included in the meta-analysis.

Pathogens in hosts: A total of 36 distinct microbial species were identified within the hosts of *H. anatolicum*. These comprised 15 human pathogens, 13 animal pathogens, and 8 microbes with unknown pathogenicity. For 17 of these microbes, sufficient quantitative data were available to conduct a meta-analysis.

Figure S3: Geographic distribution of *Hyalomma anatolicum* in 32 countries

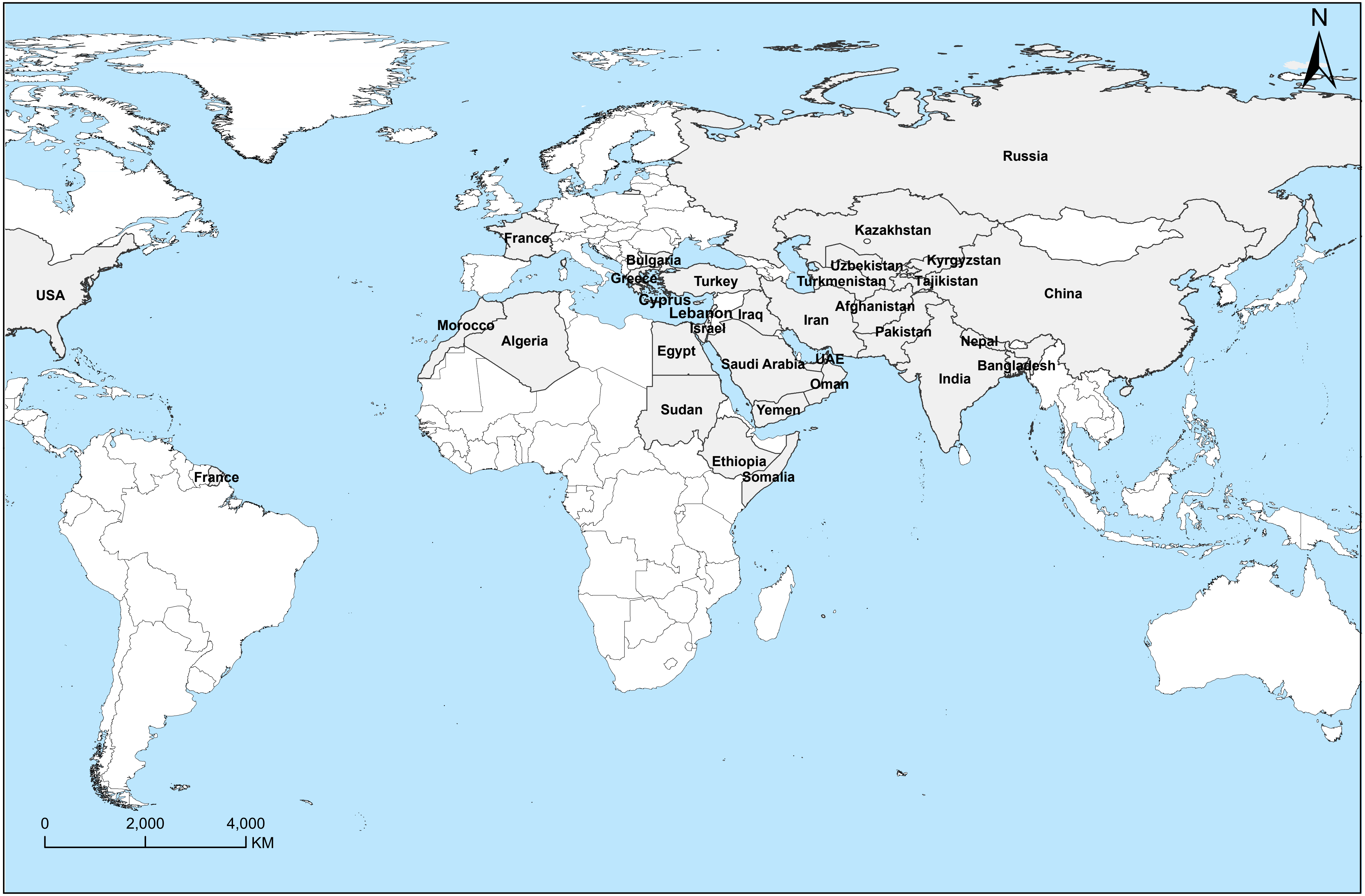

Figure S4: The distribution of *H. anatolicum* in Pakistan and China

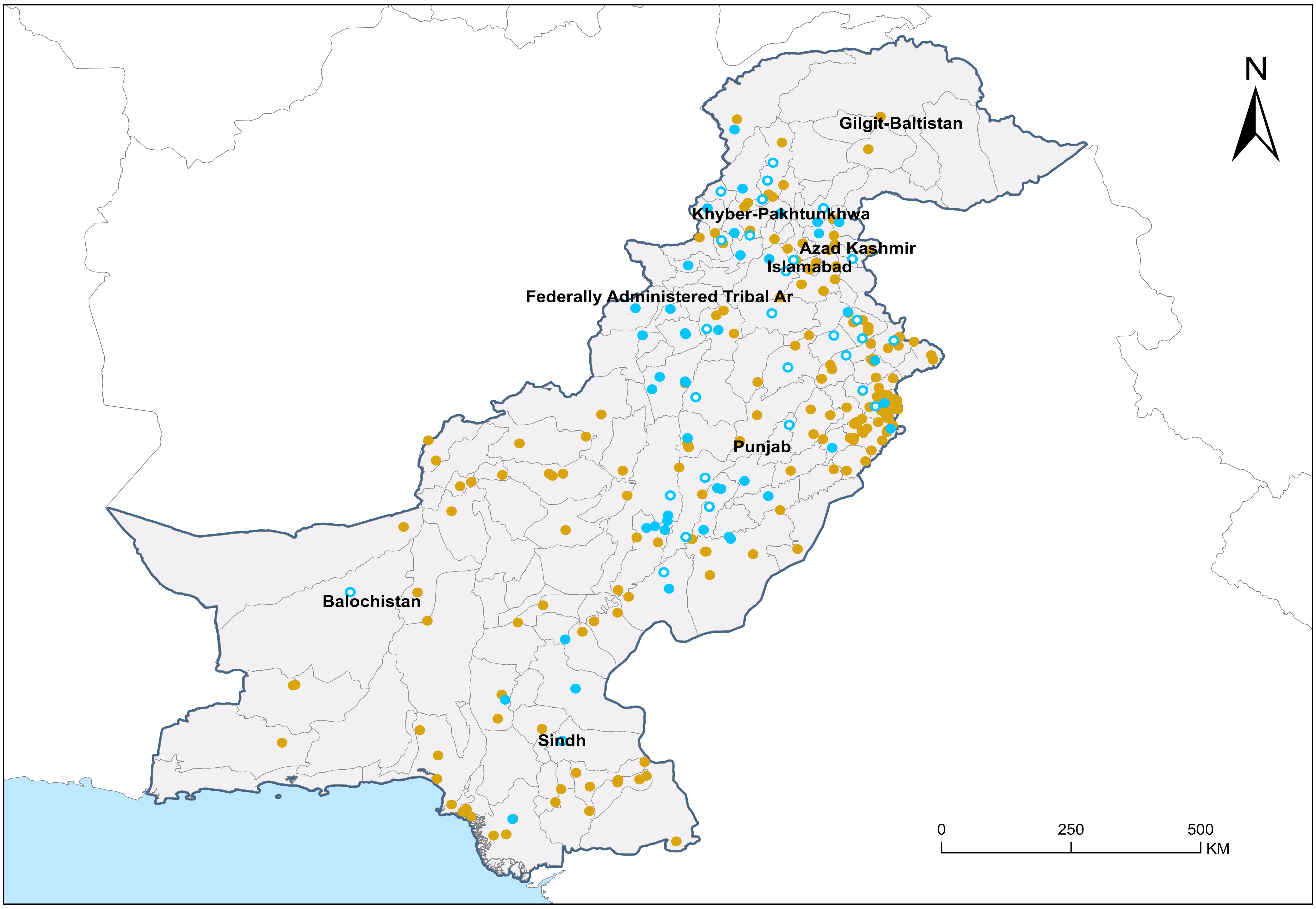

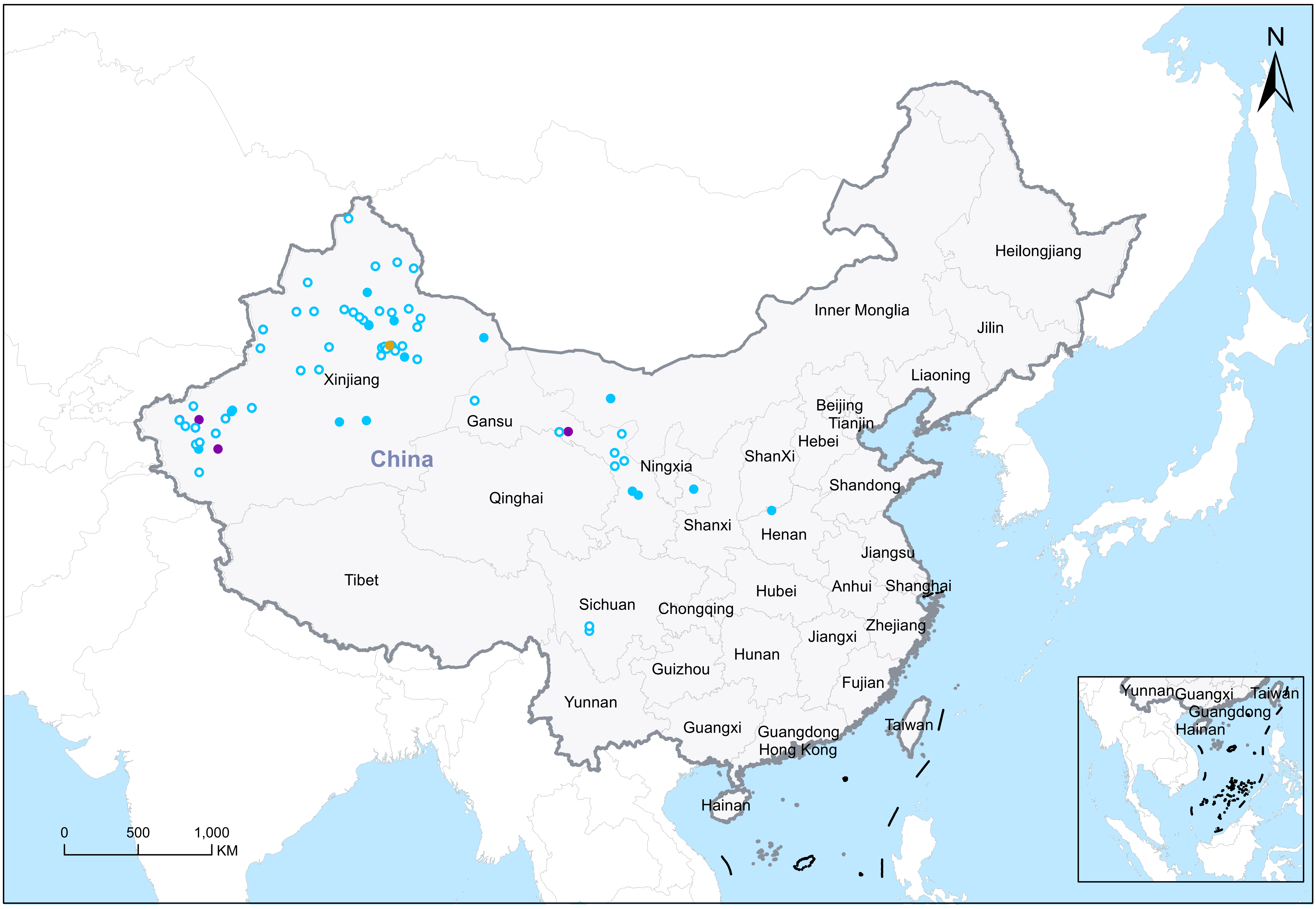

Blue open circles denote precise coordinates or third-level administrative centroids, while blue filled circles represent centroids at the second level or higher. Purple symbols distinguish reference book at the third-level (open) and higher-level (filled) administrative divisions. Yellow filled circles indicate records from GBIF.

Figure S5: Prevalence of *H. anatolicum*-associated microbes

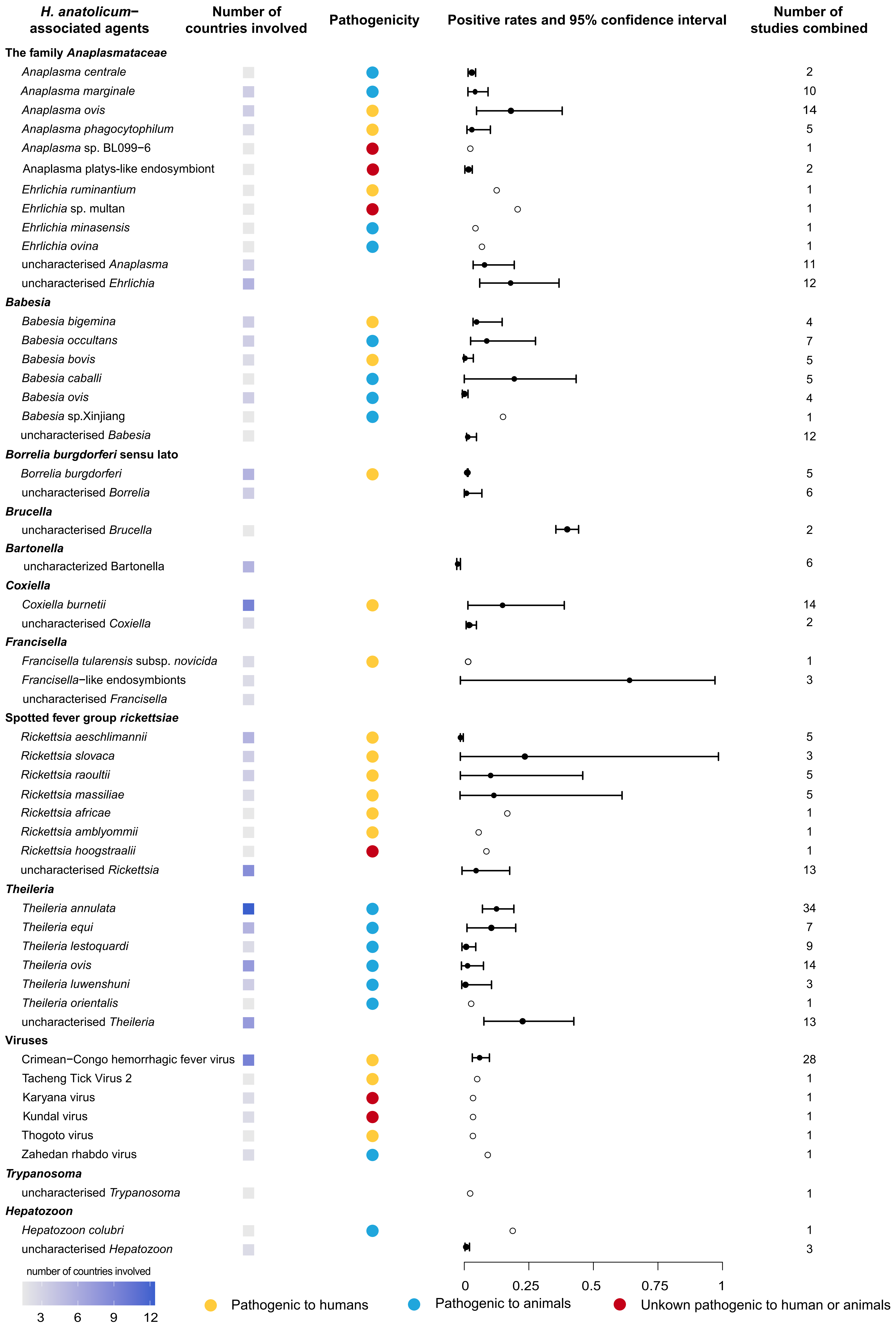

Figure S6: Meta-analysis of the prevalence of each *H. anatolicum*-associated microbes

The heterogeneity of combined studies was quantified by I^2 statistic. The fixed effect model would be applied if I^2<50%; Otherwise, the random effect model would be applied.

Anaplasma centrale

Study	Events	Total	Proportion	95%−CI	Weight (common)	Weight (random)
Ghafar et al. (2020)	6	212	0.028302	[0.010456; 0.060580]	42.8%	42.8%
Rehman et al. (2019)	9	300	0.030000	[0.013808; 0.056184]	57.2%	57.2%
**Common effect model**		**512**	**0.029273**	**[0.014672; 0.043875]**	**100.0%**	**.**
**Random effects model**			**0.029273**	**[0.014672; 0.043875]**	**.**	**100.0%**

Heterogeneity: I^2 = 0.0%, τ^2 = 0, p = 0.910207

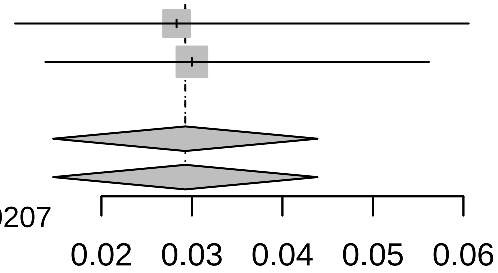

Anaplasma marginale

Study	Events	Total	Proportion	95%−CI	Weight (common)	Weight (random)
Adegoke et al. (2020)	5	198	0.025253	[0.008249; 0.057944]	16.0%	12.6%
Alam et al. (2022)	10	42	0.238095	[0.120516; 0.394502]	3.4%	10.8%
Ali et al. (2023)	0	8	0.000000	[0.000000; 0.369417]	0.6%	6.0%
Choubdar et al. (2021)	0	114	0.000000	[0.000000; 0.031841]	9.2%	12.2%
Mossaad et al. (2021)	28	239	0.117155	[0.079277; 0.164855]	19.3%	12.7%
Ghafar et al. (2020)	14	212	0.066038	[0.036571; 0.108319]	17.1%	12.6%
Loftis et al. (2006)	0	10	0.000000	[0.000000; 0.308497]	0.8%	6.8%
Choubdar et al. (2021)	0	114	0.000000	[0.000000; 0.031841]	9.2%	12.2%
Rehman et al. (2019)	8	300	0.026667	[0.011582; 0.051866]	24.2%	12.8%
Sadeddine et al. (2019)	0	1	0.000000	[0.000000; 0.975000]	0.1%	1.3%
**Common effect model**		**1238**	**0.037361**	**[0.027520; 0.048637]**	**100.0%**	**.**
**Random effects model**			**0.029727**	**[0.003318; 0.081183]**	**.**	**100.0%**

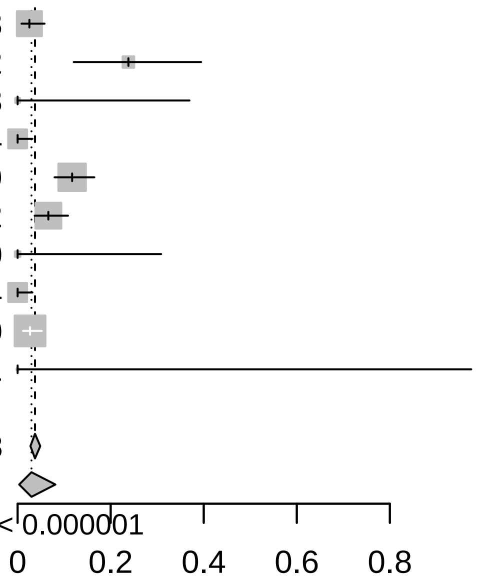

Heterogeneity: $I^2 = 89.1\%$, $\tau^2 = 0.0264$, $p < 0.000001$

Anaplasma ovis

Study	Events	Total	Proportion	95%−CI	Weight (common)	Weight (random)
Ghafar et al. (2020)	6	6	1.000000	[0.540742; 1.000000]	0.3%	6.4%
Choubdar et al. (2021)	78	114	0.684211	[0.590510; 0.768067]	5.0%	8.0%
Ghafar et al. (2020)	4	212	0.018868	[0.005164; 0.047602]	9.2%	8.0%
Li et al. (2020)	73	240	0.304167	[0.246625; 0.366621]	10.5%	8.0%
Rehman et al. (2019)	3	300	0.010000	[0.002067; 0.028945]	13.1%	8.0%
Sadeddine et al. (2019)	0	1	0.000000	[0.000000; 0.975000]	0.0%	3.2%
Yu et al. (2017)	1	20	0.050000	[0.001265; 0.248733]	0.9%	7.5%
Jiang et al. (2020)	66	680	0.097059	[0.075861; 0.121826]	29.6%	8.0%
Liu et al. (2023)	0	3	0.000000	[0.000000; 0.707598]	0.1%	5.4%
Wen et al. (2019)	73	250	0.292000	[0.236407; 0.352602]	10.9%	8.0%
Yu et al. (2019)	74	322	0.229814	[0.184981; 0.279694]	14.0%	8.0%
K?seo?lu AE et al. (2021)	0	4	0.000000	[0.000000; 0.602365]	0.2%	5.8%
Aktas et al. (2009)	0	118	0.000000	[0.000000; 0.030778]	5.1%	8.0%
Tajedin et al. (2016)	12	25	0.480000	[0.277968; 0.686943]	1.1%	7.6%
**Common effect model**		**2295**	**0.134778**	**[0.121116; 0.149051]**	**100.0%**	**.**
**Random effects model**			**0.174539**	**[0.041401; 0.373099]**	**.**	**100.0%**

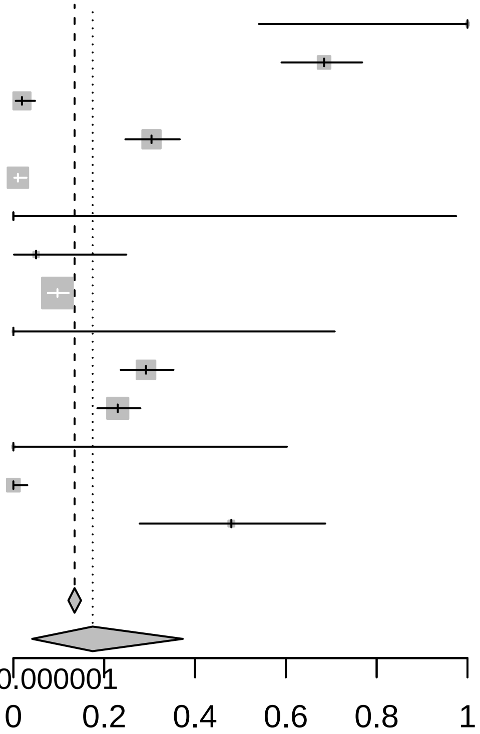

Heterogeneity: $I^2 = 97.6\%$, $\tau^2 = 0.1650$, $p < 0.000001$

Anaplasma phagocytophilum

Study	Events	Total	Proportion	95%−CI	Weight (common)	Weight (random)
Ghafar et al. (2020)	1	212	0.004717	[0.000119; 0.026000]	62.2%	37.6%
Polat et al. (2020)	0	5	0.000000	[0.000000; 0.521824]	1.5%	11.3%
Liu et al. (2023)	0	3	0.000000	[0.000000; 0.707598]	0.9%	7.6%
Wu et al. (2021)	12	118	0.101695	[0.053661; 0.170903]	34.6%	35.9%
Nader et al. (2018)	0	3	0.000000	[0.000000; 0.707598]	0.9%	7.6%
**Common effect model**		**341**	**0.023847**	**[0.010363; 0.042690]**	**100.0%**	**.**
**Random effects model**			**0.020156**	**[0.000000; 0.096686]**	**.**	**100.0%**

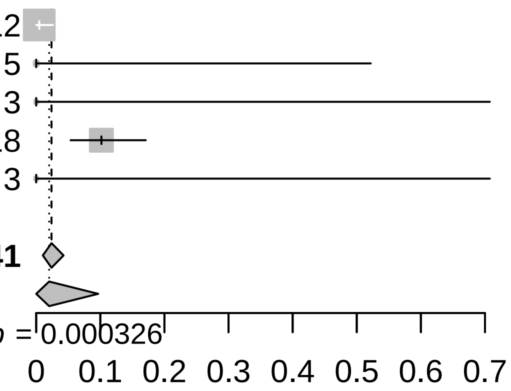

Heterogeneity: I^2 = 80.9%, τ^2 = 0.0197, p = 0.000326

uncharacterised *Anaplasma*

Study	Events	Total	Proportion	95%−CI	Weight (common)	Weight (random)
JafarBekloo et al. (2018)	6	73	0.082192	[0.030762; 0.170361]	4.5%	11.4%
Bekloo et al. (2017)	2	5	0.400000	[0.052745; 0.853367]	0.3%	7.5%
Khan et al. (2022)	0	136	0.000000	[0.000000; 0.026760]	8.4%	11.6%
Aktas et al. (2009)	0	118	0.000000	[0.000000; 0.030778]	7.3%	11.5%
Zhao et al. (2020)	0	853	0.000000	[0.000000; 0.004315]	52.8%	11.8%
Xiao et al. (2022)	14	24	0.583333	[0.366431; 0.778903]	1.5%	10.6%
K?seo?lu et al. (2021)	0	4	0.000000	[0.000000; 0.602365]	0.2%	6.9%
Orkun et al. (2022)	0	4	0.000000	[0.000000; 0.602365]	0.2%	6.9%
Ranjbar et al. (2020)	2	17	0.117647	[0.014579; 0.364409]	1.1%	10.1%
Kartashov et al. (2020)	0	382	0.000000	[0.000000; 0.009610]	23.6%	11.7%
**Common effect model**		**1616**	**0.001014**	**[0.000056; 0.003158]**	**100.0%**	**.**
**Random effects model**			**0.044178**	**[0.000166; 0.159362]**	**.**	**100.0%**

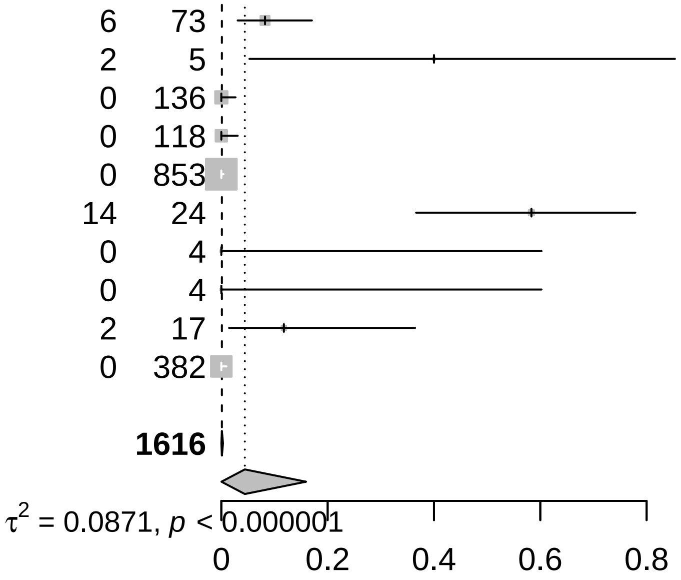

Heterogeneity: I^2 = 91.7%, τ^2 = 0.0871, p < 0.000001

uncharacterised *Ehrlichia*

Study	Events	Total	Proportion	95%−CI	Weight (common)	Weight (random)
Ghafar et al. (2020)	6	6	1.000000	[0.540742; 1.000000]	0.6%	7.3%
JafarBekloo et al. (2018)	6	73	0.082192	[0.030762; 0.170361]	7.5%	9.3%
Aktas et al. (2009)	3	118	0.025424	[0.005274; 0.072502]	12.1%	9.3%
Bekloo et al. (2017)	2	5	0.400000	[0.052745; 0.853367]	0.5%	6.9%
Ghafar et al. (2020)	47	212	0.221698	[0.167677; 0.283677]	21.8%	9.4%
Kartashov et al. (2020)	5	382	0.013089	[0.004263; 0.030280]	39.3%	9.4%
Loftis et al. (2006)	0	10	0.000000	[0.000000; 0.308497]	1.0%	8.0%
Aktas et al. (2009)	3	118	0.025424	[0.005274; 0.072502]	12.1%	9.3%
Yu et al. (2017)	3	20	0.150000	[0.032071; 0.378927]	2.1%	8.7%
Orkun ? et al. (2022)	0	4	0.000000	[0.000000; 0.602365]	0.4%	6.5%
Tajedin L et al. (2016)	7	18	0.388889	[0.172986; 0.642549]	1.9%	8.6%
Atefeh et al. (2013)	1	6	0.166667	[0.004211; 0.641235]	0.6%	7.3%
**Common effect model**		**972**	**0.060813**	**[0.046666; 0.076695]**	**100.0%**	**.**
**Random effects model**			**0.150289**	**[0.030653; 0.337811]**	**.**	**100.0%**

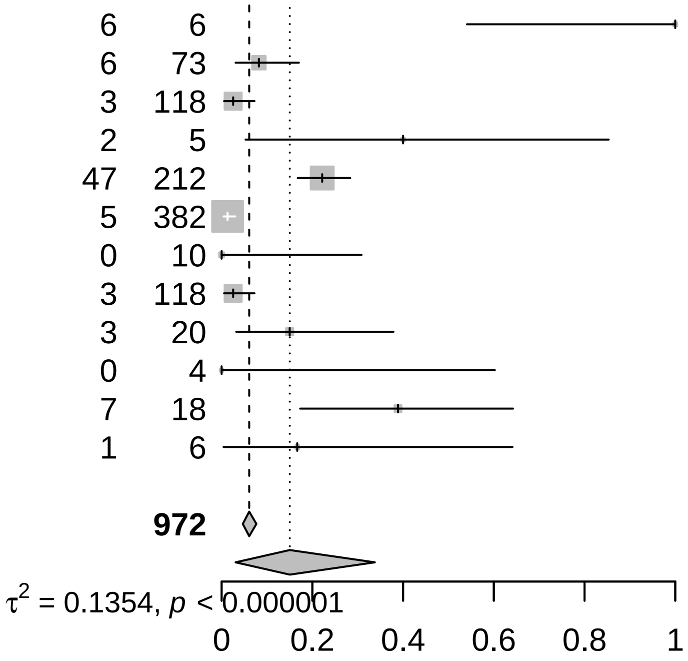

Heterogeneity: $I^2 = 92.6\%$, $\tau^2 = 0.1354$, $p < 0.000001$

Babesia bigemina

Study	Events	Total	Proportion	95%−CI	Weight (common)	Weight (random)
Abdallah et al. (2017)	0	20	0.000000	[0.000000; 0.168433]	3.0%	21.5%
Mossaad et al. (2021)	44	239	0.184100	[0.137070; 0.239154]	36.0%	26.4%
Rajabi et al. (2017)	0	105	0.000000	[0.000000; 0.034522]	15.8%	25.7%
Rehman et al. (2019)	0	300	0.000000	[0.000000; 0.012221]	45.2%	26.5%
**Common effect model**		**664**	**0.025263**	**[0.014711; 0.038560]**	**100.0%**	**.**
**Random effects model**			**0.013610**	**[0.000000; 0.112734]**	**.**	**100.0%**

Heterogeneity: $I^2 = 97.5\%$, $\tau^2 = 0.0491$, $p < 0.000001$

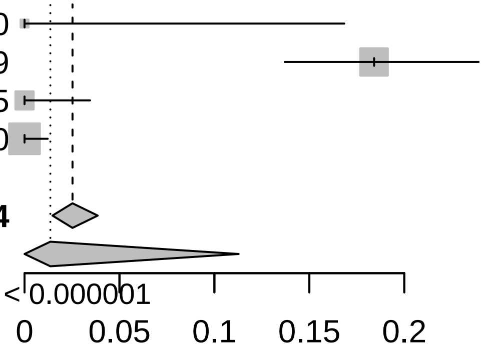

Babesia occultans

Study	Events	Total	Proportion	95%−CI	Weight (common)	Weight (random)
Bekloo et al. (2017)	1	5	0.200000	[0.005051; 0.716418]	0.7%	11.5%
Zeb et al. (2019)	2	44	0.045455	[0.005553; 0.154732]	6.2%	15.6%
Orkun et al. (2019)	0	3	0.000000	[0.000000; 0.707598]	0.4%	9.6%
Rehman et al. (2019)	1	300	0.003333	[0.000084; 0.018431]	42.3%	16.3%
Sang et al. (2021)	0	40	0.000000	[0.000000; 0.088097]	5.6%	15.6%
Qiu et al. (2022)	17	25	0.680000	[0.464999; 0.850505]	3.5%	15.1%
Zhang et al. (2021)	1	293	0.003413	[0.000086; 0.018868]	41.3%	16.3%
**Common effect model**		**710**	**0.009818**	**[0.003898; 0.018390]**	**100.0%**	**.**
**Random effects model**			**0.062381**	**[0.000000; 0.251275]**	**.**	**100.0%**

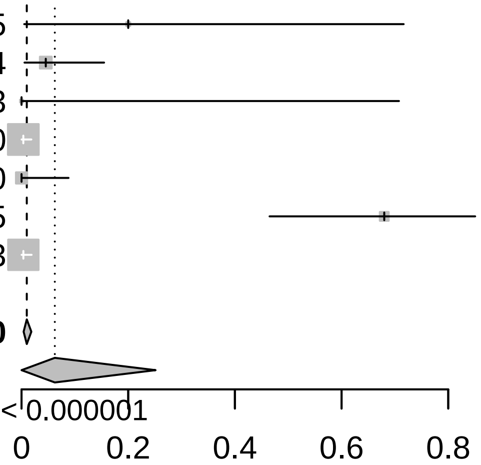

Heterogeneity: $I^2 = 93.1\%$, $\tau^2 = 0.1180$, $p < 0.000001$

Babesia bovis

Study	Events	Total	Proportion	95%−CI	Weight (common)	Weight (random)
Abdallah et al. (2017)	0	20	0.000000	[0.000000; 0.168433]	3.0%	15.7%
Mossaad et al. (2021)	13	239	0.054393	[0.029277; 0.091218]	35.9%	28.2%
Rajabi et al. (2017)	0	105	0.000000	[0.000000; 0.034522]	15.8%	25.8%
Rehman et al. (2019)	0	300	0.000000	[0.000000; 0.012221]	45.1%	28.6%
Sadeddine et al. (2019)	0	1	0.000000	[0.000000; 0.975000]	0.2%	1.5%
**Common effect model**		**665**	**0.007140**	**[0.002170; 0.014956]**	**100.0%**	**.**
**Random effects model**			**0.004408**	**[0.000000; 0.036249]**	**.**	**100.0%**

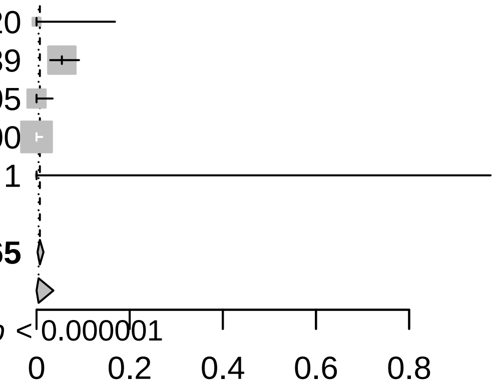

Heterogeneity: $I^2 = 88.2\%$, $\tau^2 = 0.0134$, $p < 0.000001$

Babesia caballi

Study	Events	Total	Proportion	95%−CI	Weight (common)	Weight (random)
Sang et al. (2021)	0	40	0.000000	[0.000000; 0.088097]	43.5%	23.5%
Wen et al. (2019)	58	250	0.232000	[0.181135; 0.289340]	18.0%	22.7%
K?seo?lu AE et al. (2021)	0	4	0.000000	[0.000000; 0.602365]	0.7%	9.0%
Li et al. (2020)	31	240	0.129167	[0.089477; 0.178307]	27.3%	23.2%
Sultankulova et al. (2022)	0	19	0.000000	[0.000000; 0.176467]	10.5%	21.7%
**Common effect model**		**553**	**0.076905**	**[0.054736; 0.099074]**	**100.0%**	**.**
**Random effects model**			**0.082472**	**[0.000000; 0.181813]**	**.**	**100.0%**

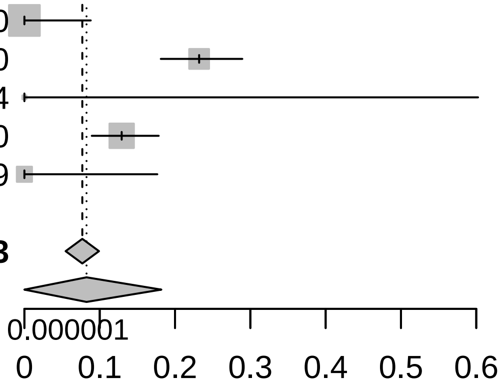

Heterogeneity: $I^2 = 93.8\%$, $\tau^2 = 0.0106$, $p < 0.000001$

Babesia ovis

Study	Events	Total	Proportion	95%−CI	Weight (common)	Weight (random)
Ghafar et al. (2020)	1	212	0.004717	[0.000119; 0.026000]	68.2%	52.6%
Orkun et al. (2019)	0	3	0.000000	[0.000000; 0.707598]	1.0%	2.7%
Spitalska et al. (2005)	3	89	0.033708	[0.007006; 0.095362]	28.6%	38.6%
Razmi et al. (2013)	0	7	0.000000	[0.000000; 0.409616]	2.3%	6.1%
**Common effect model**		**311**	**0.009906**	**[0.001946; 0.023914]**	**100.0%**	**.**
**Random effects model**			**0.011499**	**[0.000142; 0.040628]**	**.**	**100.0%**

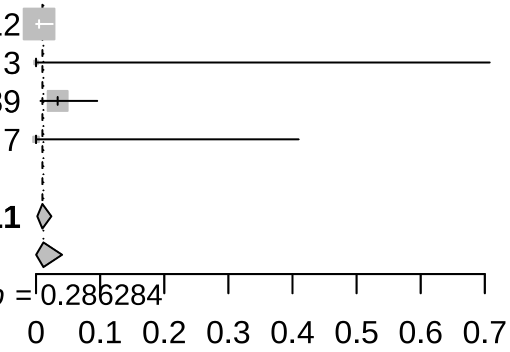

Heterogeneity: $I^2 = 20.6\%$, $\tau^2 = 0.0033$, $p = 0.286284$

uncharacterised *Babesia*

Study	Events	Total	Proportion	95%−CI	Weight (common)	Weight (random)
Omer et al. (2023)	0	44	0.000000	[0.000000; 0.080420]	1.8%	10.5%
Jafarbekloo et al. (2018)	0	73	0.000000	[0.000000; 0.049277]	3.0%	11.1%
Norouzi et al. (2023)	0	264	0.000000	[0.000000; 0.013876]	10.7%	11.9%
Orkun et al. (2022)	0	4	0.000000	[0.000000; 0.602365]	0.2%	4.5%
Polat et al. (2020)	0	5	0.000000	[0.000000; 0.521824]	0.2%	5.1%
Sadeddine et al. (2019)	0	1	0.000000	[0.000000; 0.975000]	0.0%	1.5%
Hasheminasab et al. (2018)	221	776	0.284794	[0.253264; 0.317969]	31.4%	12.1%
Tavassoli et al. (2013)	0	59	0.000000	[0.000000; 0.060609]	2.4%	10.9%
Zhao et al. (2020)	0	853	0.000000	[0.000000; 0.004315]	34.6%	12.1%
K?seo?lu AE et al. (2021)	0	4	0.000000	[0.000000; 0.602365]	0.2%	4.5%
Nader et al. (2018)	0	3	0.000000	[0.000000; 0.707598]	0.1%	3.7%
Kartashov et al. (2020)	0	382	0.000000	[0.000000; 0.009610]	15.5%	12.0%
**Common effect model**		**2468**	**0.031002**	**[0.024531; 0.038203]**	**100.0%**	**.**
**Random effects model**			**0.004623**	**[0.000000; 0.038880]**	**.**	**100.0%**

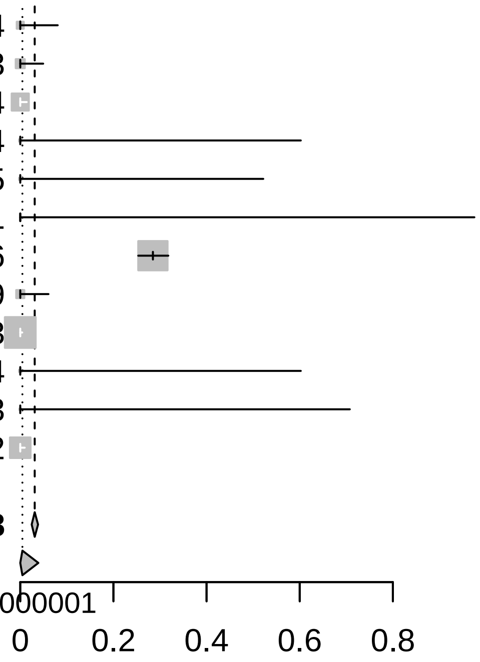

Heterogeneity: $I^2 = 98.4\%$, $\tau^2 = 0.0363$, $p < 0.000001$

Borrelia burgdorferi

Study	Events	Total	Proportion	95%−CI	Weight (common)	Weight (random)
Ashour et al. (2023)	0	66	0.000000	[0.000000; 0.054359]	15.1%	15.1%
Yu et al. (2017)	1	20	0.050000	[0.001265; 0.248733]	4.6%	4.6%
Wen et al. (2019)	0	250	0.000000	[0.000000; 0.014647]	57.3%	57.3%
Nader et al. (2018)	0	3	0.000000	[0.000000; 0.707598]	0.7%	0.7%
Patra G et al. (2020)	0	97	0.000000	[0.000000; 0.037316]	22.2%	22.2%
**Common effect model**		**436**	**0.000107**	**[0.000000; 0.003277]**	**100.0%**	**.**
**Random effects model**			**0.000107**	**[0.000000; 0.003277]**	**.**	**100.0%**

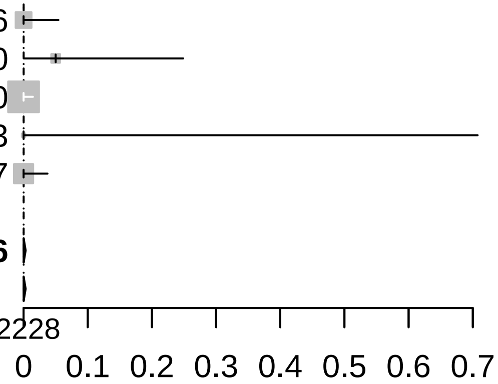

Heterogeneity: $I^2 = 0.0\%$, $\tau^2 = 0$, $p = 0.422228$

uncharacterised *Borrelia*

Study	Events	Total	Proportion	95%−CI	Weight (common)	Weight (random)
Ghafar et al. (2020)	14	212	0.066038	[0.036571; 0.108319]	19.3%	27.2%
Zhao et al. (2020)	0	861	0.000000	[0.000000; 0.004275]	78.6%	28.0%
K?seo?lu AE et al. (2021)	0	4	0.000000	[0.000000; 0.602365]	0.4%	9.3%
Khan M et al. (2023)	0	10	0.000000	[0.000000; 0.308497]	0.9%	15.5%
Orkun et al. (2022)	0	4	0.000000	[0.000000; 0.602365]	0.4%	9.3%
Polat E et al. (2020)	2	5	0.400000	[0.052745; 0.853367]	0.5%	10.7%
**Common effect model**		**1096**	**0.002848**	**[0.000566; 0.006873]**	**100.0%**	**.**
**Random effects model**			**0.020598**	**[0.000000; 0.102353]**	**.**	**100.0%**

Heterogeneity: $I^2 = 90.8\%$, $\tau^2 = 0.0303$, $p < 0.000001$

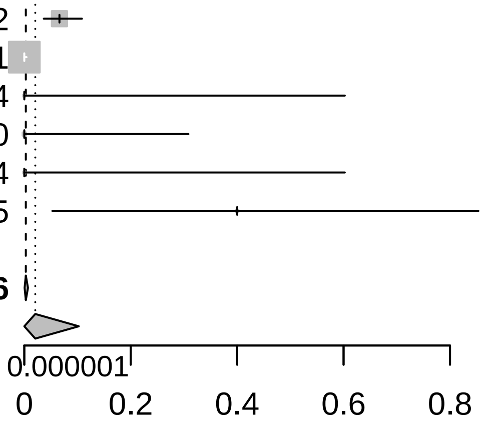

uncharacterised *Brucella*

Study	Events	Total	Proportion	95%−CI	Weight (common)	Weight (random)
Li et al. (2020)	111	240	0.462500	[0.398147; 0.527790]	48.8%	48.8%
Wen et al. (2019)	111	250	0.444000	[0.381401; 0.507943]	51.2%	51.2%
**Common effect model**		**490**	**0.453029**	**[0.408961; 0.497097]**	**100.0%**	**.**
**Random effects model**			**0.453029**	**[0.408961; 0.497097]**	**.**	**100.0%**

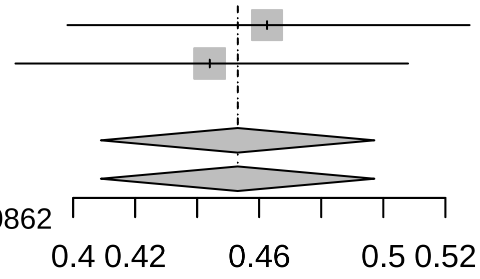

Heterogeneity: $I^2 = 0.0\%$, $\tau^2 = 0$, $p = 0.680862$

Coxiella burnetii

Study	Events	Total	Proportion	95%−CI	Weight (common)	Weight (random)
Ali et al. (2023)	2	28	0.071429	[0.008770; 0.235035]	1.6%	7.1%
ALTAY et al. (2013)	0	133	0.000000	[0.000000; 0.027355]	7.8%	7.3%
Dhaka et al. (2022)	0	416	0.000000	[0.000000; 0.008828]	24.5%	7.4%
NourollahiFard et al. (2016)	1	395	0.002532	[0.000064; 0.014024]	23.2%	7.4%
Dabaja et al. (2020)	6	6	1.000000	[0.540742; 1.000000]	0.4%	6.3%
Ghashghaei et al. (2016)	2	168	0.011905	[0.001445; 0.042341]	9.9%	7.4%
Ghoneim et al. (2020)	1	19	0.052632	[0.001332; 0.260281]	1.1%	7.0%
Hussain et al. (2021)	14	64	0.218750	[0.125068; 0.339696]	3.8%	7.3%
Loftis et al. (2006)	0	10	0.000000	[0.000000; 0.308497]	0.6%	6.7%
Ni et al. (2020)	24	25	0.960000	[0.796483; 0.998988]	1.5%	7.1%
Patra et al. (2020)	0	97	0.000000	[0.000000; 0.037316]	5.7%	7.3%
Sultankulova et al. (2022)	10	19	0.526316	[0.288643; 0.755525]	1.1%	7.0%
Wen et al. (2019)	0	250	0.000000	[0.000000; 0.014647]	14.7%	7.4%
Ghazanabad et al. (2023)	11	70	0.157143	[0.081144; 0.263800]	4.1%	7.3%
**Common effect model**		**1700**	**0.009849**	**[0.005710; 0.015095]**	**100.0%**	**.**
**Random effects model**			**0.127881**	**[0.010951; 0.343822]**	**.**	**100.0%**

Heterogeneity: $I^2 = 96.4\%$, $\tau^2 = 0.2394$, $p < 0.000001$

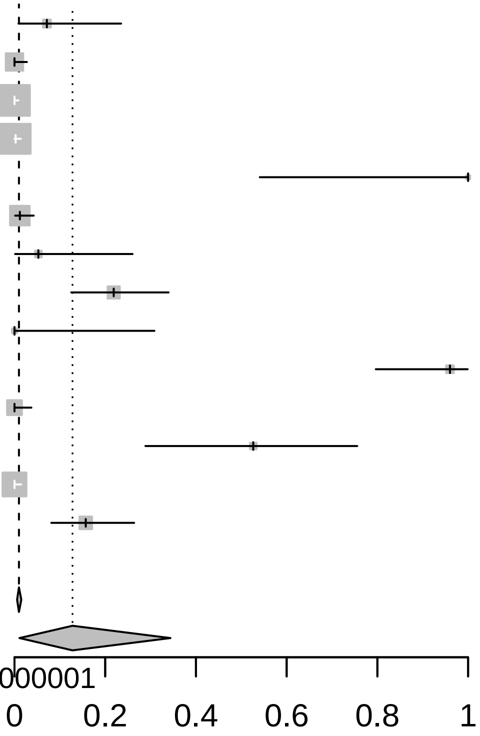

uncharacterised *Coxiella*

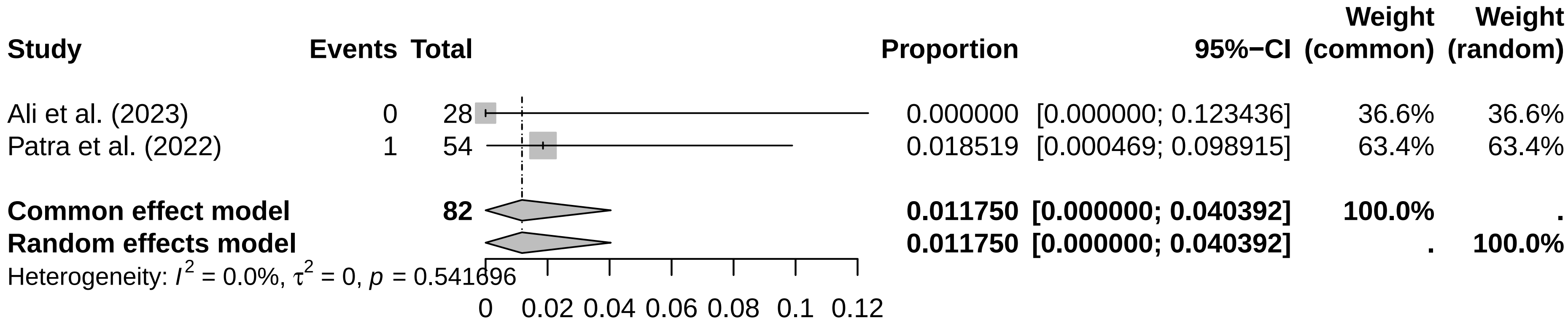

Francisella-like endosymbiont

Study	Events	Total	Proportion	95%−CI	Weight (common)	Weight (random)
Duzlu et al. (2016)	0	120	0.000000	[0.000000; 0.030273]	35.5%	33.9%
Ghafar et al. (2020)	199	212	0.938679	[0.897422; 0.966949]	62.7%	34.0%
Ghafar et al. (2020)	6	6	1.000000	[0.540742; 1.000000]	1.8%	32.1%
**Common effect model**		**338**	**0.570532**	**[0.517460; 0.622802]**	**100.0%**	**.**
**Random effects model**			**0.664482**	**[0.000000; 1.000000]**	**.**	**100.0%**

Heterogeneity: $I^2 = 99.6\%$, $\tau^2 = 0.7033$, $p < 0.000001$

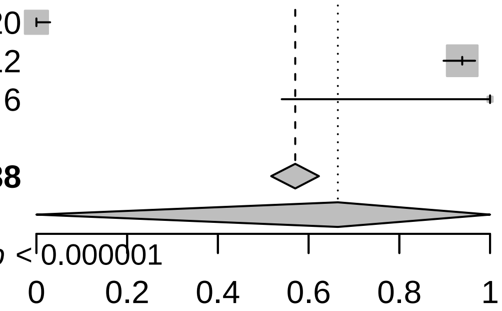

Rickettsia aeschlimannii

Study	Events	Total	Proportion	95%−CI	Weight (common)	Weight (random)
Fern..ndez de Mera et al. (2017)	1	6	0.166667	[0.004211; 0.641235]	4.7%	4.7%
Loftis et al. (2006)	0	10	0.000000	[0.000000; 0.308497]	7.9%	7.9%
Orkun et al. (2019)	0	3	0.000000	[0.000000; 0.707598]	2.4%	2.4%
Qu et al. (2019)	0	75	0.000000	[0.000000; 0.047995]	59.1%	59.0%
Majid et al. (2023)	0	33	0.000000	[0.000000; 0.105763]	26.0%	26.0%
**Common effect model**		**127**	**0.000395**	**[0.000000; 0.011369]**	**100.0%**	**.**
**Random effects model**			**0.000396**	**[0.000000; 0.011387]**	**.**	**100.0%**

Heterogeneity: I^2 = 1.1%, τ^2 < 0.0001, p = 0.400103

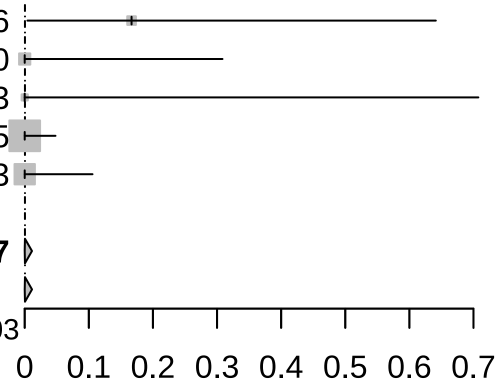

Rickettsia slovaca

Study	Events	Total	Proportion	95%−CI	Weight (common)	Weight (random)
Ghafar et al. (2020)	6	6	1.000000	[0.540742; 1.000000]	7.1%	33.3%
Orkun et al. (2019)	0	3	0.000000	[0.000000; 0.707598]	3.6%	31.7%
Li et al. (2019)	0	75	0.000000	[0.000000; 0.047995]	89.3%	35.0%
**Common effect model**		**84**	**0.012536**	**[0.000028; 0.047252]**	**100.0%**	**.**
**Random effects model**			**0.249900**	**[0.000000; 0.999584]**	**.**	**100.0%**

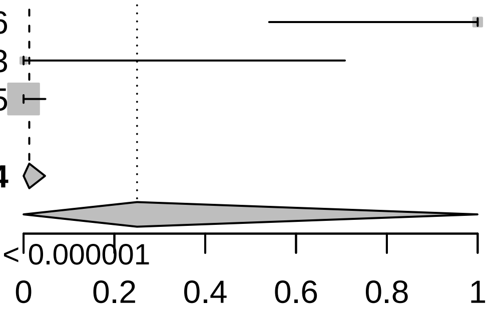

Heterogeneity: $I^2 = 96.4\%$, $\tau^2 = 0.7821$, $p < 0.000001$

Rickettsia raoultii

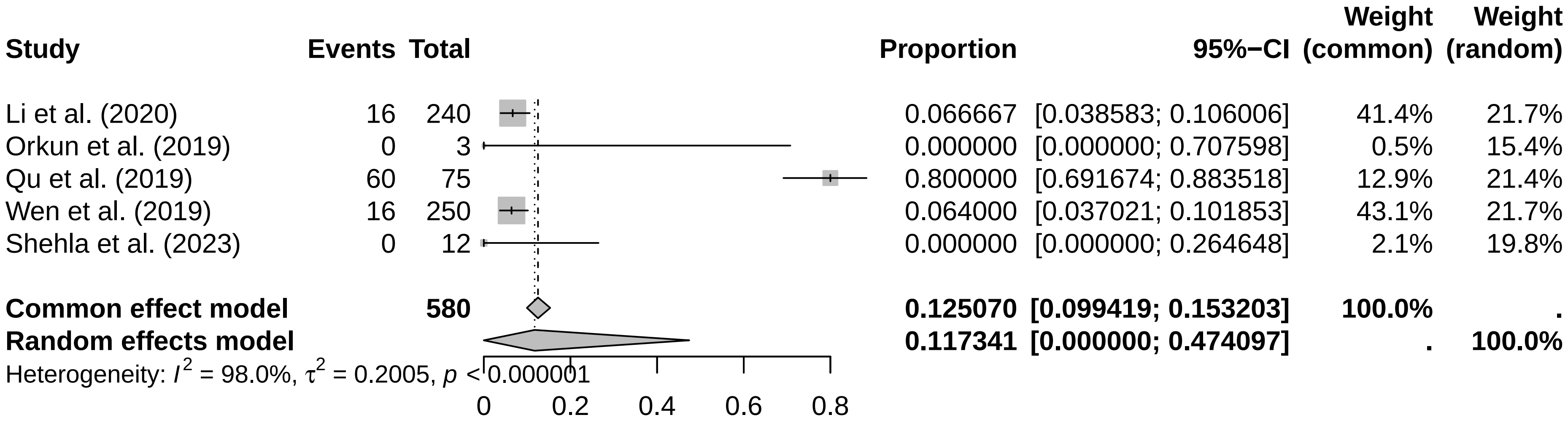

Rickettsia massiliae

Study	Events	Total	Proportion	95%−CI	Weight (common)	Weight (random)
Ghafar et al. (2020)	6	6	1.000000	[0.540742; 1.000000]	1.0%	18.6%
Ghafar et al. (2020)	3	212	0.014151	[0.002928; 0.040796]	34.8%	20.5%
Rehman et al. (2019)	1	300	0.003333	[0.000084; 0.018431]	49.3%	20.5%
Yu et al. (2017)	1	20	0.050000	[0.001265; 0.248733]	3.3%	19.9%
Shehla et al. (2023)	0	71	0.000000	[0.000000; 0.050629]	11.7%	20.4%
**Common effect model**		**609**	**0.008596**	**[0.002821; 0.017469]**	**100.0%**	**.**
**Random effects model**			**0.132835**	**[0.000000; 0.636276]**	**.**	**100.0%**

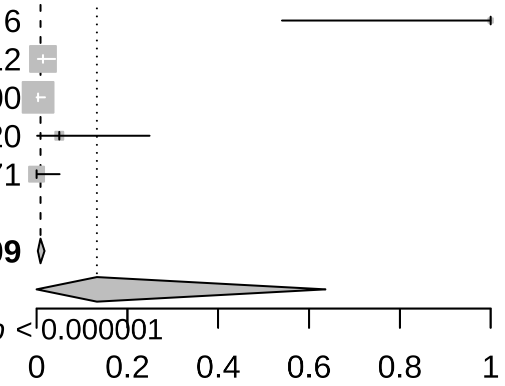

Heterogeneity: $I^2 = 93.1\%$, $\tau^2 = 0.3829$, $p < 0.000001$

uncharacterised *Rickettsia*

Study	Events	Total	Proportion	95%−CI	Weight (common)	Weight (random)
Demir et al. (2020)	0	4	0.000000	[0.000000; 0.602365]	0.3%	6.6%
Fern..ndez de Mera et al. (2017)	2	6	0.333333	[0.043272; 0.777222]	0.4%	7.5%
Ghafar et al. (2020)	4	212	0.018868	[0.005164; 0.047602]	13.6%	10.2%
Hamlili et al. (2022)	0	1	0.000000	[0.000000; 0.975000]	0.1%	3.2%
Kartashov et al. (2020)	0	382	0.000000	[0.000000; 0.009610]	24.5%	10.2%
Lange et al. (1992)	2	2	1.000000	[0.158114; 1.000000]	0.1%	4.8%
Loftis et al. (2006)	0	10	0.000000	[0.000000; 0.308497]	0.6%	8.4%
Perveen et al. (2021)	0	46	0.000000	[0.000000; 0.077062]	2.9%	9.8%
Orkun et al. (2022)	0	4	0.000000	[0.000000; 0.602365]	0.3%	6.6%
Polat et al. (2020)	1	5	0.200000	[0.005051; 0.716418]	0.3%	7.1%
Zhao et al. (2020)	0	861	0.000000	[0.000000; 0.004275]	55.2%	10.3%
Xiao et al. (2022)	11	24	0.458333	[0.255530; 0.671792]	1.5%	9.4%
Nader et al. (2018)	0	3	0.000000	[0.000000; 0.707598]	0.2%	5.9%
**Common effect model**		**1560**	**0.001298**	**[0.000126; 0.003698]**	**100.0%**	**.**
**Random effects model**			**0.056118**	**[0.000875; 0.188210]**	**.**	**100.0%**

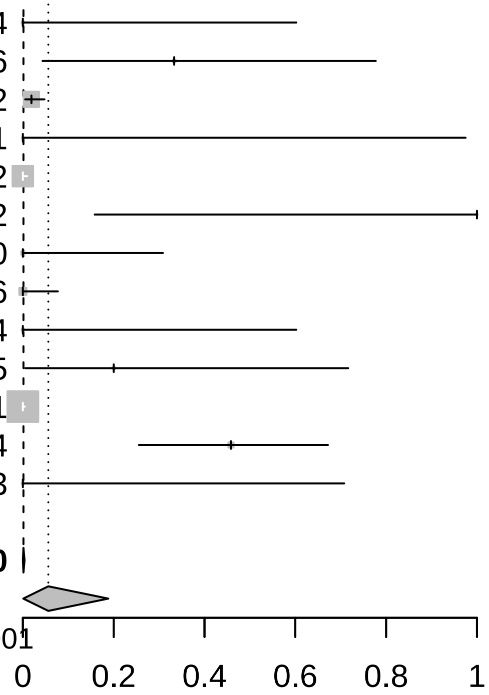

Heterogeneity: $I^2 = 87.3\%$, $\tau^2 = 0.1110$, $p < 0.000001$

Theileria annulata

Study	Events	Total	Proportion	95%−CI	Weight (common)	Weight (random)
Inci et al. (2008)	75	427	0.175644	[0.140735; 0.215120]	3.8%	3.2%
Omer et al. (2022)	17	44	0.386364	[0.243572; 0.545045]	0.4%	3.0%
Abdallah et al. (2017)	3	20	0.150000	[0.032071; 0.378927]	0.2%	2.7%
Z.Ali et al. (2013)	10	20	0.500000	[0.271958; 0.728042]	0.2%	2.7%
Tiwari et al. (2015)	36	60	0.600000	[0.465405; 0.724378]	0.5%	3.1%
ArjmandYamchi et al. (2016)	47	143	0.328671	[0.252492; 0.412118]	1.3%	3.2%
Dehuri et al. (2022)	26	178	0.146067	[0.097688; 0.206653]	1.6%	3.2%
Mossaad et al. (2021)	113	239	0.472803	[0.408101; 0.538189]	2.1%	3.2%
Sayin et al. (2003)	4	161	0.024845	[0.006810; 0.062388]	1.4%	3.2%
Sohrabi et al. (2013)	2	43	0.046512	[0.005683; 0.158111]	0.4%	3.0%
Haque et al. (2010)	31	266	0.116541	[0.080573; 0.161328]	2.4%	3.2%
Arjmand Yamchi et al. (2014)	47	143	0.328671	[0.252492; 0.412118]	1.3%	3.2%
Zeb et al. (2019)	12	44	0.272727	[0.149577; 0.427896]	0.4%	3.0%
Kartashov et al. (2020)	3	382	0.007853	[0.001622; 0.022778]	3.4%	3.2%
Samiurahman Amiri et al. (2019)	7	258	0.027132	[0.010976; 0.055100]	2.3%	3.2%
Rahmani−Varmale et al. (2019)	2	55	0.036364	[0.004435; 0.125264]	0.5%	3.0%
Perveen et al. (2021)	1	46	0.021739	[0.000550; 0.115272]	0.4%	3.0%
Orkun et al. (2019)	0	3	0.000000	[0.000000; 0.707598]	0.0%	1.5%
Razmi et al. (2013)	0	8	0.000000	[0.000000; 0.369417]	0.1%	2.2%
Rehman et al. (2019)	26	300	0.086667	[0.057395; 0.124407]	2.7%	3.2%
Sadeddine et al. (2019)	0	1	0.000000	[0.000000; 0.975000]	0.0%	0.7%
Sang et al. (2021)	0	40	0.000000	[0.000000; 0.088097]	0.4%	3.0%
Sultankulova et al. (2022)	0	19	0.000000	[0.000000; 0.176467]	0.2%	2.7%
Tavassoli et al. (2011)	79	203	0.389163	[0.321696; 0.459917]	1.8%	3.2%
Wang et al. (2014)	3	50	0.060000	[0.012549; 0.165482]	0.4%	3.0%
Aihemaiti et al. (2018)	20	248	0.080645	[0.049955; 0.121803]	2.2%	3.2%
Wang et al. (2016)	1	3223	0.000310	[0.000008; 0.001727]	28.8%	3.3%
Wang et al. (2015)	1	39	0.025641	[0.000649; 0.134764]	0.3%	3.0%
Xie et al. (2018)	1	3750	0.000267	[0.000007; 0.001485]	33.6%	3.3%
Xie et al. (2021)	11	120	0.091667	[0.046652; 0.158099]	1.1%	3.2%
Zhang et al. (2021)	67	293	0.228669	[0.181835; 0.281083]	2.6%	3.2%
Velusamy et al. (2023)	26	76	0.342105	[0.237057; 0.459876]	0.7%	3.1%
Shiri et al. (2024)	0	18	0.000000	[0.000000; 0.185302]	0.2%	2.7%
Sajid et al. (2023)	126	252	0.500000	[0.436601; 0.563399]	2.3%	3.2%
**Common effect model**		**11172**	**0.026451**	**[0.023557; 0.029508]**	**100.0%**	**.**
**Random effects model**			**0.118259**	**[0.065560; 0.183881]**	**.**	**100.0%**

Heterogeneity: $I^2 = 98.7\%$, $\tau^2 = 0.0679$, $p = 0$

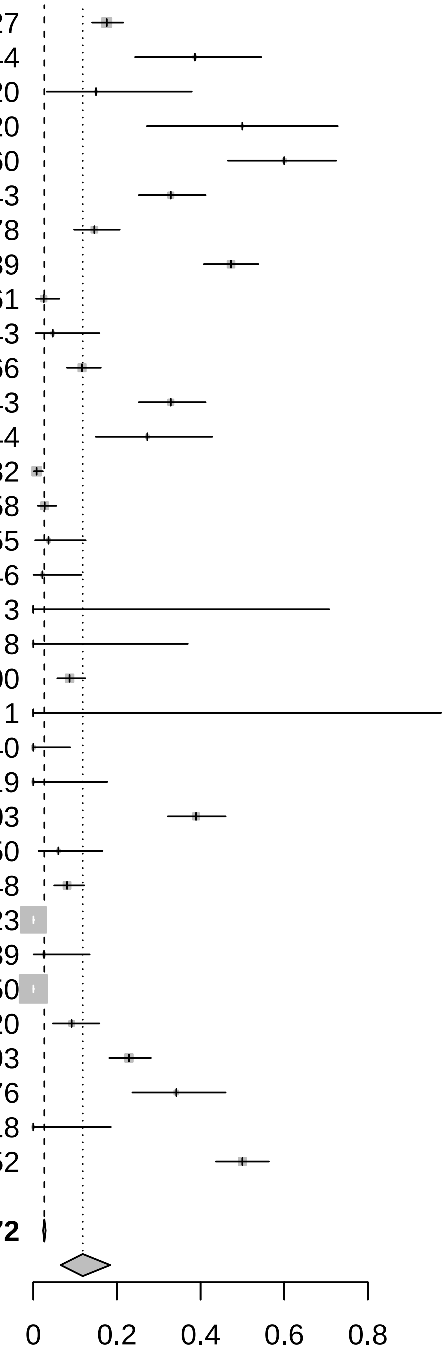

Theileria equi

Study	Events	Total	Proportion	95%−CI	Weight (common)	Weight (random)
Sumbria et al. (2017)	10	68	0.147059	[0.072840; 0.253863]	0.0%	17.4%
Abedi et al. (2013)	0	1	0.000000	[0.000000; 0.975000]	0.0%	2.2%
Li et al. (2020)	58	240	0.241667	[0.188918; 0.300917]	0.0%	19.0%
Sadeddine et al. (2019)	0	1	0.000000	[0.000000; 0.975000]	0.0%	2.2%
Sang et al. (2021)	0	40	0.000000	[0.000000; 0.088097]	0.0%	19.7%
Wang et al. (2016)	0	3223	0.000000	[0.000000; 0.001144]	100.0%	20.2%
Wen et al. (2019)	48	250	0.192000	[0.145091; 0.246375]	0.0%	19.2%
**Common effect model**		**3823**	**0.000034**	**[0.000000; 0.000464]**	**100.0%**	**.**
**Random effects model**			**0.108367**	**[0.013951; 0.202783]**	**.**	**100.0%**

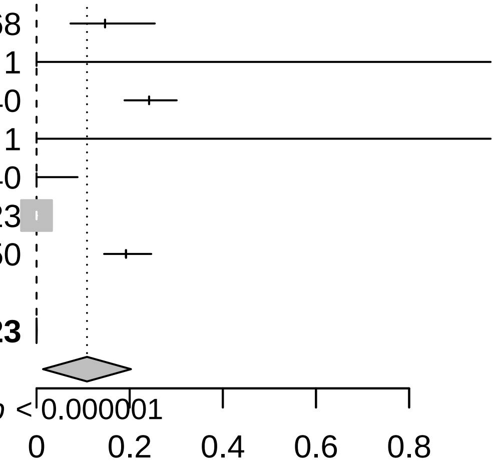

Heterogeneity: $I^2 = 95.9\%$, $\tau^2 = 0.0115$, $p < 0.000001$

Theileria lestoquardi

Study	Events	Total	Proportion	95%−CI	Weight (common)	Weight (random)
Abdigoudarzi et al. (2013)	2	20	0.100000	[0.012349; 0.316983]	2.9%	9.5%
Zarei et al. (2017)	0	18	0.000000	[0.000000; 0.185302]	2.6%	9.1%
Norouzi et al. (2023)	0	264	0.000000	[0.000000; 0.013876]	37.7%	15.4%
Mohammadi et al. (2017)	0	185	0.000000	[0.000000; 0.019742]	26.4%	15.1%
Rahmani−Varmale et al. (2019)	1	55	0.018182	[0.000460; 0.097191]	7.9%	12.9%
Razmi et al. (2013)	0	8	0.000000	[0.000000; 0.369417]	1.1%	5.8%
Razmi et al. (2013)	0	7	0.000000	[0.000000; 0.409616]	1.0%	5.3%
Spitalska et al. (2005)	8	89	0.089888	[0.039610; 0.169452]	12.7%	14.0%
Nasser et al. (2014)	5	54	0.092593	[0.030753; 0.203002]	7.7%	12.9%
**Common effect model**		**700**	**0.006772**	**[0.002055; 0.014195]**	**100.0%**	**.**
**Random effects model**			**0.016897**	**[0.000672; 0.054124]**	**.**	**100.0%**

Heterogeneity: $I^2 = 83.0\%$, $\tau^2 = 0.0175$, $p < 0.000001$

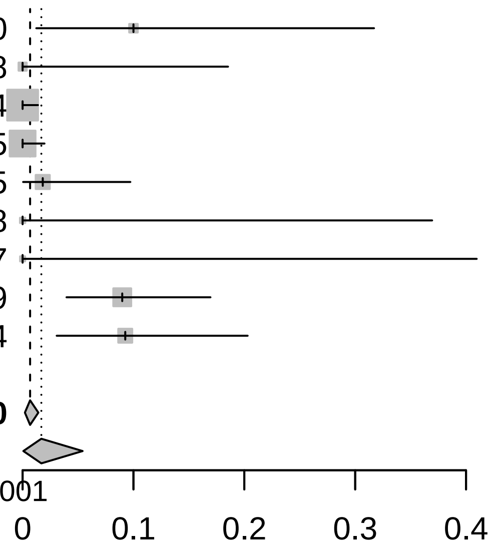

Theileria ovis

Study	Events	Total	Proportion	95%−CI	Weight (common)	Weight (random)
Bekloo et al. (2017)	1	5	0.200000	[0.005051; 0.716418]	0.4%	4.8%
Zarei et al. (2017)	0	18	0.000000	[0.000000; 0.185302]	1.4%	7.3%
Li et al. (2020)	17	240	0.070833	[0.041802; 0.110990]	18.5%	8.9%
Norouzi et al. (2023)	1	264	0.003788	[0.000096; 0.020923]	20.3%	8.9%
Rahmani−Varmale et al. (2019)	0	55	0.000000	[0.000000; 0.064871]	4.2%	8.4%
Perveen et al. (2021)	1	46	0.021739	[0.000550; 0.115272]	3.5%	8.3%
Razmi et al. (2013)	0	8	0.000000	[0.000000; 0.369417]	0.6%	5.9%
Rehman et al. (2019)	0	300	0.000000	[0.000000; 0.012221]	23.1%	8.9%
Sadeddine et al. (2019)	0	1	0.000000	[0.000000; 0.975000]	0.1%	1.7%
Sang et al. (2021)	0	40	0.000000	[0.000000; 0.088097]	3.1%	8.2%
Wen et al. (2019)	11	250	0.044000	[0.022166; 0.077363]	19.2%	8.9%
K?seo?lu et al. (2021)	0	4	0.000000	[0.000000; 0.602365]	0.3%	4.3%
Shiri et al. (2024)	0	18	0.000000	[0.000000; 0.185302]	1.4%	7.3%
Li et al. (2012)	29	50	0.580000	[0.432060; 0.718118]	3.8%	8.3%
**Common effect model**		**1299**	**0.020397**	**[0.013423; 0.028789]**	**100.0%**	**.**
**Random effects model**			**0.023792**	**[0.000172; 0.085433]**	**.**	**100.0%**

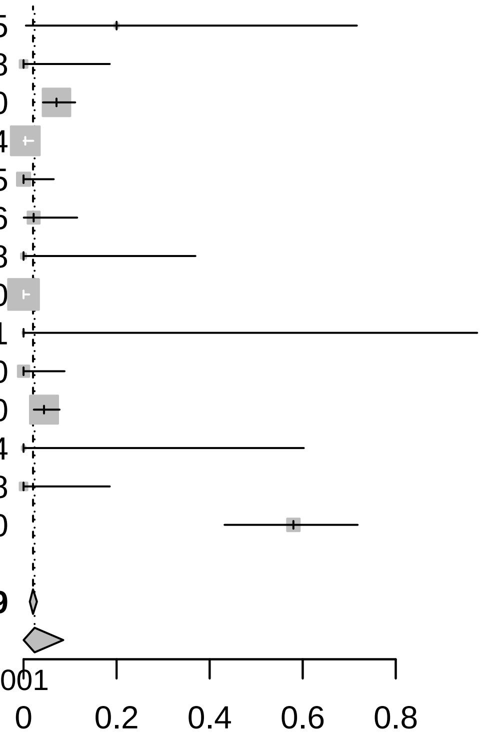

Heterogeneity: $I^2 = 92.3\%$, $\tau^2 = 0.0579$, $p < 0.000001$

Theileria luwenshuni

Study	Events	Total	Proportion	95%−CI	Weight (common)	Weight (random)
Abdallah et al. (2017)	0	20	0.000000	[0.000000; 0.168433]	11.4%	28.8%
Patra et al. (2022)	11	106	0.103774	[0.052953; 0.178097]	60.2%	36.9%
Li et al. (2012)	0	50	0.000000	[0.000000; 0.071122]	28.4%	34.4%
**Common effect model**		**176**	**0.038516**	**[0.015216; 0.071871]**	**100.0%**	**.**
**Random effects model**			**0.014568**	**[0.000000; 0.115247]**	**.**	**100.0%**

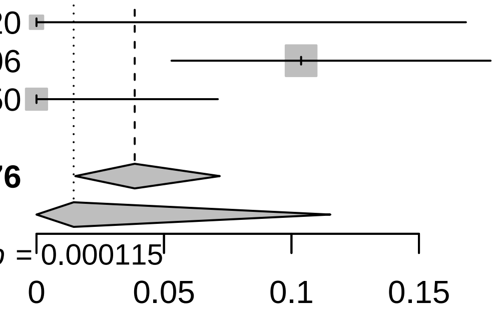

Heterogeneity: $I^2 = 89.0\%$, $\tau^2 = 0.0335$, $p = 0.000115$

uncharacterised *Theileria*

Study	Events	Total	Proportion	95%−CI	Weight (common)	Weight (random)
Ghafar et al. (2020)	6	6	1.000000	[0.540742; 1.000000]	0.1%	6.4%
Adegoke et al. (2020)	72	198	0.363636	[0.296610; 0.434814]	2.4%	8.2%
Aktas et al. (2004)	1562	4594	0.340009	[0.326307; 0.353914]	54.9%	8.3%
Asadollahi et al. (2018)	41	390	0.105128	[0.076504; 0.139909]	4.7%	8.2%
Jafarbekloo et al. (2018)	0	73	0.000000	[0.000000; 0.049277]	0.9%	8.1%
Razmi et al. (2003)	30	200	0.150000	[0.103550; 0.207159]	2.4%	8.2%
Hasheminasab et al. (2018)	431	776	0.555412	[0.519651; 0.590752]	9.3%	8.3%
Salih et al. (2005)	75	285	0.263158	[0.212981; 0.318335]	3.4%	8.2%
Luo et al. (2020)	48	100	0.480000	[0.379005; 0.582210]	1.2%	8.1%
K?seo?lu AE et al. (2021)	0	4	0.000000	[0.000000; 0.602365]	0.0%	5.8%
Sangwan et al. (1986)	546	1650	0.330909	[0.308220; 0.354202]	19.7%	8.3%
Orkun et al. (2022)	0	4	0.000000	[0.000000; 0.602365]	0.0%	5.8%
Spitalska et al. (2005)	1	89	0.011236	[0.000284; 0.061018]	1.1%	8.1%
**Common effect model**		**8369**	**0.328642**	**[0.318618; 0.338744]**	**100.0%**	**.**
**Random effects model**			**0.230483**	**[0.080448; 0.428770]**	**.**	**100.0%**

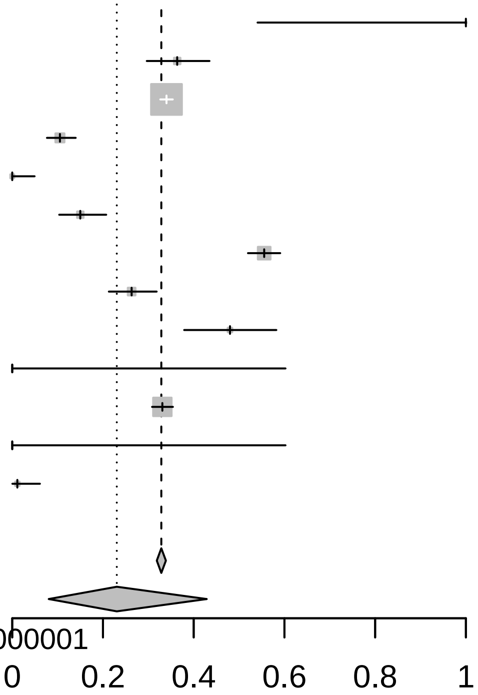

Heterogeneity: $I^2 = 97.9\%$, $\tau^2 = 0.1428$, $p < 0.000001$

uncharacterised *Hepatozoon*

Study	Events	Total	Proportion	95%−CI	Weight (common)	Weight (random)
Ghafar et al. (2020)	1	212	0.004717	[0.000119; 0.026000]	96.4%	96.4%
K?seo?lu et al. (2021)	0	4	0.000000	[0.000000; 0.602365]	1.8%	1.8%
Orkun et al. (2022)	0	4	0.000000	[0.000000; 0.602365]	1.8%	1.8%
**Common effect model**		**220**	**0.004381**	**[0.000000; 0.017403]**	**100.0%**	**.**
**Random effects model**			**0.004381**	**[0.000000; 0.017403]**	**.**	**100.0%**

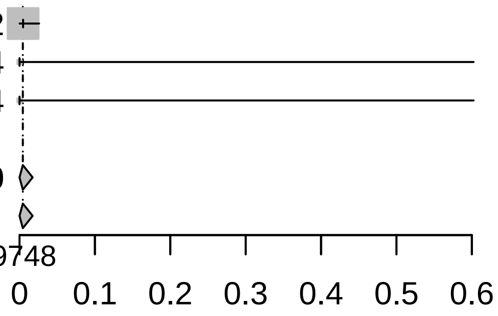

Heterogeneity: $I^2 = 0.0\%$, $\tau^2 = 0$, $p = 0.929748$

Crimean-Congo hemorrhagic fever virus

Study	Events	Total	Proportion	95%−CI	Weight (common)	Weight (random)
Yaser et al. (2011)	1	12	0.083333	[0.002108; 0.384796]	0.3%	2.9%
Ozdarendeli et al. (2008)	0	12	0.000000	[0.000000; 0.264648]	0.3%	2.9%
Shuaib et al. (2020)	0	848	0.000000	[0.000000; 0.004341]	21.4%	5.0%
Albayrak et al. (2016)	0	2	0.000000	[0.000000; 0.841886]	0.1%	0.9%
Champour et al. (2016)	7	29	0.241379	[0.102984; 0.435400]	0.7%	3.8%
Choubdar et al. (2019)	0	1026	0.000000	[0.000000; 0.003589]	25.9%	5.0%
Petrova et al. (2013)	58	1026	0.056530	[0.043202; 0.072469]	25.9%	5.0%
Farhadpour et al. (2016)	3	52	0.057692	[0.012059; 0.159473]	1.3%	4.3%
Kayedi et al. (2015)	12	91	0.131868	[0.070035; 0.219020]	2.3%	4.6%
Kasi et al. (2019)	4	20	0.200000	[0.057334; 0.436614]	0.5%	3.5%
Sedaghat et al. (2017)	3	130	0.023077	[0.004785; 0.065959]	3.3%	4.7%
Mohammadian et al. (2016)	3	17	0.176471	[0.037985; 0.434318]	0.4%	3.3%
Champour et al. (2014)	7	29	0.241379	[0.102984; 0.435400]	0.7%	3.8%
Salehi−Vaziri et al. (2020)	0	37	0.000000	[0.000000; 0.094891]	0.9%	4.0%
Sharifinia et al. (2014)	1	125	0.008000	[0.000203; 0.043767]	3.2%	4.7%
Orkun et al. (2017)	0	4	0.000000	[0.000000; 0.602365]	0.1%	1.5%
Williams et al. (2000)	16	122	0.131148	[0.076858; 0.204224]	3.1%	4.7%
Shahhosseini et al. (2017)	0	16	0.000000	[0.000000; 0.205907]	0.4%	3.2%
Sultankulova et al. (2022)	0	138	0.000000	[0.000000; 0.026377]	3.5%	4.7%
Tekin et al. (2012)	1	9	0.111111	[0.002809; 0.482497]	0.2%	2.5%
Yadav et al. (2013)	2	2	1.000000	[0.158114; 1.000000]	0.1%	0.9%
Yadav et al. (2019)	2	61	0.032787	[0.003996; 0.113472]	1.5%	4.4%
Nowotny et al. (2018)	1	14	0.071429	[0.001807; 0.338684]	0.4%	3.0%
Tahmasebi et al. (2010)	2	11	0.181818	[0.022831; 0.517756]	0.3%	2.8%
Telmadarraiy et al. (2008)	1	10	0.100000	[0.002529; 0.445016]	0.3%	2.6%
Biglari et al. (2016)	3	70	0.042857	[0.008927; 0.120179]	1.8%	4.4%
Saghafipour et al. (2019)	3	15	0.200000	[0.043312; 0.480891]	0.4%	3.1%
Fakoorziba et al. (2012)	1	34	0.029412	[0.000744; 0.153268]	0.9%	4.0%
**Common effect model**		**3962**	**0.014333**	**[0.010868; 0.018269]**	**100.0%**	**.**
**Random effects model**			**0.054662**	**[0.026289; 0.092543]**	**.**	**100.0%**

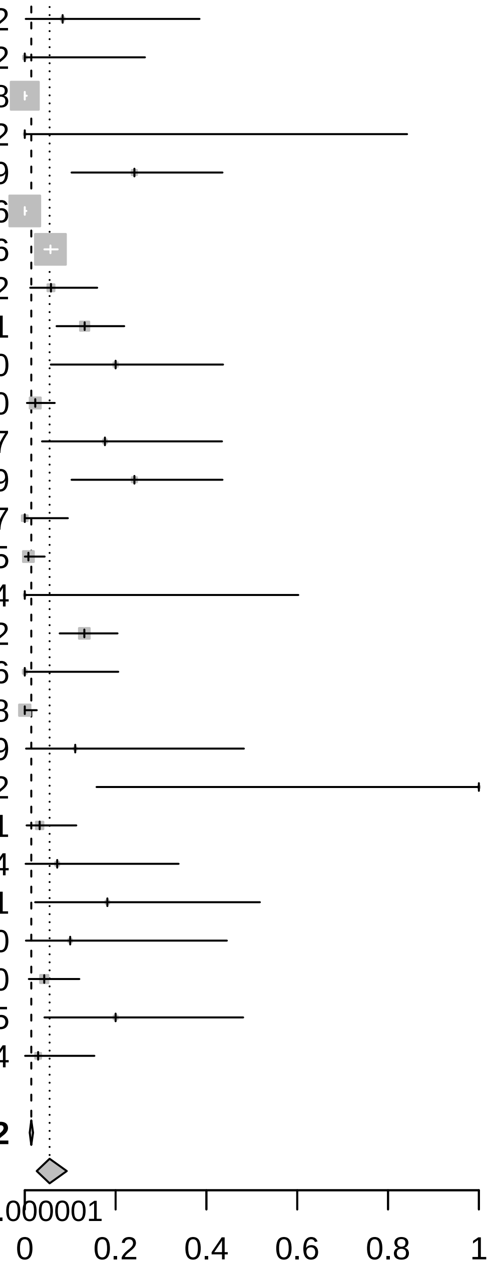

Heterogeneity: $I^2 = 91.7\%$, $\tau^2 = 0.0278$, $p < 0.000001$

Ehrlichia canis

Study	Events	Total	Proportion	95%−CI	Weight (common)	Weight (random)
Kartashov et al. (2020)	2	382	0.005236	[0.000635; 0.018784]	96.0%	96.0%
Atefeh et al. (2013)	0	6	0.000000	[0.000000; 0.459258]	1.5%	1.5%
Loftis et al. (2006)	0	10	0.000000	[0.000000; 0.308497]	2.5%	2.5%
**Common effect model**		**398**	**0.004824**	**[0.000416; 0.014008]**	**100.0%**	**.**
**Random effects model**			**0.004824**	**[0.000416; 0.014008]**	**.**	**100.0%**

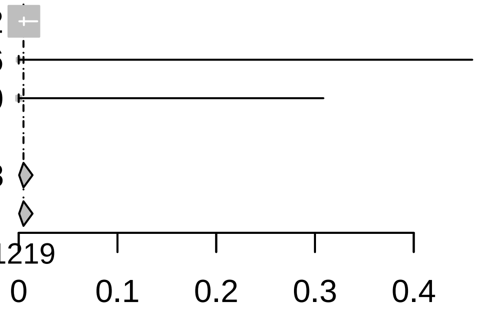

Heterogeneity: $I^2 = 0.0\%$, $\tau^2 = 0$, $p = 0.851219$

uncharacterised *Bartonella*

Study	Events	Total	Proportion	95%−CI	Weight (common)	Weight (random)
K?seo?lu et al. (2021)	0	4	0.000000	[0.000000; 0.602365]	1.6%	1.6%
Ghafar et al. (2020)	1	212	0.004717	[0.000119; 0.026000]	84.1%	84.1%
Hamlili et al. (2022)	0	1	0.000000	[0.000000; 0.975000]	0.4%	0.4%
Loftis et al. (2006)	0	10	0.000000	[0.000000; 0.308497]	4.0%	4.0%
Sadeddine et al. (2019)	0	1	0.000000	[0.000000; 0.975000]	0.4%	0.4%
Xiao et al. (2022)	0	24	0.000000	[0.000000; 0.142474]	9.5%	9.5%
**Common effect model**		**252**	**0.003340**	**[0.000000; 0.014226]**	**100.0%**	**.**
**Random effects model**			**0.003340**	**[0.000000; 0.014226]**	**.**	**100.0%**

Heterogeneity: $I^2 = 0.0\%$, $\tau^2 = 0$, $p = 0.986300$

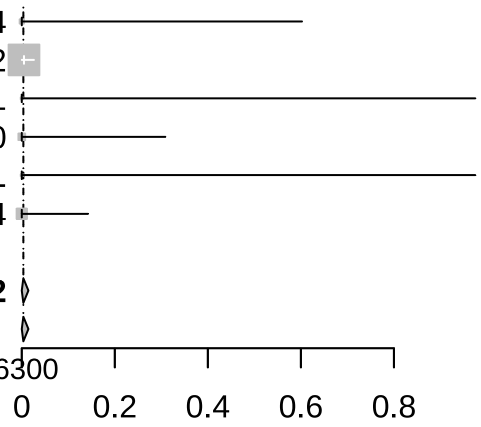

Anaplasma platys-like endosymbiont

Study	Events	Total	Proportion	95%−CI	Weight (common)	Weight (random)
Rehman et al. (2019)	5	300	0.016667	[0.005433; 0.038464]	97.8%	97.8%
Yu et al. (2017)	1	20	0.050000	[0.001265; 0.248733]	2.2%	2.2%
**Common effect model**		**320**	**0.017416**	**[0.003093; 0.031739]**	**100.0%**	**.**
**Random effects model**			**0.017416**	**[0.003093; 0.031739]**	**.**	**100.0%**

Heterogeneity: $I^2 = 0.0\%$, $\tau^2 = 0$, $p = 0.498881$

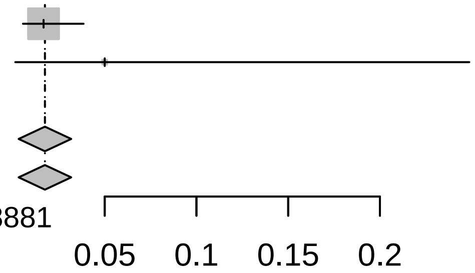

Figure S7: Meta-analysis of pathogens carried by hosts

Anaplasma centrale

Study	Events	Total	Proportion	95%−CI	Weight (common)	Weight (random)
Ghafar et al. (2020)	1	8	0.125000	[0.003160; 0.526510]	0.8%	0.8%
Patra et al. (2022)	140	1053	0.132953	[0.113020; 0.154963]	99.2%	99.2%
**Common effect model**		**1061**	**0.132890**	**[0.112465; 0.153316]**	**100.0%**	**.**
**Random effects model**			**0.132890**	**[0.112465; 0.153316]**	**.**	**100.0%**

Heterogeneity: $I^2 = 0.0\%$, $\tau^2 = 0$, $p = 0.945984$

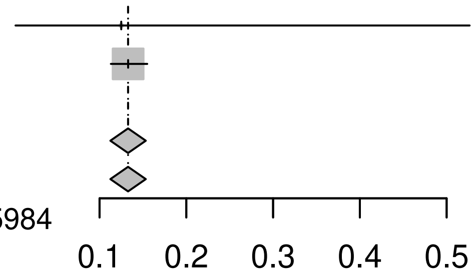

Anaplasma marginale

Study	Events	Total	Proportion	95%-CI	Weight (common)	Weight (random)
Ghafar et al. (2020)	11	90	0.122222	[0.062621; 0.208174]	1.4%	18.9%
Ashraf et al. (2021)	76	844	0.090047	[0.071602; 0.111412]	9.5%	20.3%
Fathi et al. (2023)	166	230	0.721739	[0.659033; 0.778630]	67.8%	20.5%
Patra et al. (2022)	140	1053	0.132953	[0.113020; 0.154963]	18.3%	20.4%
Ali et al. (2023)	11	19	0.578947	[0.334998; 0.797479]	3.0%	19.8%
**Common effect model**		**2236**	**0.420743**	**[0.393846; 0.449477]**	**100.0%**	**.**
**Random effects model**			**0.228859**	**[0.097482; 0.537296]**	**.**	**100.0%**

Heterogeneity: $I^2 = 99.3\%$, $\tau^2 = 0.9213$, $p < 0.000001$

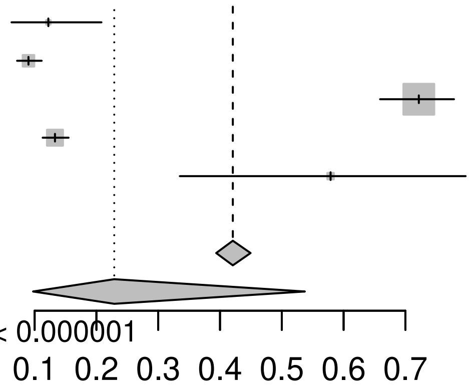

Anaplasma ovis

Study	Events	Total	Proportion	95%−CI	Weight (common)	Weight (random)
Yu et al. (2019)	22	60	0.366667	[0.245941; 0.501031]	1.8%	17.9%
Alanazi et al. (2021)	21	261	0.080460	[0.050495; 0.120362]	25.0%	21.1%
Ghafar et al. (2020)	7	14	0.500000	[0.230361; 0.769639]	0.4%	11.3%
Sadeddine et al. (2019)	3	8	0.375000	[0.085233; 0.755137]	0.2%	8.7%
Noaman et al. (2012)	50	150	0.333333	[0.258556; 0.414843]	4.8%	19.9%
Jiang et al. (2020)	174	1192	0.145973	[0.126402; 0.167308]	67.8%	21.2%
**Common effect model**		**1685**	**0.144558**	**[0.128059; 0.161056]**	**100.0%**	**.**
**Random effects model**			**0.268611**	**[0.140702; 0.396521]**	**.**	**100.0%**

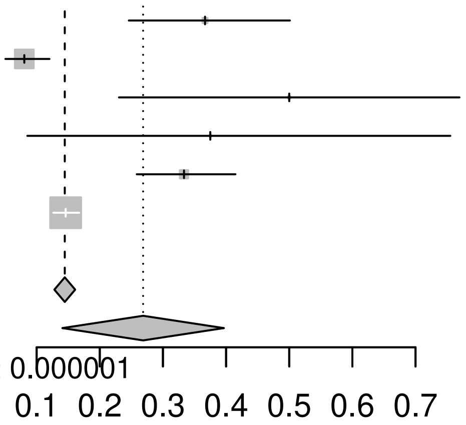

Heterogeneity: $I^2 = 91.7\%$, $\tau^2 = 0.0199$, $p < 0.000001$

Anaplasma phagocytophilum

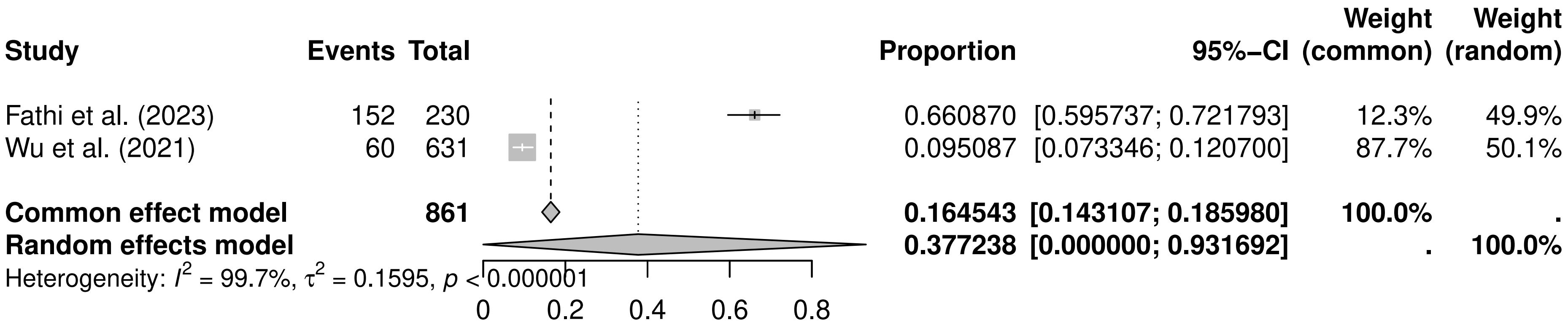

Babesia bigemina

Study	Events	Total	Proportion	95%-CI	Weight (common)	Weight (random)
Sahibi et al. (1998)	28	475	0.058947	[0.039524; 0.084071]	50.1%	41.7%
Fathi et al. (2023)	23	230	0.100000	[0.064454; 0.146266]	15.0%	22.3%
Rajabi et al. (2017)	37	450	0.082222	[0.058551; 0.111552]	34.9%	35.9%
**Common effect model**		**1155**	**0.073215**	**[0.058218; 0.088211]**	**100.0%**	**.**
**Random effects model**			**0.076483**	**[0.053965; 0.099000]**	**.**	**100.0%**

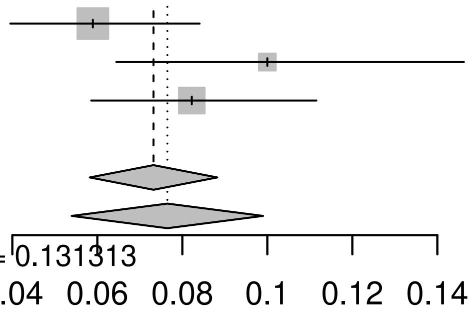

Heterogeneity: $I^2 = 50.7\%$, $\tau^2 = 0.0002$, $p = 0.131313$

Babesia caballi

Study	Events	Total	Proportion	95%−CI	Weight (common)	Weight (random)
Erkin et al. (2015)	3	215	0.013953	[0.002887; 0.040234]	42.3%	37.9%
Tursun et al. (2016)	8	78	0.102564	[0.045331; 0.192127]	2.3%	23.8%
Abedi et al. (2013)	0	100	0.000000	[0.000000; 0.036217]	55.4%	38.2%
**Common effect model**		**393**	**0.008247**	**[0.000000; 0.018440]**	**100.0%**	**.**
**Random effects model**			**0.029722**	**[0.000000; 0.082102]**	**.**	**100.0%**

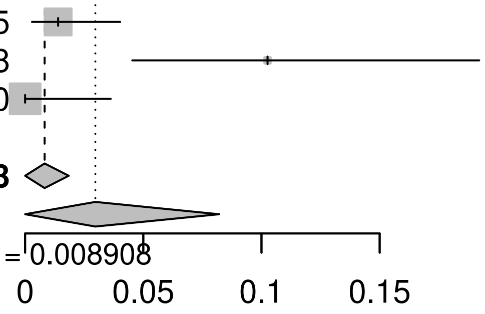

Heterogeneity: $I^2 = 78.8\%$, $\tau^2 = 0.0018$, $p = 0.008908$

Babesia motasi

Study	Events	Total	Proportion	95%–CI	Weight (common)	Weight (random)
Razmi et al. (2003)	4	776	0.005155	[0.001406; 0.013145]	88.8%	52.3%
Razmi et al. (2002)	25	677	0.036928	[0.024038; 0.054032]	11.2%	47.7%
**Common effect model**		**1453**	**0.008705**	**[0.003956; 0.013454]**	**100.0%**	**.**
**Random effects model**			**0.020318**	**[0.000000; 0.051423]**	**.**	**100.0%**

Heterogeneity: $I^2 = 94.1\%$, $\tau^2 = 0.0005$, $p = 0.000036$

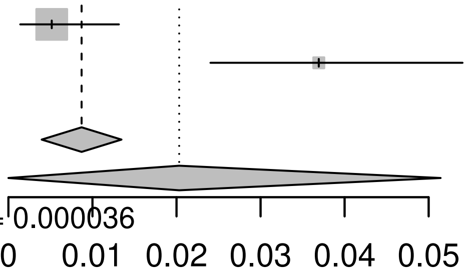

Babesia ovis

Study	Events	Total	Proportion	95%-CI	Weight (common)	Weight (random)
Sahibi et al. (1998)	79	475	0.166316	[0.133942; 0.202910]	14.7%	24.8%
Ozubek et al. (2016)	32	590	0.054237	[0.037390; 0.075707]	49.2%	25.4%
Razmi et al. (2003)	146	776	0.188144	[0.161229; 0.217453]	21.7%	25.1%
Razmi et al. (2002)	188	677	0.277696	[0.244252; 0.313091]	14.4%	24.8%
**Common effect model**		**2518**	**0.132005**	**[0.119188; 0.144822]**	**100.0%**	**.**
**Random effects model**			**0.170920**	**[0.080477; 0.261363]**	**.**	**100.0%**

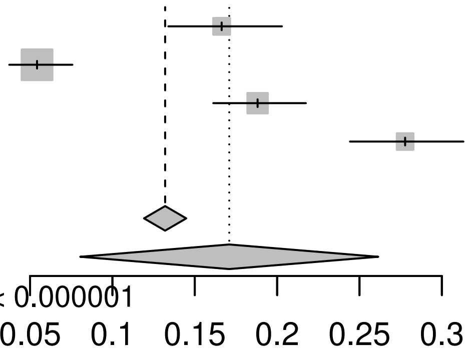

Heterogeneity: $I^2 = 98.1\%$, $\tau^2 = 0.0083$, $p < 0.000001$

uncharacterised *Babesia*

Study	Events	Total	Proportion	95%-CI	Weight (common)	Weight (random)
Alanazi et al. (2021)	0	724	0.000000	[0.000000; 0.005082]	97.6%	28.2%
Hasheminasab et al. (2018)	511	3056	0.167212	[0.154141; 0.180923]	2.0%	28.2%
Al–Hosary et al. (2018)	513	1068	0.480337	[0.449995; 0.510788]	0.4%	28.2%
Guan et al. (2001)	1	1	1.000000	[0.025000; 1.000000]	0.0%	15.4%
**Common effect model**		**4849**	**0.005319**	**[0.003432; 0.007207]**	**100.0%**	**.**
**Random effects model**			**0.336272**	**[0.000000; 0.684994]**	**.**	**100.0%**

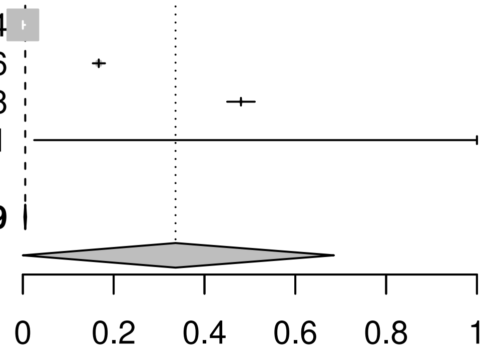

Heterogeneity: $I^2 = 99.8\%$, $\tau^2 = 0.1121$, $p = 0$

Coxiella burnetii

Study	Events	Total	Proportion	95%−CI	Weight (common)	Weight (random)
Dhaka et al. (2022)	87	146	0.595890	[0.511591; 0.676217]	0.3%	32.9%
Patra et al. (2020)	5	1053	0.004748	[0.001544; 0.011046]	97.5%	33.6%
Ghazanabad et al. (2023)	218	927	0.235167	[0.208204; 0.263825]	2.3%	33.5%
**Common effect model**		**2126**	**0.011512**	**[0.007412; 0.015611]**	**100.0%**	**.**
**Random effects model**			**0.276669**	**[0.000000; 0.612592]**	**.**	**100.0%**

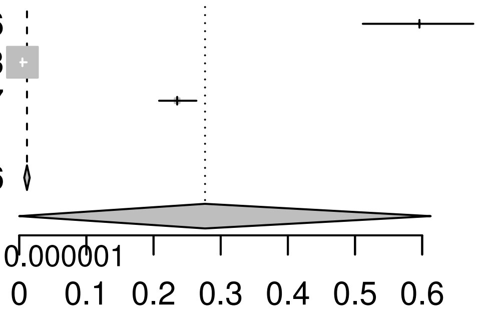

Heterogeneity: $I^2 = 99.6\%$, $\tau^2 = 0.0875$, $p < 0.000001$

Theileria annulata

Study	Events	Total	Proportion	95%−CI	Weight (common)	Weight (random)
Inci et al. (2008)	374	554	0.675090	[0.634332; 0.713969]	6.0%	4.2%
Abaker et al. (2017)	39	150	0.260000	[0.191876; 0.337857]	1.6%	4.2%
Aktas et al. (2006)	99	252	0.392857	[0.332154; 0.456112]	2.7%	4.2%
Al−Hosary et al. (2018)	679	1068	0.635768	[0.606097; 0.664688]	11.6%	4.2%
Dumanli et al. (2005)	277	426	0.650235	[0.602853; 0.695513]	4.6%	4.2%
Samiurahman et al. (2019)	74	100	0.740000	[0.642688; 0.822606]	1.1%	4.2%
Guo et al. (2017)	408	2571	0.158693	[0.144770; 0.173399]	28.0%	4.2%
He et al. (2015)	60	104	0.576923	[0.476131; 0.673219]	1.1%	4.2%
Liu et al. (2018)	11	16	0.687500	[0.413379; 0.889830]	0.2%	3.7%
Wu et al. (2011)	30	50	0.600000	[0.451794; 0.735922]	0.5%	4.1%
Zhang et al. (2021)	42	568	0.073944	[0.053809; 0.098638]	6.2%	4.2%
Parveen et al. (2021)	174	844	0.206161	[0.179341; 0.235051]	9.2%	4.2%
Fathi et al. (2023)	157	230	0.682609	[0.618207; 0.742230]	2.5%	4.2%
Arjmand et al. (2016)	22	138	0.159420	[0.102687; 0.231357]	1.5%	4.2%
Alanazi et al. (2021)	7	228	0.030702	[0.012431; 0.062232]	2.5%	4.2%
Luo et al. (2020)	38	303	0.125413	[0.090299; 0.168054]	3.3%	4.2%
Wang et al. (2009)	44	50	0.880000	[0.756899; 0.954665]	0.5%	4.1%
Alimijiang et al. (2012)	10	30	0.333333	[0.172874; 0.528120]	0.3%	4.0%
Du et al. (2018)	78	304	0.256579	[0.208424; 0.309548]	3.3%	4.2%
Sajid et al. (2023)	139	328	0.423780	[0.369680; 0.479270]	3.6%	4.2%
Ozubek et al. (2016)	8	257	0.031128	[0.013533; 0.060412]	2.8%	4.2%
Rahmani−Varmale et al. (2019)	0	400	0.000000	[0.000000; 0.009180]	4.4%	4.2%
Razmi et al. (2013)	8	119	0.067227	[0.029469; 0.128170]	1.3%	4.2%
Nangru et al. (2022)	2	91	0.021978	[0.002673; 0.077145]	1.0%	4.1%
**Common effect model**		**9181**	**0.270828**	**[0.261787; 0.279966]**	**100.0%**	**.**
**Random effects model**			**0.321410**	**[0.202537; 0.453407]**	**.**	**100.0%**

Heterogeneity: $I^2 = 99.2\%$, $\tau^2 = 0.1132$, $p = 0$

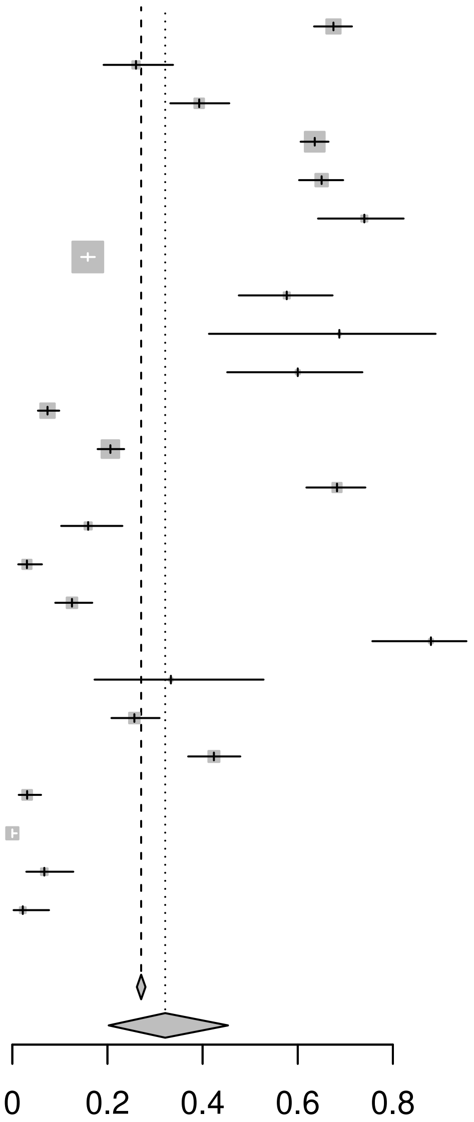

Theileria lestoquardi

Study	Events	Total	Proportion	95%−CI	Weight (common)	Weight (random)
Hasheminasab et al. (2018)	779	3056	0.254908	[0.239534; 0.270753]	49.7%	12.0%
Rahmani−Varmale et al. (2019)	8	160	0.050000	[0.021831; 0.096141]	10.4%	11.9%
Taha et al. (2011)	10	16	0.625000	[0.354346; 0.848016]	0.2%	7.0%
Mohammadi et al. (2017)	25	400	0.062500	[0.040851; 0.090882]	21.1%	12.0%
Magzoub et al. (2020)	26	200	0.130000	[0.086708; 0.184652]	5.5%	11.8%
Razmi et al. (2013)	10	150	0.066667	[0.032430; 0.119184]	7.4%	11.8%
Razmi et al. (2003)	68	188	0.361702	[0.293039; 0.434803]	2.5%	11.4%
Nangru et al. (2022)	21	91	0.230769	[0.148882; 0.330866]	1.6%	11.0%
Zarei et al. (2017)	16	80	0.200000	[0.118859; 0.304369]	1.5%	11.0%
**Common effect model**		**4341**	**0.174375**	**[0.163480; 0.185271]**	**100.0%**	**.**
**Random effects model**			**0.200036**	**[0.102420; 0.297652]**	**.**	**100.0%**

Heterogeneity: $I^2 = 97.5\%$, $\tau^2 = 0.0205$, $p < 0.000001$

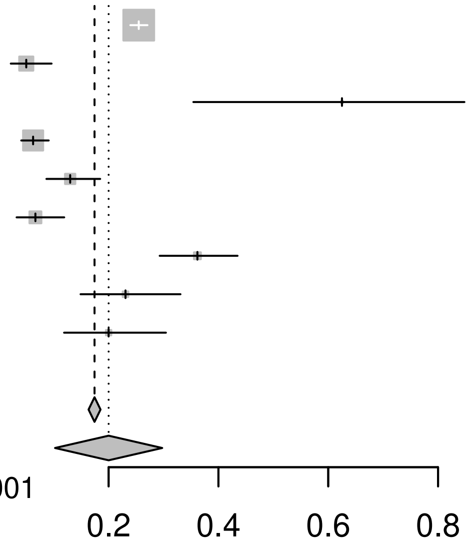

Theileria luwenshuni

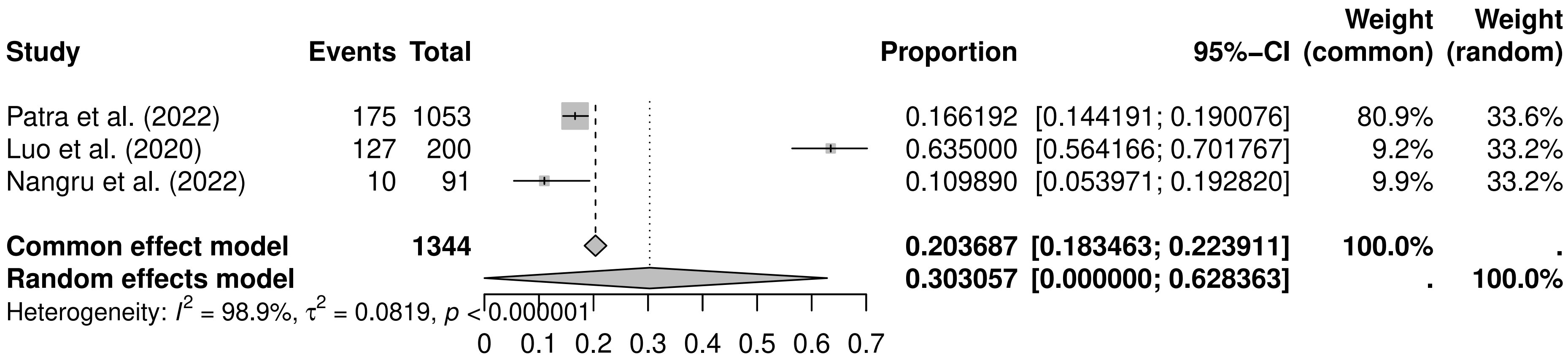

Theileria ovis

Study	Events	Total	Proportion	95%−CI	Weight (common)	Weight (random)
Alanazi et al. (2021)	2	228	0.008772	[0.001064; 0.031326]	78.7%	17.1%
Rahmani−Varmale et al. (2019)	58	400	0.145000	[0.111981; 0.183378]	9.7%	17.0%
Ozubek et al. (2016)	209	590	0.354237	[0.315610; 0.394332]	7.7%	17.0%
Razmi et al. (2013)	88	150	0.586667	[0.503469; 0.666372]	1.9%	16.6%
Nangru et al. (2022)	46	91	0.505495	[0.398619; 0.612003]	1.1%	16.2%
Zarei et al. (2017)	34	80	0.425000	[0.315110; 0.540611]	1.0%	16.1%
**Common effect model**		**1539**	**0.068918**	**[0.058183; 0.079652]**	**100.0%**	**.**
**Random effects model**			**0.333935**	**[0.155790; 0.512080]**	**.**	**100.0%**

Heterogeneity: $I^2 = 99.2\%$, $\tau^2 = 0.0482$, $p < 0.000001$

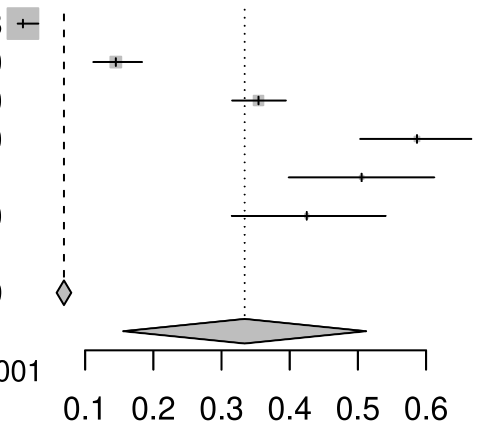

uncharacterised *Theileria*

Study	Events	Total	Proportion	95%–CI
Aktas et al. (2006)	18	252	0.071429	[0.042880; 0.110540]
Arjmand et al. (2014)	22	138	0.159420	[0.102687; 0.231357]
Hayati et al. (2015)	7	60	0.116667	[0.048215; 0.225716]
Hayati et al. (2020)	33	200	0.165000	[0.116383; 0.223843]
Ghafar et al. (2020)	8	14	0.571429	[0.288609; 0.823389]
Nangru et al. (2022)	210	810	0.259259	[0.229385; 0.290897]
Razmi et al. (2006)	100	840	0.119048	[0.097919; 0.142889]
Nasser et al. (2014)	19	150	0.126667	[0.078015; 0.190721]
**Common effect model**		**2464**	**0.169237**	**[0.154943; 0.184562]**
**Random effects model**			**0.159780**	**[0.107689; 0.230559]**

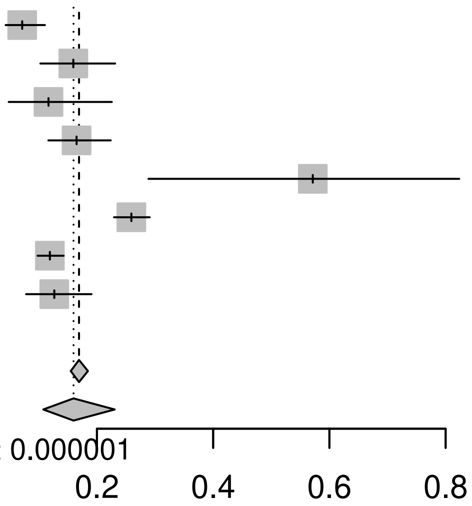

Heterogeneity: $I^2 = 92.2\%$, $\tau^2 = 0.3537$, $p < 0.000001$

Crimean-Congo haemorrhagic fever virus

Study	Events	Total	Proportion	95%-CI	Weight (common)	Weight (random)
Williams et al. (2000)	91	380	0.239474	[0.197424; 0.285634]	88.6%	88.6%
Telmadarraiy et al. (2008)	15	54	0.277778	[0.164555; 0.416435]	11.4%	11.4%
**Common effect model**		**434**	**0.243851**	**[0.203468; 0.284233]**	**100.0%**	**.**
**Random effects model**			**0.243851**	**[0.203468; 0.284233]**	**.**	**100.0%**

Heterogeneity: $I^2 = 0.0\%$, $\tau^2 = 0$, $p = 0.554227$

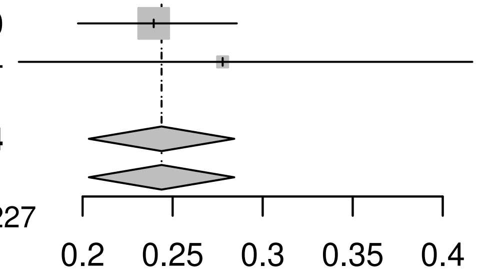

Theileria equi

Study	Events	Total	Proportion	95%−CI	Weight (common)	Weight (random)
Abedi et al. (2013)	45	100	0.450000	[0.350320; 0.552720]	51.2%	50.2%
Tursun et al. (2016)	22	78	0.282051	[0.185938; 0.395331]	48.8%	49.8%
**Common effect model**		**178**	**0.368032**	**[0.298265; 0.437798]**	**100.0%**	**.**
**Random effects model**			**0.366386**	**[0.201801; 0.530971]**	**.**	**100.0%**

Heterogeneity: $I^2 = 82.0\%$, $\tau^2 = 0.0116$, $p = 0.018352$

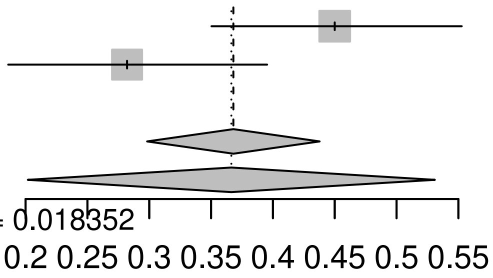

Figure S8: The results of Maxent model for *H. anatolicum*

Receiver operating characteristic (ROC) curve of the Maxent model for *H. anatolicum*. The ROC curve averaged over the 25 replicate runs. The specificity is defined using predicted area

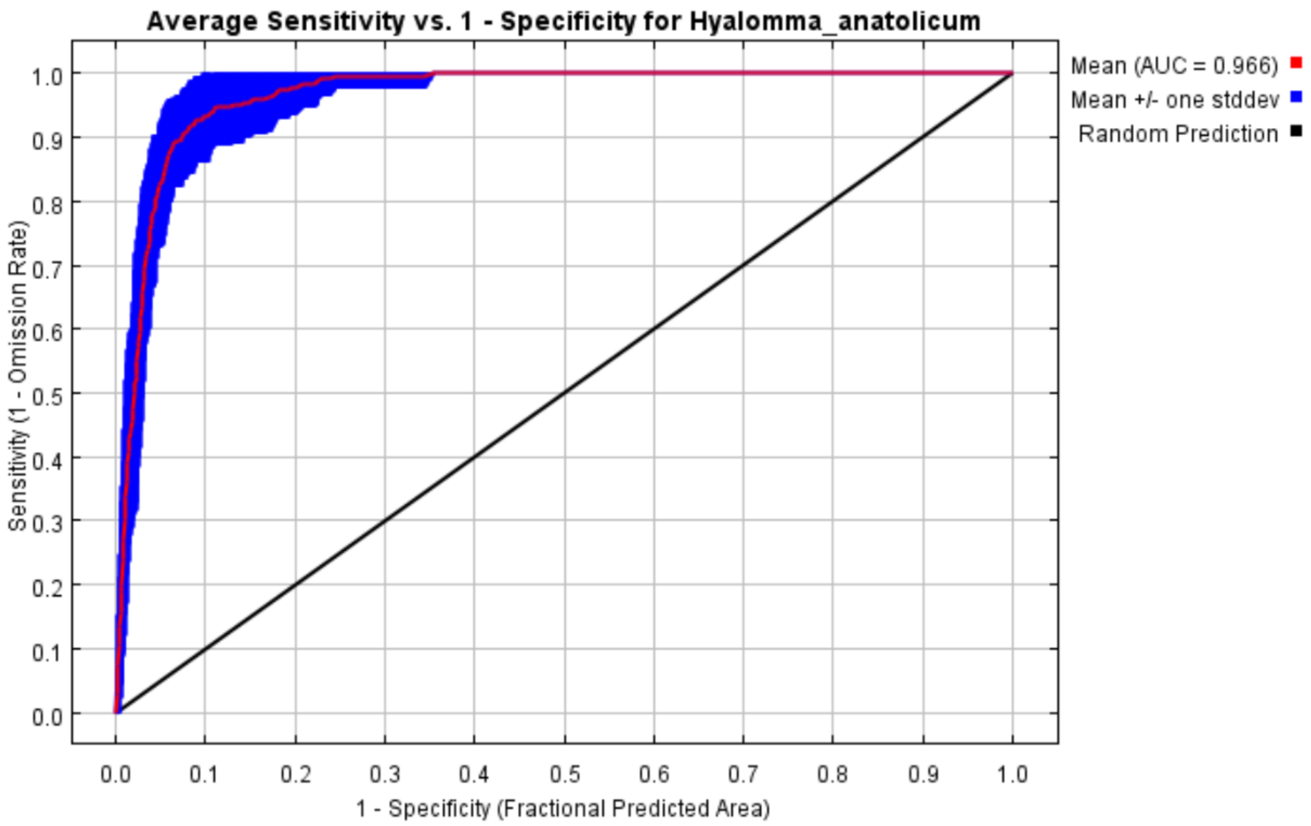

Figure S9: Jackknife plots of Maxent model for *H. anatolicum*

prediction

A. Trai-ning gain plot B. test gain plot C. AUC plot

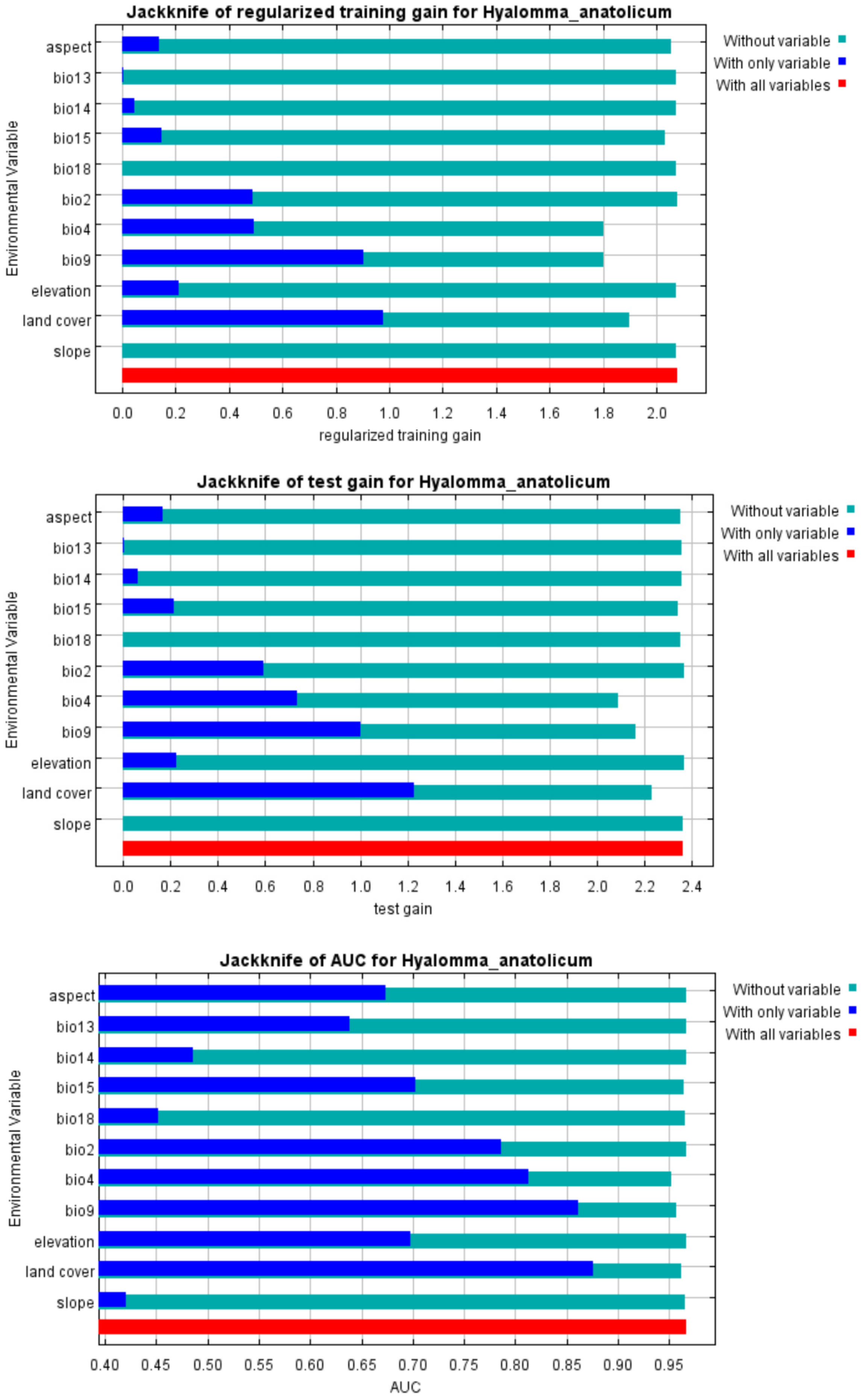

Table S8: Relative contributions of the environmental and meteorological variables to the Maxent model

Variable	Percent contribution	Permutation importance
BIO9	41.8	73.6
Land cover	36.1	4.8
BIO4	10.2	16.7
BIO15	3.7	3.1
BIO2	3.3	0
Aspect	2.8	0.3
BIO13	1	0.3
Slope	0.4	0.2
BIO18	0.4	0.5
BIO14	0.2	0.3
Elevation	0.1	0.2

Figure S10: Response curves of environmental variables to probability of *H. anatolicum* presence
The curves show the mean response of 25 replicate MaxEnt runs (red line) and standard deviation (blue shades)

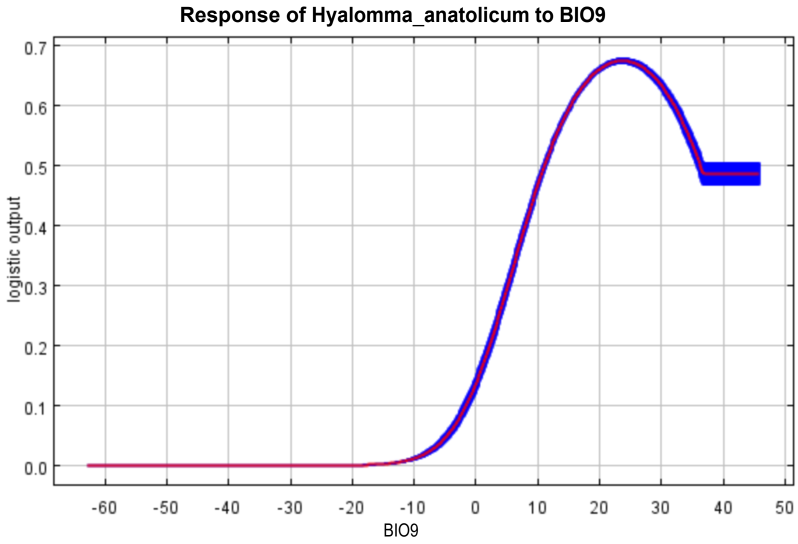

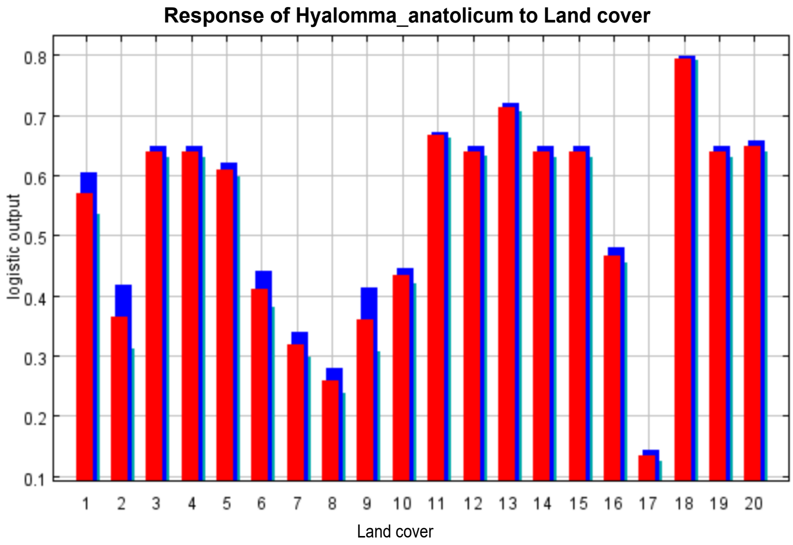

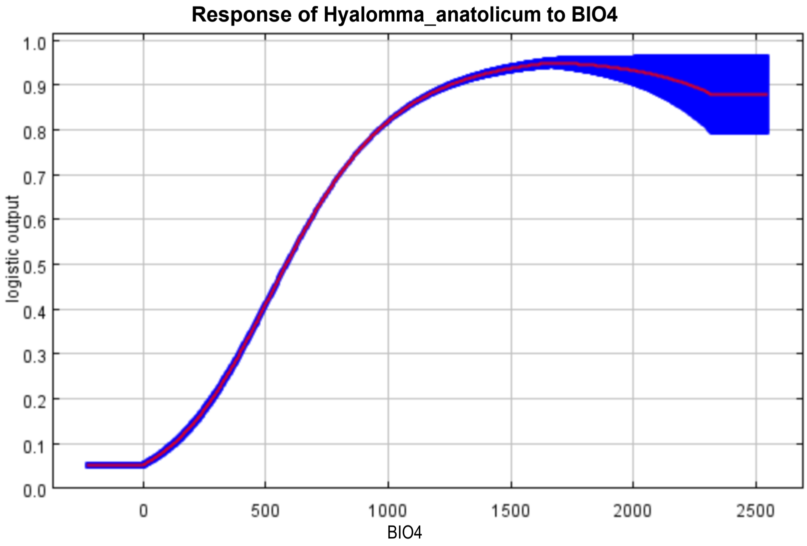

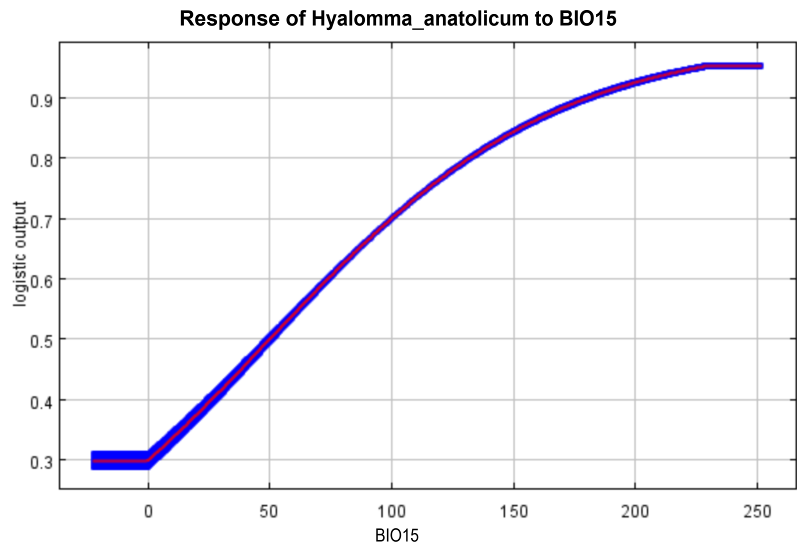
Response of Hyalomma_anatolicum to BIO15
logistic output
0.3
0.4
0.5
0.6
0.7
0.8
0.9
0
50
100
150
200
250
BIO15

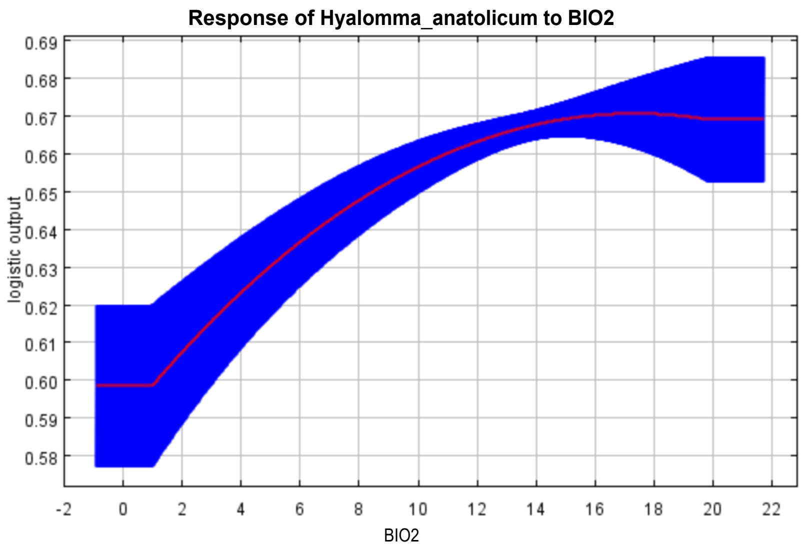
Response of Hyalomma_anatolicum to BIO2
logistic output
0.58
0.59
0.60
0.61
0.62
0.63
0.64
0.65
0.66
0.67
0.68
0.69
-2
0
2
4
6
8
10
12
14
16
18
20
22
BIO2

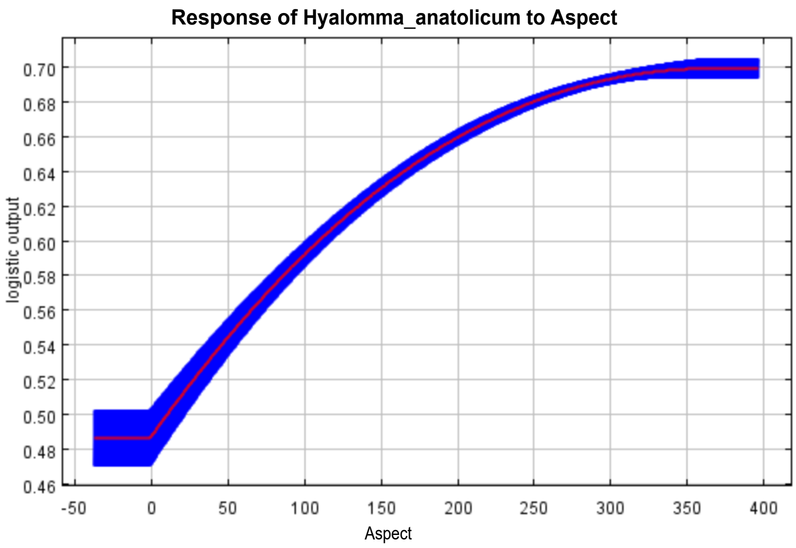
Response of Hyalomma_anatolicum to Aspect
logistic output
0.46
0.48
0.50
0.52
0.54
0.56
0.58
0.60
0.62
0.64
0.66
0.68
0.70
-50
0
50
100
150
200
250
300
350
400
Aspect

Response of Hyalomma_anatolicum to BIO13

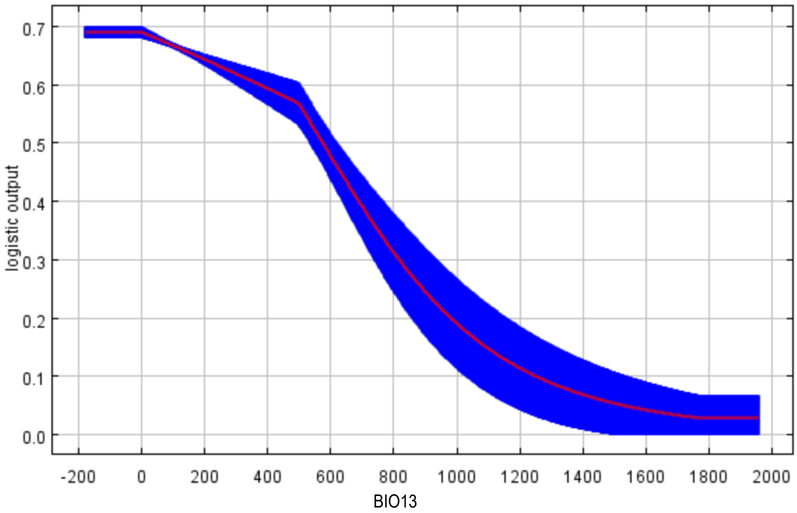

Response of Hyalomma_anatolicum to Slope

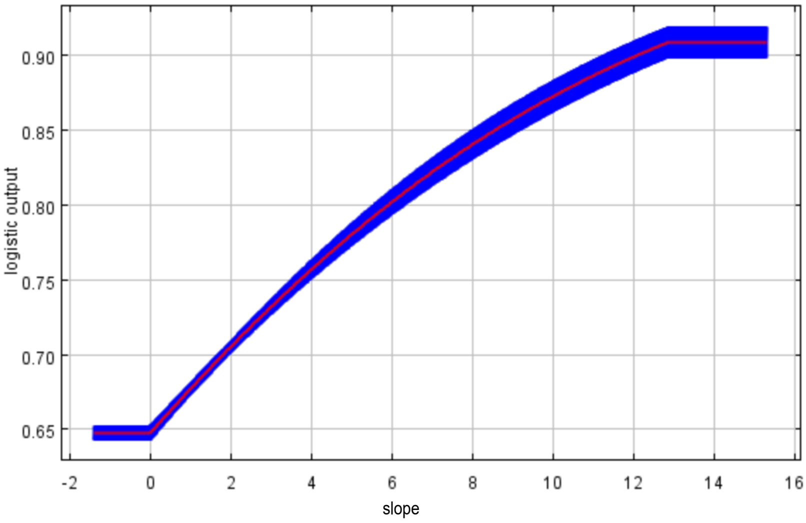

Response of Hyalomma_anatolicum to BIO18

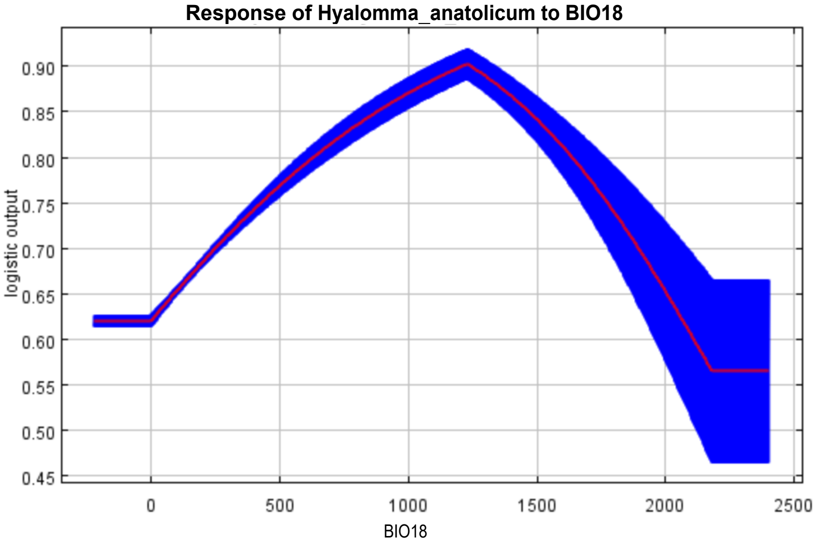

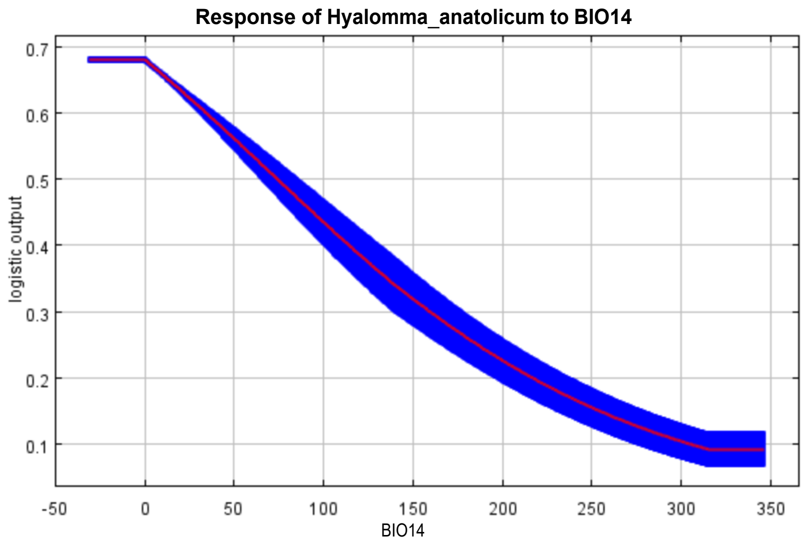
Response of Hyalomma_anatolicum to BIO14
logistic output
BIO14
0.7
0.6
0.5
0.4
0.3
0.2
0.1
-50
0
50
100
150
200
250
300
350

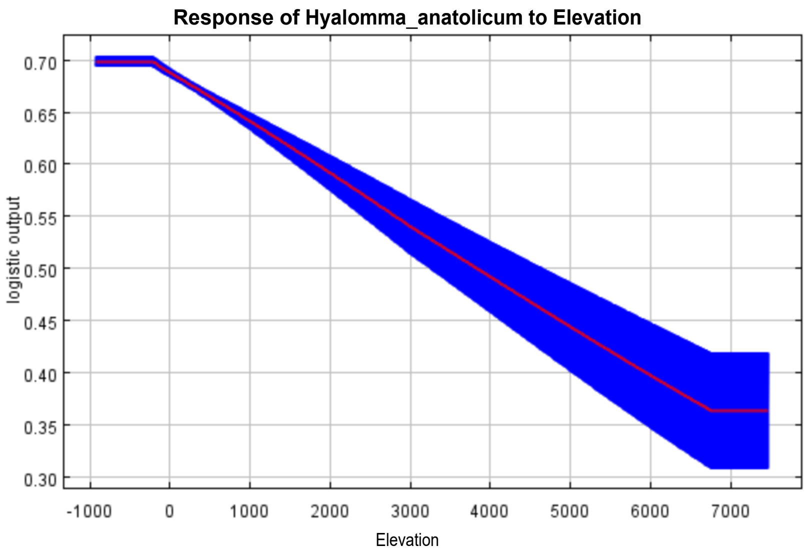
Response of Hyalomma_anatolicum to Elevation
logistic output
Elevation
0.70
0.65
0.60
0.55
0.50
0.45
0.40
0.35
0.30
-1000
0
1000
2000
3000
4000
5000
6000
7000
